# Biocatalysis versus Molecular Recognition in Sialoside-Selective Neuraminidase Biosensing

**DOI:** 10.1021/acschembio.2c00913

**Published:** 2023-02-15

**Authors:** Israel Alshanski, Suraj Toraskar, Ariel Shitrit, Daniel Gordon-Levitan, Prashant Jain, Raghavendra Kikkeri, Mattan Hurevich, Shlomo Yitzchaik

**Affiliations:** † The Institute of Chemistry and Center of Nanotechnology, The Hebrew University of Jerusalem, Jerusalem 91904, Israel; ‡ Indian Institute of Science Education and Research, Dr. Homi Bhabha Road, Pune 411008, India

## Abstract

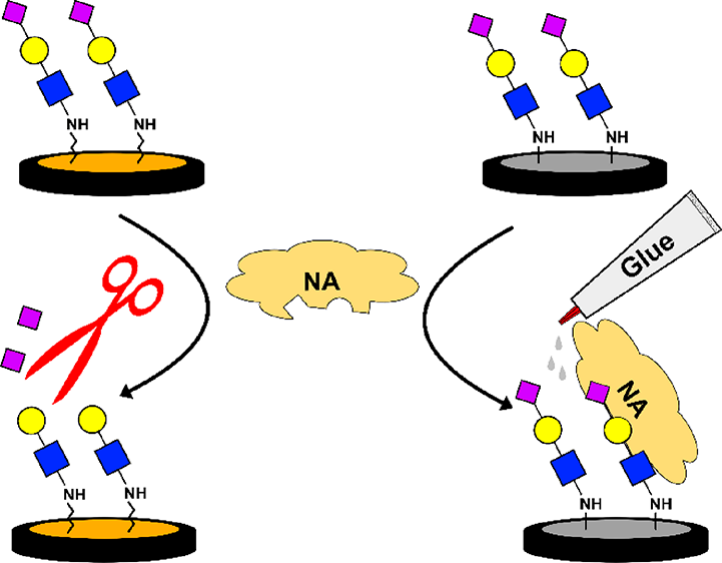

Sialic acid recognition and hydrolysis are essential parts of cellular function and pathogen infectivity. Neuraminidases are enzymes that detach sialic acid from sialosides, and their inhibition is a prime target for viral infection treatment. The connectivity and type of sialic acid influence the recognition and hydrolysis activity of the many different neuraminidases. The common strategies to evaluate neuraminidase activity, recognition, and inhibition rely on extensive labeling and require a large amount of sialylated glycans. The above limitations make the effort of finding viral inhibitors extremely difficult. We used synthetic sialylated glycans and developed a label-free electrochemical method to show that sialoside structural features lead to selective neuraminidase biosensing. We compared Neu5Ac to Neu5Gc sialosides to evaluate the organism-dependent neuraminidase selectivity–sensitivity relationship. We demonstrated that the type of surface and the glycan monolayer density direct the response to either binding or enzymatic activity. We proved that while the hydrophobic glassy carbon surface increases the interaction with the enzyme hydrophobic interface, the negatively charged interface of the lipoic acid monolayer on gold repels the protein and enables biocatalysis. We showed that the sialoside monolayers can serve as tools to evaluate the inhibition of neuraminidases both by biocatalysis and molecular recognition.

## Introduction

Sialic acid (SA) is an important and unique monosaccharide that decorates *N*-glycans, *O*-glycans, gangliosides, and even RNA on the cell membrane (sialosides).^1−4^ SA is important for cell recognition, intercellular communication, and immune system regulation.^2^ SA is a common target for infection.^5−7^ Neuraminidases (NAs) are enzymes that remove SA from sialosides, therefore regulating SA expression.^8,9^ Viral pathogens use NAs or similar proteins as part of the infection process.^10−12,11^ Viral pathogens can differentiate between cells based on the type of SA and the specific glycoconjugate connectivity.^13,14^

There are a few common approaches for the evaluation of the NA activity. The first approach is based on substrate labeling. In this case, the substrate can be either fluorescently or metabolically labeled.^15−17^ The labeled substrate undergoes enzymatic reaction or binds the enzyme in a manner that produces a detectable signal, e.g., fluorescence.^17^ However, this method requires large quantities of the labeled substrate and hence is in limited use for hardly accessible sialosides because their synthesis or isolation from natural sources is not trivial.^18^ The second approach requires an inhibitor that binds the catalytic site and enables binding screening. In this approach, the binding properties of the enzyme can be studied in a glycan array, which enables fingerprint patterning.^13^ The third approach is the use of a label-free sensory interface. In this case, the substrate is attached to an interface, and a signal is produced upon enzyme binding or reaction.^19−21^

Electrochemical impedance spectroscopy (EIS) is a label-free electrochemical technique for the evaluation of interactions and biosensing.^21−25^ EIS relies on changes to the interfacial properties, which affect the diffusion through the layer when external RedOx active species is used.^21−24,26^ EIS is a sensitive technique that requires small amounts of material to produce a detectable signal in the sensory layer.^18,27^ The high sensitivity of EIS can be used for the evaluation of enzymatic reactions or protein binding to a substrate containing monolayer in the various interfaces.^28−31^

Sensors for enzymes operate on two principles. In the first approach, the enzyme is anchored to the electrode and the substrate (or inhibitor) is the analyte. The other approach relies on an anchored substrate monolayer on the electrode, and the enzyme is the analyte. Previous work showed that both the binding of the substrate and the catalytic reaction can be monitored by the surface-immobilized enzyme.^32^ In this case, the enzymatic reaction produced a change in pH and substrate–enzyme binding that resulted in dipole change, which affected the measured signal on a field effect transistor. A mathematic model was able to distinguish between the contribution of the reaction and binding to the measured signal. In the case of the substrate anchoring to the electrode, it was shown that the activity of a kinase enzyme can be detected by impedimetric measurements in the presence of a co-factor.^33^ By removing the co-factor from the system, binding of the specific kinase to a substrate can be detected rather than activity.^31^ Another approach for the detection of a kinase enzyme is by targeting the allosteric inhibitory site for impedimetric detection.^30^ Additionally, the ability to study the binding and catalysis of enzymes by EIS relies on the chemistry of the interface.^22^ These studies demonstrate that EIS might be used for molecular recognition and biocatalysis that can be applied to study sialoside-selective NA biosensing.

**Figure 1:**
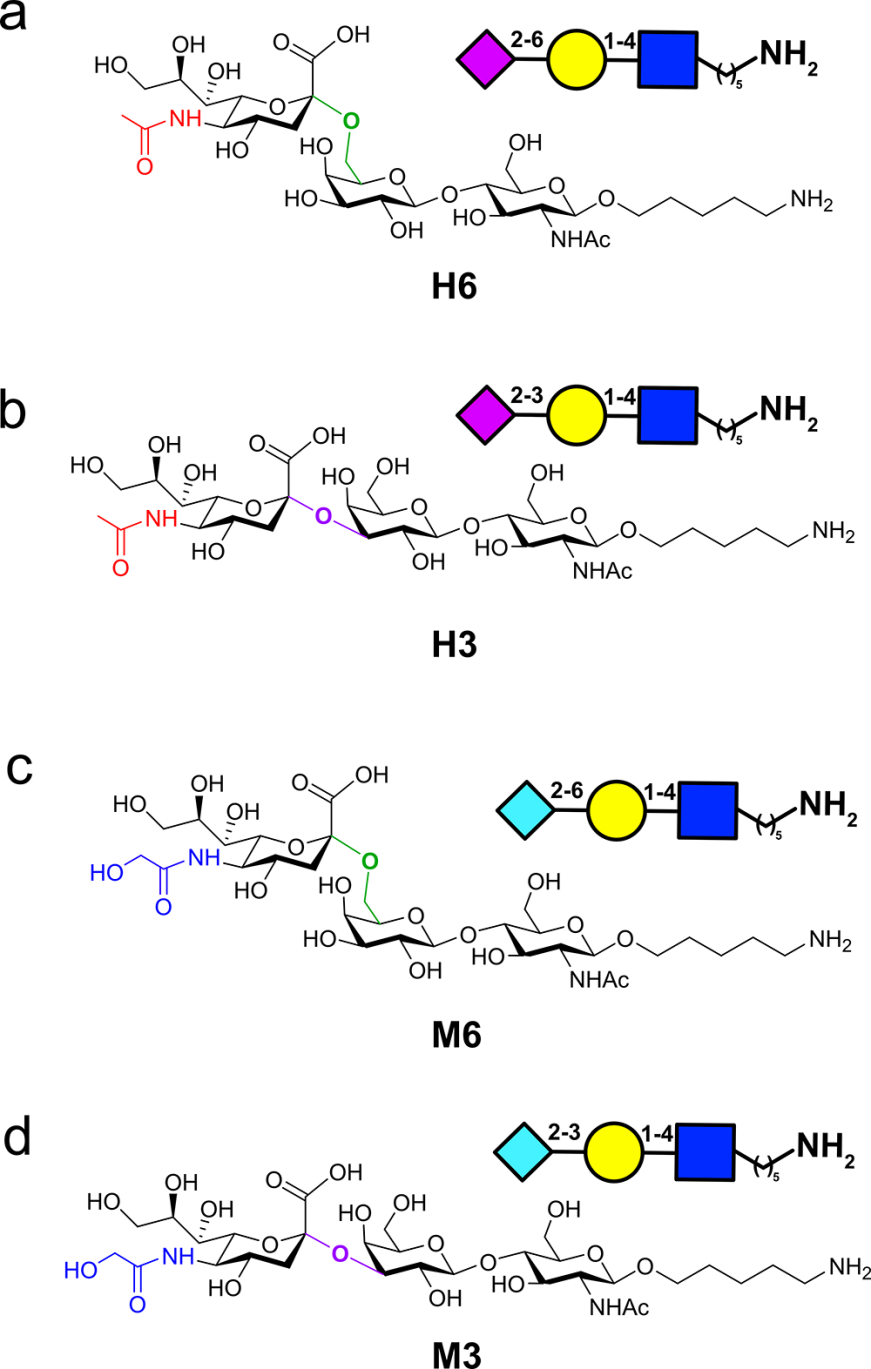
Trisaccharides that were used in this study with the structure and their respective symbol nomenclatures for glycan code: (a) 2,6-Neu5Ac-Gal-GlcNAc trisaccharide (**H6**), (b) 2,3-Neu5Ac-Gal-GlcNAc trisaccharide (**H3**), (c) 2,6-Neu5Gc-Gal-GlcNAc trisaccharide (**M6**), and (d) 2,3-Neu5Gc-Gal-GlcNAc trisaccharide (**M3**). Red is the acetyl in **H3** and **H6**, blue is the glycollate in **M3** and **M6**, green is the 2,6-glycosidic bond, and purple is the 2,3-glycosidic bond.

Previously, we showed a platform based on bi-antennary *N*-glycan that enables impedimetric biosensing of sialylation and desialylation processes.^29^ However, that platform required time-consuming multistep modification on the oxide layer of the glassy carbon electrode (GCE). Herein, four sialylated trisaccharides substrates were synthesized with an amine at the terminus on the reducing end to enable surface anchoring. Synthetic sialosides proved very useful for developing glycan-based applications.^34,35^ The sialoside in the library differs by the sialic acid type, Neu5Ac and Neu5Gc, and regiochemistry, 2,3 and 2,6 (Figure 1). These saccharides were attached to either the GCE by a single-step electrochemical grafting or the Au electrode (AuE) by amidation reaction with a lipoic acid-based monolayer. The modifications were characterized by EIS, contact potential difference (CPD), contact angle (CA), variable angle ellipsometry (VASE), and X-ray photoelectron spectroscopy (XPS). The modified GCEs and AuEs were exposed to two types of bacterial NA to determine preferential response. To test our system, we choose to use commercial NAs with a known sialoside linkage specificity. The first type of NA used is preferential to 2,3-sialosides and originated from *Clostridium perfringens* (*3NACP*),^36^ while the second type is preferential to 2,6-sialosides and originated from *Arthrobacter ureafaciens* (*6NAAU*).^37^ Surface characterizations were used to elucidate if the signal arises from enzymatic activity or binding after exposure to the enzyme. Additionally, the effect of a NA inhibitor on binding and activity was examined.

## Results and Discussion

Various types of NA can be differentiated by preferential activity with the sialylated substrate. Four trisaccharide substrates were synthesized via multistep processes to allow high control of the regiochemistry on common core sialosides (Figure 1). The four substrates contain the same core structure of 6-(β-d-Gal-(1-4)-β-d-GlcNAc-(1-4))-1-amine with two types of sialic acid and two types of connectivity. There are two human sialosides with acetyl at position 5, namely, Neu5Ac, with connectivity 2,6 (**H6**) and 2,3 (**H3**), and there are two monkey-type sialosides with hydroxy acetyl at position 5, namely, Neu5Gc with connectivity 2,6 (**M6**) and 2,3 (**M3**) (Figure 1).

### Sialoside Synthesis

The chemical synthesis of sialic acid glycans is a formidable synthetic challenge due to its instability, difficulties in α-glycosylation, and low reactivity. Previous studies evaluated the optimized sialylation conditions to synthesize sialylated glycans.^38−41^ Using these strategies, two Neu5Ac analogues (**H6** and **H3**) were synthesized utilizing the SA donor 13 (Schemes S6 and S7). However, the synthesis of Neu5Gc glycans using these methods is still a challenging task. Therefore, an enzymatic method has been extensively used in the synthesis of complex sialylated glycans.^42^ In the present synthetic strategy, we adopted two key steps to synthesize Neu5Gc glycans: (a) we constructed the sialic acid glycans using allyl ester instead of the traditional methyl ester-ligand to avoid harsh deprotection conditions, which may cleave α-sialyl linkage; (b) we have employed a labile method to deprotect the oxazolidinone ring to control the selective *N*-glycolyl substitution.

**Scheme 1:**
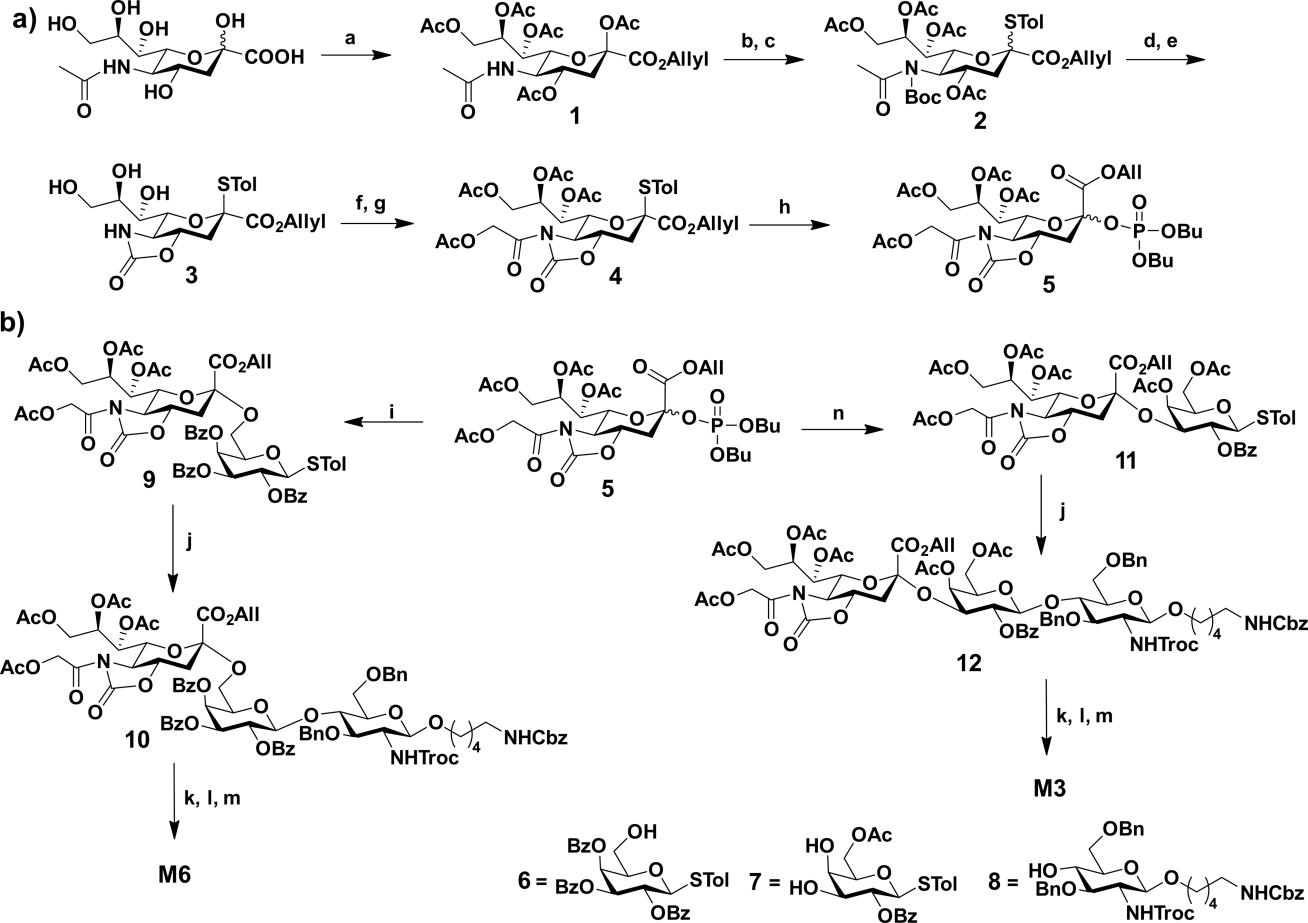
(a) Synthesis of Donor **5**. Reagents and Conditions: (a) (i) Ac_2_O, Pyridine, rt, 12 h; (ii) Cs_2_CO_3_, AllylBr, DMF, 40 °C, 4 h, 69% over Two Steps; (b) *p*-Thiocresol, BF_3_·OEt_2_, CH_2_Cl_2_, rt, 24 h, 78%; (c) Boc_2_O, DMAP, THF, 60 °C, 4 h, 85%; (d) NaOMe, AllylOH, rt, 4 h, 56%; (e) (i) TFA/CH_2_Cl_2_ (1:1, v/v), rt, 3 h; (ii) NO_2_C_6_H_4_OCOCl, NaHCO_3_, H_2_O/MeCN (2:1, v/v), 0 °C, 4 h, 47% over two steps; (f) Ac_2_O, Pyridine, rt, 12 h, 84%; (g) Acetoxyacetyl Chloride, DIPEA, CH_2_Cl_2_, 0 °C to rt, 2 h, 83%; (h) NIS, TfOH, Dibutyl Phosphate, CH_2_Cl_2_, 0 °C, 6 h, 74%. (b) Synthesis of **M6** and **M3** Trisaccharides. Reagents and Conditions: (i) **6**, TMSOTf, CH_2_Cl_2_, −50 °C, 2 h, 72%; (j) **8**, NIS, TfOH, CH_2_Cl_2_, −20 °C, 2 h, **10**: 71%; **12**: 68%; (k) Zn, THF/AcOH/Ac_2_O (3:2:1, v/v), rt, 4 h; (l) 1,2-Ethanedithiol, DBU, CH_2_Cl_2_, 0 °C, 2 h; (m) (i) LiOH, THF/H_2_O/MeOH (2:2:1, v/v), rt, 12 h; (ii) Pd(OH)_2_/C, H_2_, H_2_O/MeOH (3,1, v/v), rt, 48 h; (n) (i) **7**, TMSOTf, CH_2_Cl_2_, −50 °C, 2 h; (ii) Ac_2_O, Pyridine, rt, 12 h, 49% over Two Steps

The sialic acid donor was obtained from Neu5Ac using several steps (Scheme 1a). Peracetylation and allyl-esterification of Neu5Ac provided **1** following *p*-thiocresol glycosylation and Boc-protection that provided **2**. Deacetylation and boc deprotection of **2** used sodium methoxide and trifluoroacetic acid, respectively, followed by oxazolidinone ring formation-yielded **3**. The compound **3** was peracetylated and treated with acetoxyacetyl chloride to obtain thio-donor **4**.

Finally, glycosylation of **4** with dibutyl phosphate in the presence of *N*-iodosuccinimide (NIS) and trifluoromethanesulfonic acid (TfOH) yielded the desire sialic acid donor **5** in an excellent yield (Scheme 1a).

To achieve α(2–6) and α(2–3) glycosylated sialic acid disaccharides **9** and **11**, two different galactose building blocks **6** and **7** were synthesized from d-galactose (Scheme 1b and Schemes S2 and S3). The glucose building block **8** was synthesized by using the previously reported method.^43^ The sialic acid disaccharides (**9** and **11**) were obtained by glycosylating the sialic acid donor **5** with **6** and **7** acceptors in the presence of TMSOTf at −50 °C in the DCM solvent (Scheme 1b). In the case of α-(2–3) disaccharides, the glycosylated product was again reacted with acetic anhydride to block the 4-OH group on the galactose residue. Then, glycosylation of disaccharide thio-donors (**9** and **11**) with the **8** acceptor was carried out with NIS/TfOH at −20 °C, giving the protected trisaccharide in moderate to good yield (Scheme 1b). To accomplish the final deprotected **M6** and **M3**, the correct order of deprotection is critical to obtain Neu5Gc analogues. It was found that the oxazolidinone deprotection before Troc-removal resulted in partial deprotection of Troc. In addition, the global deprotection of oxazolidinone, acetate, and benzoyl group using strong basic conditions also resulted in complete deprotection of glucose *N*-acetate. Thus, Troc-protection removal and acetylation are the first necessary steps to maintain the *N*-glycolyl group. This was followed by selective oxazolidinone deprotection using 1,2-ethanethiol and DBU mixture followed by global deprotection using lithium hydroxide and hydrogenolysis-yielded (**M6**) and (**M3**) (Scheme 1b and Schemes S4 and S5).

The trisaccharides (**H6**, **H3**, **M6**, and **M3**) were synthesized with a primary amine at the terminus of the extending linker (Figure 1). This enables electrochemical grafting on the glassy carbon electrode (GCE) or amidation of the carboxy-terminated Au electrode (AuE) that was modified with lipoic acid.^25,44,45^

### Sialoside Assembly on Glassy Carbon Surfaces

**Figure 2:**
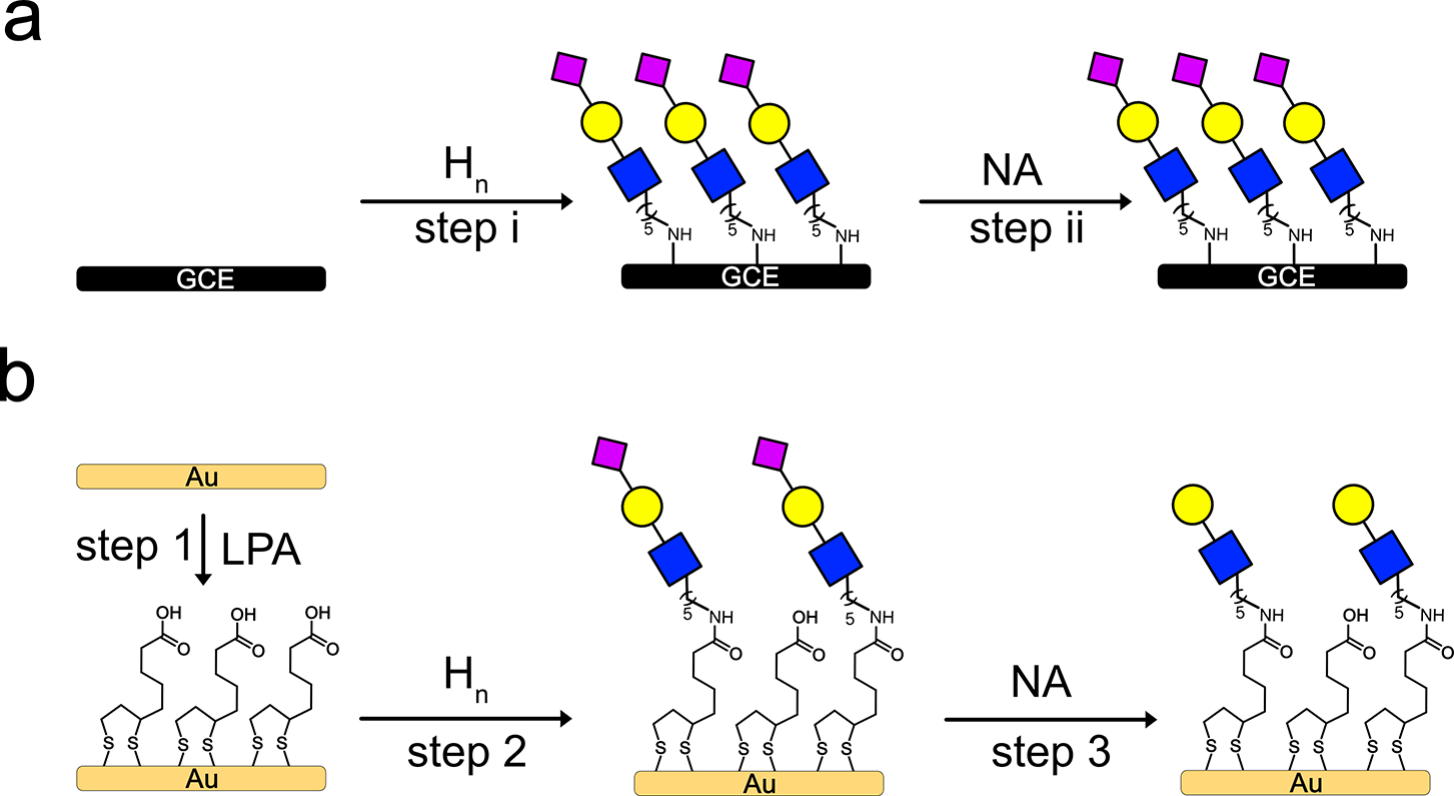
(a) (i) Electrografting process of the GCE with amino-terminated trisaccharides (demonstrated for H_n_ sialosides) applying five CV cycles of scans from 0.6 to 1.2 V at a rate of 0.01 V/s. (Step ii) incubation with NA; (b) modification of the AuE with trisaccharides: (step 1) self-assembly of LPA and (step 2) coupling the amino-terminated trisaccharide with the LPA using (1-cyano-2-ethoxy-2-oxoethylidenaminooxy)dimethylamino-morpholino-carbenium hexafluorophosphate (COMU) (demonstrated for H_n_ sialosides); (step 3) incubation with NA.

The substrates **H6**, **H3**, **M6**, and **M3** were electrochemically grafted with the GCE by applying five CV cycles with a scan rate of 0.01 V/s from 0.6 to 1.2 V referenced to the Ag/AgCl (3 M KCl) electrode to give **GCE-H6**, **GCE-H3**, **GCE-M6**, and **GCE-M3**, respectively (Figure 2a, step 1 and Figure S1). This resulted in a charge transfer resistance (*R*_CT_) increase to an approximate value of 500 Ω after the deposition suggests grafting with the glycan (Figure S1c). To support this claim, deposition with the same conditions was performed on glassy carbon plates (GCPs) with **H3** to give **GCP-H3**. These GCPs were characterized by CPD and CA analyses. A decrease in CA from 75 to 55° suggests the addition of hydrophilic molecules on the surface. The increase in *V*_CPD_ from −315 to −106 mV (ΔCPD = +209 mV) suggests the addition of negative charges, which are correlated with the deprotonated carboxylates of the sialic acid. The collective data suggest that the saccharides were electrografted on the GCE. Therefore, they can be further evaluated for impedimetric analyses.

### Sialoside Assembly on Gold Surfaces

The AuE was modified with the sialosides to form another type of sensory layer with variation in the interface because we previously showed that the interface can affect our ability to sense the enzymatic process.^22^ AuEs were modified with lipoic acid (LPA; Figure 2b, step 1) chemisorption and amidation^45^ with **H3**, **H6**, **M3**, and **M6** to provide the glycated monolayers (Figure 2b, step 2). Each step of modification showed an increase in the *R*_CT_, suggesting modification of the AuE (Figure S2). VASE analyses of the modified Au surfaces showed the formation of a monolayer with a thickness of 5 Å. XPS analysis was performed to confirm the presence of LPA on the surface and quantify the coverage by observing the binding energy of the S2p signal in the measurement. Calculation of LPA coverage based on XPS S2p/Au4f, which is 0.28, shows that there are approximately 1.6 × 10^14^ molecules/cm^2^ LPA,^46^ which is correlated to a footprint of 63 Å^2^/molecule on the Au for chemisorption. Additionally, MM2 force field calculation using Chem3D (Figure S2) indicated an LPA cross section of 30 A^2^, which correlates with an optical thickness of 5 Å. Ellipsometry and XPS indicate the same coverage, which is in line with a previous report of LPA on Au.^46^ Wettability studies showed that the addition of LPA to gold resulted in decreased CA from 87 to 60°. Surface potential analysis showed a decrease in *V*_CPD_ from +70 to −192 mV following chemisorption. The addition of the negatively charged coupling layer increases the hydrophilicity and decreases the surface potential of the gold surface. This is in line with previous works of assembly on gold.^47−49^

Coupling of the trisaccharide to LPA-Au resulted in an increase in the layer thickness to 13 Å, signifying the addition of a new glycan layer. The decrease in *V*_CPD_ to −232 mV (ΔCPD = −40 mV) suggests the addition of a sialoside layer on the AuE. Calculation of the sialoside concentration on the Au surface was performed by XPS measurements (see Section S2.3), suggesting that the coverage of sialoside is approximately 7.5 × 10^13^ molecules/cm^2^, which is correlated to a footprint of 133 Å^2^. The VASE analysis is based on calculated stretched molecule length dimensions of 33 Å (MM2 force field minimization in Chem3D; Figure S3) compared to a measured optical thickness of 13 Å, which is an addition of 8 Å compared to the LPA layer. The XPS analysis was done by comparing the signal ratio of sialoside characteristic N1s (Figure S13) to the LPA-associated S2p signal (Figure S32). The hydrophobicity of the glycated monolayer remained unchanged with a CA of 60°.

### Electrochemical Response of Human Sialosides to NAs

**Figure 3:**
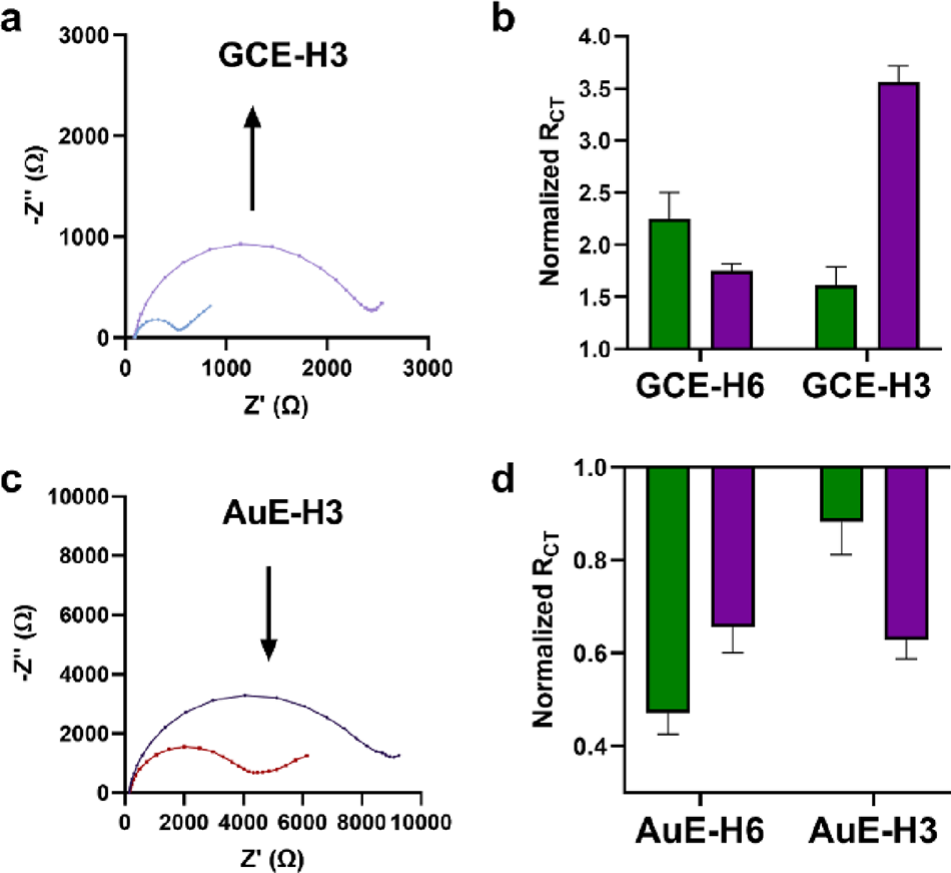
(a) Nyquist plot of impedimetric response of **GCE-H3** before (blue) and after (purple) exposure to *3NACP* (Figure 2a, step ii). (b) Normalized *R*_CT_ for the response of **GCE-H6** and **GCE-H3** after exposure to 3 mU/mL *6NAAU* (green) or *3NACP* (purple). (c) Nyquist plot of impedimetric response of **AuE-H3** before (purple) and after (red) exposure to *3NACP* (Figure 2b, step 3). (d) Normalized *R*_CT_ for the response of **AuE-H6** and **AuE-H3** after exposure to 3 mU/mL *6NAAU* (green) or *3NACP* (purple). In panels (b, d), the normalized *R*_CT_ (NR_CT_) was calculated by dividing the *R*_CT_ after exposure by the *R*_CT_ prior to exposure. Errors are the standard deviation of five electrodes.

EIS analyses were performed on **GCE-H6** and **GCE-H3** prior to and after exposure to 3 mU/mL of two NAs (Figure 2a, step ii), *3NACP* with a preference for 2,3-Neu5Ac-Gal cleavage and *6NAAU* with 2,6-bond cleavage preference. Nyquist plots of **GCE-H6** and **GCE-H3** show an increase in the *R*_CT_ after incubation with either *6NAAU* or *3NACP*, albeit at different magnitudes (Figure 3a and Figures S5–S7). To better emphasize the differences in the sialoside response to the two NAs, the *R*_CT_ was normalized to the value before the exposure to the enzyme for 120 min, which is based on optimal time dependent-response analyses (Figure S6 and presented in a histogram (Figure 3b). When **GCE-H6** was exposed to the NAs, there was a larger increase in *R*_CT_ for *6NAAU* in comparison to *3NACP*. In the case of **GCE-H3**, there was a preferential response to *3NACP* (Figure 3b). These results are in line with the reported enzyme sialoside specificity.^50,51^ The impedimetric control experiment was carried out by exposing *3NACP* to the GCE modified with propylamine by the same procedure (Figure S8), which resulted in no response, thus indicating that the glycan is required for the recognition event.

EIS analyses were performed on **AuE-H6** and **AuE-H3** prior to and after incubation with 3 mU/mL of the same two NAs (Figure 3c and Figures S9–S11). Nyquist plots of **AuE-H6** and **AuE-H3** show a decrease in the *R*_CT_ after incubation with either *6NAAU* or *3NACP* (Figures S9–S11). Here again, the normalized *R*_CT_ presents the glycan NA-specific preference (Figure 3d). The relative decrease in *R*_CT_ of **AuE-H6** and **AuE-H3** was preferential to *6NAAU* and *3NACP*, respectively. The NA-specific sialoside response preference observed on both surfaces proved that glycan-based EIS analysis can be used to distinguish between different NAs.

To evaluate the working window for these biosensors, **GCE-H3** and **AuE-H3** were exposed to 3, 0.3, and 0.03 mU/mL 3NACP. The normalized responses with different concentrations of the NA were evaluated (Figures S15–S19). These results show that there is a concentration-dependent behavior for enzyme binding on the GCE and the enzymatic reaction on the AuE.

The GCE-based system has higher sensitivity (higher signal-to-noise ratio, SNR) for enzyme binding detection than the AuE system that exhibits an order of magnitude lower impedance signal at low enzyme concentrations. These differences are in line with the fact that the impedimetric signal on the GCE arises from the addition of a high molecular weight entity to the electrode, while on the AuE, a negatively charged moiety is removed from the glycan monolayer. These results suggest that the different biosensing mechanisms dictate the enzyme concentration-sensitivity window.

### Surface–Enzyme Interactions

We performed extended surface studies to elucidate the different EIS responses of gold and glassy carbon surfaces. The incubation of the same NAs with GCE anchored sialosides leads to an increase in *R*_CT_, while the incubation with the AuE anchored with the same two glycans results in a decrease. Surface characterization techniques were used to rationalize the above differences. XPS, CPD, and CA analyses were performed on **GCP-H3** before and after incubation with *3NACP*.

XPS of GCP after electrografting of the glycans (Figure 2a, step i) showed a relative increase in amine and amide characteristic N1s peaks, at 400.4 and 402.1 eV binding energy (BE). The increase was also observed in the peak at 288.9 eV of C1s, which is related to the amide carbonyl (Figure S12). The observed peaks correlate with the molecular features of the linker and the glycan. An atomic concentration of 1.8% was observed (Section S3.10), which is an increase of 1.3% in an atomic nitrogen concentration compared to bare GCP (Figures S12 and S13). The XPS-derived coverage calculations are similar to the ones measured previously also for electrografted sulfated glycans on GCP.^44^ The resulting coverage is 10-fold lower than on the gold surfaces.

XPS of glycated-GCP after incubation with *3NACP* (Figure 2a, step ii) showed an increase in N1s peaks to indicate a nitrogen atomic concentration of 4.4%. The addition of these amide-derived BE peaks proves that proteins were adhered to the glycated-GCP. However, this indicated a lower protein concentration on the surface in comparison with the one reported for nonspecific adhesion of proteins to GCP, which reaches approximately 12%.^52−54^ Additionally, there is an appearance of thiols and disulfide S2p peaks related to the NA (Figure S12). The above results show that the binding is selective to the type of anchored sialoside and prove that the NA does not adsorb non-specifically but rather requires a sialoside recognition motif on the surface to bind. The decrease in *V*_CPD_ to −304 mV after exposure to *3NACP* (ΔCPD = −198 mV), which is very close to the initial value of the clean electrode, −315 mV, implies that there is an addition of an enzyme dipole that cancels the initial glycan–monolayer dipole. An increase in CA from 55 to 82° was observed after adding *3NACP* to **GCP-H3** (Figures S34–S36). Both methods suggest a change in the interfacial nature of the glycated glassy carbon surface, which supports the observed changes in *R*_CT_.

The interface plays a major role in the mode of NA-glycan monolayer interactions on the GCE. We previously showed that sialylation-related enzymatic processes are governed by the characteristic of the sub-monolayer and not only by the glycan.^22^ A systematic study showed that the charge of the sub-monolayer can dictate either adhesion or enable biocatalysis. The response on the modified GCE layer differs from our previous report.^29^ Although both studies were performed on the sialylated-GCE, the two systems are completely different in the monolayer density, charge, dipole, spacer length, anchoring chemistry, and sialoside type. The differences in GCE surface characteristics lead either to adhesion or to biocatalysis of NA.

XPS, CPD, VASE, and CA analyses were performed on **Au-H3** before and after incubation with *3NACP*. XPS analysis of **Au-H3** after incubation with *3NACP* showed no addition of amide bonds related to the NA at a BE of 400.4 eV related to N1s (Figure S14). This suggests that, unlike the glassy carbon surfaces, the enzyme is not adsorbed to the glycated gold surface. In addition, the calculation of the N1s/S2p ratio before and after NA activity suggests that about 52% of sialic acid was enzymatically hydrolyzed from the sialosides. To further explore this, VASE analyses showed no change to the **Au-H3** monolayer thickness after incubation with *3NACP*, which remains 13 Å thick. Furthermore, wettability studies show a small increase in contact angle from 60 to 66°, suggesting the formation of a more slightly, more hydrophobic surface that results from the removal of SA. Surface potential studies show an increase in *V*_CPD_ from −232 to −215 mV, which can be correlated with the removal of the negatively charged SA. The different surface characterizations provided two crucial observations: (a) no enzyme was absorbed into the **Au-H3**, and (b) SA was enzymatically removed from the monolayer. This collective evidence implies that the incubation of the NAs on GCE-sialosides leads to glycan-specific binding, while for gold, it results in biocatalysis. This explains why incubation of NA with glassy carbon-sialoside leads to an increase in *R*_CT_ related to enzyme adhesion (Figure 3b), while the decrease in *R*_CT_ observed on the AuE is related to the selective removal of the negatively charged SA from the sialosides by the enzymes (Figure 3d).

**Scheme 2:**
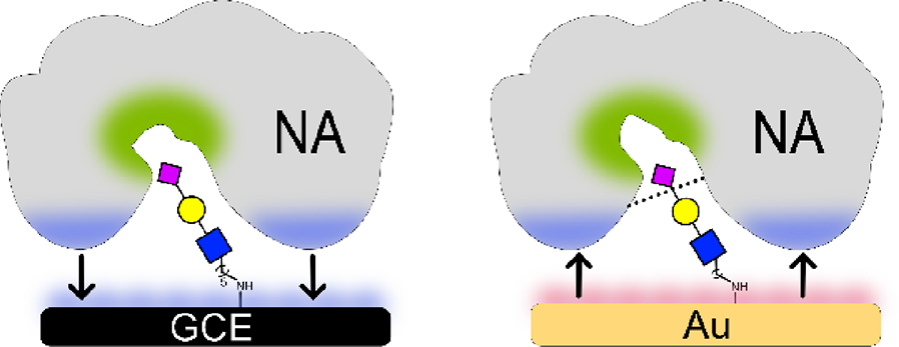
Illustrating the NA Mode of Interactions with the Two Glycated Surfaces Both surfaces permit the recognition of the sialoside with the NA arginine-rich catalytic site (green). The NA hydrophobic interface (blue) is repelled from the negatively charged (red) gold surface and attracts the hydrophobic glassy carbon. The arrow’s direction represents the forces exerted on NA.

We suggest that here, see Scheme 2, the negatively charged sub-monolayers of the lipoic acid-coated gold surfaces prevent enzyme adsorption and promote the enzymatic hydrolysis of sialic acid. Contrary to gold surfaces, the GC interface enables sialoside-selective adhesion of the enzyme to the monolayer and the hydrophobic electrode promotes adhesion. *NA3CP* structure analysis (PDB id 5TSP) indicates the hydrophobic interface surrounding the catalytic site.

### Surface–Sialoside–Neuraminidase Interactions

There are several dominant structural modifications of SA, which determine their binding affinities to sialoside binding proteins.^55,56^ The Neu5Gc SA differs from Neu5Ac by the additional hydroxyl group on the acetylated moiety on C-5. This small molecular modification differentiates between primates (Neu5Gc) to human (Neu5Ac) sialosides and might play a role in the NA-related recognition events.

To explicate the enzyme binding and reaction preferences on sialosides that derive from a different organism, sialosides decorated with Neu5Gc (**M3** and **M6**; Figure 1) were used to functionalize the GCE and AuE. The resulting **GCE-M6**, **GCE-M3**, **AuE-M6**, and **AuE-M3** were incubated with *6NAAU* and *3NACP*, and impedimetric analyses were performed (Figures S20–S29).

**Figure 4:**
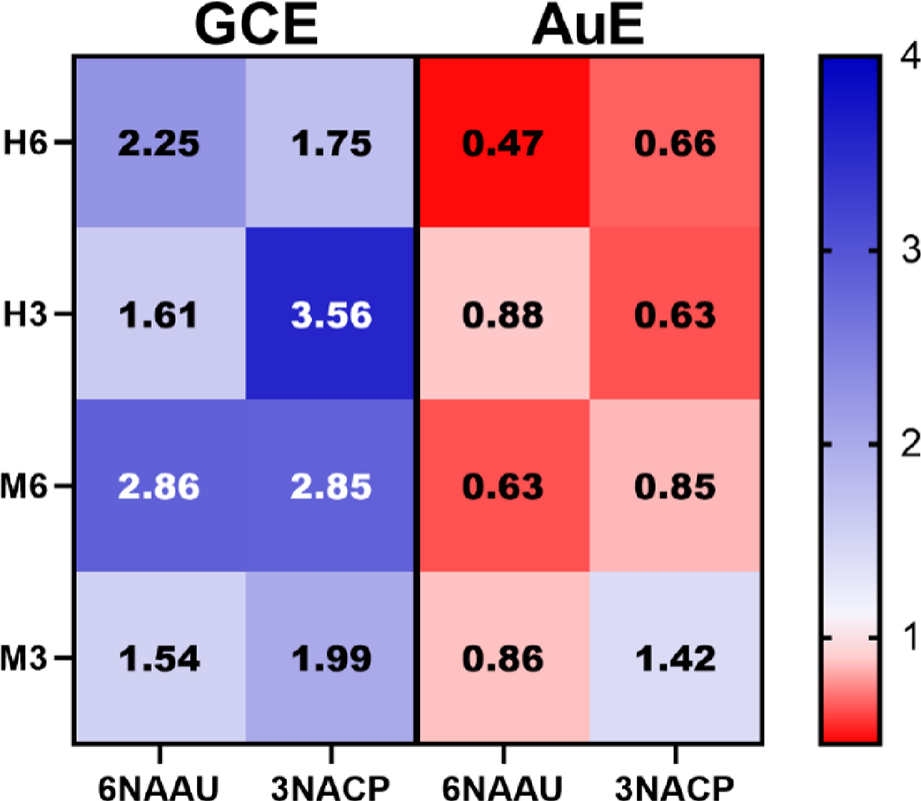
Heat map of the response magnitude after exposing the substrates to the enzyme in different platforms. The response is in normalized *R*_CT_. For the AuE, the decrease in NR_CT_ correlates with larger enzymatic response as manifested in the red color intensity. For the GCE, the increase in NR_CT_ correlates with larger enzyme binding as manifested in the blue color intensity.

To enable a comparison between the two surfaces, four sialosides, and the two enzymes, a heat map that represents the relative change in *R*_CT_ was generated (Figure 4). **GCE-M3** showed a slight preference for *3NACP*, while **GCE-M6** did not show a preferential response to any of the NAs. The binding preferential response is related not only to the position of the SA but also to its type/origin. Therefore, this can imply that the binding and reaction preferences using enzymes can be changed by the type of SA.

The enzymatic response using **AuE-M6** showed a clear preference for the *6NAAU* over *3NACP* compared to the lack of preference observed in the case of **GCE-M6** with lower response intensity than **AuE-H6** (Figure 4). **AuE-M3** showed a low response to the disfavored enzyme *6NAAU*, which is in line with enzymatic preference. However, **AuE-M3** showed an increase in impedance when exposed to *3NACP*, which is an opposite signal trend to other AuE-based systems.

XPS analyses showed that there is a slight increase in the amide signals on **AuE-M3** after incubation with *3NACP*, indicating the adsorption of an enzyme (Figure S30). This may be attributed to the specific binding of *3NACP* to **M3**, which again has Neu5Gc, which might change the type of interaction between the glycated surface and the enzyme. This explains the observed increase in impedimetric response in the case of **AuE-M3** response to *3NACP*.

Previous studies in solution showed that the preferential regioselectivity of *3NACP* and *6NAAU* is pronounced, in terms of kinetic parameters, for human sialosides and also depends on the type of terminal SA.^51^ We compared the response of surface-bound Neu5Ac sialosides to the Neu5Gc ones. When comparing **GCE-M6** with **GCE-H6**, there is a stronger response for binding to **M6** for both enzymes. However, when comparing **GCE-M3** to **GCE-H3**, there is a higher preferential response to *3NACP*. This implies that electrochemical screening against the set of sialosides can provide a tool to differentiate between the NAs. The variability in activity on the AuE and binding on the GCE modified with different types of sialosides is not entirely in line with the preferences determined in solution for *6NAAU* and *3NACP*, where there is a high dependency on the substrate type for the sialoside selectivity.^50,51^

Our results indicate that the enzyme response toward Neu5Ac and Neu5Gc sialoside originates from multiparametric factors including interface nature, SA type, regiochemistry, and electrode type in the present system. The density, the anchoring method, and the proximity to the surface all influence the binding; hence, surface-bound glycans often differ in the binding affinity and preference from the ones in solution. This was reported in ELISA-type assays and should always be considered.^57−60^ However, surface-bound glycan antigens are essential for providing high throughput screening in the form of glycan arrays. There is a need to improve surface-derived screening strategies. The presented work provides a way to modulate the system and use systematic surface characterization methods to decipher the effects of the interface to give a better understanding of the biological activity.

### Neuraminidase Inhibition Studies

**Figure 5:**
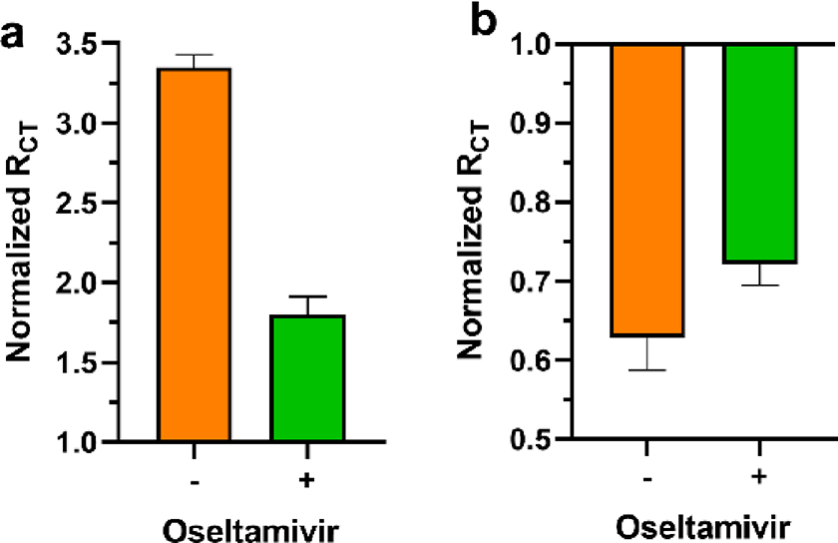
Normalization of impedimetric response of (a) **GCE-H3** and (b) **AuE-H3** to 3 mU/mL *3NACP* with 1 μM oseltamivir (green) and without the inhibitor (orange) for the reactions (Figure 2).

To evaluate the effect of the NA inhibitor on the affinity and enzymatic reaction, **GCE-H3** and **AuE-H3** were chosen because systems containing **H3** had a high response to the enzyme. **GCE-H3** was exposed to 3 mU/mL *3NACP* in the absence and presence of 1 μM of the antiviral drug oseltamivir (Figure 5a and Figure S31), which is a known NA inhibitor.^61^ The addition of the oseltamivir resulted in a lower increase in *R*_CT_ of **GCE-H3** compared to the non-treated system (Figure 3).

The inhibition of **GCE-H3** response as a result of incubation with *3NACP* in the presence of oseltamivir, which is a competitive inhibitor for the catalytic site of neuraminidase, suggests that the inhibition decreases the enzyme binding affinity to the glycated layer (see also Figure S12). Additionally, an evaluation of inhibition was performed on **AuE-H3** with 3 mU/mL *3NACP* in the presence and absence of 1 μM oseltamivir (Figure 5b and Figure S32). The decrease in response using the system of **AuE-H3** in the presence of the inhibitor suggests that the biocatalytic activity of the enzyme was inhibited.

The fact that the same inhibitor inhibits both binding and catalysis confirms that it functions through the catalytic site on both surfaces. This suggests that the interaction of NA with sialosides on the GCE is mediated through specific binding of the catalytic site to the glycans on the surface.

In this work, the modified AuE was sensitive to the enzymatic process while the modified GCE was selectively responsive to the presence of the enzyme binding on the surface. The control experiment with the GCE modified with propyl amine showed that the sialoside in the interface is mandatory for NA binding; hence, the platform requires combination of substrate and interfacial properties for the selective binding. The intensity of the response was correlated with the sialoside-enzyme preference regardless of the surface type. It was observed that the modified GCE has higher sensitivity compared to the modified AuE counterpart. Inhibition analyses showed that both platforms can detect inhibition in binding and reaction; however, the higher sensitivity of binding analyses suggests that the GCE is the preferable method to evaluate NA inhibitors. Both systems are viable for sensing where the AuE can be used for detection of the desialylation enzymatic reaction, while the GCE can be used for a more sensitive detection of sialoside-specific NA affinity.

The ability to determine binding or enzymatic catalytic reactions relies on the surface properties such as interfacial charge, layer flexibility, exposed functionalities of the interface, substrate density, and surface type. The observed catalysis on gold surfaces can be attributed to two factors: the high glycan density and the negatively charged sub-monolayer. While the high density of the glycans ensures significant hydrolysis, the negative charge prevented enzyme adhesion.^22^ The low coverage of sialosides on GC surfaces leads to both specific glycan recognition by the catalytic site of the enzyme and probably an additional interaction of the protein with functional moieties of the bare surface. This explains why the enzymes do not bind the bare electrodes and do not detach from the glycated ones.

## Conclusions

In this work, we show that NA binding, catalysis, and inhibition can be evaluated electrochemically using glycated surfaces. A detailed analytical effort proved that the surface type influences the interaction characteristics and allows differentiating between NA-sialoside binding to enzymatic catalysis. These unique properties of the glycated surfaces were used to study the effect of sialoside chemical features on their interaction’s preferences with different NAs. We found that enzyme-sialoside specificity can be detected electrochemically both not only in the binding level but also by the distinct enzymatic activity. The platform enabled us to demonstrate that NAs can distinguish between sialosides with different regiochemistry. We proved that the ability to differentiate between Neu5Ac and Neu5Gc thereby provides organism-dependent analysis. The two interaction cascades can be used to evaluate NA inhibitor efficiency. The developed platforms can be expended for biosensing of NA that originated from viral or bacterial origin and for NA-derived drug developments.

## Methods

Full experimental details of synthesis and surface characterizations are provided in the Supporting Information.

### Preparation of the Modified Glassy Carbon Electrode

GCEs were manually polished on a micro-cloth pad (Buehler) with deagglomerated alumina suspension with a particle size of 0.05 μm (Buehler) and washed with TDW. The sialylated trisaccharides (0.1 mg) were dissolved in 3 mL of 0.1 M KCl. The trisaccharides were electrografted on the GCE by applying cyclic voltammetry (CV) in the range of 0.6–1.2 V (vs Ag/AgCl 3 M KCl reference electrode) at a scan rate of 10 mV/s for five cycles using BioLogic SAS SP-300 potentiostat. The modified electrodes were rinsed with TDW and stabilized in 50 mM acetate buffer, pH 5, for 1 h at 37 °C before exposure to the enzyme.

### Preparation of the Modified Au Electrode

AuEs were manually polished on a micro-cloth pad (Buehler) with deagglomerated alumina suspension with a particle size of 0.05 μm (Buehler) and washed with TDW. Electrodes were deep-casted with 1 mL of 1 mM LPA in EtOH for 1 h. The electrodes were rinsed with ethanol. A solution of 1 mg/mL COMU in acetonitrile (ACN) with 1% triethylamine (TEA) was prepared. The electrodes were incubated in the solution for 30 min at 25 °C and washed two times with ACN. The sialylated trisaccharides (0.2 mg) were dissolved in 0.2 mL of TDW. The electrodes were drop-casted with 30 μL of the solution for 30 min at 25 °C. The modified electrodes were rinsed with TDW and stabilized in 50 mM acetate buffer, pH 5, for 1 h at 37 °C before exposure to the enzyme.

### Electrochemical Measurements

EIS measurements were performed using a three-electrode standard electrochemical cell with a BioLogic SAS SP-300 potentiostat under single sine AC excitation at a potential of 0.21 V with 10 mV and an amplitude in the frequency range of 100 kHz to 0.1 Hz. The system contains (a) Ag/AgCl (in 3 M KCl) as the reference electrode, (b) Pt rod as the counter electrode, and (c) 3 mm GCE as the working electrode. The measurements were performed in a solution of 5 mM [Fe(CN)6]^3–^/[Fe(CN)6]^4–^, 100 mM KCl, and 50 mM acetate buffer at pH 5. The results were fitted to the equivalent circuit of R_S_[(*R*_CT_*W*)∥*Q*], where RS is the resistance of the solution, *R*_CT_ is the charge-transfer resistance of the layer, *Q* is the constant phase element, and *W* is the Warburg diffusion element. The normalized *R*_CT_ (NR_CT_) was calculated by dividing the *R*_CT_ after exposure (Rf) by the *R*_CT_ prior to exposure (Ri). The value for charge transfer resistance was normalized by the following equation: NR_CT_ = Rf/Ri.

### Exposure to the Enzyme

Stock samples of NA were prepared as described in a previous paper. Stock samples of NA were dissolved in 200 μL of 50 mM acetate buffer (pH 5). Each stock (20 μL) was added to 1780 μL of 50 mM acetate buffer, giving a final volume of 1.8 mL (3 mU/mL). Each modified electrode was drop-casted with 50 μL of the solution for 120 min. After the exposure, the electrodes were rinsed with the acetate buffer. For lower enzyme concentrations, the reaction stock was diluted with 50 mM acetate buffer. Each modified electrode was drop-casted with 50 μL of the solution for different durations from 5 to 120 min. After the reaction, the electrodes were rinsed with the acetate buffer.

### Exposure to the Enzyme in the Presence of Oseltamivir

Stock samples of NA were dissolved in 200 μL of 50 mM acetate buffer (pH 5). Each stock (2 μL) was added to 1780 μL of 1 μM or 0.1 μM oseltamivir in 50 mM acetate buffer solution, giving a solution final volume of 1.8 mL (0.3 mU/mL). Each modified electrode was drop-casted with 50 μL of the solution for 120 min. After the reaction, the electrodes were rinsed with the acetate buffer.

## Supporting Information Available

Synthetic procedures of the sialosides with their corresponding characterizations, experiment for electrochemistry and surface characterizations, and EIS and XPS data (PDF)
