# Supplementary material for: Biocatalysis versus Molecular Recognition in Sialoside-Selective Neuraminidase Biosensing
Source: ACS Chem Biol. 2023 Feb 15;18(3):605–14. doi: 10.1021/acschembio.2c00913 (PMC10028605; doi:10.1021/acschembio.2c00913)
Supplement: Supplementary file 1 — cb2c00913_si_001.pdf [file cb2c00913_si_001.pdf]

Supporting Information

Biocatalysis versus molecular recognition in sialoside-selective neuraminidase biosensing

Israel Alshanski,[a] Suraj Toraskar,[b] Ariel Shitrit,[a] Daniel Gordon-Levitan,[a] Prashant Jain,[b] Raghavendra Kikkeri,[b]* Mattan Hurevich,[a]* Shlomo Yitzchaik[a]*

[a] The institute of chemistry and Center of Nanotechnology, The Hebrew University of Jerusalem. Jerusalem, 91904, Israel

[b] Indian Institute of Science Education and Research, Dr. Homi Bhabha Road, Pune-411008, India.

*Corresponding authors: rkikkeri@iiserpune.ac.in (R.K.), mattan.hurevich@mail.huji.ac.il (M.H.), and shlomo.yitzchaik@mail.huji.ac.il (S.Y.)

Table of Contents

1. Materials S4

2. Methods S5

2.1 Synthesis of the saccharides S5

2.1.1 Synthesis of *N*-Glycolylneuraminic acid donor S5

2.1.2 Synthesis of galactose acceptor 12 .... S12

2.1.3 Synthesis of galactose acceptor 7 .... S15

2.1.4 Synthesis of Gc-2,6 trisaccharide M6. .... S20

2.1.5 Synthesis of Gc-2,3 trisaccharide M3 .... S27

2.1.6 Synthesis of Ac-2,6 trisaccharide H6. .... S32

2.1.7 Synthesis of Ac-2,3 trisaccharide H3. .... S38

2.2 Surface modifications and characterizations S42

3. Figures S44

3.1 Electrografting with the sialosides S44

3.2 Modification of Au Electrode S45

3.3 3D structures of the molecules for surface characterizations S46

3.4 Nyquist plot of impedimetric response for **GCE-H3** and **GCE-H6** prior and after exposure to *3NACP* and *6NAAU* S47

3.5 Response of Control GCE-Propyl amine to *3NACP* without glycan S48

3.6 Nyquist plot of impedimetric response for **AuE-H3** and **AuE-H6** prior and after exposure to *3NACP* and *6NAAU* S49

3.7 XPS analyses of **GCP-H3** before and after exposure to *3NACP* S51

3.8 XPS analyses of **Au-H3** before and after exposure to *3NACP* S53

3.9 Enzyme concentration dependent response of **GCE-H3** to *3NACP* S54

3.10 Enzyme concentration dependent response of **AuE-H3** to *3NACP* S55

3.11 Enzyme concentration dependent response summary S56

3.12 Nyquist plot of impedimetric response for **GCE-M3** and **GCE-M6** prior and after exposure to *3NACP* and *6NAAU* S57

3.13 Summary of the response of exposure of **GCE-M3** and **GCE-M6** to *3NACP* and *6NAAU* S59

3.14 Nyquist plot of impedimetric response for **AuE-M3** and **AuE-M6** prior and after exposure to *3NACP* and *6NAAU* S60

3.15 Summary of the response of exposure of **AuE-M3** and **AuE-M6** to *3NACP* and *6NAAU* S62

3.16 XPS analyses of **Au-M3** before and after exposure to *3NACP* S63

3.17 Nyquist plot of **GCE-H3** response to *3NACP* in presence of Oseltamivir S64

3.18 Nyquist plot of **AuE-H3** response to *3NACP* in presence of Oseltamivir S64

3.19 XPS analyses of Au-LPA before and after coupling with **H3** S65

3.20 XPS data for GCE S65
3.21 Contact Angle Measurements S66
4. NMR and MS analyses of the compounds S67
5. References S127

1. Materials

All reagents and solvents obtained from suppliers were used without further purification. All reactions were carried out under a nitrogen atmosphere in anhydrous solvents unless otherwise noted. Reactions were monitored by TLC on Merck silica gel 60 F_{254}. The compounds were visualized under UV light or dipping the TLC plate in CAM solution followed by heating. Column chromatography was carried out using the force flow of the indicated solvents on Fluka kieselgel 60 (230-400 mesh). ^{1}H and ^{13}C NMR spectra of the compounds were recorded on Bruker 400 MHz, Bruker 600 MHz and Jeol 400 MHz with cryoprobe using residual solvents as an internal reference ($CDCl_3$ δH 7.26 ppm, δC 77.3 ppm, CD_3OD δH 3.31 ppm, δC 49.0 ppm, and D_2O δH 4.79 ppm). The chemical shifts (δ) are reported in ppm and coupling constants (J) in Hz.

2. Methods

2.1 Synthesis of the saccharides

2.1.1 Synthesis of *N*-Glycolylneuramininc acid donor

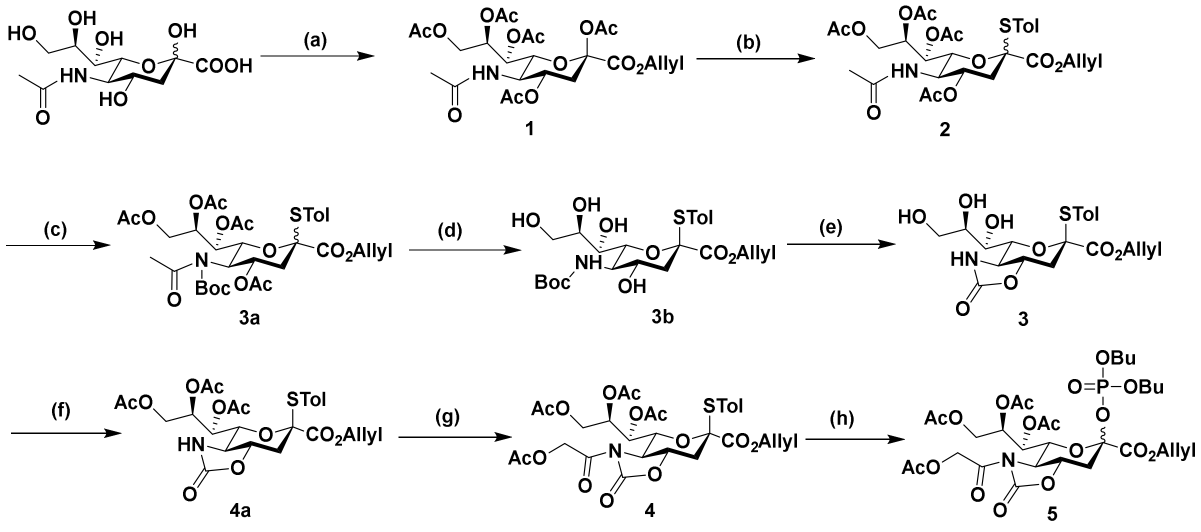

Scheme S1. Synthesis of donor 8. Reagents and conditions: (a) i) Ac_2O, pyridine, rt, 12 h; ii) Cs_2CO_3, AllylBr, DMF, 40 °C, 4 h, 69% over two steps; (b) *p*-Thiocresol, $BF_3{\cdot}OEt_2$, CH_2Cl_2, rt, 24 h, 78%; (c) Boc_2O, DMAP, THF, 60 °C, 4 h, 85%; (d) NaOMe, AllylOH, rt, 4 h, 56 %; (e) i)TFA/CH_2Cl_2 (1:1, v/v), rt, 3 h ii) $NO_2C_6H_4OCOCl$, $NaHCO_3$, H_2O/MeCN (2:1, v/v), 0 °C, 4 h, 47% over two steps; (f) Ac_2O, pyridine, rt, 12 h, 84%; (g) Acetoxyacetyl chloride, DIPEA, CH_2Cl_2, 0 °C to rt, 2 h, 83 %; (h) NIS, TfOH, dibutyl phosphate, CH_2Cl_2, 0 °C, 6 h, 74%.

Synthesis of compound 1

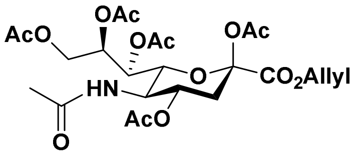

To a solution of *N*-Acetylneuraminic acid (1.5 gm, 4.85 mmol) in pyridine (10 mL) was added acetic anhydride (3.67 mL, 38.8 mmol) at 0 °C and stirred at RT for 12 hours. The reaction mixture was quenched with methanol and concentrated *in vacuo,* and the residue was co-evaporated with toluene and used as such for next step without further

purification. Next, the residue in DMF (20 mL), along with cesium carbonate (4.7 gm, 14.55 mmol), allyl bromide (1.2 mL, 14.55 mmol) was stirred at 40 °C for 4 hours and quenched with methanol. The reaction mixture was filtered through celite bed, and the filtrate was concentrated. The residue was purified by silica gel column chromatography using a mixture of (4:1, v/v) dichloromethane and methanol as eluent to afford compound 1 (1.9 gm, 69% over two steps). ^{1}H NMR (400 MHz, Chloroform-*d*) δ 5.98-5.82 (m, 1H), 5.45-5.31 (m, 3H), 5.30-5.20 (m, 2H), 5.07 (ddd, *J* = 6.7, 5.0, 2.6 Hz, 1H), 4.74-4.58 (m, 2H), 4.45 (dd, *J* = 12.4, 2.6 Hz, 1H), 4.19-4.05 (m, 3H), 2.55 (dd, *J* = 13.5, 5.0 Hz, 1H), 2.14 (s, 3H), 2.14 (s, 3H), 2.10 (dt, *J* = 4.5, 2.4 Hz, 1H), 2.05 (s, 3H), 2.03 (s, 3H), 2.03 (s, 3H), 1.89 (s, 3H). ^{13}C NMR (101 MHz, Chloroform-*d*) δ 171.13, 170.71, 170.4, 170.40, 170.38, 168.37, 165.57, 131.28, 119.14, 97.74, 73.04, 71.49, 68.44, 68.02, 66.96, 62.22, 49.50, 36.02, 23.32, 21.05, 20.98, 20.92, 20.89. HRMS (ESI): Calcd for $C_{24}H_{33}NNaO_{14}$ $[M+Na]^+$ 582.1799, found 582.1798.

Synthesis of compound 2

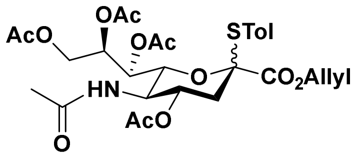

To a stirred solution of compound 1 (1.2 gm, 2.14 mmol) and 4-methylbenzenethiol (0.4 gm, 3.21 mmol) in anhydrous dichloromethane (15 mL) was added boron trifluoride diethyl etherate (0.8 mL, 6.42 mmol) at 0 °C. The reaction mixture was stirred at room temperature for 24 hours, neutralized with triethylamine and washed with brine solutions. The organic layer was dried over sodium sulphate, concentrated *in vacuo* and purified by silica gel column chromatography using a mixture of (9:1, v/v) dichloromethane and methanol as eluent to afford compound 2 (1.05 gm, 78%, α/β=1:13). β anomer: ^{1}H NMR (400 MHz, Chloroform-*d*) δ 7.33 (d, *J* = 8.1 Hz, 2H), 7.12 (d, *J* = 8.0 Hz, 2H), 5.83-5.70 (m,1H), 5.53 (s, 1H), 5.45 (t, *J* = 2.5 Hz, 1H), 5.39 (ddd, *J* = 11.6, 10.4, 4.8 Hz, 1H), 5.30-5.17 (m, 2H), 4.95 (dt, *J* = 8.4, 2.4 Hz, 1H), 4.61 (dd, *J* = 10.5, 2.6 Hz, 1H), 4.50 (tddd, *J* = 12.9, 11.6, 5.9, 1.2 Hz, 2H), 4.39 (dd, *J* = 12.3, 2.3 Hz, 1H), 4.19-4.08 (m, 2H), 2.64 (dd, *J* = 13.9, 4.9 Hz, 1H), 2.32 (s, 3H), 2.10 (s, 3H), 2.06 (s, 3H), 2.03 (s, 3H), 1.97 (s, 3H),

1.89 (s, 3H). ^{13}C NMR (101 MHz, Chloroform-*d*) δ 171.21, 171.06, 170.40, 170.36, 167.51, 140.23, 136.29, 131.42, 130.05, 125.36, 119.25, 88.79, 73.26, 73.10, 69.22, 68.98, 66.64, 62.59, 49.62, 37.49, 23.31, 21.42, 21.21, 21.01, 20.88. HRMS (ESI): Calcd. for $C_{29}H_{38}NO_{12}S$ $[M+H]^+$ 624.2115, found 624.2114.

Synthesis of compound 3a

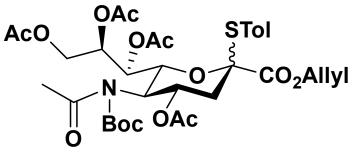

To a stirred solution of compound **2** (1.8 gm, 2.89 mmol) in anhydrous THF (25 mL) was added di-tert-butyl dicarbonate (3.3 mL, 14.45 mmol), DMAP (70 mg, 0.57 mmol) and stirred at 60 °C for 4 hours. The reaction mixture was cooled, quenched with methanol and concentrated *in vacuo*. The residue was purified by silica gel column chromatography using a mixture of (3:2, v/v) ethyl acetate and hexane as eluent to afford compound **3a** (1.78 gm, 85%). β anomer: ^{1}H NMR (400 MHz, Chloroform-*d*) δ 7.34 (d, *J* = 8.1 Hz, 2H), 7.12 (d, *J* = 8.0 Hz, 2H), 5.84-5.69 (m, 2H), 5.43 (dd, *J* = 10.1, 2.1 Hz, 1H), 5.34 (dd, *J* = 3.1, 2.0 Hz, 1H), 5.28-5.16 (m, 2H), 5.11 (dt, *J* = 8.0, 2.6 Hz, 1H), 4.85 (t, *J* = 10.5 Hz, 1H), 4.53-4.43 (m, 2H), 4.39 (dd, *J* = 12.4, 2.2 Hz, 1H), 4.16 (dd, *J* = 12.4, 8.0 Hz, 1H), 2.72 (dd, *J* = 13.8, 4.9 Hz, 1H), 2.35 (s, 3H), 2.32 (s, 3H), 2.05 (s, 3H), 2.04 (s, 3H), 1.97 (s, 3H), 1.95 (s, 3H), 1.70 (s, 9H). ^{13}C NMR (101 MHz, Chloroform-*d*) δ 173.94, 170.60, 170.51, 170.32, 169.96, 167.38, 152.11, 140.17, 136.54, 136.36, 131.45, 130.00, 125.77, 119.07, 89.33, 85.42, 72.79, 72.34, 68.66, 66.56, 66.49, 62.33, 52.98, 38.87, 28.28, 26.74, 21.42, 21.13, 20.95, 20.85, 20.81. HRMS (ESI): Calcd. for $C_{34}H_{45}NNaO_{14}S$ $[M+Na]^+$ 746.2458, found 746.2458.

Synthesis of compound 3b

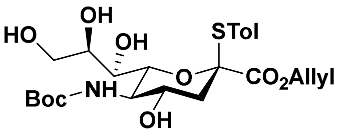

Compound **3a** (2.6 gm, 3.59 mmol) in allyl alcohol, was basified with 1M NaOMe until the pH of the reaction mixture reached 9. The reaction mixture was stirred at room temperature for 4 hours, quenched with acetic acid, and concentrated *in vacuo*. The residue was purified by silica gel column chromatography using a mixture of (4:1, v/v) dichloromethane and methanol as eluent to afford compound **3b** (1.05 gm, 56%). β anomer: ^{1}H NMR (400 MHz, Methanol-d_4) δ 7.47 (d, J = 8.1 Hz, 2H), 7.16 (d, J = 7.9 Hz, 2H), 5.81-5.74 (m, 1H), 5.28 (dq, J = 17.3, 1.6 Hz, 1H), 5.20 (dq, J = 10.3, 1.3 Hz, 1H), 4.48 (d, J = 10.6 Hz, 1H), 4.40-4.42 (m, 2H), 4.06 (ddd, J = 11.6, 9.9, 4.6 Hz, 1H), 3.87-3.78 (m, 2H), 3.71-3.66 (m, 2H), 3.60 (t, J = 10.2 Hz, 1H), 2.67 (dd, J = 13.6, 4.6 Hz, 1H), 2.34 (s, 3H), 1.96 (dd, J = 13.6, 11.6 Hz, 1H), 1.48 (s, 9H). ^{13}C NMR (101 MHz, Methanol-d_4) δ 170.11, 159.10, 140.93, 137.33, 132.81, 130.63, 127.91, 118.94, 91.22, 80.51, 73.84, 71.51, 70.74, 68.29, 67.30, 65.23, 55.06, 42.31, 28.78, 21.27. HRMS (ESI): Calcd. for $C_{24}H_{36}NO_9S$ $[M+H]^+$ 514.2111, found 514.2107.

Synthesis of compound 3

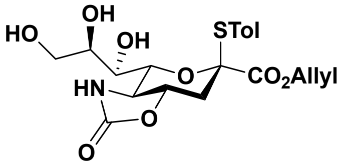

Compound **3b** (3.5 gm, 6.81 mmol) in a 1:1 (v/v) mixture of CH_2Cl_2/TFA (30mL) was stirred at room temperature for 3 hours. The reaction mixture was concentrated *in vacuo,* and the residue was co-evaporated with toluene and dried under a high vacuum for 12 hours. The residue in a 1:2 (v/v) mixture of ACN/H_2O (60 mL) was treated with sodium bicarbonate (2.86 gm, 34.05 mmol). The reaction mixture was cooled to 0 °C, and 4-nitrophynylchloroformate (4.1 gm, 20.43 mmol) was added. Next, the resulting mixture was stirred at 0 °C for 4 hours and extracted with ethyl acetate (50 mL x 3). The organic

layer was dried over sodium sulphate and concentrated *in vacuo*. The residue was purified by silica gel column chromatography using a mixture of (4:1, v/v) ethyl acetate and methanol as eluent to afford compound **3** (1.4 gm, 47% over two steps). ^{1}H NMR (400 MHz, Methanol-d_4) δ 7.46 (d, J = 8.1 Hz, 2H), 7.15 (d, J = 8.0 Hz, 2H), 5.83-5.70 (m, 1H), 5.32-5.15 (m, 2H), 4.71 (dd, J = 10.5, 0.6 Hz, 1H), 4.42 (dt, J = 5.8, 1.2 Hz, 2H), 4.20 (ddd, J = 11.6, 10.3, 4.7 Hz, 1H), 4.11-4.01 (m, 1H), 3.81 (dd, J = 11.2, 2.8 Hz, 1H), 3.78-3.72 (m, 1H), 3.65 (dd, J = 11.2, 5.3 Hz, 1H), 3.49 (d, J = 8.9 Hz, 1H), 2.68 (dd, J = 13.6, 4.7 Hz, 1H), 2.32 (s, 3H), 1.98 (dd, J = 13.6, 11.6 Hz, 1H). ^{13}C NMR (101 MHz, Methanol-d_4) δ 170.39, 141.32, 137.53, 130.91, 127.84, 119.35, 91.37, 72.72, 71.58, 70.86, 67.81, 67.71, 65.19, 54.61, 42.39, 21.50. HRMS (ESI): Calcd for $C_{20}H_{26}NO_8S$ $[M+H]^+$ 440.1339, found 440.1335.

Synthesis of compound 4a

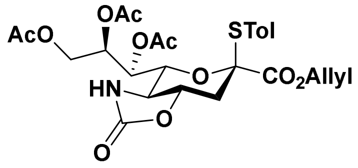

Compound **3** (1.7 gm, 3.87 mmol) in pyridine (20 mL) was added dropwise acetic anhydride (1.7 mL, 19.35 mmol) at 0 °C and stirred at room temperature for 12 hours and concentrated *in vacuo*. The residue was purified by silica gel column chromatography using a mixture of (7:3, v/v) ethyl acetate and hexane as eluent to afford compound **4a** (1.85 gm, 84%). ^{1}H NMR (400 MHz, Chloroform-d) δ 7.27 (d, J = 8.2 Hz, 2H), 7.12 (d, J = 8.0 Hz, 2H), 5.84-5.67 (m, 1H), 5.51 (s, 1H), 5.30-5.23 (m, 1H), 5.23-5.18 (m, 2H), 5.15 (td, J = 5.7, 2.4 Hz, 1H), 4.73-4.63 (m, 1H), 4.57 (dd, J = 9.7, 2.6 Hz, 1H), 4.54-4.42 (m, 2H), 4.35 (dd, J = 12.6, 2.4 Hz, 1H), 4.28 (dd, J = 12.5, 6.0 Hz, 1H), 3.10 (ddd, J = 11.1, 9.7, 1.4 Hz, 1H), 2.79 (dd, J = 13.1, 3.8 Hz, 1H), 2.33 (s, 3H), 2.24 (t, J = 12.8 Hz, 1H), 2.13 (s, 3H), 2.07 (d, J = 1.0 Hz, 3H), 2.01 (d, J = 0.8 Hz, 3H). ^{13}C NMR (101 MHz, Chloroform-d) δ 171.37, 170.37, 170.17, 167.01, 159.26, 140.52, 136.09, 131.20, 130.10, 125.31, 119.14, 88.87, 76.96, 73.22, 70.84, 70.29, 66.60, 61.77, 58.55, 36.42, 21.38, 21.04, 20.80, 20.76. HRMS (ESI): Calcd. for $C_{26}H_{32}NO_{11}S$ $[M+H]^+$ 566.1696, found 566.1698.

Synthesis of compound 4

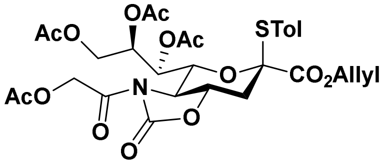

Compound **4a** (1.5 gm, 2.65 mmol) in anhydrous dichloromethane (20 mL) was mixed with acetoxyacetyl choride (1.4 mL, 13.5 mmol), DIPEA (3.69 mL, 21.2 mmol) at 0 °C. The reaction mixture was brought to room temperature and stirred at room temperature for 2 h. The resulting mixture was diluted with dichloromethane, washed with sodium bicarbonate and brine solutions. The organic layer was dried over sodium sulphate and concentrated *in vacuo.* The residue was purified by silica gel chromatography using a mixture of (1:1, v/v) ethyl acetate and hexane as eluent to afford compound **4** (1.46 gm, 83%). ^{1}H NMR (400 MHz, Chloroform-*d*) δ 7.35 (d, *J* = 8.1 Hz, 2H), 7.13 (d, *J* = 7.9 Hz, 2H), 5.78-5.71 (m, 1H), 5.55 (t, *J* = 2.6 Hz, 1H), 5.34-5.18 (m, 3H), 4.98 (dt, *J* = 7.8, 2.6 Hz, 1H), 4.85 (dd, *J* = 9.1, 2.6 Hz, 2H), 4.76 (ddd, *J* = 12.8, 11.3, 3.7 Hz, 1H), 4.61-4.44 (m, 2H), 4.30 (dd, *J* = 12.1, 2.7 Hz, 1H), 4.02 (dd, *J* = 12.1, 7.9 Hz, 1H), 3.73 (dd, *J* = 11.3, 9.2 Hz, 1H), 2.83 (dd, *J* = 13.0, 3.7 Hz, 1H), 2.50 (s, 3H), 2.33 (s, 3H), 2.29 (t, *J* = 12.9 Hz, 1H), 2.11 (s, 3H), 2.06 (s, 3H), 1.96 (s, 3H).^{13}C NMR (101 MHz, Chloroform-*d*) δ 172.52, 171.13, 170.44, 169.81, 167.08, 153.71, 140.66, 131.23, 130.13, 124.79, 119.41, 88.05, 75.69, 75.22, 73.70, 72.52, 66.76, 62.81, 59.78, 35.81, 24.81, 21.41, 21.20, 20.84, 20.82. HRMS (ESI): Calcd. for $C_{30}H_{35}NNaO_{14}S$ $[M+Na]^+$ 688.1676, found 688.1675.

Synthesis of compound 5

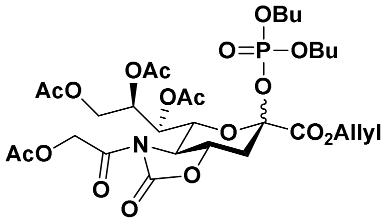

Compound **4** (1.9 gm, 2.85 mole) and dibutyl phosphate (1.7 mL, 8.55 mmol) was dissolved in anhydrous dichloromethane (30 mL) and activated 4 Å molecular sieves was added. The reaction mixture was stirred at room temperature for 2 hours. The resultant mixture was cooled to 0 °C, and NIS (0.96 gm, 4.27 mmol), TfOH (53 µl, 0.57 mmol) were added. The reaction mixture was stirred at 0 °C for

6 hours, neutralized with DIPEA and concentrated *in vacuo.* The residue was purified by silica gel column chromatography using a mixture of (3:2, v/v) ethyl acetate and hexane as eluent to afford compound **5** (1.59 gm, 74%, α/β=1:1).

^{1}H NMR (400 MHz, Chloroform-*d*) δ 5.94 (dddt, *J* = 17.5, 10.3, 7.3, 5.9 Hz, 2H), 5.73 (dd, *J* = 8.1, 1.5 Hz, 1H), 5.64 (dd, *J* = 5.4, 1.9 Hz, 1H), 5.42-5.35 (m, 2H), 5.31-5.24 (m, 4H), 5.15-5.00 (m, 4H), 4.83 (dd, *J* = 9.7, 1.5 Hz, 1H), 4.76-7.64 (m, 6H), 4.42 (dd, *J* = 12.2, 2.8 Hz, 1H), 4.35 (dd, *J* = 12.3, 2.7 Hz, 1H), 4.26 (ddd, *J* = 13.3, 11.2, 4.0 Hz, 1H), 4.17-4.01 (m, 12H), 3.83 (ddd, *J* = 15.2, 11.2, 9.6 Hz, 2H), 2.99 (dd, *J* = 12.3, 4.0 Hz, 1H), 2.90 (dd, *J* = 12.8, 3.7 Hz, 1H), 2.72 (t, *J* = 12.7 Hz, 1H), 2.37 (td, *J* = 12.8, 2.2 Hz, 1H), 2.16 (s, 3H), 2.15 (s, 3H), 2.12 (s, 3H), 2.09 (s, 3H), 2.08 (s, 3H), 2.08 (s, 3H), 2.03 (s, 3H), 2.02 (s, 3H), 1.6-1.61 (m, 8H), 1.38 (m, 8H), 0.95-0.91 (m, 12H). ^{13}C NMR (101 MHz, Chloroform-*d*) δ 172.25, 171.97, 170.75, 170.70, 170.65, 170.09, 170.05 169.90, 166.62, 166.55, 164.84, 153.64, 153.59, 131.09, 130.91, 120.11, 119.44, 99.05, 99.00, 98.28, 98.21, 76.74, 74.22, 74.12, 72.60, 71.70, 71.64, 70.03, 68.72, 68.66, 68.57, 68.51, 68.32, 68.26, 68.20, 68.14, 67.51, 67.47, 62.83, 62.62, 59.03, 58.43, 36.15, 36.02, 35.98, 32.29, 32.25, 32.22, 32.18, 32.15, 29.80, 24.77, 24.7, 21.12, 21.09, 20.90, 20.86, 18.73, 18.69, 13.69, 13.66. HRMS (ESI): Calcd. for $C_{31}H_{47}NO_{18}P$ $[M+H]^{+}$ 752.2531, found 752.2528.

2.1.2 Synthesis of galactose acceptor 12

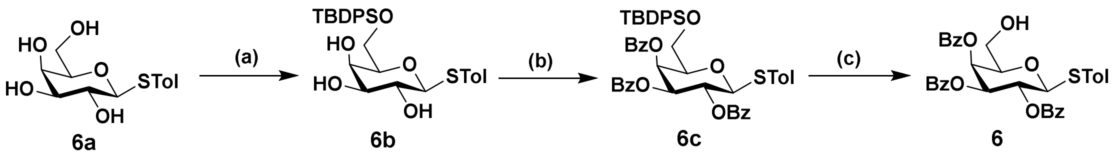

Scheme S2. Synthesis of galactose acceptor **6**. Reagents and conditions: (a) TBDPSCl, Imidazole, DMF, rt, 12 h, 77%; (d) BzCl, pyridine, rt, 12 h, 89%; (e) TBAF, AcOH, THF, rt, 36 h, 73%.

Synthesis of compound 6a

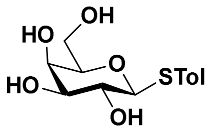

Compound **6a** was synthesised by following known literature protocol.[1] ^{1}H NMR (400 MHz, Methanol-*d*$_4$) δ 7.46 (d, *J* = 8.2 Hz, 2H), 7.13 (d, *J* = 7.9 Hz, 2H), 4.52 (d, *J* = 9.6 Hz, 1H), 3.90 (dd, *J* = 3.3, 1.1 Hz, 1H), 3.77-3.72 (m, 2H), 3.61-3.53 (m, 2H), 3.50 (dd, *J* = 9.2, 3.3 Hz, 1H), 2.32 (s, 3H). ^{13}C NMR (101 MHz, Chloroform-*d*) δ 138.40, 132.88, 132.05, 130.52, 90.64, 80.55, 76.32, 70.99, 70.39, 62.57, 21.07. HRMS (ESI): Calcd. for $C_{13}H_{18}NaO_5S$ $[M+Na]^+$ 309.0773, found 309.0773.

Synthesis of compound 6b

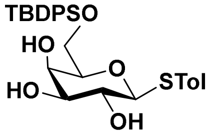

Compound **6a** (1.10 gm, 3.84 mmol) in anhydrous DMF (10 mL) was added to ter-butyldiphenylchlorosilane (1.10 mL, 4.22 mmol) and imidazole (0.4 gm, 5.76 mmol). The

reaction mixture was stirred at room temperature for 12 hours, quenched with methanol and concentrated *in vacuo*. The residue was purified by silica gel column chromatography using a mixture of (3:2, v/v) ethyl acetate and hexane as eluent to afford compound **6b** (1.55 gm, 77%). ^{1}H NMR (400 MHz, Chloroform-*d*) δ 7.73-7.67 (m, 4H), 7.46-7.35 (m, 8H), 7.05 (d, *J* = 7.7 Hz, 2H), 4.45 (d, *J* = 9.6 Hz, 1H), 4.10 (s, 1H), 3.96-3.93 (m, 2H), 3.66 (t, *J* = 9.3 Hz, 1H), 3.60-3.52 (m, 2H), 3.22 (s, 1H), 3.06 (s, 1H), 2.31 (s, 3H), 1.78 (s, 1H), 1.06 (s, 9H). ^{13}C NMR (101 MHz, Chloroform-*d*) δ 135.79, 135.71, 133.01, 130.02, 129.87, 127.96, 88.98, 78.27, 75.08, 70.01, 69.58, 63.88, 26.91, 21.28, 19.2. HRMS (ESI): Calcd. for $C_{29}H_{36}NaO_5SSi$ $[M+Na]^+$ 547.1950, found 547.1958.

Synthesis of compound 6c

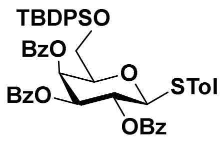

Compound **6b** (1.3 gm, 2.48 mmol) in pyridine (10 mL) was mixed with benzoyl chloride (1.44 mL, 12.4 mmol) at 0 °C. The reaction mixture was stirred at room temperature for 12 h neutralized with methanol and concentrated *in vacuo*. The residue was purified by silica gel column chromatography using a mixture of (2:3, v/v) ethyl acetate and hexane as eluent to afford compound **6c** (1.86 gm, 89%). ^{1}H NMR (400 MHz, Chloroform-*d*) δ 7.95 (dd, *J* = 8.4, 1.1 Hz, 2H), 7.83 (dd, *J* = 8.1, 1.1 Hz, 2H), 7.75 (dd, *J* = 8.2, 1.2 Hz, 2H), 7.66 (dd, *J* = 7.9, 1.5 Hz, 2H), 7.60 (t, *J* = 7.4 Hz, 1H), 7.54-7.46 (m, 3H), 7.43-7.35 (m, 10H), 7.28 (d, *J* = 7.4 Hz, 1H), 7.22 (t, *J* = 7.8 Hz, 2H), 7.11 (t, *J* = 6.8 Hz, 4H), 6.04-6.00 (m, 1H), 5.66-5.54 (m, 2H), 4.89 (d, *J* = 9.2 Hz, 1H), 4.09 (t, *J* = 6.9 Hz, 1H), 3.86 (dd, *J* = 10.2, 6.0 Hz, 1H), 3.76 (dd, *J* = 10.2, 7.7 Hz, 1H), 2.37 (s, 3H), 0.99 (s, 9H). ^{13}C NMR (101 MHz, Chloroform-*d*) δ 165.71, 165.34, 165.20, 135.76, 135.61, 134.60, 133.37, 130.10, 129.96, 129.80, 129.71, 129.55, 128.53, 128.35, 127.95, 127.75, 127.43, 86.07, 77.81, 73.51, 68.26, 68.15, 61.57, 26.80, 21.51, 19.15.

Synthesis of compound 6

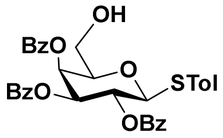

Compound **6c** (2.5 gm, 2.99 mmol) in anhydrous THF (30 mL) was mixed with tertabutylammonioum fluoride (2.35 gm, 7.47 mmol) and acidify with acetic acid until the pH reached 6. The resulting mixture was stirred at room temperature for 36 hours at room temperature. The residue was purified by silica gel column chromatography using a mixture of (1:1, v/v) ethyl acetate and hexane as eluent to afford compound **6** (1.31 gm, 73%). ^{1}H NMR (400 MHz, Chloroform-*d*) δ 7.98 (dd, *J* = 8.5, 1.3 Hz, 2H), 7.90 (dd, *J* = 8.3, 1.3 Hz, 2H), 7.77 (dd, *J* = 8.4, 1.2 Hz, 2H), 7.66-7.55 (m, 1H), 7.53 (t, *J* = 7.4 Hz, 1H), 7.43 (dd, *J* = 14.8, 7.8 Hz, 6H), 7.28-7.17 (m, 3H), 7.14 (d, *J* = 7.9 Hz, 2H), 5.81 (d, *J* = 3.1 Hz, 1H), 5.76 (t, *J* = 9.9 Hz, 1H), 5.56 (dd, *J* = 10.0, 3.2 Hz, 1H), 4.95 (d, *J* = 9.9 Hz, 1H), 4.07 (t, *J* = 6.9 Hz, 1H), 3.84 (dt, *J* = 12.0, 6.8 Hz, 1H), 3.61 (dt, *J* = 12.0, 7.0 Hz, 1H), 2.58 (t, *J* = 7.1 Hz, 1H), 2.37 (s, 3H). ^{13}C NMR (101 MHz, Chloroform-*d*) δ 166.69, 165.63, 165.32, 138.92, 134.62, 133.90, 133.49, 133.46, 130.24, 129.95, 129.89, 129.82, 129.47, 128.85, 128.76, 128.69, 128.59, 128.45, 127.17, 85.98, 77.96, 73.28, 69.13, 68.17, 60.89, 21.49. HRMS (ESI): Calcd. for $C_{34}H_{31}O_8S$ [M+H]$^+$ 599.1740, found 599.1736.

2.1.3 Synthesis of galactose acceptor 7

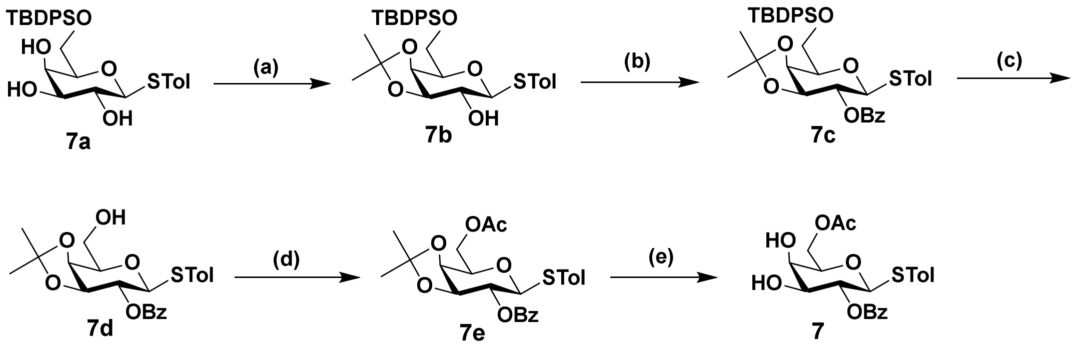

Scheme S3. Synthesis of galactose acceptor **7**. Reagents and conditions: (a) DMP, CSA, acetone, rt, 4 h, 86%; (b) BzCl, pyridine, rt, 12 h, 89%; (c) TBAF, AcOH, THF, rt, 36 h, 74%; (d) Ac_2O, pyridine, rt, 12 h, 88%; (e) AcOH/H_2O (8:2, v/v), 60 °C, 6 h, 82%.

Synthesis of compound 7b

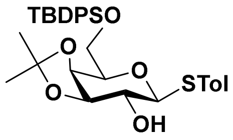

Compound **7a** (1.90 gm, 3.62 mmol) in anhydrous acetone (20 mL) was mixed with 2,2-methoxypropane (0.66 mL, 5.43 mmol) and (1S)-(+)-10-camphorsulfonic acid (40 mg, 0.18 mmol) and stirred at room temperature for 4 hours. The reaction mixture was neutralized with triethylamine and concentrated *in vacuo*. The residue was purified by silica gel column chromatography using a mixture of (2:3, v/v) ethyl acetate and hexane as eluent to afford **7b** (1.76 gm, 86%).^{1}H NMR (400 MHz, Chloroform-*d*) δ 7.77-7.70 (m, 4H), 7.48-7.36 (m, 8H), 7.12-7.04 (m, 2H), 4.41 (d, *J* = 10.2 Hz, 1H), 4.30 (dd, *J* = 5.4, 2.1 Hz, 1H), 4.10 (dd, *J* = 6.9, 5.4 Hz, 1H), 3.98 (m, 2H), 3.90 (ddd, *J* = 6.8, 6.0, 2.1 Hz, 1H), 3.56 (ddd, *J* = 10.2, 7.0, 2.3 Hz, 1H), 2.59 (d, *J* = 2.4 Hz, 1H), 2.33 (s, 3H), 1.43 (s, 3H), 1.35 (s, 3H), 1.08 (s, 9H). ^{13}C NMR (101 MHz, Chloroform-*d*) δ 138.26, 135.75, 135.73, 133.44, 133.02, 129.86, 129.82, 129.80, 128.56, 127.82, 127.75, 110.15, 88.67, 79.11, 77.26, 73.43, 71.68, 63.02, 28.23, 26.88, 26.43, 21.25, 19.33. HRMS (ESI): Calcd. for $C_{32}H_{40}NaO_5SSi$ $[M+Na]^+$ 587.2263, found 587.2291.

Synthesis of compound 7c

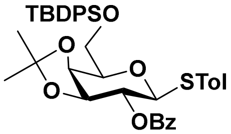

Compound **7b** (1.5 gm, 2.36 mmol) in pyridine (10 mL) was mixed with benzoyl chloride (0.9 mL, 7.9 mmol) at 0 ºC and stirred at room temperature for 12 h. The reaction mixture was quenched with methanol and concentrated *in vacuo*. The residue was purified by silica gel column chromatography using a mixture of (2:3, v/v) ethyl acetate and hexane as eluent to afford compound **7c** (1.58 gm, 89%). ^{1}H NMR (400 MHz, Chloroform-*d*) δ 8.09 (dd, *J* = 8.3, 1.2 Hz, 2H), 7.73 (dd, *J* = 7.9, 1.5 Hz, 4H), 7.58 (t, *J* = 7.5 Hz, 1H), 7.48-7.33 (m, 10H), 7.03 (d, *J* = 8.0 Hz, 2H), 5.33-5.23 (m, 1H), 4.73 (d, *J* = 10.1 Hz, 1H), 4.38-4.32 (m, 2H), 4.06-3.94 (m, 3H), 2.30 (s, 3H), 1.57 (s, 3H), 1.35 (s, 3H), 1.09 (s, 9H). ^{13}C NMR (101 MHz, Chloroform-*d*) δ 165.53, 137.88, 135.78, 133.41, 133.25, 132.57, 130.04, 129.87, 129.75, 128.45, 127.86, 127.80, 110.62, 86.71, 73.62, 72.38, 63.05, 27.87, 26.91, 26.49, 21.26, 19.36. HRMS (ESI): Calcd. for $C_{39}H_{44}NaO_6SSi$ $[M+Na]^+$ 691.2526, found 691.2526.

Synthesis of compound 7d

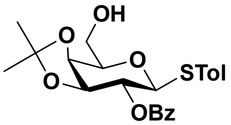

Compound **7c** (2.1 gm, 3.14 mmol) in anhydrous THF (20 mL) was mixed with tetrabutylammonium fluoride (2.4 gm, 7.85 mmol) and acidify with acetic acid until the pH reached 6. The reaction mixture was stirred at room temperature for 36 hours, concentrated *in vacuo* and purified by silica gel column chromatography using a mixture of (1:1, v/v) ethyl acetate and hexane as eluent to afford compound **7d** (1.01 gm, 74%). ^{1}H NMR (400 MHz, Chloroform-*d*) δ 8.07 (dd, *J* = 8.3, 1.2 Hz, 2H), 7.58 (t, *J* = 7.4 Hz, 1H), 7.45 (t, *J* = 7.7 Hz, 2H), 7.34 (d, *J* = 8.1 Hz, 2H), 7.08 (d, *J* = 8.0 Hz, 2H), 5.28 (dd, *J* = 9.9, 7.1 Hz,

1H), 4.74 (d, *J* = 9.9 Hz, 1H), 4.37 (dd, *J* = 7.0, 5.5 Hz, 1H), 4.26 (dd, *J* = 5.4, 2.1 Hz, 1H), 4.03 (ddd, *J* = 10.8, 7.5, 1.9 Hz, 1H), 3.97-3.87 (m, 1H), 3.84 (td, *J* = 12.4, 10.7, 3.9 Hz, 1H), 2.31 (s, 3H), 2.23 (dd, *J* = 9.0, 2.8 Hz, 1H), 1.57 (s, 3H), 1.34 (s, 3H). ^{13}C NMR (101 MHz, Chloroform-*d*) δ 165.48 , 138.21, 133.35, 132.69, 130.04, 129.86, 129.48, 128.49, 111.04, 86.27, 77.05, 74.03, 72.27, 62.67, 27.75, 26.50, 21.25. HRMS (ESI): Calcd. for $C_{23}H_{27}O_6S$ [M+H]$^+$ 431.1528, found 431.1532.

Synthesis of compound 7e

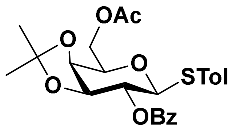

Compound **7d** (1.32 gm, 3.07 mmol) in pyridine (10 mL) was mixed with acetic anhydride dropwise (0.86 mL, 9.21 mmol) at 0 °C and stirred at room temperature for 12 h. Then the reaction mixture was quenched with methanol and concentrated *in vacuo.* The residue was purified by silica gel column chromatography using a mixture of (2:3, v/v) ethyl acetate and hexane as eluent to afford compound **7e** (1.28 gm, 88%). ^{1}H NMR (400 MHz, Chloroform-*d*) δ 8.07 (dd, *J* = 8.4, 1.3 Hz, 2H), 7.58 (t, *J* = 7.4 Hz, 1H), 7.45 (t, *J* = 7.8 Hz, 2H), 7.38 (d, *J* = 8.2 Hz, 2H), 7.07 (d, *J* = 8.4 Hz, 2H), 5.27 (dd, *J* = 9.9, 7.0 Hz, 1H), 4.70 (d, *J* = 9.9 Hz, 1H), 4.40 (d, *J* = 6.2 Hz, 2H), 4.36 (dd, *J* = 7.0, 5.5 Hz, 1H), 4.25 (dd, *J* = 5.4, 2.2 Hz, 1H), 4.04 (td, *J* = 6.0, 2.2 Hz, 1H), 2.31 (s, 3H), 2.10 (s, 3H), 1.57 (s, 3H), 1.34 (s, 3H). ^{13}C NMR (101 MHz, Chloroform-*d*) δ 170.91, 165.46, 138.13, 133.34, 132.83, 130.03, 129.85, 129.75, 129.66, 128.48, 111.08, 86.29, 77.31, 74.38, 73.70, 72.12, 63.80, 27.70, 26.44, 21.24, 20.98. HRMS (ESI): Calcd. for $C_{25}H_{29}O_7S$ [M+H]$^+$ 473.1634, found 473.1632.

Synthesis of compound 7

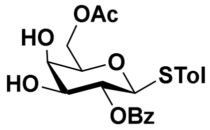

Compound **7e** (1.8 gm, 3.81 mmol) was dissolved in an 8:2 (v/v) mixture of AcOH/H_2O (30 mL) and stirred at 60 °C for 6 h. The reaction mixture was concentrated *in vacuo.* The residue was purified by silica gel column chromatography using a mixture of (1:1, v/v) ethyl acetate and hexane as eluent to afford compound **7** (1.35 gm, 82%). ^{1}H NMR (400 MHz, Chloroform-*d*) δ 8.08 (dd, *J* = 8.4, 1.4 Hz, 2H), 7.60 (t, *J* = 7.4 Hz, 1H), 7.47 (t, *J* = 7.7 Hz, 2H), 7.37 (d, *J* = 8.2 Hz, 2H), 7.08 (d, *J* = 7.8 Hz, 2H), 5.13 (dd, *J* = 10.0, 9.2 Hz, 1H), 4.73 (d, *J* = 10.0 Hz, 1H), 4.45-4.31 (m, 2H), 3.98 (t, *J* = 3.5 Hz, 1H), 3.82 (ddd, *J* = 9.2, 6.7, 3.4 Hz, 1H), 3.77 (t, *J* = 6.4 Hz, 1H), 3.46 (d, *J* = 6.8 Hz, 1H), 2.90 (d, *J* = 4.6 Hz, 1H), 2.32 (s, 3H), 2.10 (s, 3H).^{13}C NMR (101 MHz, Chloroform-*d*) δ 171.23, 167.24, 138.52, 133.69, 133.42, 130.21, 129.79, 129.51, 128.62, 86.30, 76.10, 73.87, 72.36, 68.97, 63.10, 21.30, 21.00. HRMS (ESI): Calcd. for $C_{22}H_{25}O_7S$ $[M+H]^+$ 433.1321, found 433.1322.

Synthesis of compound 8a

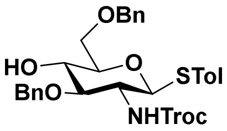

Compound **8a** was synthesised by following synthetic reported methods[2]. ^{1}H NMR (400 MHz, Chloroform-*d*) δ 7.41-7.28 (m, 12H), 7.05 (d, *J* = 7.9 Hz, 2H), 5.12 (d, *J* = 8.3 Hz, 1H), 4.91 (d, *J* = 10.3 Hz, 1H), 4.80-4.72 (m, 4H), 4.61-4.53 (m, 2H), 3.81-3.74 (m, 3H), 3.67 (t, *J* = 9.2 Hz, 1H), 3.51 (dt, *J* = 9.5, 4.7 Hz, 1H), 3.35 (q, *J* = 9.6 Hz, 1H), 2.77 (s, 1H), 2.31 (s, 3H). ^{13}C NMR (101 MHz, Chloroform-*d*) δ 153.95, 138.38, 138.11, 137.83, 133.34, 129.86, 128.73, 128.61, 128.32, 128.17, 127.99, 127.90, 86.21, 81.91, 77.97, 74.91, 74.58, 73.89, 73.10, 70.70, 56.18, 21.28. HRMS (ESI): Calcd. for $C_{30}H_{32}Cl_3NNaO_6S$ $[M+Na]^+$ 662.0914, found 662.0908.

Synthesis of compound 8

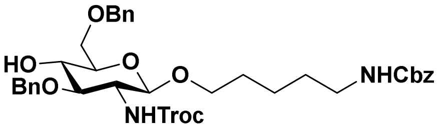

Donor **8a** (1.3 gm, 2.03 mmol) and 5-(Z-Amino)-1-pentanol (0.53 gm, 2.23 mmol) were dissolved in anhydrous dichloromethane (20 mL) and activated by 4 Å molecular sieves. The resulting mixture was stirred at room temperature for 2 h. Next, the reaction mixture was cooled to -20 °C, and NIS (0.59 gm, 2.63 mmol) and TfOH (36 μL, 0.40 mmol) were added and stirred 2 h more. The reaction mixture was neutralized with DIPEA and filtered through celite pad, and the filtrate was concentrated *in vacuo*. The residue was purified by silica gel column chromatography using a mixture of (1:1, v/v) ethyl acetate and hexane as eluent to afford compound **8** (1.24 gm, 81%). ^{1}H NMR (400 MHz, Chloroform-*d*) δ 7.27-7.41 (m, 15H), 5.40 (s, 1H), 5.09 (s, 2H), 4.78 (s, 2H), 4.71 (s, 2H), 4.62-4.54 (m, 3H), 3.87-3.82 (m, 1H), 3.74 (d, *J* = 4.9 Hz, 3H), 3.67 (m, 1H), 3.50-3.41 (m, 2H), 3.36-3.30 (m, 1H), 3.16 (q, *J* = 6.7 Hz, 2H), 2.84 (s, 1H), 1.71 (s, 1H), 1.61.1.53 (m, 2H), 1.49-1.45 (m, 2H), 1.39-1.32 (m, 2H). ^{13}C NMR (101 MHz, Chloroform-*d*) δ 156.59, 154.20, 138.38, 137.79, 136.75, 128.67, 128.61, 128.22, 128.04, 128.00, 127.90, 100.51, 95.73, 80.61, 74.49, 73.85, 73.75, 73.34, 70.70, 69.63, 66.73, 57.60, 41.04, 29.54, 28.96, 23.19. HRMS (ESI): Calcd. for $C_{36}H_{44}Cl_3N_2O_9$ $[M+H]^+$ 753.2112, found 753.2104.

2.1.4 Synthesis of Gc-2,6 trisaccharide M6.

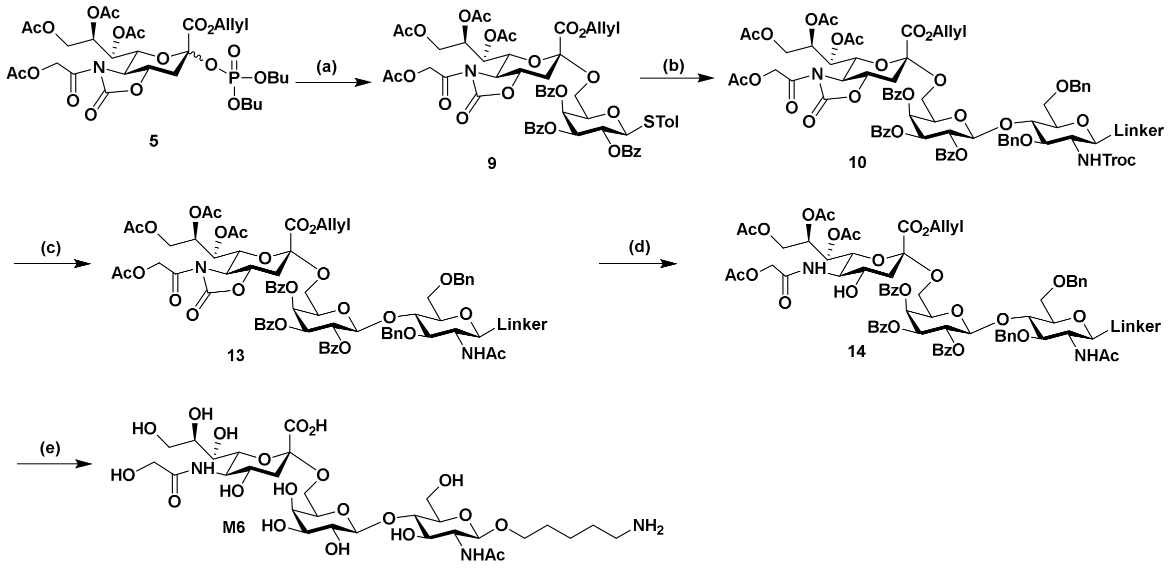

Scheme S4. Synthesis of 2,6 trisaccharide **M6**. Reagents and conditions: (a) **6**, TMSOTf, CH_2Cl_2, -50 °C, 2 h, 72%; (b) **8**, NIS, TfOH, CH_2Cl_2, -20 °C, 2 h, 71%; (c) Zn, THF/AcOH/Ac_2O (3:2:1, v/v), rt, 4 h, 65%; (d) 1,2-ethanedithiol, DBU, CH_2Cl_2, 0 °C, 2 h, 74%; (e) i) LiOH, THF/H_2O/MeOH (2:2:1, v/v), rt, 12 h; ii) $Pd(OH)_2$/C, H_2, H_2O/MeOH (3:1, v/v), rt, 48 h, 63% over two steps.

1) General Procedure A

Sialyl phosphate donor (1.73 mmol) and galactose acceptor (1.73 mmol) were dissolved in anhydrous dichloromethane (20 mL) and activated by 4 Å molecular sieves. The resulting mixture was stirred under an N_2 atmosphere at room temperature for 2 h. Next, the reaction mixture was cooled to -50 °C, followed by the addition of TMSOTf (2.07 mmol). The reaction mixture was stirred at the same temperature for 2 h, then neutralized with DIPEA, filtered and washed with brine. The organic layer was dried over sodium sulphate, concentrated in vacuo, and the residue was purified by silica gel column chromatography using a mixture of (3:2, v/v) ethyl acetate and hexane as eluent.

2) General Procedure B

Disaccharide donor (0.49 mmol) and glucose acceptor (0.58 mmol) were dissolved in anhydrous dichloromethane (10 mL), and activated by 4 Å molecular sieves. The resulting

mixture was stirred at room temperature for 2 h then cooled to -40 ^{o}C, followed by the addition of NIS (0.58 mmol) and TfOH (0.1 mmol). After being stirred at -20 ^{o}C for 2 h, the reaction mixture was neutralized with DIPEA, filtered through celite pad and the filtrate was concentrated *in vacuo*. The residue was purified by silica gel column chromatography using (7:3, v/v) ethyl acetate and hexane as eluent.

3) General Procedure C

Troc protected trisaccharide (0.23 mmol) was dissolved in a 3:2:1(v/v) mixture of THF/AcOH/Ac_2O (6 mL) and Zn (4.6 mmol). To this solution, a saturated aqueous solution of $CuSO_4$ (50 µL) was added, and the reaction mixture was stirred at room temperature for 4 h. Next, the reaction mixture was diluted with dichloromethane and filtered through celite pad, and the filtrate was concentrated *in vacuo*. The residue was purified by silica gel column chromatography by using (9:1, v/v) dichloromethane and methanol and as eluent.

4) General Procedure D

Trisaccharide (0.13 mmol) in anhydrous CH_2Cl_2 (5 mL) was mixed with 1,2-ethanedithiol (0.65 mmol) and DBU (0.065 mmol) at 0 ^{o}C and stirred at room temperature for 3 h and concentrated. The residue was purified by silica gel column chromatography using (4:2, v/v) dichloromethane and methanol and as eluent.

5) General Procedure E

Trisaccharide (43 µmole) in a 2:2:1 (v/v) in mixture of THF/ H_2O/MeOH (1.5 mL) was mixed with to LiOH (430 µmmol), and stirred at room temperature for 12 h. then reaction mixture was neutralized with Amberlite IR120 acidic resin, filtered and concentrated. The crude compound was desalted by using Bond elute C18 column using water and methanol as eluent. Next, the desalted compound was dissolved in a 3:1 (v/v) mixture of H_2O/MeOH (1.6 mL), and $Pd(OH)_2/C$ (250 mg) was added. The resulting mixture was stirred under a hydrogen atmosphere at room temperature for 48 hours, filtered and concentrated. The residue was purified by the Bond elute C18 column using water as eluent. The combined solvent fraction was pooled and lyophilized to yield a fully deprotected trisaccharide.

Compound 9

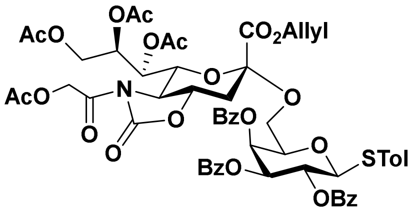

Compound **9** was synthesised from donor **5** and acceptor **6** using the general procedure **A** (72%). ^{1}H NMR (400 MHz, Chloroform-*d*) δ 7.97 (d, *J* = 7.2 Hz, 2H), 7.88 (d, *J* = 7.0 Hz, 2H), 7.73 (d, *J* = 7.1 Hz, 2H), 7.60 (t, *J* = 7.4 Hz, 1H), 7.51 (dd, *J* = 13.7, 7.7 Hz, 3H), 7.45-7.35 (m, 5H), 7.22 (t, *J* = 7.8 Hz, 2H), 7.16 (d, *J* = 8.0 Hz, 2H), 6.02 (d, *J* = 2.7 Hz, 1H), 5.83-5.72 (m, 1H), 5.72-5.67 (m, 2H), 5.66-5.61 (m, 1H), 5.53-5.46 (m, 1H), 5.32-5.19 (m, 2H), 5.15 (d, *J* = 9.4 Hz, 1H), 5.12-5.02 (m, 2H), 4.70 (dd, *J* = 9.6, 1.5 Hz, 1H), 4.57 (dd, *J* = 12.7, 6.3 Hz, 1H), 4.45 (td, *J* = 12.4, 4.4 Hz, 2H), 4.37 (t, *J* = 7.1 Hz, 1H), 4.14-3.99 (m, 2H), 3.92 (dd, *J* = 10.9, 6.1 Hz, 1H), 3.73 (d, *J* = 9.7 Hz, 1H), 3.70-3.62 (m, 1H), 2.76 (dd, *J* = 12.2, 3.6 Hz, 1H), 2.39 (s, 3H), 2.18 (s, 3H), 2.17 (s, 3H), 2.13 (s, 3H), 2.07-2.03 (m, 1H), 1.97 (s, 3H). ^{13}C NMR (101 MHz, Chloroform-*d*) δ 171.07, 170.66, 170.49, 170.19, 168.22, 167.41, 165.54, 165.28, 153.52, 138.37, 134.12, 133.40, 133.31, 133.22, 130.62, 130.05, 129.96, 129.86, 129.66, 129.12, 128.56, 128.49, 128.35, 127.79, 120.82, 99.79, 85.34, 76.21, 75.46, 75.10, 73.29, 71.44, 68.61, 68.19, 67.11, 63.68, 59.44, 36.57, 21.50, 21.24, 21.04, 20.83, 20.62. HRMS (ESI): Calcd. for $C_{57}H_{58}NO_{22}S$ $[M+H]^+$ 1140.3171, found 1140.3164.

Compound 10

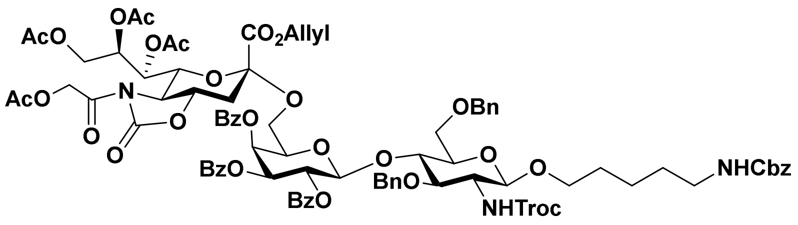

Compound 10 was synthesised from donor 9 and acceptor 8 using the general procedure B (72%). ^{1}H NMR (400 MHz, Chloroform-*d*) δ 7.94 (d, *J* = 7.2 Hz, 2H), 7.89 (dd, *J* = 8.3, 1.1 Hz, 2H), 7.76 (dd, *J* = 8.4, 1.1 Hz, 2H), 7.55 (t, *J* = 7.4 Hz, 1H), 7.49 (t, *J* = 7.4 Hz, 1H), 7.41 (dd, *J* = 8.3, 1.7 Hz, 4H), 7.38-7.28 (m, 14H), 7.26 (d, *J* = 6.7 Hz, 2H), 7.23 (d, *J* = 7.7 Hz, 2H), 5.92 (d, *J* = 3.3 Hz, 1H), 5.74-5.63 (m, 3H), 5.52 (dd, *J* = 10.5, 3.5 Hz, 1H), 5.46 (dq, *J* = 9.2, 3.0 Hz, 2H), 5.24-5.12 (m, 2H), 5.12-4.98 (m, 6H), 4.82 (d, *J* = 11.4 Hz, 2H), 4.68 (s, 2H), 4.63 (dd, *J* = 9.6, 1.4 Hz, 1H), 4.59 (d, *J* = 12.2 Hz, 1H), 4.53 (dd, *J* = 12.7, 6.4 Hz, 1H), 4.46 (s, 1H), 4.41-4.33 (m, 2H), 4.23 (dd, *J* = 12.7, 5.9 Hz, 1H), 4.15-3.95 (m, 4H), 3.88 (td, *J* = 10.7, 5.7 Hz, 2H), 3.76 (dt, *J* = 9.5, 6.1 Hz, 1H), 3.68-3.53 (m, 4H), 3.45 (q, *J* = 8.5 Hz, 1H), 3.37 (s, 1H), 3.33-3.25 (m, 1H), 3.14 (q, *J* = 6.6 Hz, 2H), 2.75-2.71 (m, 1H), 2.17 (s, 3H), 2.16-2.12 (m, 1H), 2.08 (s, 3H), 2.07 (s, 3H), 2.00 (s, 3H), 1.48 (m, 4H), 1.32 (q, *J* = 7.1, 6.6 Hz, 2H). ^{13}C NMR (101 MHz, Chloroform-*d*) δ 171.00, 170.60, 170.22, 170.15, 168.29, 167.15, 165.55, 165.35, 165.32, 156.53, 154.20, 153.54, 138.84, 138.34, 136.85, 133.47, 133.30, 130.64, 129.91, 129.86, 129.64, 129.33, 129.10, 128.71, 128.64, 128.61, 128.44, 128.39, 128.19, 128.13, 128.03, 127.95, 127.65, 120.71, 100.08, 99.63, 76.23, 75.09, 74.80, 74.55, 73.84, 73.50, 72.04, 71.85, 71.59, 70.66, 69.48, 68.70, 68.62, 68.17, 67.08, 66.71, 63.72, 63.48, 63.31, 59.48, 57.14, 41.08, 36.35, 29.60, 29.03, 23.24, 21.19, 20.93, 20.86, 20.57. HRMS (ESI): Calcd. for $C_{86}H_{94}Cl_3N_3O_{31}$ $[M+H]^+$ 1768.4859, found 1768.4856

Compound 13

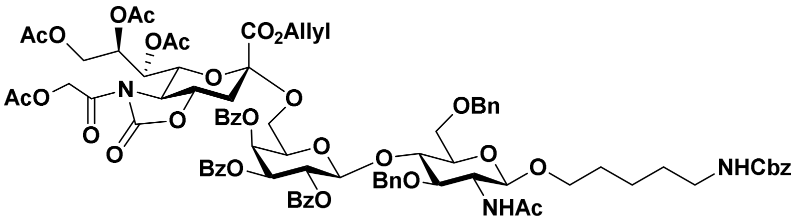

Compound **13** was synthesised from **12** using the general procedure **C** (65%). ^{1}H NMR (400 MHz, Chloroform-*d*) δ 7.97 (d, *J* = 7.0 Hz, 2H), 7.90 (d, *J* = 7.0 Hz, 2H), 7.75 (d, *J* = 7.0 Hz, 2H), 7.57 (t, *J* = 7.5 Hz, 1H), 7.50 (t, *J* = 7.6 Hz, 1H), 7.44-7.30 (m, 14H), 7.33-7.26 (m, 5H), 7.29-7.18 (m, 3H), 6.06 (d, *J* = 8.4 Hz, 1H), 5.94 (dd, *J* = 3.3, 1.1 Hz, 1H), 5.77-5.62 (m, 3H), 5.60 (dd, *J* = 10.4, 3.4 Hz, 1H), 5.45 (ddd, *J* = 8.5, 6.7, 3.0 Hz, 1H), 5.26-5.14 (m, 2H), 5.08-5.03 (m, 4H), 5.00 (d, *J* = 7.8 Hz, 1H), 4.91 (d, *J* = 11.6 Hz, 2H), 4.77 (d, *J* = 11.7 Hz, 1H), 4.64 (dd, *J* = 9.6, 1.6 Hz, 1H), 4.60-4.49 (m, 3H), 4.44-4.33 (m, 2H), 4.29 (dd, *J* = 12.7, 6.0 Hz, 1H), 4.15-3.92 (m, 5H), 3.90 (dd, *J* = 10.8, 5.9 Hz, 1H), 3.72-3.57 (m, 6H), 3.51 (dt, *J* = 8.1, 4.5 Hz, 1H), 3.19 (dt, *J* = 9.7, 6.5 Hz, 1H), 3.12 (q, *J* = 6.5 Hz, 2H), 2.73 (dd, *J* = 12.3, 3.6 Hz, 1H), 2.17 (s, 3H), 2.07 (s, 3H), 2.06 (s, 3H), 1.98 (s, 3H), 1.92 (s, 3H), 1.45 (m, 4H), 1.28 (m, 2H). ^{13}C NMR (101 MHz, Chloroform-*d*) δ 171.02, 170.62, 170.37, 170.25, 170.11, 168.22, 167.15, 165.66, 165.50, 165.34, 156.54, 153.55, 138.98, 138.31, 136.82, 133.54, 133.50, 133.32, 130.60, 129.90, 129.84, 129.60, 129.26, 129.03, 128.71, 128.62 , 128.41, 128.38, 128.20, 128.16, 127.98, 127.96, 127.62, 120.75, 100.31, 99.84, 99.66, 78.04, 76.21, 75.83 , 75.02 74.72, 73.44, 73.33, 72.05, 71.57, 71.50, 70.60, 69.14, 68.57, 68.16, 67.09, 66.64, 63.71, 63.59, 63.34, 59.45, 54.27, 41.06, 36.38, 29.83, 29.60, 28.96, 23.49, 23.32, 21.18, 20.92, 20.85, 20.59. HRMS (ESI): Calcd. for HRMS (ESI): Calcd. for $C_{85}H_{93}N_{3}NaO_{30}$ $[M+Na]^{+}$ 1658.5742, found 1658.5736.

Compound 14

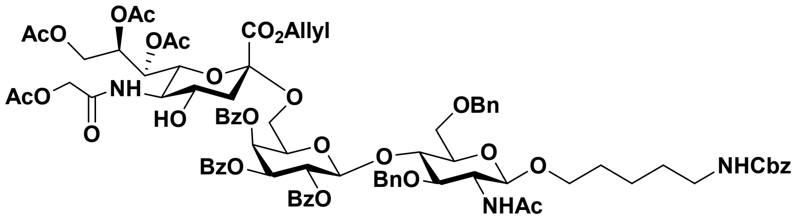

Compound **14** was synthesised from **13** using the general procedure **D** (74%). ^{1}H NMR (400 MHz, Chloroform-*d*) δ 7.95 (d, *J* = 7.0 Hz, 2H), 7.90 (d, *J* = 7.0 Hz, 2H), 7.76 (d, *J* = 7.0 Hz, 2H), 7.56-7.47 (m, 2H), 7.42-7.30 (m, 19H), 7.27-7.20 (m, 3H), 6.44 (d, *J* = 7.7 Hz, 1H), 6.03 (d, *J* = 8.5 Hz, 1H), 5.91 (dd, *J* = 3.4, 1.1 Hz, 1H), 5.77-5.61 (m, 2H), 5.54 (dd, *J* = 10.4, 3.4 Hz, 1H), 5.40 (ddd, *J* = 8.9, 4.9, 2.6 Hz, 1H), 5.24-5.10 (m, 3H), 5.07 (s, 2H), 5.01-4.87 (m, 3H), 4.81 (d, *J* = 11.9 Hz, 1H), 4.61-4.46 (m, 4H), 4.45-4.35 (m, 2H), 4.33-4.25 (m, 2H), 4.19-4.07 (m, 3H), 4.06-3.87 (m, 4H), 3.74 (q, *J* = 7.6 Hz, 1H), 3.68-3.62 (m, 3H), 3.57 (dd, *J* = 10.5, 7.7 Hz, 1H), 3.49 (dt, *J* = 6.8, 4.5 Hz, 1H), 3.32 (td, *J* = 10.3, 7.7 Hz, 1H), 3.22-3.06 (m, 4H), 2.62 (dd, *J* = 13.1, 4.4 Hz, 1H), 2.18 (s, 3H), 2.08 (s, 3H), 2.05 (s, 3H), 2.00 (s, 3H), 1.91 (s, 3H), 1.79 (t, *J* = 12.5 Hz, 1H), 1.44 (h, *J* = 7.1, 6.3 Hz, 4H), 1.27 (m, 2H). ^{13}C NMR (101 MHz, Chloroform-*d*) δ 171.04, 170.71, 170.31, 169.83, 169.80, 168.39, 167.09, 165.56, 165.39, 165.13, 156.46, 138.74, 138.19, 136.72, 133.44, 133.29, 133.17, 130.95, 129.80, 129.73, 129.53, 129.15, 128.98, 128.55, 128.51, 128.36, 128.25, 128.08, 127.91, 127.88, 127.54, 119.72, 100.34, 99.70, 99.46, 75.52, 74.65, 73.38, 73.04, 72.07, 71.61, 71.56, 70.49, 69.00, 68.13, 67.98, 67.86, 66.56, 63.08, 62.58, 62.26, 54.17, 53.96, 40.95, 40.47, 29.46, 28.82, 23.37, 23.20, 20.99, 20.72. HRMS (ESI): Calcd. for $C_{84}H_{96}N_3O_{29}$ $[M+H]^+$ 1610.6129, found 1610.6113.

Compound M6

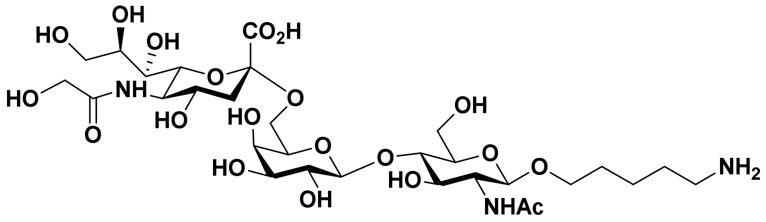

Compound **M6** was synthesised from **14** using the general procedure **E** (63%).^{1}H NMR (400 MHz, Deuterium Oxide) δ 4.56 (d, *J* = 8.2 Hz, 1H), 4.46 (d, *J* = 7.9 Hz, 1H), 4.12 (s, 2H), 4.01 (t, *J* = 10.0 Hz, 2H), 3.95-3.91 (m, 2H), 3.90-3.85 (m, 3H), 3.84-3.80 (m, 3H), 3.79-3.71 (m, 3H), 3.70-3.58 (m, 5H), 3.57-3.52 (m, 3H), 3.05-2.95 (m, 2H), 2.69 (dd, *J* = 12.3, 4.6 Hz, 1H), 2.07 (s, 3H), 1.77-1.58 (m, 5H), 1.45-1.38 (m, 2H). ^{13}C NMR (151 MHz, Deuterium Oxide) δ 175.63, 174.41, 172.72, 103.43, 100.88, 99.65, 80.73, 74.41, 73.60, 72.40, 72.36, 72.32, 71.41, 70.64, 70.00, 68.30, 67.65, 63.34, 62.68, 60.93, 60.29, 54.81, 51.45, 39.74, 39.28, 28.01, 26.31, 22.23, 22.08. HRMS (ESI): Calcd. for $C_{30}H_{54}N_3O_{20}$ $[M+H]^+$ 776.3301, found 776.3306.

2.1.5 Synthesis of Gc-2,3 trisaccharide M3

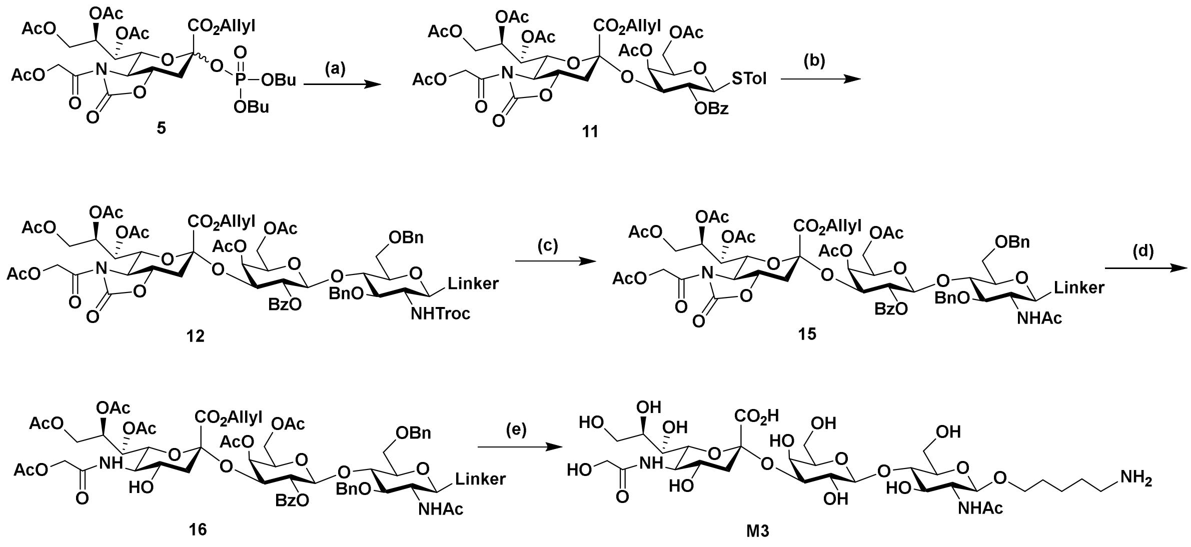

Scheme S5. Synthesis of 2,3 trisaccharide M3. Reagents and conditions: (a) 7, TMSOTf, CH_2Cl_2, -50 °C, 2 h; ii) Ac_2O, pyridine, rt, 12 h, 49% over two steps; (b) 8, NIS, TfOH, CH_2Cl_2, -20 °C, 2 h, 68%; (c) Zn, THF/AcOH/Ac_2O (3:2:1, v/v), rt, 4 h, 70%; (d) 1,2 ethanedithiol, DBU, CH_2Cl_2, 0 °C, 2 h, 75%; (e) i) LiOH, THF/H_2O/MeOH (2:2:1, v/v), rt, 12 h; ii) $Pd(OH)_2$/C, H_2, H_2O/MeOH (3:1, v/v), 48 h, 59% over two steps.

Compound 10

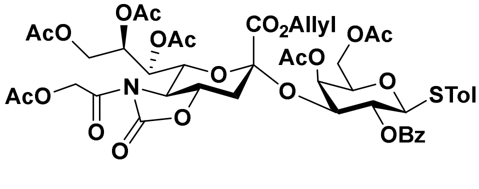

Compound 10 was synthesised from donor 5 and acceptor 7 using the general procedure A. Further, the compound was dissolved in pyridine (10 mL), and acetic anhydride was added (0.5 mL) at 0 °C. The resulting mixture was stirred at RT for 12 hours, concentrated *in vacuo* and purified using mixture of (3:2, v/v) ethyl acetate and hexane as eluent to afford compound 10 (49% over two steps). ^{1}H NMR (400 MHz, Chloroform-*d*) δ 8.24-8.15 (m, 2H), 7.59 (t, *J* = 7.4 Hz, 1H), 7.48 (t, *J* = 7.6 Hz, 2H), 7.35 (d, *J* = 8.1 Hz, 2H), 7.06 (d, *J* = 7.8 Hz, 2H), 5.96 (ddt, *J* = 17.0, 10.3, 6.4 Hz, 1H), 5.61 (ddd, *J* = 9.5, 7.2, 2.4 Hz, 1H), 5.50 (dd, *J* = 9.4, 2.1 Hz, 1H), 5.40 (dq, *J* = 17.2, 1.4 Hz, 1H), 5.35 (dq, *J* = 10.3, 1.1 Hz, 1H), 5.28 (t, *J* = 9.8

Hz, 1H), 5.06-4.88 (m, 4H), 4.81 (dd, *J* = 12.6, 6.5 Hz, 1H), 4.76 (dd, *J* = 9.7, 3.2 Hz, 1H), 4.58 (ddt, *J* = 12.6, 6.1, 1.3 Hz, 1H), 4.35 (dd, *J* = 12.3, 2.5 Hz, 1H), 4.26 (dd, *J* = 9.6, 2.2 Hz, 1H), 4.08 (d, *J* = 6.3 Hz, 2H), 4.02-3.96 (m, 2H), 3.94-3.87 (m, 1H), 3.43 (dd, *J* = 11.4, 9.6 Hz, 1H), 2.91 (dd, *J* = 11.8, 3.5 Hz, 1H), 2.31 (s, 3H), 2.16 (s, 3H), 2.13 (s, 3H), 2.09 (s, 3H), 2.06 (s, 3H), 1.98 (s, 3H), 1.86 (dd, *J* = 13.3, 11.9 Hz, 1H), 1.24 (s, 3H). ^{13}C NMR (101 MHz, Chloroform-*d*) δ 170.91, 170.82, 170.67, 170.46, 170.28, 169.84, 167.70, 166.85, 165.61, 153.30, 138.29, 133.57, 133.50, 130.75, 130.56, 130.31, 129.56, 128.72, 128.67, 121.08, 97.29, 86.83, 76.98, 73.80, 72.66, 71.38, 69.44, 68.16, 68.05, 67.90, 63.59, 63.50, 62.45, 59.24, 36.18, 21.65, 21.28, 20.87, 20.84, 20.80, 20.57, 20.00. HRMS (ESI): Calcd. for $C_{47}H_{54}NO_{22}S$ $[M+H]^+$ 1016.2858, found: 1016.2855.

Compound 12

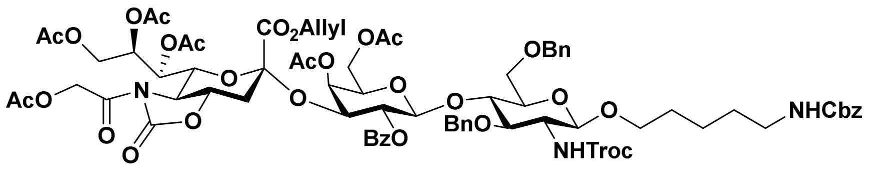

Compound **12** was synthesised from donor **7** and acceptor **8** using the general glycosylation procedure B (69%). ^{1}H NMR (400 MHz, Chloroform-*d*) δ 8.24 (d, *J* = 7.2 Hz, 2H), 7.56 (t, *J* = 7.4 Hz, 1H), 7.46 (t, *J* = 7.6 Hz, 2H), 7.37-7.2 (m, 15H), 5.96 (ddt, *J* = 16.9, 10.2, 6.3 Hz, 1H), 5.69 (ddd, *J* = 10.2, 7.4, 2.5 Hz, 1H), 5.53 (dd, *J* = 9.5, 2.2 Hz, 1H), 5.45-5.29 (m, 4H), 5.11-4.93 (m, 6H), 4.91 (d, *J* = 10.2 Hz, 1H), 4.81 (dd, *J* = 12.4, 6.6 Hz, 2H), 4.72-4.68 (m, 2H), 4.68-4.53 (m, 3H), 4.45-4.35 (m, 4H), 4.26 (dd, *J* = 9.6, 2.2 Hz, 1H), 4.10-3.90 (m, 4H), 3.88-3.74 (m, 3H), 3.71-3.63 (m, 2H), 3.59-3.51 (m, 1H), 3.47-3.35 (m, 3H), 3.21 (s, 1H), 3.12 (q, *J* = 6.8 Hz, 2H), 2.89 (dd, *J* = 11.9, 3.4 Hz, 1H), 2.17 (s, 3H), 2.14 (s, 3H), 2.05 (s, 3H), 1.96 (s, 3H), 1.91 (s, 3H), 1.87 (d, *J* = 11.6 Hz, 1H), 1.53-1.36 (m, 4H), 1.32-1.27 (m, 5H). ^{13}C NMR (101 MHz, Chloroform-*d*) δ 170.85 , 170.66, 170.55, 170.19, 170.06, 169.69, 167.63, 166.59, 165.48, 153.17, 138.45, 136.65 , 133.54 , 130.61 , 130.38 , 129.86 , 128.67 , 128.51 , 128.21 (d, *J* = 1.4 Hz), 128.06 , 127.75 , 127.49 , 127.38, 127.24, 120.96, 99.86, 97.38, 76.85, 74.69, 73.61, 72.91, 71.85, 71.55, 71.33, 70.77, 69.30, 67.79, 67.68, 66.56, 63.51, 63.38, 61.80, 59.13, 40.91, 38.74, 36.64, 35.94, 31.93, 29.70, 29.44, 29.36, 24.69, 21.48, 20.67, 20.65,

20.58, 20.43, 20.04. HRMS (ESI): Calcd. for $C_{76}H_{89}Cl_3N_3O_{31}$ $[M+H]^+$ 1644.4546, found: 1644.4545.

Compound 15

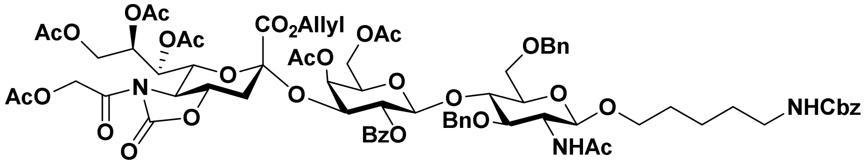

Compound **15** was synthesised from **12** using the general Troc deprotection procedure **C** (70%). ^{1}H NMR (400 MHz, Chloroform-*d*) δ 8.22 (d, *J* = 1.2 Hz, 2H), 7.57 (t, *J* = 7.4 Hz, 1H), 7.46 (t, *J* = 7.6 Hz, 2H), 7.36-7.20 (m, 15H), 6.01-5.87 (m, 2H), 5.69 (ddd, *J* = 9.9, 7.5, 2.5 Hz, 1H), 5.53 (dd, *J* = 9.5, 2.2 Hz, 1H), 5.40 (dq, *J* = 17.2, 1.4 Hz, 1H), 5.35 (dd, *J* = 10.2, 1.2 Hz, 2H), 5.13-4.90 (m, 7H), 4.81 (ddt, *J* = 12.6, 6.6, 1.1 Hz, 1H), 4.77-4.71 (m, 2H), 4.65 (d, *J* = 11.8 Hz, 1H), 4.59 (ddt, *J* = 12.6, 6.0, 1.2 Hz, 1H), 4.48-4.37 (m, 3H), 4.34 (d, *J* = 6.2 Hz, 1H), 4.27 (dd, *J* = 9.6, 2.2 Hz, 1H), 4.08 (t, *J* = 5.5 Hz, 1H), 4.04-3.97 (m, 2H), 3.96-3.90 (m, 1H), 3.90-3.85 (m, 2H), 3.83 (t, *J* = 5.9 Hz, 1H), 3.71 (td, *J* = 7.7, 4.8 Hz, 2H), 3.59 (tdd, *J* = 12.3, 9.2, 5.7 Hz, 3H), 3.46 (dd, *J* = 11.4, 9.6 Hz, 1H), 3.10 (q, *J* = 6.7 Hz, 2H), 2.98 (dt, *J* = 9.6, 6.5 Hz, 1H), 2.89 (dd, *J* = 11.8, 3.5 Hz, 1H), 2.17 (s, 3H), 2.14 (s, 3H), 2.08 (s, 3H), 1.96 (s, 3H), 1.93 (s, 3H), 1.91 (s, 3H), 1.87 (d, *J* = 12.3 Hz, 1H), 1.42-1.38 (m, 4H), 1.32 (s, 3H), 1.28-1.19 (m, 2H). ^{13}C NMR (101 MHz, Chloroform-*d*) δ 170.99, 170.82, 170.69, 170.34, 170.20, 169.84, 167.81, 166.71, 166.18, 156.56, 153.29, 138.72, 138.53, 136.82, 133.74, 130.72, 130.46, 129.95, 128.82, 128.61, 128.33, 128.17, 127.72, 127.58, 127.52, 127.37, 121.09, 100.28, 99.39, 97.44, 78.06, 74.46, 73.82, 73.04, 72.79, 71.70, 71.49, 71.38, 70.94, 69.81, 69.09, 67.94, 67.85, 66.62, 63.69, 63.51, 61.88, 59.22, 41.04, 36.08, 29.81, 29.57, 28.91, 23.39, 23.33, 21.62, 20.82, 20.74, 20.70, 20.56, 20.23. HRMS (ESI): Calcd. for $C_{75}H_{90}N_3O_{30}$ $[M+H]^+$ 1512.5609, found: 1512.5599.

Compound 16

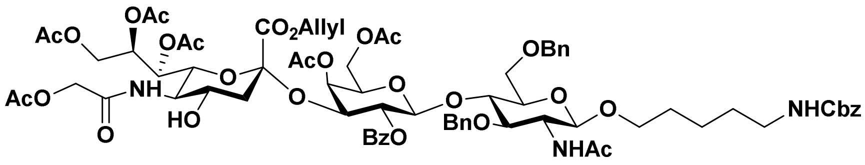

Compound **16** was synthesised from **15** using the general oxazolidinone ring deprotection procedure **D** (75%). ^{1}H NMR (400 MHz, Chloroform-*d*) δ 8.15 (d, *J* = 7.3 Hz, 2H), 7.56 (t, *J* = 7.3 Hz, 1H), 7.46 (t, *J* = 7.6 Hz, 2H), 7.35-7.28 (m, 12H), 7.27-7.22 (m, 3H), 6.17 (d, *J* = 8.4 Hz, 1H), 6.03-5.88 (m, 2H), 5.66-5.57 (m, 1H), 5.38-5.24 (m, 3H), 5.16 (dd, *J* = 9.4, 2.4 Hz, 1H), 5.06 (s, 2H), 5.03 (d, *J* = 3.4 Hz, 1H), 4.98 (s, 1H), 4.92 (d, *J* = 7.9 Hz, 1H), 4.80-4.68 (m, 3H), 4.64 (d, *J* = 11.7 Hz, 1H), 4.60-4.43 (m, 4H), 4.37-4.26 (m, 3H), 4.10-3.99 (m, 3H), 3.96-3.76 (m, 4H), 3.77 (dd, *J* = 10.7, 2.4 Hz, 1H), 3.71 (dd, *J* = 9.9, 5.0 Hz, 2H), 3.64 (dd, *J* = 10.0, 5.0 Hz, 1H), 3.57 (dt, *J* = 10.1, 5.8 Hz, 2H), 3.24 (q, *J* = 10.1 Hz, 1H), 3.09 (q, *J* = 6.5 Hz, 2H), 3.03-2.87 (m, 2H), 2.62 (dd, *J* = 12.7, 4.5 Hz, 1H), 2.16 (s, 3H), 2.12 (s, 3H), 2.06 (s, 3H), 1.99 (s, 3H), 1.94 (s, 3H), 1.91 (s, 3H), 1.40 (m, 4H), 1.26 (m, 5H). ^{13}C NMR (101 MHz, Chloroform-*d*) δ 170.78, 170.52, 170.38, 170.30, 170.21, 169.88, 168.51, 167.57, 165.89, 156.56, 138.70, 138.48, 136.78, 133.46, 131.28, 130.21, 130.08, 128.71, 128.57, 128.32, 128.12, 127.75, 127.54, 127.42, 119.89, 100.36, 99.41, 97.22, 78.01, 74.57, 74.38, 73.04, 72.82, 71.67, 70.97, 70.83, 69.54, 69.06, 67.93, 67.48, 67.41, 67.30, 66.58, 63.16, 62.38, 61.92, 53.36, 53.06, 41.01, 40.31. HRMS (ESI): Calcd. for $C_{74}H_{92}N_3O_{29}$ $[M+H]^+$ 1486.5816, found: 1486.5808.

Compound M3

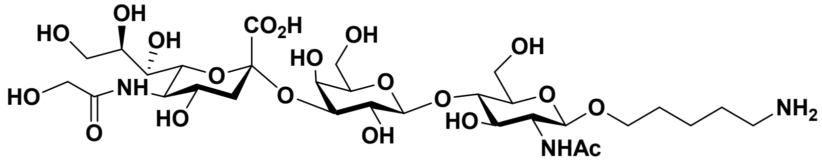

Compound **M3** was synthesised from **16** using the general global deprotection procedure **E** (59%). ^{1}H NMR (400 MHz, Deuterium Oxide) δ 4.56 (d, *J* = 7.9 Hz, 1H), 4.52 (d, *J* = 7.5 Hz, 1H), 4.19-4.07 (m, 3H), 4.04-3.76 (m, 8H), 3.80-3.68 (m, 7H), 3.69-3.53 (m, 5H), 2.99 (dd, *J* = 8.9, 6.4 Hz, 2H), 2.78 (dd, *J* = 12.4, 4.6 Hz, 1H), 2.03 (s, 3H), 1.82 (t, *J* = 12.1 Hz, 1H), 1.74-1.54 (m, 4H), 1.41 (m, 2H). ^{13}C NMR (101 MHz, Deuterium Oxide) δ 175.75, 174.37, 173.85, 102.54, 101.11, 99.80, 78.30, 75.46, 75.16, 74.74, 72.58, 72.35, 71.80, 70.05, 69.36, 68.05, 67.99, 67.44, 62.53, 61.01, 60.95, 60.02, 55.06, 51.36, 39.67, 39.30, 28.04, 26.33, 22.13, 22.07. HRMS (ESI): Calc. for $C_{30}H_{54}N_{3}O_{20}$ $[M+H]^{+}$ 776.3301, found: 776.3294.

2.1.6 Synthesis of Ac-2,6 trisaccharide H6.

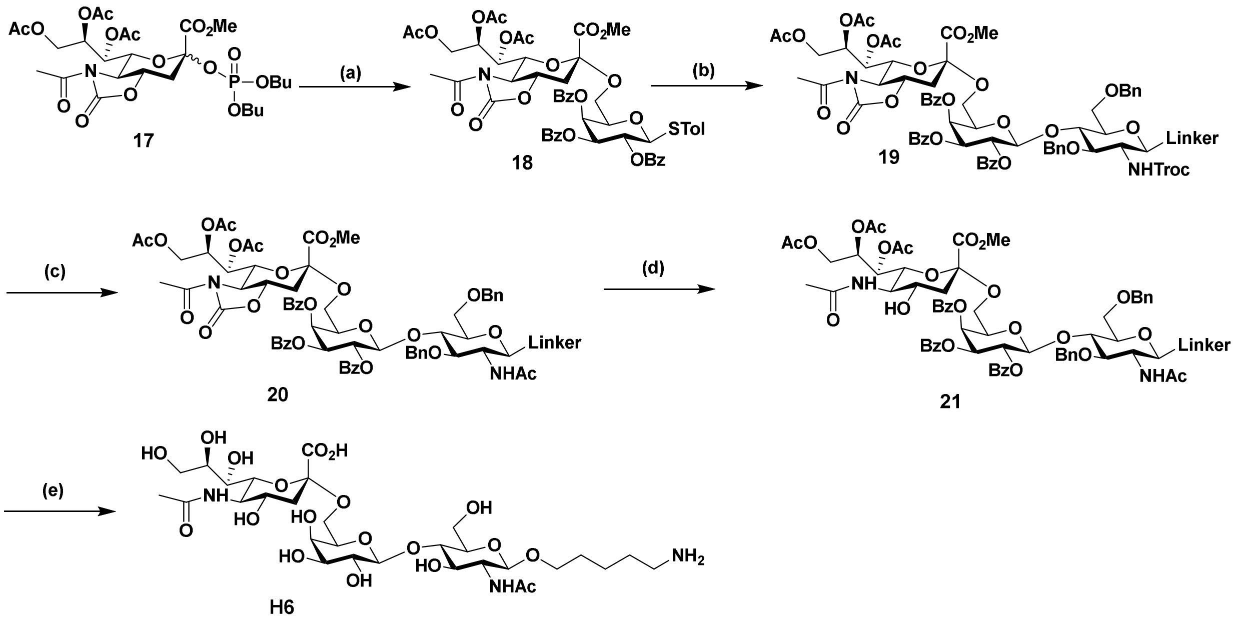

Scheme S6. Synthesis of Ac-2,6 trisaccharide **H6**. Reagents and conditions: (a) **6**, TMSOTf, CH_2Cl_2, -50 °C, 2 h, 83%; (b) **8**, NIS, TfOH, CH_2Cl_2, -20 °C, 2 h, 76%; (c) Zn,.THF/AcOH/Ac_2O (3:2:1 v/v), rt, 4 h, 69%; (d) 1,2-ethanedithiol, DBU, CH_2Cl_2, 0 °C, 2 h, 81%; (e) i) LiOH, THF/H_2O/MeOH (2:2:1, v/v), rt, 12 h; ii) $Pd(OH)_2$/C, H_2, H_2O/MeOH (3:1, v/v), rt, 48 h, 65% over two steps.

Compound 17

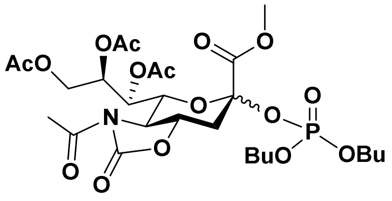

Compound **17** was synthesised by following synthetic reported methods[3]. ^{1}H NMR (400 MHz, Chloroform-*d*) δ 5.66 (dd, *J* = 7.5, 1.5 Hz, 1H), 5.32-5.28 (m, 1H), 4.74 (dd, *J* = 9.6, 1.6 Hz, 1H), 4.39 (dd, *J* = 12.3, 2.8 Hz, 1H), 4.20-4.04 (m, 6H), 3.83 (s, 3H), 2.99 (dd, *J* = 12.2, 4.0 Hz, 1H), 2.74 (s, 1H), 2.67 (t, *J* = 12.7 Hz, 1H), 2.49 (s, 3H), 2.13 (s, 3H), 2.09 (s, 3H), 2.03 (s,

3H), 1.70-1.62 (m, 4H), 1.40 (h, *J* = 7.4 Hz, 4H), 0.93 (t, *J* = 7.4 Hz, 6H). ^{13}C NMR (101 MHz, Chloroform-*d*) δ 171.96, 170.75, 170.11, 170.01, 167.36, 153.65, 98.34, 77.35, 74.31, 71.60, 68.22, 62.63, 58.45, 53.58, 36.00, 32.23, 29.71, 24.76, 21.09, 20.92, 20.87, 18.76, 13.69. HRMS (ESI): Calcd. for $C_{27}H_{42}NNaO_{16}P$ [M+Na]$^+$ 690.2139, found 690.2131.

Compound 18

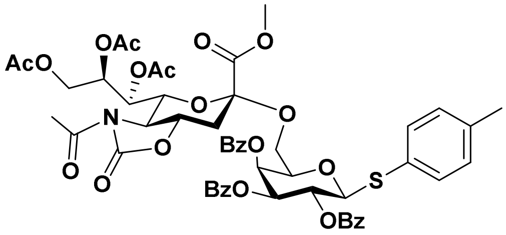

Compound 18 was synthesised from donor 17 and acceptor 6 using the general glycosylation procedure A (83%). ^{1}H NMR (400 MHz, Chloroform-*d*) δ 7.98-7.96 (m, 2H), 7.90-7.87 (m, 2H), 7.75-7.73 (m, 2H), 7.62-7.58 (m, 1H), 7.54-7.48 (m, 3H), 7.45-7.36 (m, 5H), 7.24-7.20 (m, 2H), 7.18-7.15 (m, 2H), 6.01 (q, *J* = 1.2 Hz, 1H), 5.65-5.63 (m, 2H), 5.60 (dd, *J* = 8.3, 1.7 Hz, 1H), 5.49 (ddd, *J* = 8.3, 7.2, 2.7 Hz, 1H), 5.13-5.10 (m, 1H), 4.63 (dd, *J* = 9.4, 1.7 Hz, 1H), 4.50 (dd, *J* = 12.3, 2.8 Hz, 1H), 4.34 (ddd, *J* = 8.4, 5.9, 1.2 Hz, 1H), 4.06 (dd, *J* = 12.3, 7.2 Hz, 1H), 3.97-3.90 (m, 2H), 3.72 (dd, *J* = 11.2, 9.4 Hz, 1H), 3.60 (dd, *J* = 10.5, 8.3 Hz, 1H), 3.52 (s, 3H), 2.74 (dd, *J* = 12.2, 3.5 Hz, 1H), 2.48 (s, 3H), 2.39 (s, 3H), 2.19 (s, 3H), 2.13 (s, 3H), 2.06 (m, 1H), 1.99 (s, 3H). ^{13}C NMR (101 MHz, Chloroform-*d*) δ 172.16, 171.03, 170.59, 170.22, 168.38, 165.60, 165.26, 165.24, 153.77, 138.50, 134.36, 133.43, 133.34, 133.23, 130.04, 129.96, 129.87, 129.67, 129.64, 129.62, 129.11, 128.58, 128.50, 128.35, 127.63, 99.69, 85.47, 75.96, 75.44, 74.90, 73.34, 71.74, 69.07, 68.12, 63.65, 63.57, 59.08, 53.10, 36.54, 24.86, 21.50, 21.22, 21.09, 20.90. HRMS (ESI): Calcd. for $C_{53}H_{54}NO_{20}S$ [M+H]$^+$ 1056.2960, found 1056.2956.

Compound 19

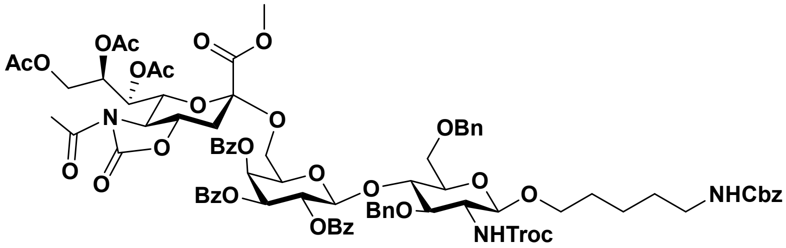

Compound 19 was synthesised from donor 18 and acceptor 8 using the general glycosylation procedure B (76%). ^{1}H NMR (400 MHz, Chloroform-*d*) δ 7.94-7.92 (m, 2H), 7.89-7.87 (m, 2H), 7.77-7.75 (m, 2H), 7.56-7.47 (m, 2H), 7.44-7.38 (m, 5H), 7.37-7.21 (m, 17H), 5.90 (d, *J* = 3.5 Hz, 1H), 5.69 (dd, *J* = 10.4, 8.0 Hz, 1H), 5.58 (dd, *J* = 7.8, 1.7 Hz, 2H), 5.50-5.45 (m, 2H), 5.12-5.04 (m, 4H), 4.86-4.81 (m, 2H), 4.67 (s, 2H), 4.61-4.56 (m, 2H), 4.43 (dd, *J* = 12.2, 2.9 Hz, 2H), 4.35 (d, *J* = 12.2 Hz, 1H), 4.10-4.05 (m, 2H), 3.98-3.86 (m, 4H), 3.79-3.73 (m, 1H), 3.68-3.59 (m, 4H), 3.54-3.42 (m, 1H), 3.37-3.28 (m, 5H), 3.13 (q, *J* = 6.7 Hz, 2H), 2.69 (d, *J* = 3.5 Hz, 1H), 2.47 (s, 3H), 2.09 (s, 3H), 2.08 (s, 3H), 1.99 (s, 3H), 1.54-1.50 (m, 2H), 1.47-1.42 (m, 2H), 1.34-1.27 (m, 2H). ^{13}C NMR (101 MHz, Chloroform-*d*) δ 172.18, 171.07, 170.33, 170.23, 168.14, 165.58, 165.26, 153.79, 138.85, 138.24, 136.77, 133.48, 133.30, 129.86, 129.83, 129.50, 129.22, 129.02, 128.71, 128.65, 128.63, 128.41, 128.37, 128.20, 128.17, 128.04, 127.98, 127.95, 127.58, 100.03, 99.43, 76.60, 75.93, 74.87, 74.63, 74.44, 73.86, 73.47, 71.82, 70.52, 69.47, 69.11, 68.47, 67.92, 66.66, 63.30, 63.01, 59.10, 53.05, 41.02, 36.38, 29.68, 29.55, 28.97, 24.83, 23.19, 21.17, 21.00, 20.94. HRMS (ESI): Calcd. for $C_{82}H_{89}Cl_{3}N_{3}O_{29}$ $[M+H]^{+}$ 1684.4647, found 1684.4661.

Compound 20

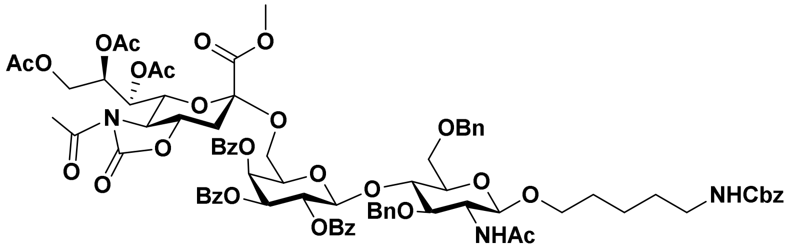

Compound **20** was synthesised from **19** using the general Troc deprotection procedure **C** (69%). ^{1}H NMR (400 MHz, Chloroform-*d*) δ 7.98-7.95 (m, 2H), 7.91-7.89 (m, 2H), 7.77-7.75 (m, 2H), 7.58-7.54 (m, 1H), 7.52-7.48 (m, 1H), 7.45-7.21 (m, 22H), 6.08 (d, *J* = 8.4 Hz, 1H), 5.92 (dd, *J* = 3.5, 1.1 Hz, 1H), 5.67 (dd, *J* = 10.5, 7.9 Hz, 1H), 5.59-5.53 (m, 2H), 5.46 (ddd, *J* = 8.0, 7.0, 2.9 Hz, 1H), 5.07 (s, 2H), 4.99 (d, *J* = 7.9 Hz, 1H), 4.93 (d, *J* = 11.7 Hz, 1H), 4.78 (d, *J* = 11.8 Hz, 1H), 4.59-4.55 (m, 3H), 4.44 (dd, *J* = 12.2, 2.9 Hz, 1H), 4.37 (d, *J* = 12.1 Hz, 1H), 4.12-3.88 (m, 6H), 3.69-3.61 (m, 5H), 3.56 (dd, *J* = 10.1, 7.6 Hz, 1H), 3.51-3.48 (m, 1H), 3.42 (s, 3H), 3.19 (dt, *J* = 9.7, 6.5 Hz, 1H), 3.12 (q, *J* = 6.7 Hz, 2H), 2.71 (dd, *J* = 12.3, 3.5 Hz, 1H), 2.47 (s, 3H), 2.07 (s, 4H), 2.06 (s, 3H), 1.98 (s, 3H), 1.92 (s, 3H), 1.50-1.41 (m, 4H), 1.31-1.25 (m, 2H). ^{13}C NMR (101 MHz, Chloroform-*d*) δ 172.12, 170.99, 170.37, 170.31, 170.10, 168.16, 165.61, 165.54, 165.26, 156.54, 153.80, 139.02, 138.30, 136.82, 133.54, 133.50, 133.31, 129.87, 129.83, 129.53, 129.23, 129.01, 128.72, 128.63, 128.60, 128.39, 128.18, 128.15, 127.99, 127.95, 127.89, 127.57, 100.33, 99.82, 99.50, 78.19, 75.90, 74.88, 74.70, 73.45, 73.38, 71.95, 71.75, 71.59, 70.51, 69.16, 69.03, 66.61, 63.32, 63.21, 59.12, 54.52, 53.08, 41.03, 36.40, 29.58, 28.93, 24.83, 23.47, 23.29, 21.15, 20.94, 20.91. HRMS (ESI): Calcd. for $C_{81}H_{89}N_3NaO_{28}$ [M+Na]$^+$ 1574.5530, found 1574.5520.

Compound 21

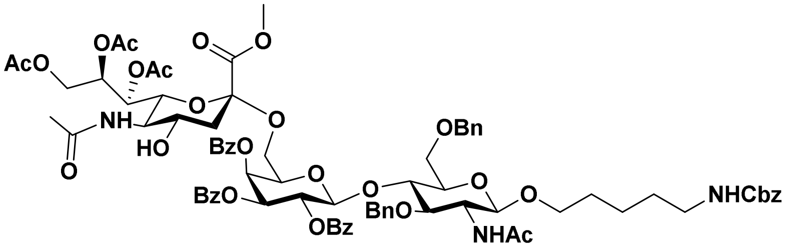

Compound **21** was synthesised from **20** using the general oxazolidinone ring deprotection procedure **D** (81%). ^{1}H NMR (400 MHz, Methanol-d_4) δ 7.95-7.93 (m, 2H), 7.90-7.87 (m, 2H), 7.76-7.73 (m, 2H), 7.62 (m, 1H), 7.55-7.52 (m, 1H), 7.50-7.44 (m, 5H), 7.43-7.23 (m, 17H), 5.93 (d, *J* = 3.3 Hz, 1H), 5.67 (dd, *J* = 10.4, 7.8 Hz, 1H), 5.57 (dd, *J* = 10.4, 3.3 Hz, 1H), 5.51-5.46 (m, 1H), 5.33 (dd, *J* = 8.4, 2.1 Hz, 1H), 5.19-5.13 (m, 2H), 5.06 (s, 2H), 4.78 (d, *J* = 11.3 Hz, 1H), 4.67 (d, *J* = 12.1 Hz, 1H), 4.40-4.36 (m, 3H), 4.16-4.10 (m, 3H), 4.06 (dd, *J* = 10.7, 2.1 Hz, 1H), 3.93 (dd, *J* = 9.9, 5.3 Hz, 1H), 3.87-3.82 (m, 2H), 3.80-3.65 (m, 3H), 3.46-3.36 (m, 7H), 3.09 (t, *J* = 6.9 Hz, 2H), 2.53 (dd, *J* = 12.9, 4.4 Hz, 1H), 2.13 (s, 3H), 2.06 (s, 3H), 2.00 (s, 3H), 1.93 (s, 3H), 1.88 (s, 3H), 1.75 (t, *J* = 12.6 Hz, 1H), 1.55-1.44 (m, 4H), 1.37-1.32 (m, 2H). ^{13}C NMR (101 MHz, Methanol-d_4) δ 173.76, 173.13, 172.42, 171.81, 171.46, 169.29, 166.70, 166.59, 158.80, 140.55, 139.51, 138.44, 134.76, 134.58, 134.46, 130.80, 130.70, 130.66, 130.49, 130.30, 130.29, 129.77, 129.75, 129.72, 129.43, 129.29, 129.18, 128.95, 128.91, 128.73, 128.36, 102.53, 101.25, 100.70, 81.45, 79.44, 77.68, 75.75, 74.85, 74.42, 73.93, 73.38, 72.85, 71.98, 70.38, 69.68, 69.34, 69.05, 68.90, 67.24, 63.78, 63.15, 56.10, 53.02, 52.69, 42.00, 41.72, 30.42, 30.13, 24.22, 23.05, 22.90, 21.22, 21.06, 20.88. HRMS (ESI): Calcd. for $C_{80}H_{92}N_3O_{27}$ [M+H]$^+$ 1526.5918, found 1526.5905.

Compound H6

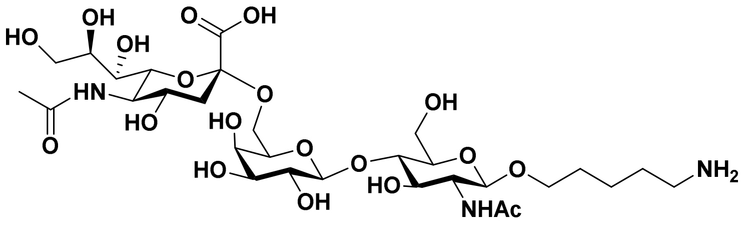

Compound **H6** was synthesised from **21** using the general global deprotection procedure **E** (65%). ^{1}H NMR (400 MHz, Deuterium Oxide) δ 4.56 (d, *J* = 7.9 Hz, 1H), 4.45 (d, *J* = 7.9 Hz, 1H), 4.03-4.00 (m, 2H), 3.95-3.59 (m, 16H), 3.57-3.52 (m, 3H), 3.00 (t, *J* = 7.7 Hz, 2H), 2.67 (dd, *J* = 12.4, 4.7 Hz, 1H), 2.06 (s, 3H), 2.03 (s, 3H), 1.75-1.58 (m, 5H), 1.45-1.36 (m, 2H). ^{13}C NMR (101 MHz, Deuterium Oxide) δ 174.90, 174.42, 173.41, 103.45, 100.91, 100.06, 80.80, 74.46, 73.68, 72.54, 72.42, 71.65, 70.70, 70.02, 68.39, 68.34, 68.17, 63.35, 62.65, 60.36, 54.86, 51.84, 40.03, 39.32, 28.03, 26.33, 22.26, 22.10, 22.01. HRMS (ESI): Calcd. for $C_{30}H_{54}N_3O_{19}$ $[M+H]^+$ 760.3352, found 760.3351.

2.1.7 Synthesis of Ac-2,3 trisaccharide H3.

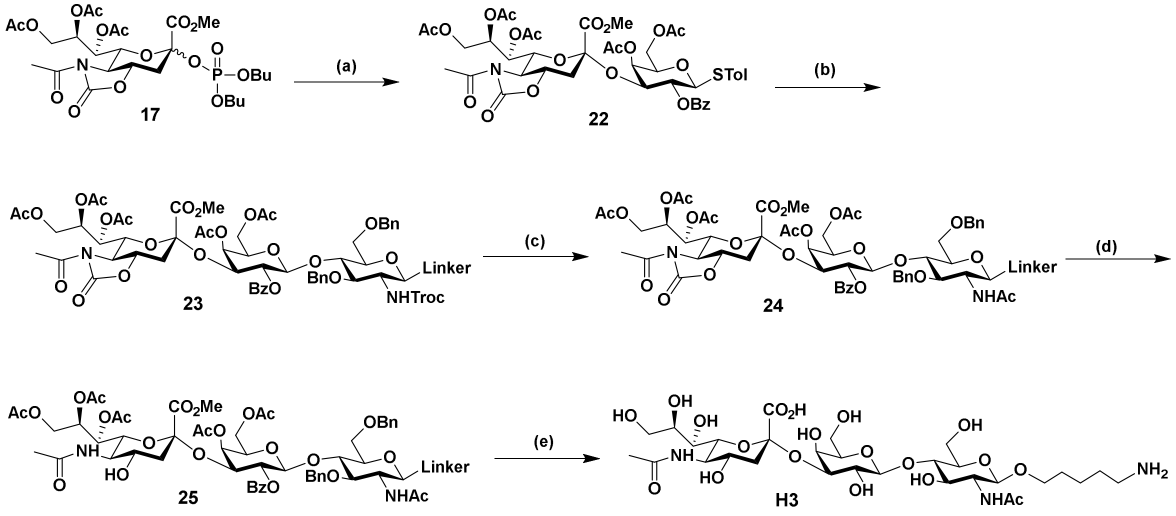

Scheme S7. Synthesis 2,3 trisaccharide **H3**. Reagents and conditions: (a) i) **7**, TMSOTf, CH_2Cl_2, -50 °C, 2 h; ii) Ac_2O, pyridine, rt, 12 h, 54%; (b) **8**, NIS, TfOH,CH_2Cl_2, -20 °C, 2 h, 73%; (c) Zn, THF/AcOH/Ac_2O (3:2:1, v/v), rt, 4 h, 77%; (d) 1,2 ethanedithiol, DBU, CH_2Cl_2, 0 °C, 2 h, 79%; (e) i) LiOH, THF/H_2O/MeOH (2:2:1, v/v), rt, 12 h; ii) $Pd(OH)_2$/C, H_2, H_2O/MeOH (3:1, v/v), 48 h, 61% over two steps.

Compound 22

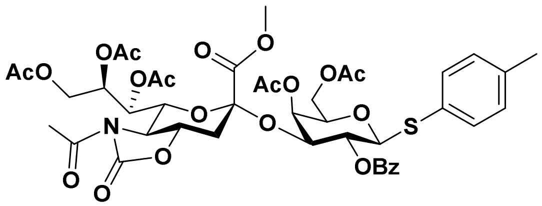

Compound **22** was synthesised from donor **17** and acceptor **7** using the general glycosylation procedure **A**. Next, the compound was dissolved in pyridine (10 mL), and acetic anhydride was added (0.5 mL) at 0 °C. The resulting mixture was stirred at RT for 12 hours, concentrated *in vacuo* and purified using mixture of (3:2, v/v) ethyl acetate and hexane as eluent to afford compound **22** (54% over two steps). ^{1}H NMR (400 MHz, Chloroform-*d*) δ 8.21-8.18 (m, 2H), 7.61-7.56 (m, 1H), 7.50-7.46 (m, 2H), 7.35-7.33 (m, 2H),

7.07-7.05 (m, 2H), 5.62 (ddd, *J* = 9.6, 7.3, 2.5 Hz, 1H), 5.45 (dd, *J* = 9.3, 2.1 Hz, 1H), 5.30-5.28 (m, 1H), 4.97-4.94 (m, 2H), 4.75 (dd, *J* = 9.7, 3.2 Hz, 1H), 4.42 (dd, *J* = 12.3, 2.6 Hz, 1H), 4.21 (dd, *J* = 9.4, 2.2 Hz, 1H), 4.08-4.06 (m, 2H), 3.99-3.91 (m, 2H), 3.81 (s, 4H), 3.44 (dd, *J* = 11.4, 9.4 Hz, 1H), 2.88 (dd, *J* = 11.8, 3.4 Hz, 1H), 2.39 (s, 3H), 2.31 (s, 3H), 2.16 (s, 3H), 2.10 (s, 3H), 2.05 (s, 3H), 1.98 (s, 3H), 1.87-1.83 (m, 1H), 1.24 (s, 3H). ^{13}C NMR (101 MHz, Chloroform-*d*) δ 171.62, 170.91, 170.84, 170.44, 170.39, 170.15, 167.79, 165.63, 153.55, 138.30, 133.59, 133.51, 130.57, 130.32, 129.56, 128.67, 97.37, 86.88, 75.75, 74.49, 74.40, 72.61, 71.48, 69.43, 68.26, 68.08, 63.56, 62.36, 58.92, 53.56, 36.07, 24.65, 21.62, 21.27, 20.84, 20.07. HRMS (ESI): Calcd. for $C_{43}H_{50}NO_{20}$ $[M+H]^+$ 932.2647, found 932.2656.

Compound 23

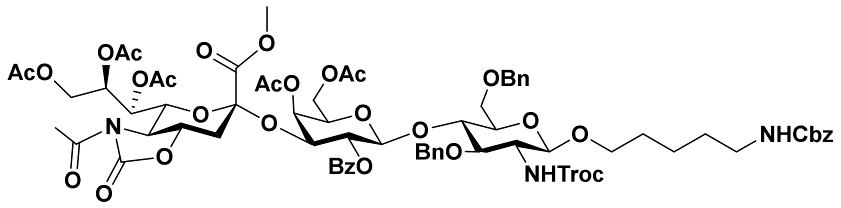

Compound 23 was synthesised from donor 22 and acceptor 8 using the general glycosylation procedure B (73%). ^{1}H NMR (400 MHz, Chloroform-*d*) δ 8.25-8.23 (m, 2H), 7.58-7.54 (m, 1H), 7.48-7.44 (m, 2H), 7.35-7.22 (m, 15H), 5.71 (ddd, *J* = 9.8, 7.4, 2.6 Hz, 1H), 5.49 (dd, *J* = 9.4, 2.2 Hz, 1H), 5.34 (dd, *J* = 10.1, 7.8 Hz, 2H), 5.07-5.03 (m, 3H), 4.92-4.89 (m, 2H), 4.84-4.77 (m, 1H), 4.72-4.69 (m, 2H), 4.66 (m, 2H), 4.48 (dd, *J* = 12.3, 2.6 Hz, 1H), 4.41 (s, 3H), 4.20 (dd, *J* = 9.4, 2.2 Hz, 1H), 4.08-3.96 (m, 3H), 3.87-3.77 (m, 7H), 3.70-3.64 (m, 2H), 3.55 (dd, *J* = 10.7, 5.5 Hz, 1H), 3.47-3.39 (m, 3H), 3.20-3.18 (m, 1H), 3.12 (q, *J* = 6.8 Hz, 2H), 2.87 (dd, *J* = 11.7, 3.5 Hz, 1H), 2.40 (s, 3H), 2.17 (s, 3H), 2.06 (s, 3H), 1.97 (s, 3H), 1.91 (s, 3H), 1.50-1.40 (m, 4H), 1.31-1.24 (m, 5H). ^{13}C NMR (101 MHz, Chloroform-*d*) δ 171.68, 171.03, 170.80, 170.32, 170.14, 167.67, 165.64, 153.56, 138.59, 136.78, 133.70, 130.55, 130.00, 128.81, 128.65, 128.35, 128.33, 128.20, 127.89, 127.62, 127.51, 127.37, 99.97, 97.59, 75.77, 74.82, 74.39, 73.03, 71.99, 71.63, 71.53, 70.86, 69.43, 67.94, 67.88, 66.69, 63.65, 61.87, 58.94, 53.58, 35.98, 29.70, 29.57, 28.98, 24.67, 23.21, 21.61, 20.86, 20.79, 20.70, 20.26.HRMS (ESI): Calcd. for $C_{72}H_{85}Cl_3N_3O_{29}$ $[M+H]^+$ 1560.4334, found 1560.4331.

Compound 24

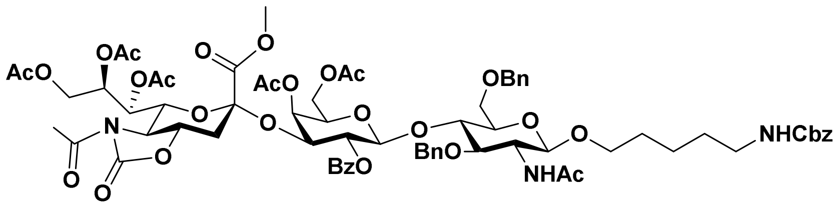

Compound **24** was synthesised from **23** using the general Troc deprotection procedure **C** (77%). ^{1}H NMR (400 MHz, Chloroform-*d*) δ 8.22-8.20 (m, 2H), 7.55 (t, *J* = 7.4 Hz, 1H), 7.45 (t, *J* = 7.6 Hz, 2H), 7.32-7.22 (m, 15H), 5.99 (d, *J* = 9.5 Hz, 1H), 5.70 (ddd, *J* = 9.9, 7.6, 2.6 Hz, 1H), 5.48 (dd, *J* = 9.4, 2.2 Hz, 1H), 5.33 (dd, *J* = 10.2, 7.9 Hz, 1H), 5.05 (s, 2H), 5.01 (s, 1H), 4.96-4.93 (m, 2H), 4.75-4.71 (m, 2H), 4.64 (d, *J* = 11.7 Hz, 1H), 4.50-4.38 (m, 3H), 4.31 (d, *J* = 6.1 Hz, 1H), 4.20 (dd, *J* = 9.4, 2.2 Hz, 1H), 4.10-4.04 (m, 1H), 4.01-3.95 (m, 1H), 3.87-3.76 (m, 7H), 3.72-3.67 (m, 2H), 3.62-3.53 (m, 3H), 3.46 (dd, *J* = 11.4, 9.4 Hz, 1H), 3.08 (q, *J* = 6.8 Hz, 2H), 2.97 (dt, *J* = 9.7, 6.4 Hz, 1H), 2.86 (dd, *J* = 11.8, 3.4 Hz, 1H), 2.38 (s, 3H), 2.16 (s, 3H), 2.08 (s, 3H), 1.95 (s, 3H), 1.91 (s, 3H), 1.89 (s, 3H), 1.41-1.36 (m, 4H), 1.30 (s, 3H), 1.25-1.20 (m, 2H). ^{13}C NMR (101 MHz, Chloroform-*d*) δ 171.59, 170.90, 170.70, 170.20, 170.17, 170.13, 170.04, 167.54, 166.07, 156.48, 153.42, 138.63, 138.44, 136.75, 133.64, 130.38, 129.86, 128.72, 128.49, 128.22, 128.03, 127.60, 127.44, 127.25, 100.21, 99.30, 97.43, 78.08, 75.55, 72.90, 72.68, 71.62, 71.47, 71.24, 70.79, 69.68, 68.98, 67.88, 67.78, 66.45, 63.60, 61.70, 58.78, 53.49, 53.27, 40.92, 35.88, 29.44, 28.80, 24.54, 23.26, 23.20, 21.50, 20.70, 20.67, 20.56, 20.19. HRMS (ESI): Calcd. for $C_{71}H_{86}N_3O_{28}$ $[M+H]^+$ 1428.5398, found 1428.5391.

Compound 25

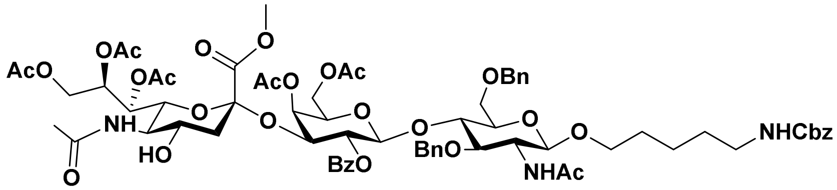

Compound **25** was synthesised from **24** using the general oxazolidinone ring deprotection procedure **D** (79%). ^{1}H NMR (400 MHz, Methanol-d_4) δ 8.28-8.25 (m, 2H), 7.70-7.65 (m, 1H), 7.59 (dd, *J* = 8.3, 6.9 Hz, 2H), 7.44-7.25 (m, 15H), 5.76 (ddd, *J* = 9.4, 6.4, 2.6 Hz, 1H), 5.30 (dd, *J* = 10.1, 7.9 Hz, 1H), 5.21 (dd, *J* = 9.9, 2.7 Hz, 1H), 5.12 (d, *J* = 8.0 Hz, 1H), 5.08-5.05 (m, 3H), 4.86-4.83 (m, 1H), 4.65-4.62 (m, 2H), 4.53 (d, *J* = 11.6 Hz, 1H), 4.36-4.32 (m, 2H), 4.10-4.02 (m, 2H), 3.99-3.95 (m, 2H), 3.84 (s, 4H), 3.81-3.73 (m, 4H), 3.65 (dd, *J* = 10.6, 2.7 Hz, 1H), 3.60-3.54 (m, 2H), 3.46-3.36 (m, 2H), 3.31-3.28 (m, 1H), 3.09 (t, *J* = 6.9 Hz, 2H), 2.53 (dd, *J* = 12.7, 4.4 Hz, 1H), 2.19 (s, 3H), 2.09 (s, 3H), 1.98 (s, 3H), 1.95 (s, 3H), 1.88 (s, 3H), 1.83 (s, 3H), 1.53-1.44 (m, 7H), 1.38-1.32 (m, 2H). ^{13}C NMR (101 MHz, Methanol-d_4) δ 173.64, 173.14, 172.37, 172.12, 171.96, 171.77, 171.56, 169.73, 166.89, 158.82, 140.34, 139.73, 138.46, 134.76, 131.32, 131.27, 129.97, 129.43, 129.33, 129.16, 128.91, 128.83, 128.73, 128.68, 128.58, 128.41, 102.44, 101.26, 98.48, 81.70, 79.46, 76.97, 75.78, 75.17, 74.10, 73.26, 72.27, 71.99, 70.36, 69.95, 69.55, 69.29, 68.48, 68.39, 67.24, 63.91, 62.64, 56.15, 53.53, 52.16, 41.71, 41.43, 30.41, 30.12, 24.21, 23.01, 22.87, 21.69, 20.92, 20.81, 20.64. HRMS (ESI): Calcd. for $C_{70}H_{88}N_3O_{27}$ $[M+H]^+$ 1402.5605, found 1402.5614.

Compound H3

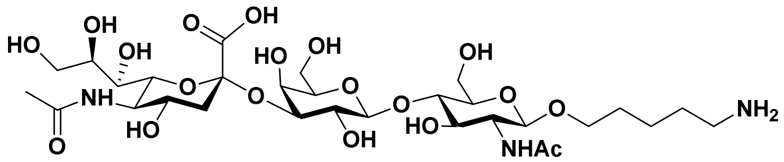

Compound **H3** was synthesised from **25** using the general global deprotection procedure **E** (61%). ^{1}H NMR (400 MHz, Deuterium Oxide) δ 4.56 (d, *J* = 7.9 Hz, 1H), 4.52 (d, *J* = 7.7 Hz, 1H), 4.12 (dd, *J* = 9.9, 3.1 Hz, 1H), 4.01 (dd, *J* = 12.3, 2.3 Hz, 1H), 3.96 (d, *J* = 3.1 Hz, 1H), 3.92-3.88 (m, 5H), 3.76-3.74 (m, 7H), 3.67-3.64 (m, 2H), 3.63-3.57 (m, 4H), 3.01-2.98 (m, 2H), 2.76 (dd, *J* = 12.4, 4.7 Hz, 1H), 2.04 (s, 6H), 1.80 (t, *J* = 12.1 Hz, 1H), 1.72-1.66 (m, 2H), 1.62-1.57 (m, 2H), 1.44-1.38 (m, 2H). ^{13}C NMR (101 MHz, Deuterium Oxide) δ 175.02, 174.43, 173.90, 102.56, 101.16, 99.81, 78.26, 75.48, 75.20, 74.77, 72.89, 72.39, 71.78, 70.08, 69.39, 68.36, 68.08, 67.46, 62.58, 61.05, 60.02, 55.08, 51.67, 39.62, 39.32, 28.07, 26.36, 22.14, 22.11, 22.03. HRMS (ESI): Calcd. for $C_{30}H_{54}N_3O_{19}$ $[M+H]^+$ 760.3352, found 760.3347.

2.2 Surface modifications and characterizations

Preparation of modified Glassy Carbon plates. GCPs polish was followed with 5 minutes sonication in TDW and drying under nitrogen stream. The trisaccharides electrografted on GCP by applying cyclic voltammetry (CV) in the range of 0.6 – 1.2 V (vs. Ag/AgCl 3M KCl reference electrode) at a scan rate of 10 mV/s for 5 cycles. Exposure to enzyme performed by same method as described for GCE. Substrates were Rinsed with TDW and dried with nitrogen before measurements.

Preparation of modified Au Surfaces. Modified Au surfaces with LPA were prepared by previously reported protocol. The saccharide was coupled to the surface by same protocol used for modification of AuE. Exposure to enzyme performed by same method as described for GCE. Substrates were Rinsed with TDW and dried with nitrogen before measurements.

Characterization of modified glassy carbon plates. Surface characterizations of modified GCP were performed by XPS, CA and CPD analysis. CA measurements were performed with TDW using Attension Theta Lite goniometer (Biolin Scientific). CPD measurements were performed with Kelvin probe S, operated by Kelvin control 07 unit (DeltaPhi Besocke, Julich, Germany), with vibrating gold grid reference electrode in a home-built faraday cage under argon atmosphere, and the signal was recorded with Keithley 2450 source meter unit. XPS measurements were performed using Axis Supra+ spectrometer (Kratos Analytical Ltd., Manchester, U.K.) with Al Kα monochromatic x-ray source (1486.7 eV). The XPS spectra were acquired with a takeoff angle of 90° (normal to analyzer); vacuum condition in the chamber was 1.9 nTorr. High-resolution XPS spectra were acquired with a pass energy of 20 eV and step size of 0.1 eV. The binding energies were calibrated according to the C 1s peak position (285.0 eV). Data were collected and analyzed by using Casa XPS (Casa Software Ltd.) and Vision data processing program (Kratos Analytical Ltd.).

Characterization of modified Au surface. Surface characterizations of modified Au surfaces were performed by the same methods as described for GCP. Additionally, variable angle spectroscopic ellipsometry (VASE) analyses were performed using VB-400 ellipsometer (Woollam Co.) at brewster angle of 75° scanning from 300 to 900 nm wavelength and fitting with Cauchy model on gold surface.

Surface coverage calculations on Au.

Surface coverage of LPA on Au were calculated according to the fully stretched molecular length and diameter. According to VASE analyses the LPA layer optical-thickness was 5 Å when the length of the molecule is 10.3 Å. This indicates that the number of molecules corresponds to 48.5 % of possible coverage. Considering that the diameter of the molecule is 6.25 Å from LPA size calculated by MM2 forcefield in chem3d (Figure S140), the number of molecules on the surface was calculated by the following equation $C = \pi r(\frac{D}{2})^2 * 48.5\%$. Resulting in coverage of $1.6*10^{14}$ molecules/cm^2. Footprint is 1/C resulting in 63 Å^2. Surface coverage of sialoside-LPA on Au and was determined by VASE the coverage is $C = \frac{L_{Cal}}{L_{Measured}} = 24\%$, where the calculated length is 33 Å (calculated by MM2 forcefield in chem3d (Figure S141) and measured addition is 8 Å, with $7.9*10^{13}$ molecules/cm^2. Footprint is 1/C resulting in 133 Å^2.

Sialoside coverage was determined using XPS analysis and was calculated based on the ratio between the peak area. For LPA determination S2p/Au4f ratio resulted with 0.28. This value was compared with the previous LPA monolayer study to clarify the covarege.[4] Corresponds to the N1s of the sialoside and the peak area related to the S2p of LPA (Figure S11 and S30) considering a relation of 3 nitrogen atoms to 2 sulfur atoms. The relation translates to 0.46 sialosides on top of the LPA layer indicating that the coverage is 7.3×10^{13} molecules/cm^2. After NA activity the concentration was calculated by the same method (3 nitrogen to 2 sulfur atoms and 2 nitrogen to 2 sulfur for non-sialylated glycan which is 0.58) by using the equation 0.46(1.5x+(1-x)) = 0.58 which will give us that 52% of the sialic acid was removed.

3. Figures

3.1 Electrografting with the sialosides

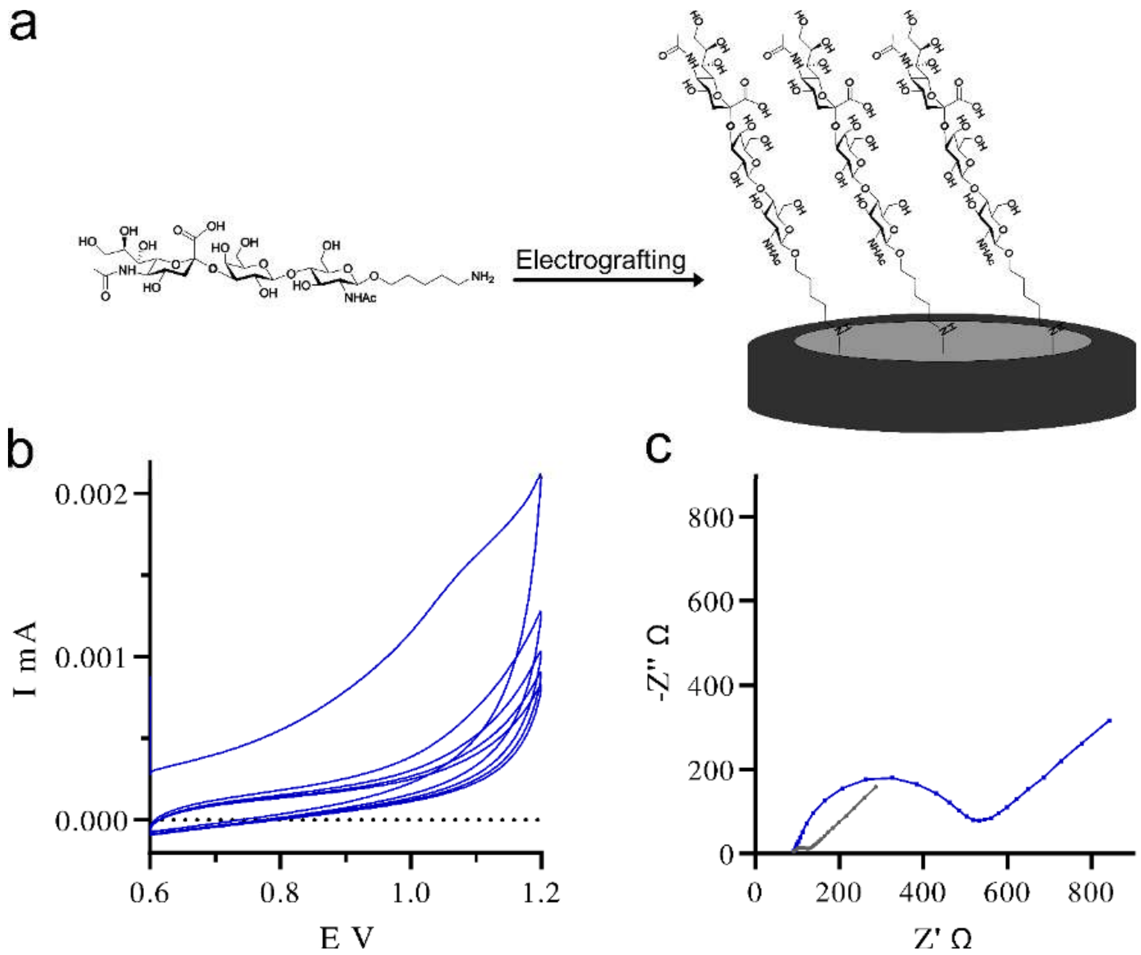

Figure S1: a) Electrografting of **H3** on GCE. **b)** CV scanning from 0.6 V to 1.2 V at a rate of 10mV/S for 5 cycles. **c)** EIS analyses before (black) and after (blue) deposition.

3.2 Modification of Au Electrode

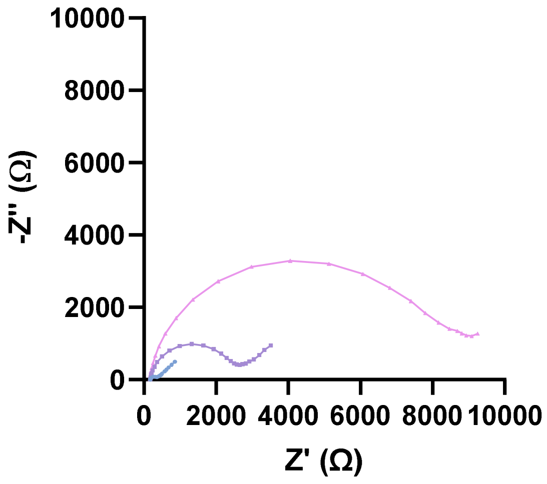

Figure S2: Nyquist plot of Impedimetric response for clean gold electrode (blue), LPA modified electrode (Purple) and AuE-H3 (Pink).

3.3 3D structures of the molecules for surface characterizations

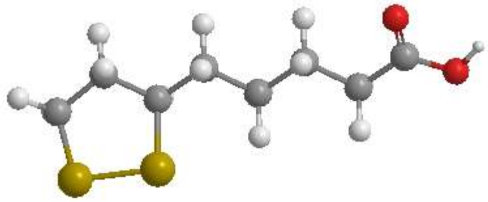

Figure S3: LPA structure optimized by MM2 force field for molecule size calculations.

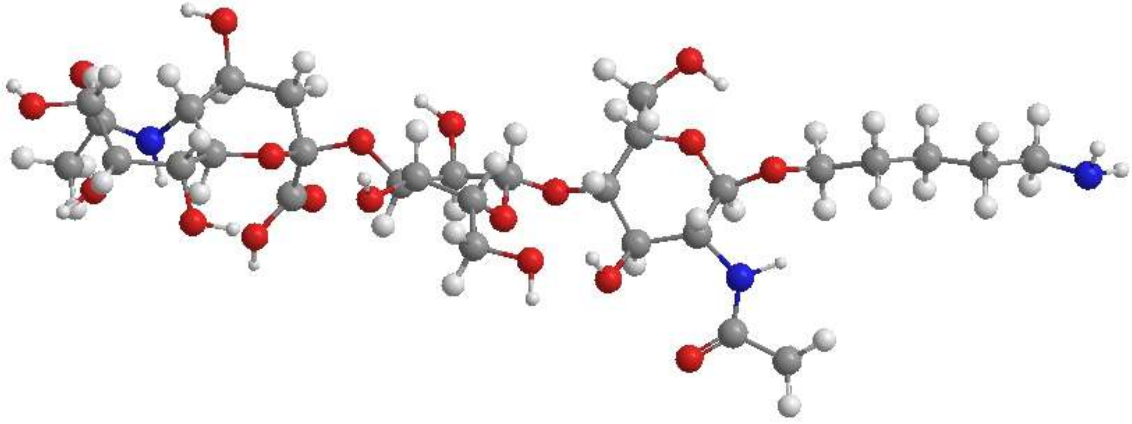

Figure S4: LPA structure optimized by MM2 force field for molecule size calculations.

<u>3.4 Nyquist plot of impedimetric response for **GCE-H3** and **GCE-H6** prior and after exposure to *3NACP* and *6NAAU*</u>

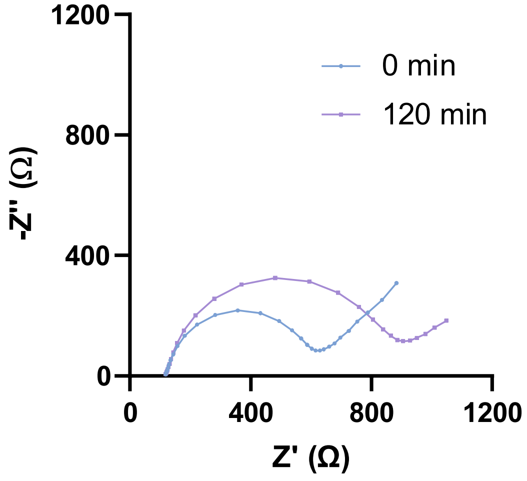

Figure S5: Nyquist plot of Impedimetric response of **GCE-H3** before (Blue) and after (Purple) exposure to *6NAAU*.

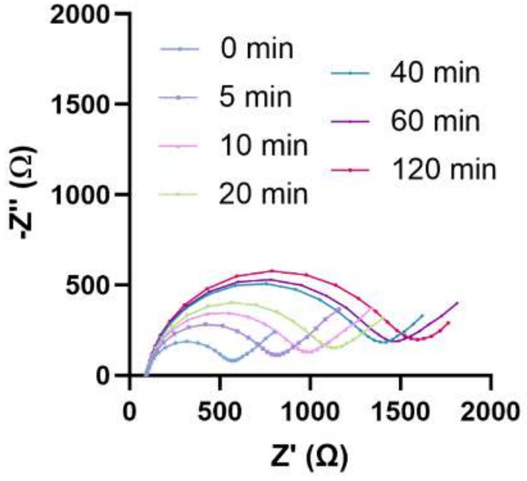

Figure S6: Nyquist plot of Impedimetric response of **GCE-H6** before and after exposure to *6NAAU* for different durations.

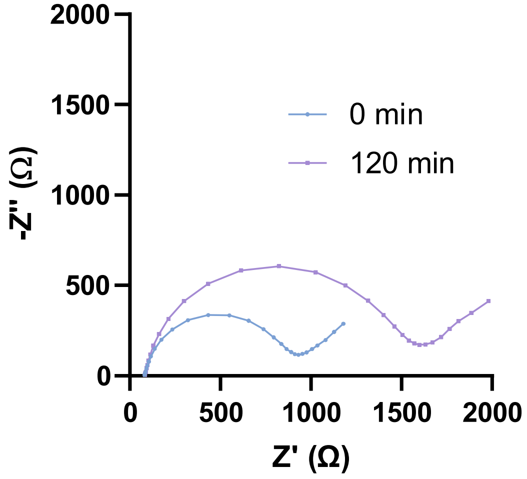

Figure S7: Nyquist plot of Impedimetric response of **GCE-H6** before (Blue) and after (Purple) exposure to *3NACP*.

3.5 Response of Control GCE-Propyl amine to *3NACP* without glycan

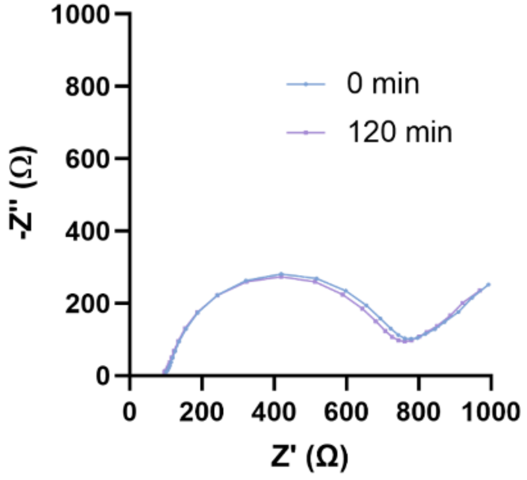

Figure S8: Nyquist plot of Impedimetric response of GCE with propyl amine before (Blue) and after (Purple) exposure to *3NACP*.

3.6 Nyquist plot of impedimetric response for AuE-H3 and AuE-H6 prior and after exposure to *3NACP* and *6NAAU*

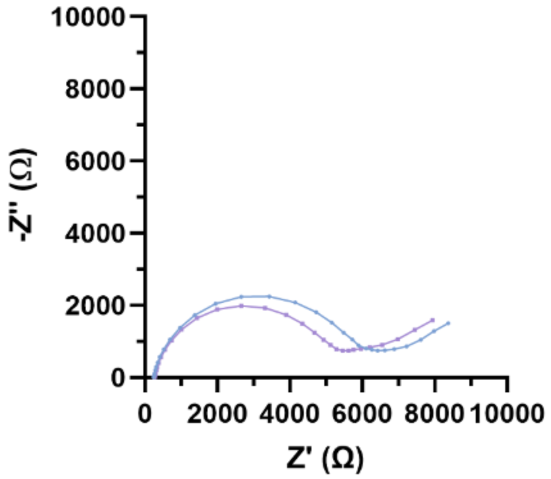

Figure S9: Nyquist plot of Impedimetric response of AuE-H3 before (Blue) and after (Purple) exposure to *6NAAU*.

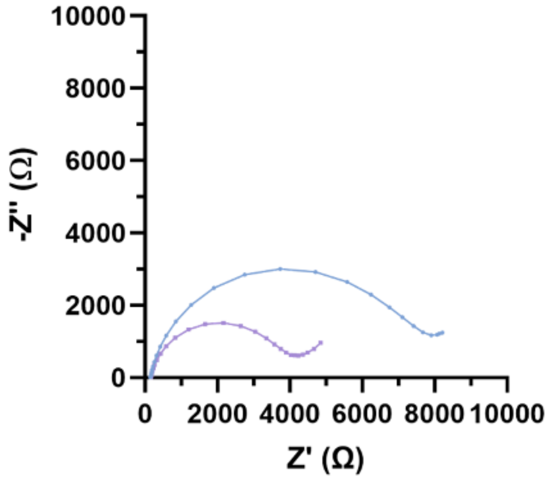

Figure S10: Nyquist plot of Impedimetric response of AuE-H6 before (Blue) and after (Purple) exposure to *6NAAU*.

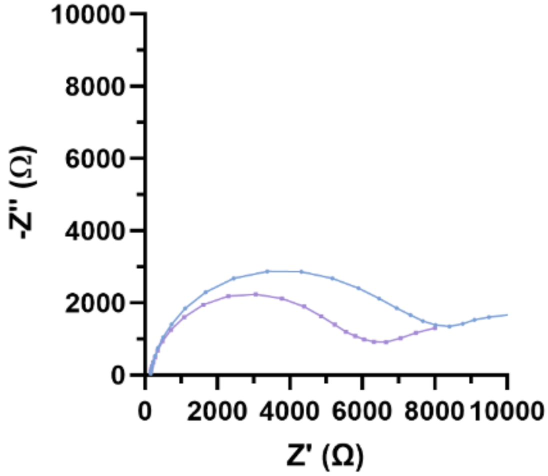

Figure S11: Nyquist plot of Impedimetric response of AuE-H6 before (Blue) and after (Purple) exposure to *3NACP*.

3.7 XPS analyses of GCP-H3 before and after exposure to *3NACP*

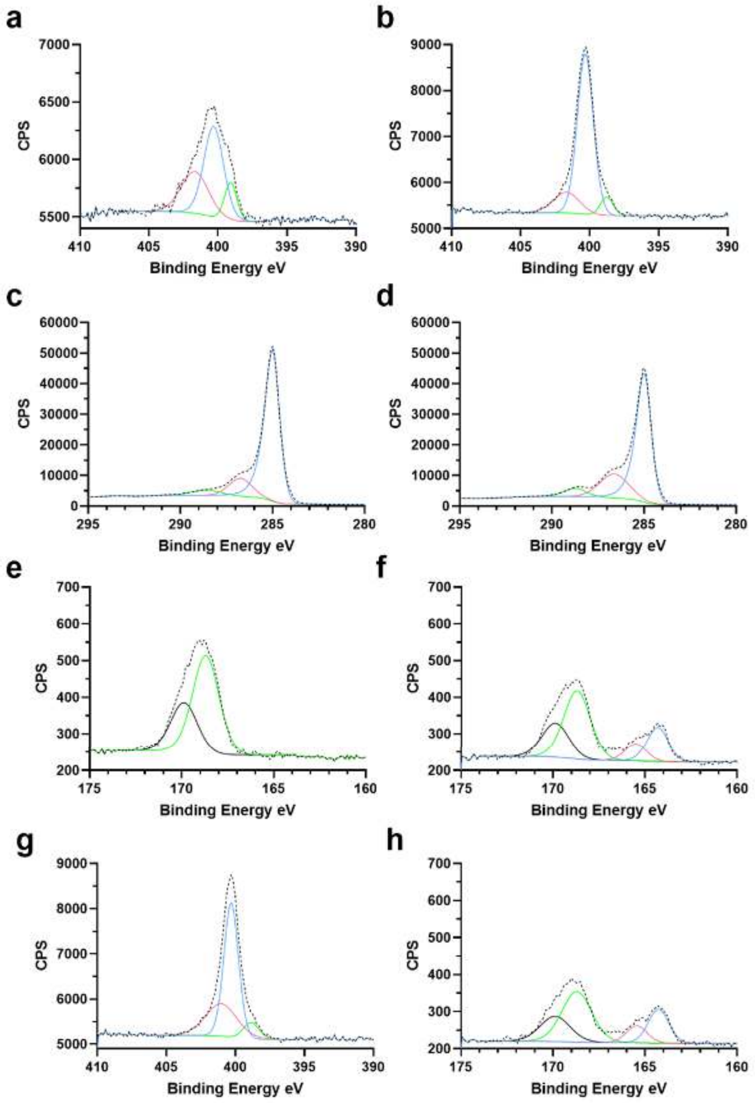

Figure S12: XPS analyses of N1s before (a) and after (b) exposure of GCE-H3 to *3NACP* where Blue is amide, red is ammonium and green is C-N=C. XPS analyses of C1s before (c) and after (d) exposure of GCE-H3 to *3NACP* where C-H is in blue, C-O is in red, and C=O is in green. XPS analyses of S2p before (e) and after (f) exposure of GCE-H3 to *3NACP* where Thiols and disulfide are in blue and red and sulfur impurities from GCE in green and black. (g) and (h) correspond to exposure of GCE-H3 to *3NACP* in presence of oseltamivir. In all cases dashed line is the raw XPS data.

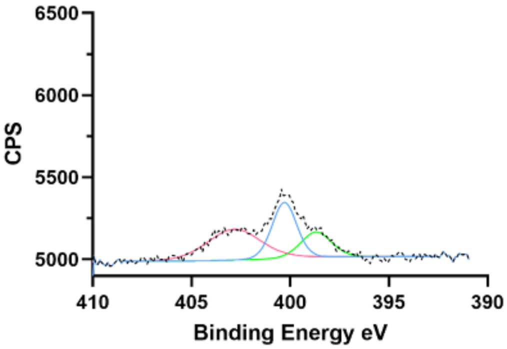

Figure S13: XPS analyses of N1s before electro-grafting where Blue is amide, red is ammonium and green is C-N=C. Dashed line is the raw XPS data.

3.8 XPS analyses of Au-H3 before and after exposure to *3NACP*

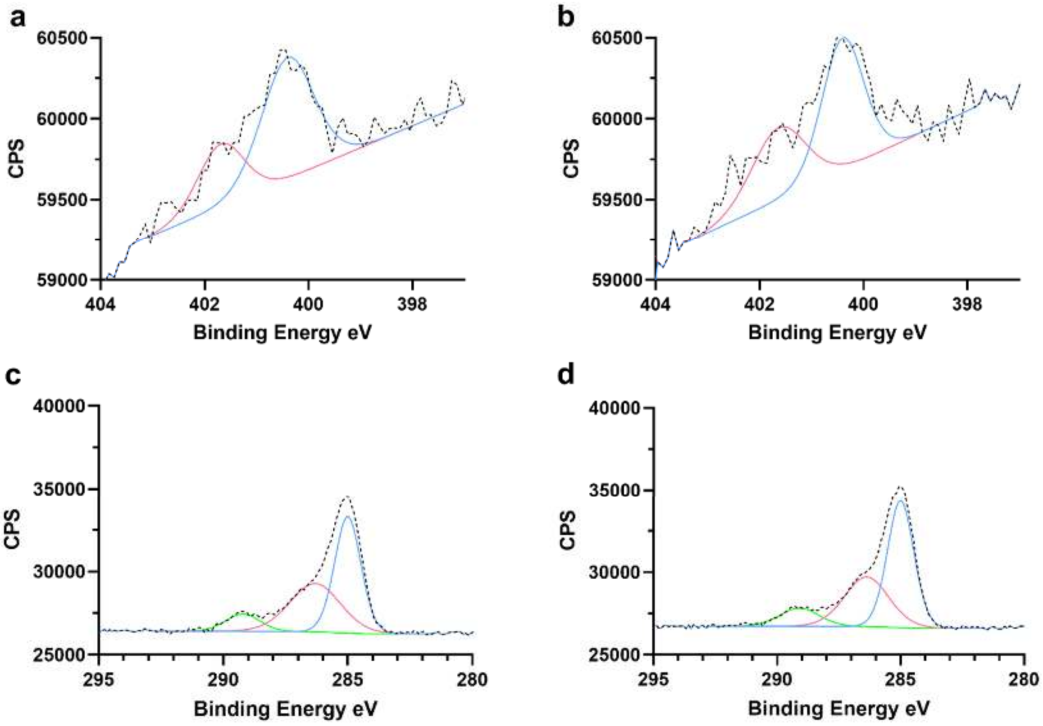

Figure S14: XPS analyses of N1s before (a) with 58% amide relative to nitrogen content and after exposure of Au-H3 to *3NACP* (b) with 54% amide relative to nitrogen content. Blue is amide, red is ammonium. XPS analyses of C1s before (c) with 9% carbonyl signal related to carbon content and after exposure of Au-H3 to *3NACP* (d) with 10% carbonyl signal related to carbon content. For (c) and (d) deconvolution related to C-H is in blue, C-O is in red, and C=O is in green. Dashed line is the raw XPS data.

<u>3.9 Enzyme concentration dependent response of **GCE-H3** to *3NACP*</u>

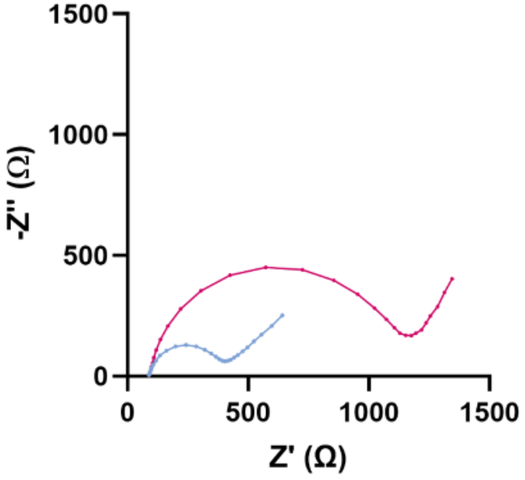

Figure S15: Nyquist plot of Impedimetric response of **GCE-H3** before (Blue) and after (Red) exposure to 0.3 mU/mL *3NACP*.

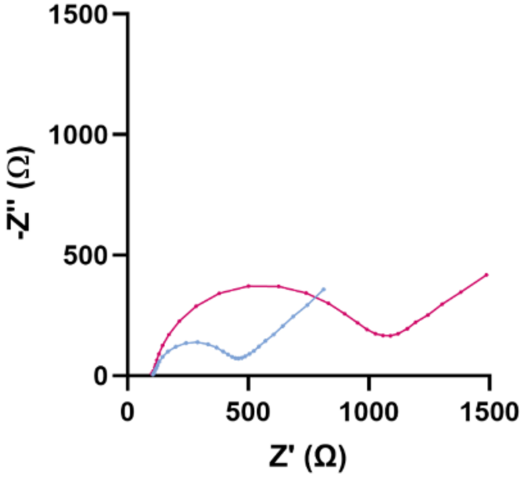

Figure S16: Nyquist plot of Impedimetric response of **GCE-H3** before (Blue) and after (Red) exposure to 0.03 mU/mL *3NACP*.

3.10 Enzyme concentration dependent response of AuE-H3 to *3NACP*

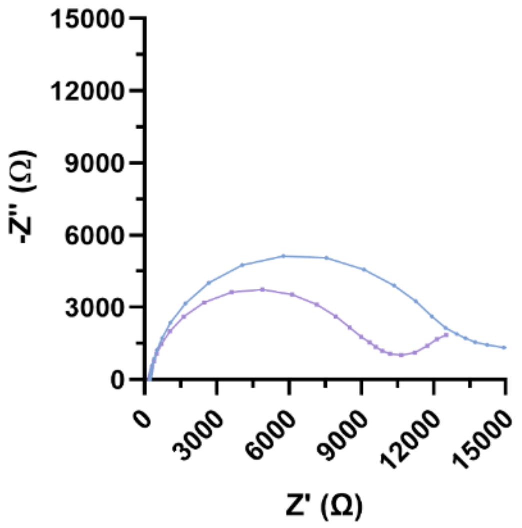

Figure S17: Nyquist plot of Impedimetric response of AuE-H3 before (Blue) and after (Purple) exposure to 0.3 mU/mL *3NACP*.

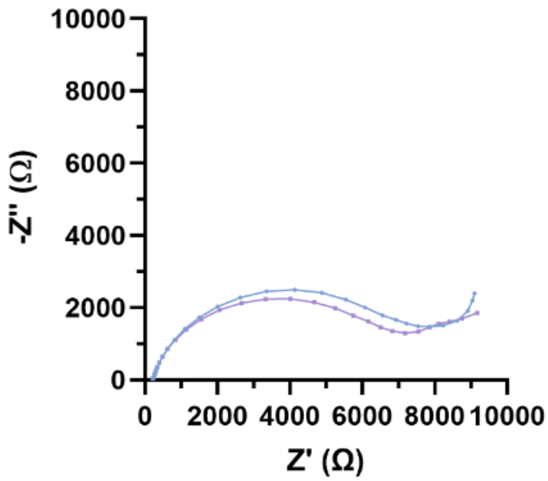

Figure S18: Nyquist plot of Impedimetric response of AuE-H3 before (Blue) and after (Purple) exposure to 0.03 mU/mL *3NACP*.

3.11 Enzyme concentration dependent response summary

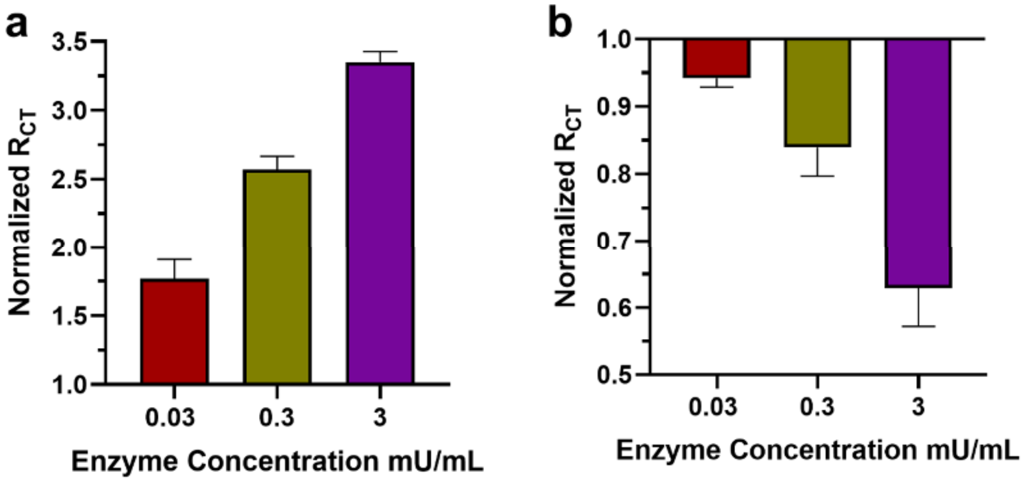

Figure S19: a) Normalized R_{CT} of concentration dependent binding-response to the *3NACP* with GCE-H3. b) Normalized R_{CT} of concentration dependent activity-response to the *3NACP* with AuE-H3.

3.12 Nyquist plot of impedimetric response for **GCE-M3** and **GCE-M6** prior and after exposure to *3NACP* and *6NAAU*

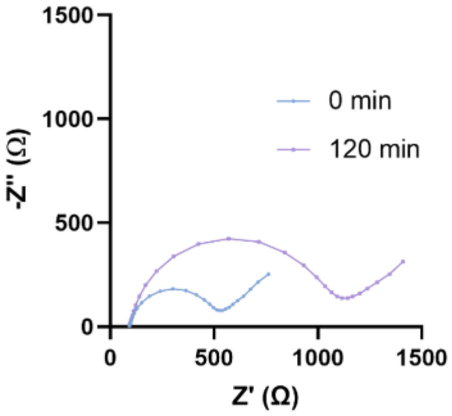

Figure S20: Nyquist plot of Impedimetric response of **GCE-M6** before (Blue) and after (Purple) exposure to *6NAAU*.

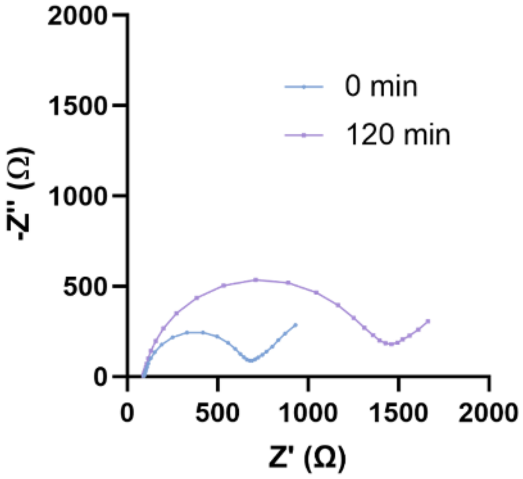

Figure S21: Nyquist plot of Impedimetric response of **GCE-M6** before (Blue) and after (Purple) exposure to *3NACP*.

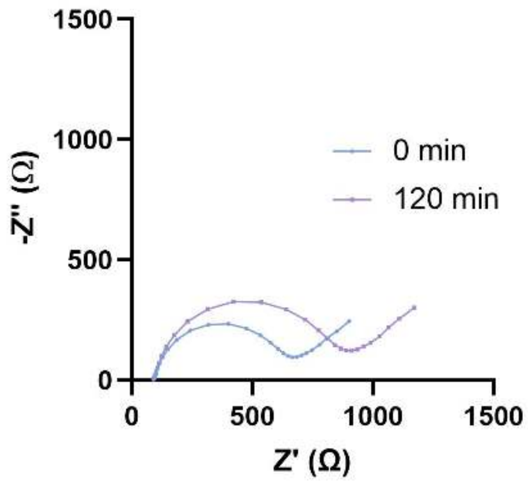

Figure S22: Nyquist plot of Impedimetric response of **GCE-M3** before (Blue) and after (Purple) exposure to *6NAAU*.

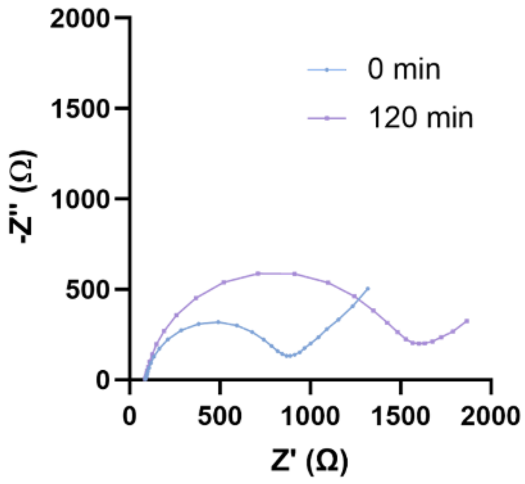

Figure S23: Nyquist plot of Impedimetric response of **GCE-M3** before (Blue) and after (Purple) exposure to *3NACP*.

<u>3.13 Summary of the response of exposure of GCE-M3 and GCE-M6 to *3NACP* and *6NAAU*</u>

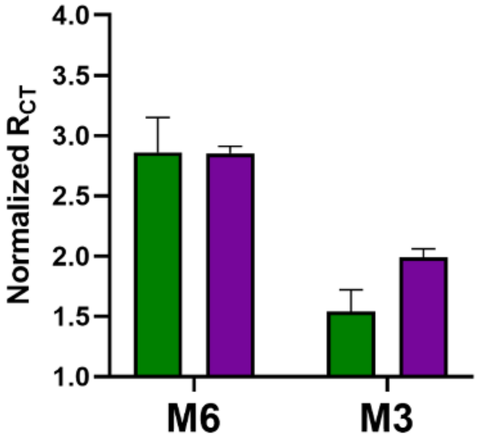

Figure S24: Normalized R_{CT} for the response of GCE-M6 and GCE-M3 after exposure to 3 mU/mL *6NAAU* (Green) or *3NACP* (Purple). Errors are the standard deviation of 5 electrodes.

3.14 Nyquist plot of impedimetric response for AuE-M3 and AuE-M6 prior and after exposure to *3NACP* and *6NAAU*

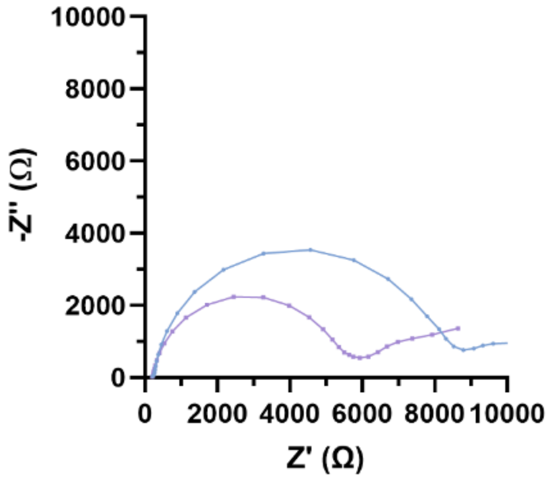

Figure S25: Nyquist plot of Impedimetric response of **AuE-M6** before (Blue) and after (Purple) exposure to *6NAAU*.

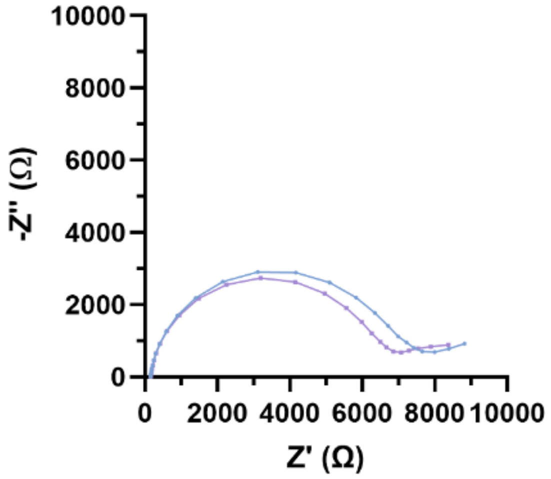

Figure S26: Nyquist plot of Impedimetric response of **AuE-M6** before (Blue) and after (Purple) exposure to *3NACP*.

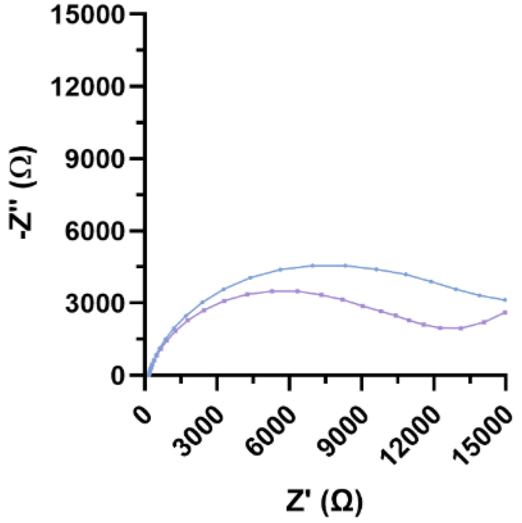

Figure S27: Nyquist plot of Impedimetric response of **AuE-M3** before (Blue) and after (Purple) exposure to *6NAAU*.

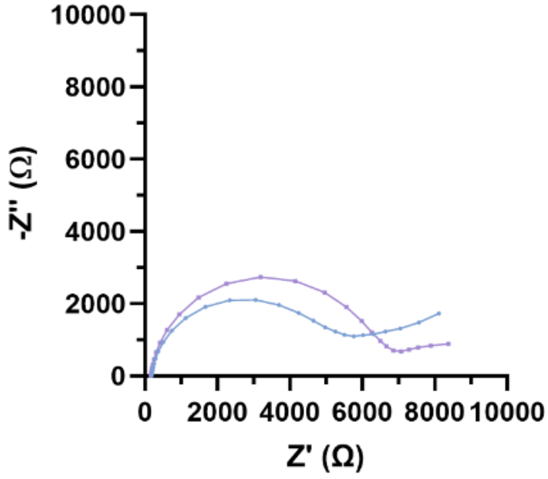

Figure S28: Nyquist plot of Impedimetric response of **AuE-M3** before (Blue) and after (Purple) exposure to *3NACP*.

3.15 Summary of the response of exposure of AuE-M3 and AuE-M6 to *3NACP* and *6NAAU*

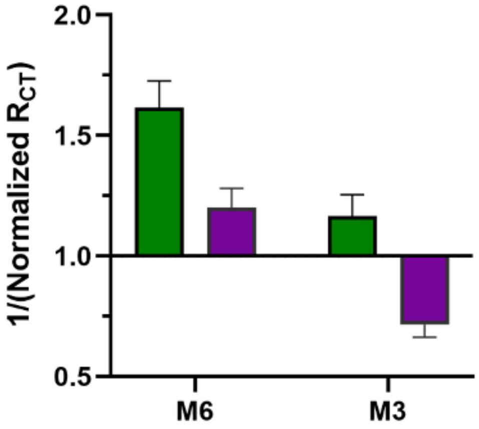

Figure S29: Normalized R_{CT} for the response of AuE-M6 and AuE-M3 after exposure to 3 mU/mL *6NAAU* (Green) or *3NACP* (Purple). Errors are the standard deviation of 5 electrodes.

<u>3.16 XPS analyses of **Au-M3** before and after exposure to *3NACP*</u>

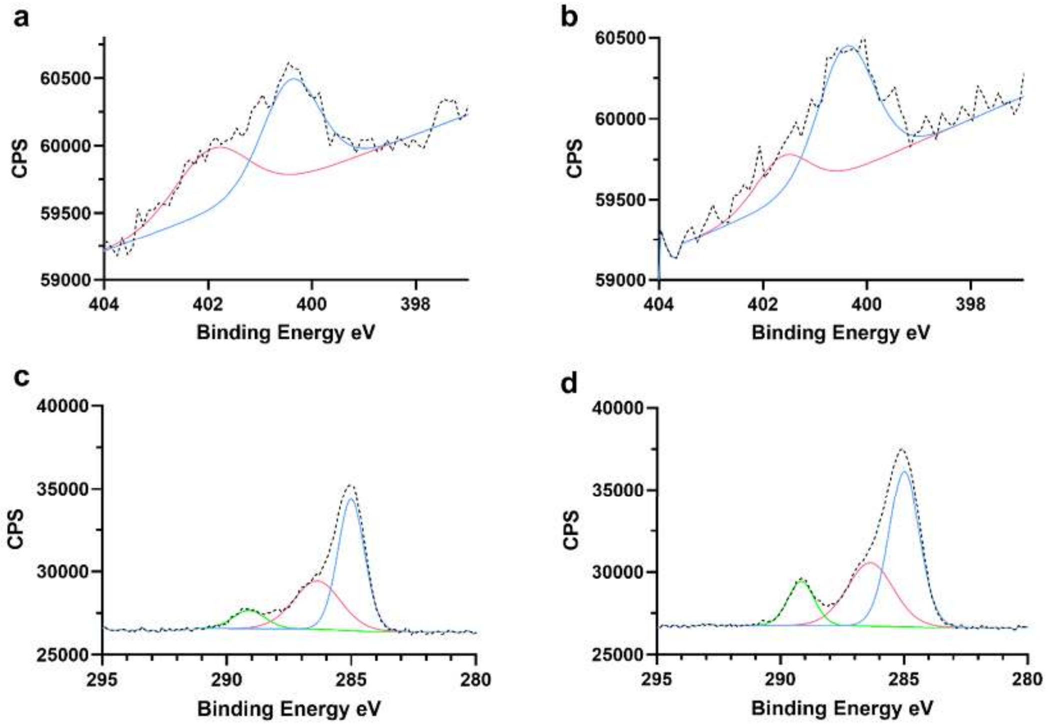

Figure S30: XPS analyses of N1s before (**a**) with 58% amide relative to nitrogen content and after exposure of **Au-M3** to *3NACP* (**b**) with 68% amide relative to nitrogen content. Amide in blue and amine in Red. XPS analyses of C1s before (**c**) with 9% carbonyl signal related to carbon content and after exposure of **Au-M3** to *3NACP* (**d**) with 13% carbonyl signal related to carbon content. For (c) and (d) deconvolution related to C-H is in blue, C-O is in red, and C=O is in green. Dashed line is the raw XPS data.

3.17 Nyquist plot of GCE-H3 response to *3NACP* in presence of Oseltamivir

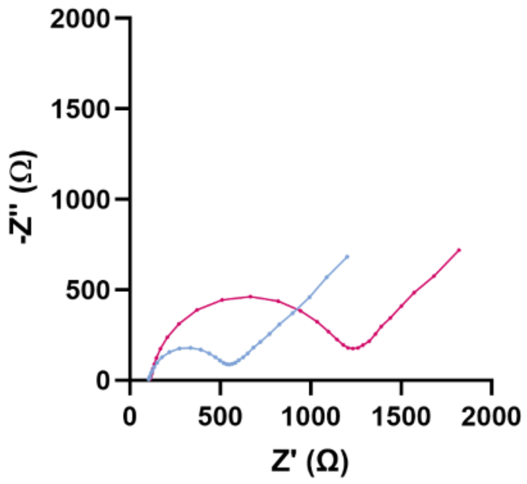

Figure S31: Nyquist plot of Impedimetric response of **GCE-H3** before (Blue) and after (Red) exposure to 3 mU/mL *3NACP* in the presence of 1 µM oseltamivir.

3.18 Nyquist plot of AuE-H3 response to *3NACP* in presence of Oseltamivir

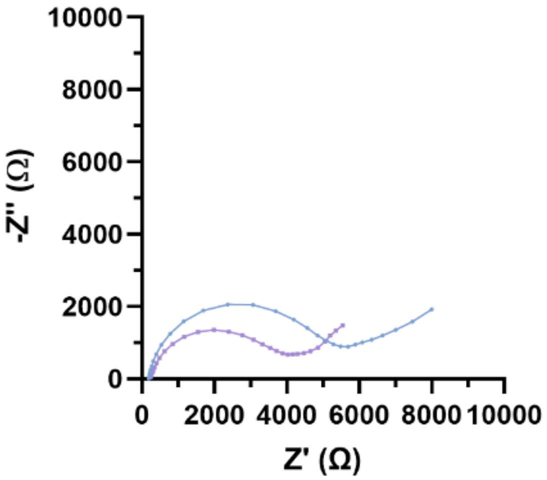

Figure S32: Nyquist plot of Impedimetric response of **AuE-H3** before (Blue) and after (Purple) exposure to 3 mU/mL *3NACP* in the presence of 1 µM oseltamivir.

3.19 XPS analyses of Au-LPA before and after coupling with H3

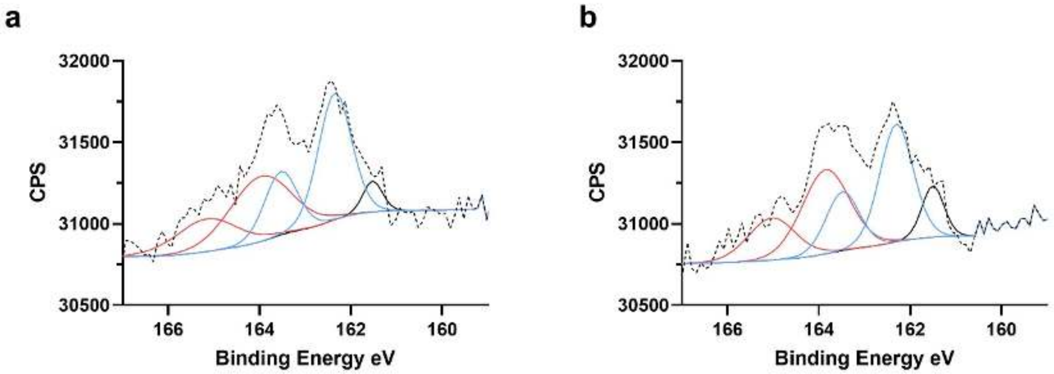

Figure S33: XPS analyses of S2p before coupling of the Glycan to the LPA (a) and after the coupling (b) where blue is bound S-Au and red is unbound. Dashed line is the raw data.

3.20 XPS data for GCE

Table 1- Atomic concentration report for Modified GCE before exposure to enzyme

	Atomic conc. [%]	Error [%]	Mass conc. [%]	Error [%]
S 2p	0,41	0,03	1,05	0,07
C 1s	85,06	0,23	80,84	0,27
N 1s	1,78	0,15	1,97	0,17
O 1s	12,75	0,19	16,15	0,23

Table 2- Atomic concentration report for modified GCE after exposure to enzyme

	Atomic conc. [%]	Error [%]	Mass conc. [%]	Error [%]
C 1s	81,18	0,33	76,61	0,37
N 1s	4,41	0,24	4,85	0,27
O 1s	14,07	0,27	17,69	0,32
S 2p	0,34	0,02	0,85	0,06

3.21 Contact Angle Measurements

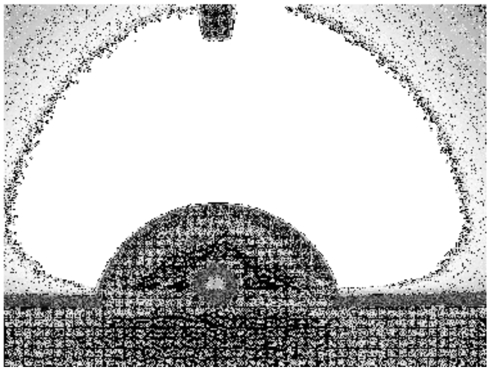

Figure S34: CA measurement of blank GCP

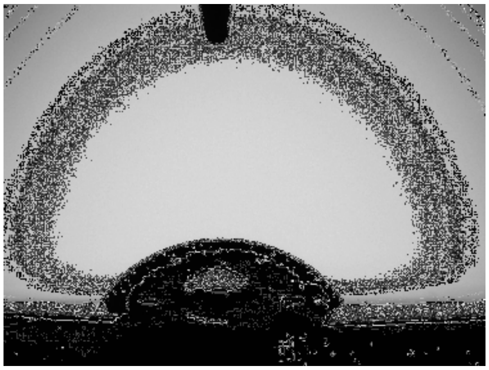

Figure S35: CA measurement of GCP modified with H3

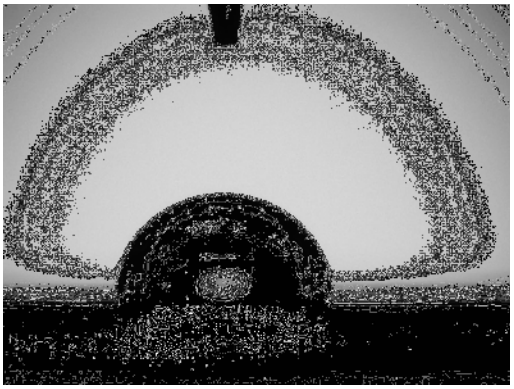

Figure S36: CA measurement of GCP modified with H3 after exposure to *3NACP*.

4. NMR and MS analyses of the compounds

NMR Analyses of compound 1

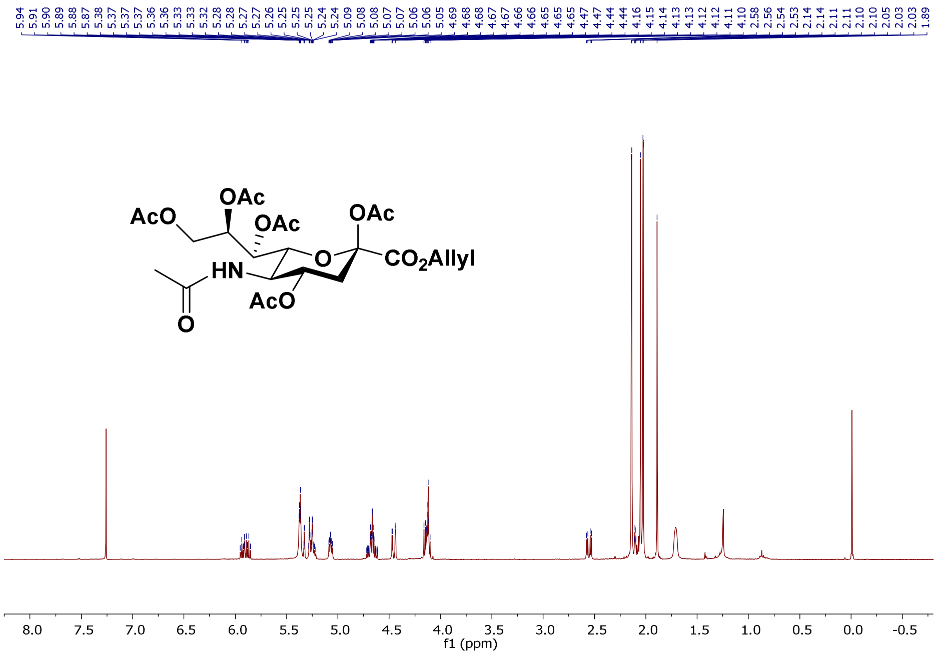

Figure S37: H1-NMR of compound 1

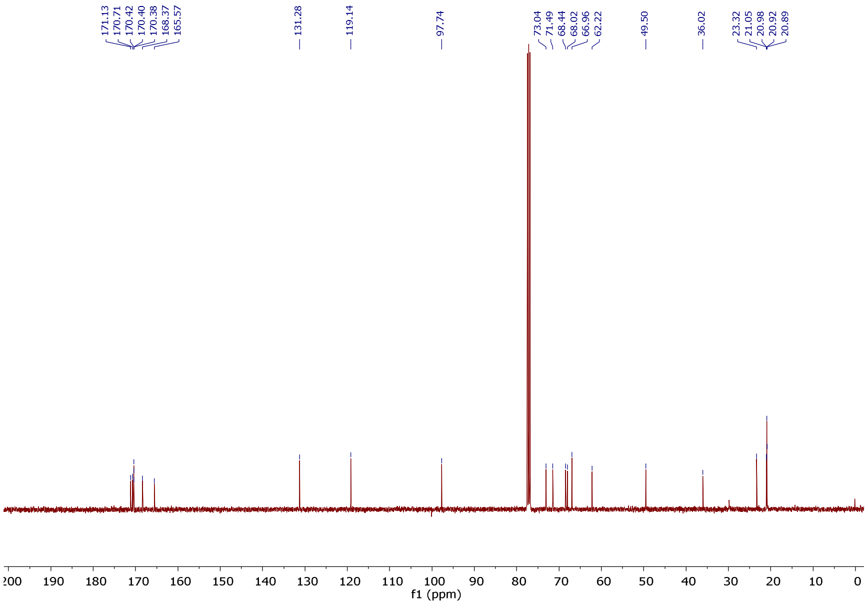

Figure S38: C13-NMR of compound 1

NMR Analyses of compound 2

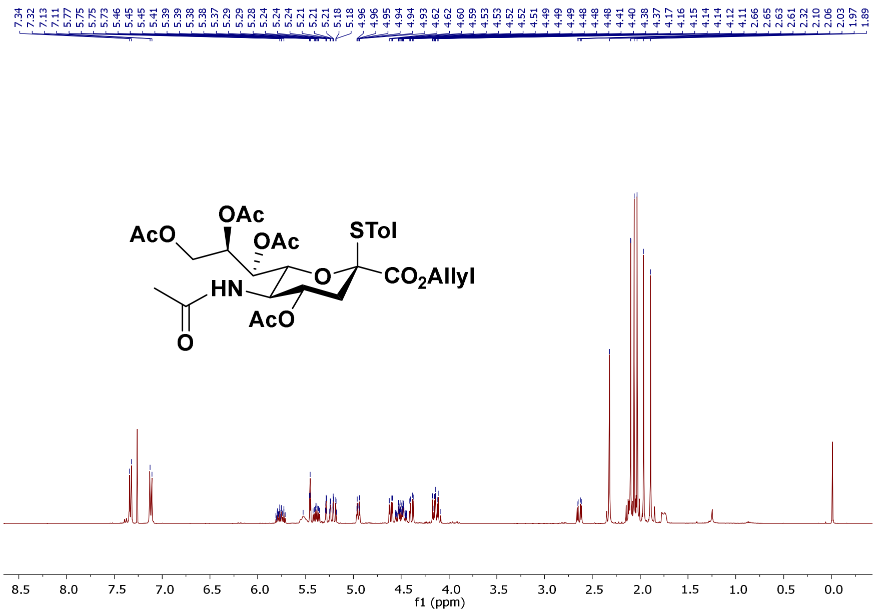

Figure S39: H1-NMR of compound 2

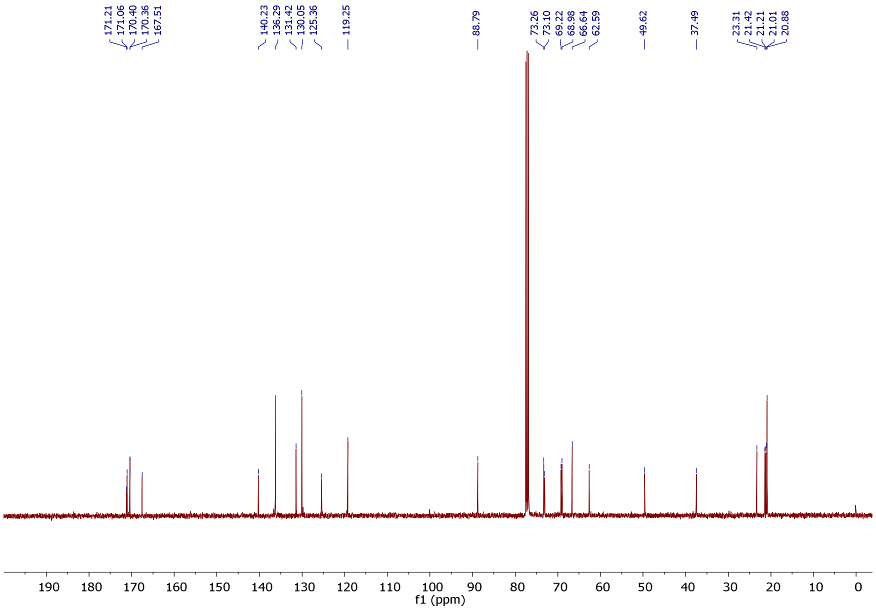

Figure S40: C13-NMR of compound 2

NMR Analyses of compound 3a

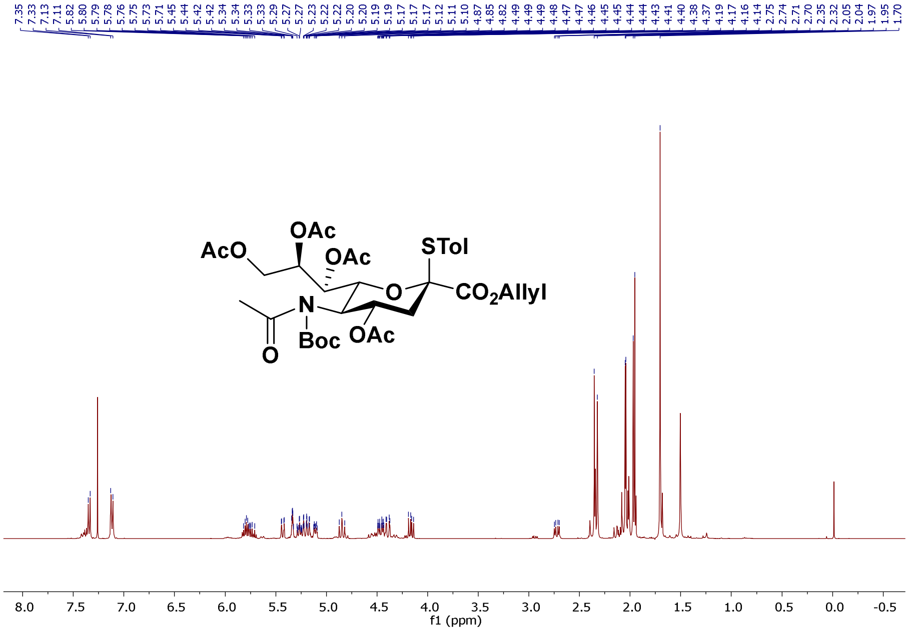

Figure S41: H1-NMR of compound 3a

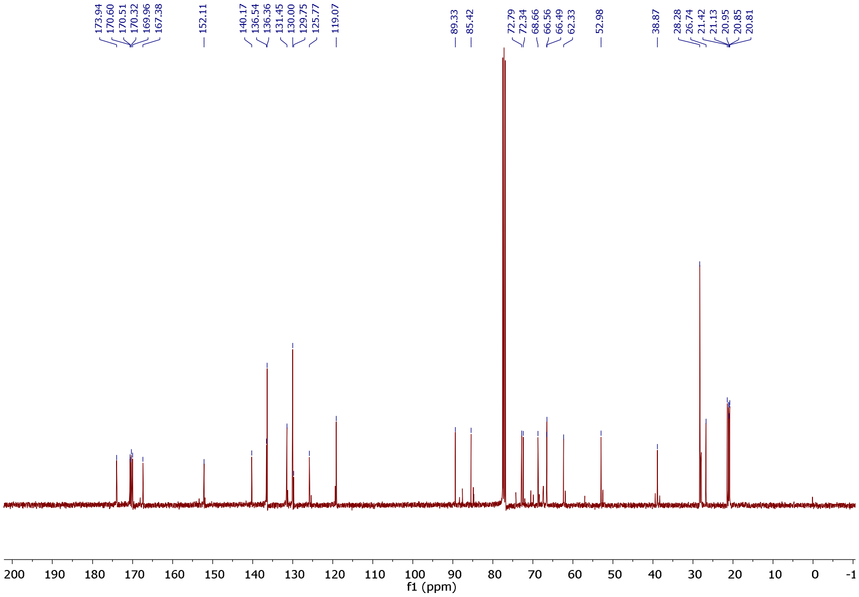

Figure S42: C13-NMR of compound 3a

NMR Analyses of compound 3b

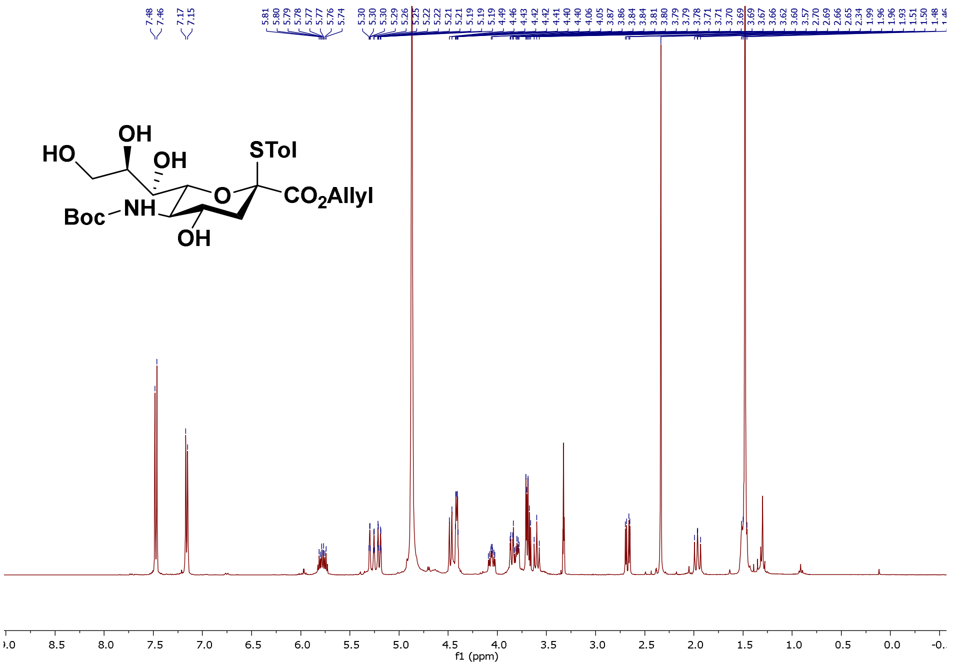

Figure S43: H1-NMR of compound 3b

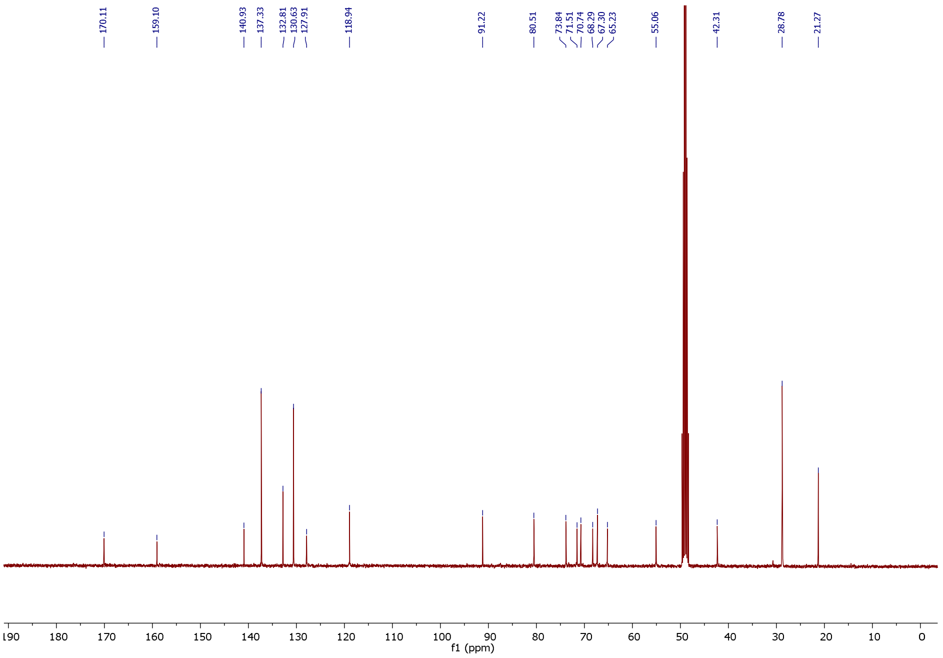

Figure S44: C13-NMR of compound 3b

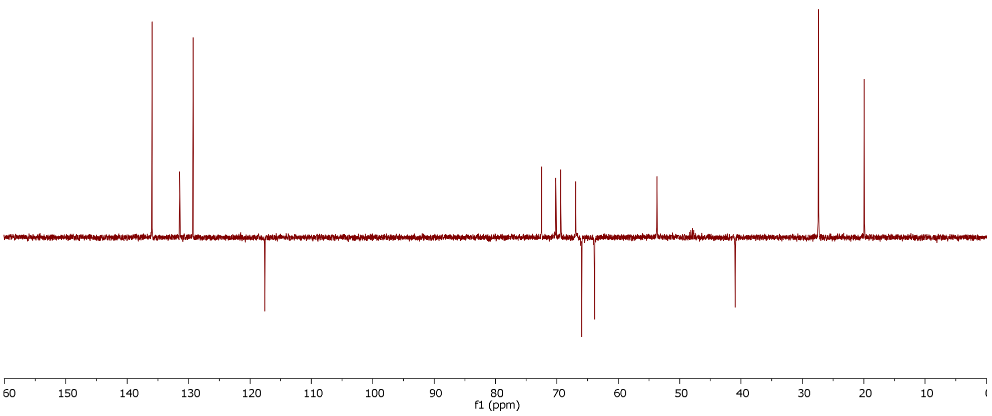

Figure S45: DEPT-NMR of compound 3b

NMR Analyses of compound 3

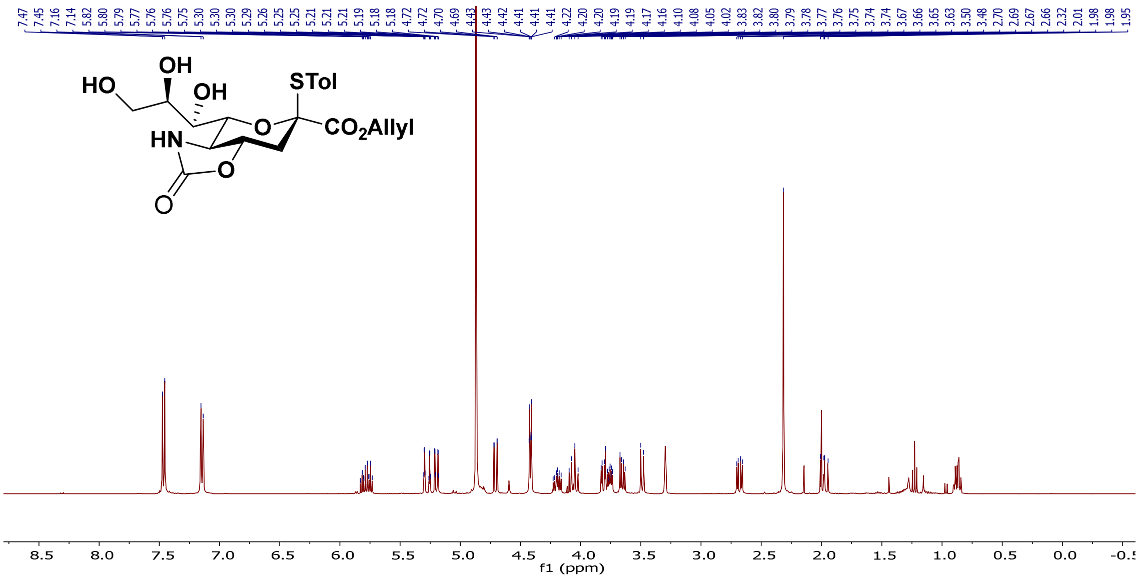

Figure S46: H1-NMR of compound 3

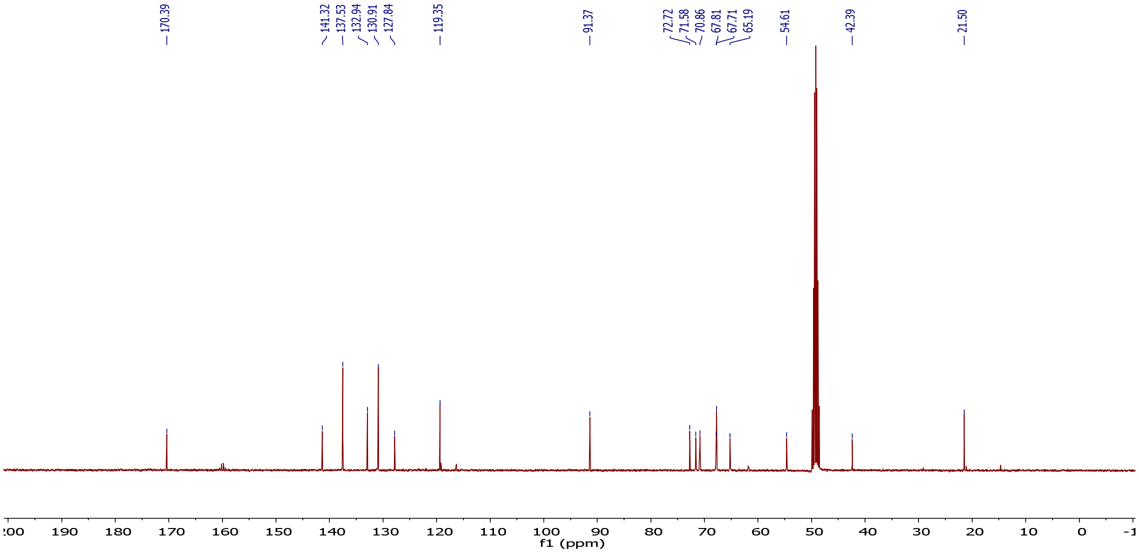

Figure S47: C13-NMR of compound 3

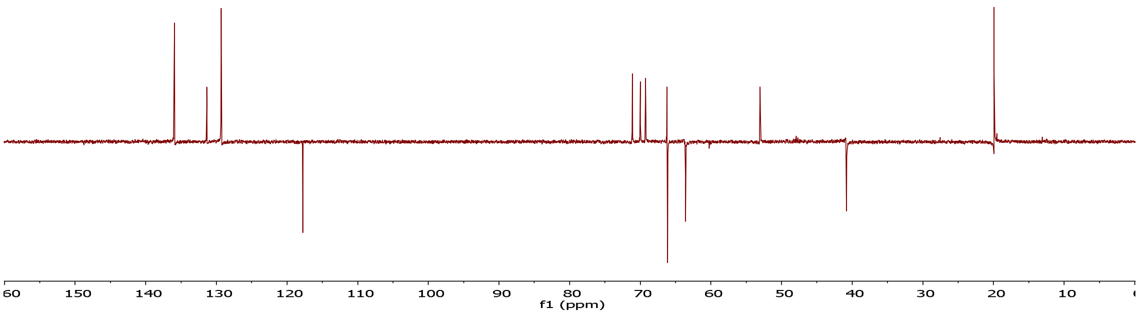

Figure S48: DEPT-NMR of compound 3

NMR Analyses of compound 4a

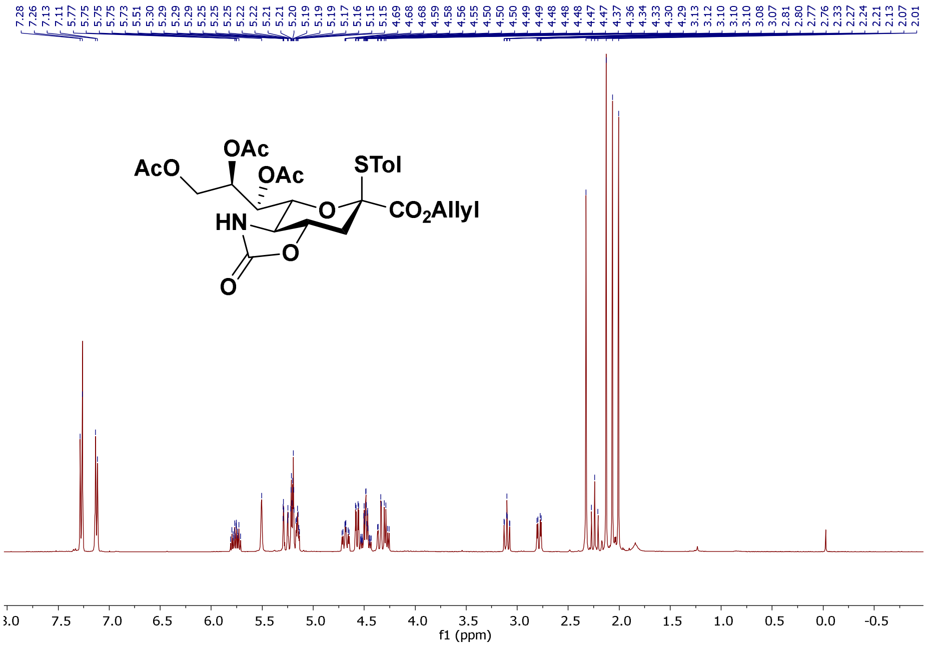

Figure S49: H1-NMR of compound 4a

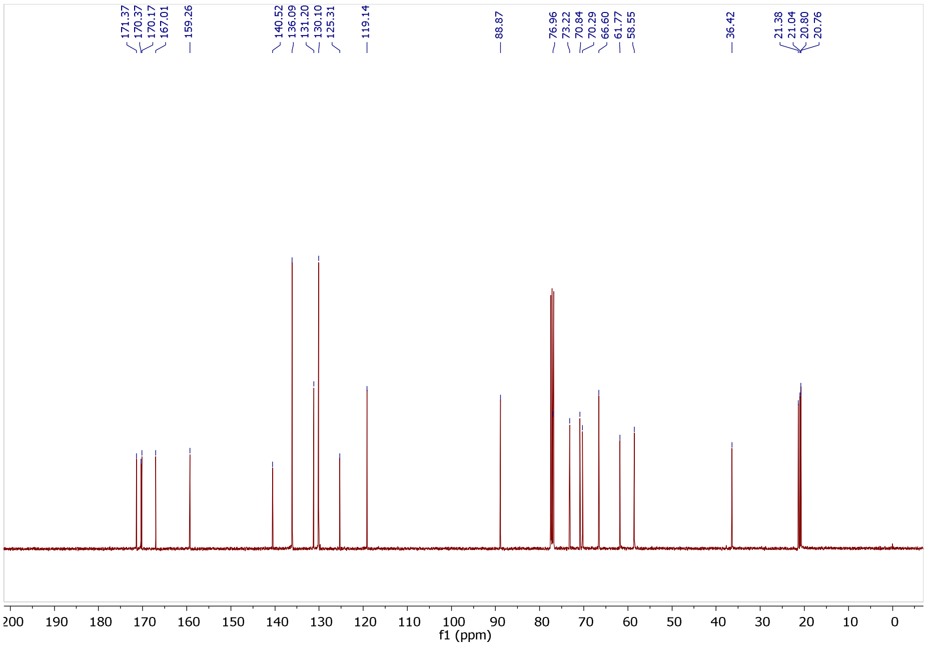

Figure S50: C13-NMR of compound 4a

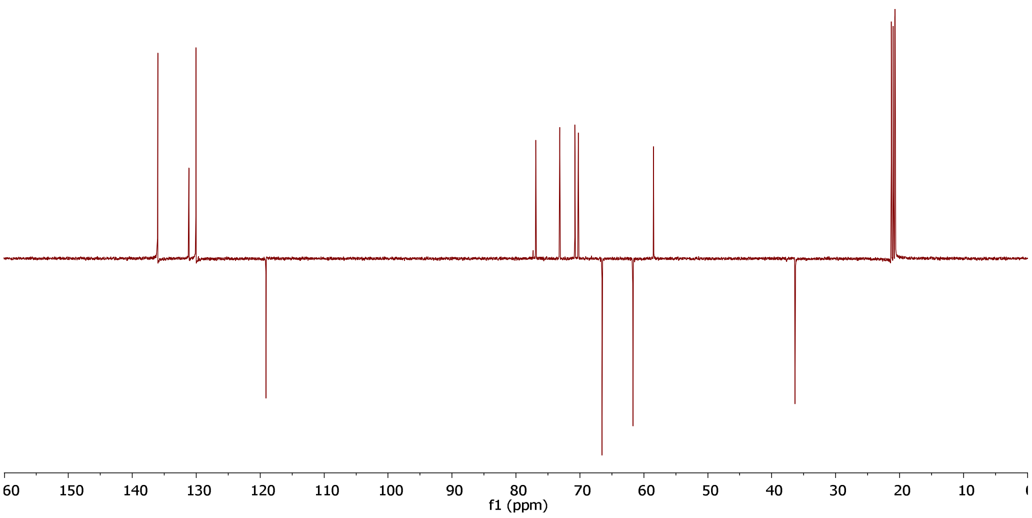

Figure S51: DEPT-NMR of compound 4a

NMR Analyses of compound 4

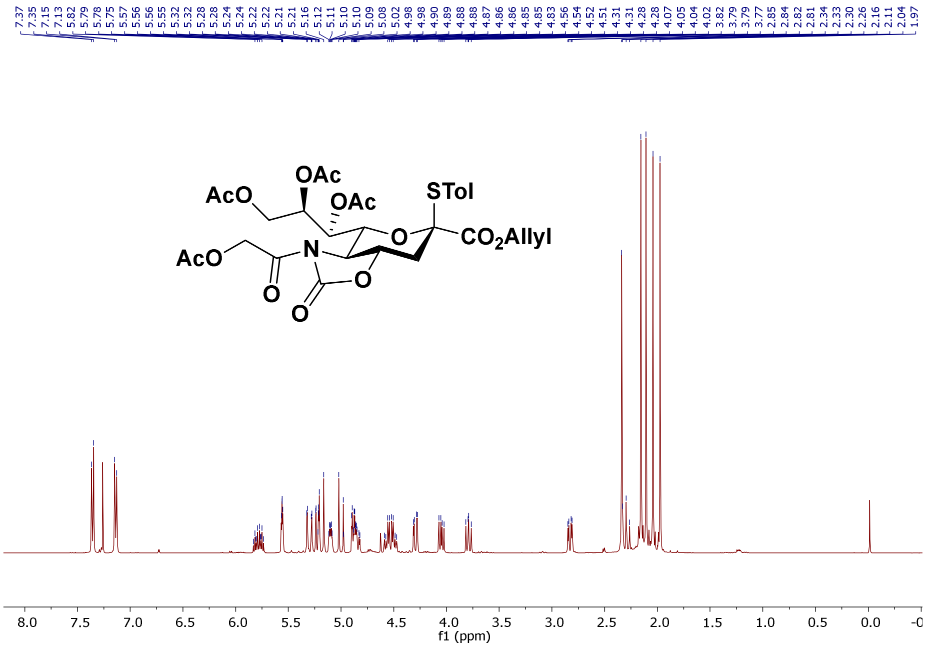

Figure S52: H1-NMR of compound 4

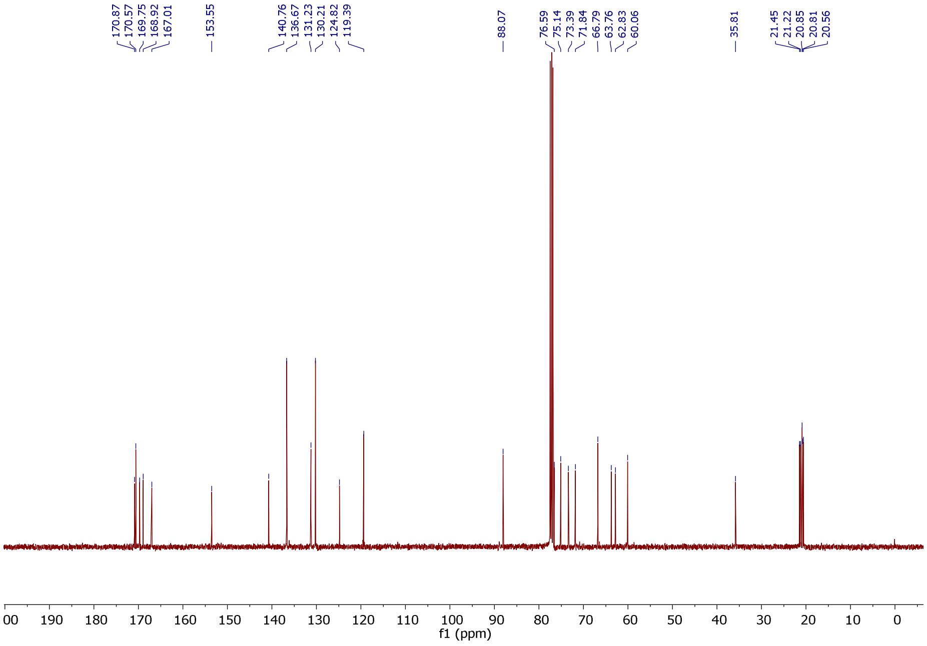

Figure S53: C13-NMR of compound 4

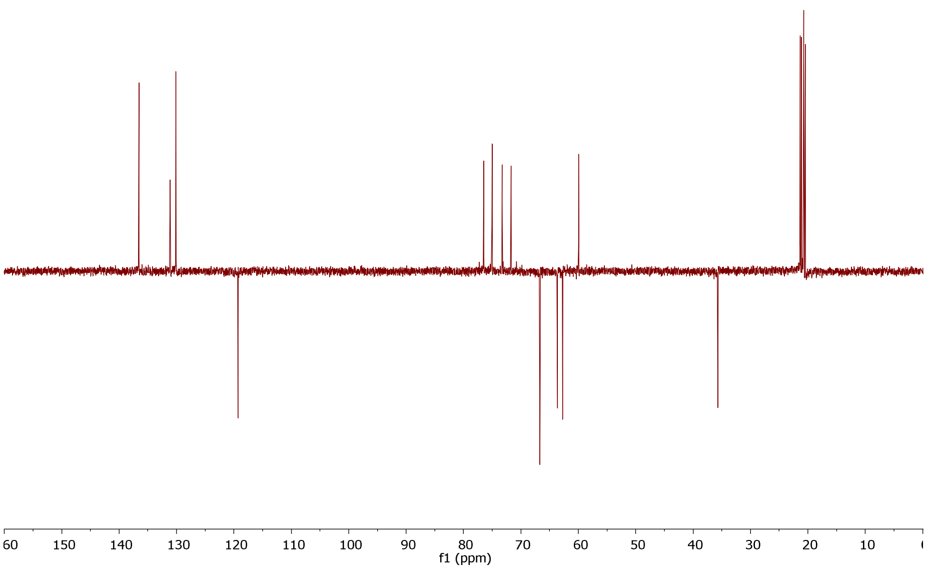

Figure S54: DEPT-NMR of compound 4

NMR Analyses of compound 5

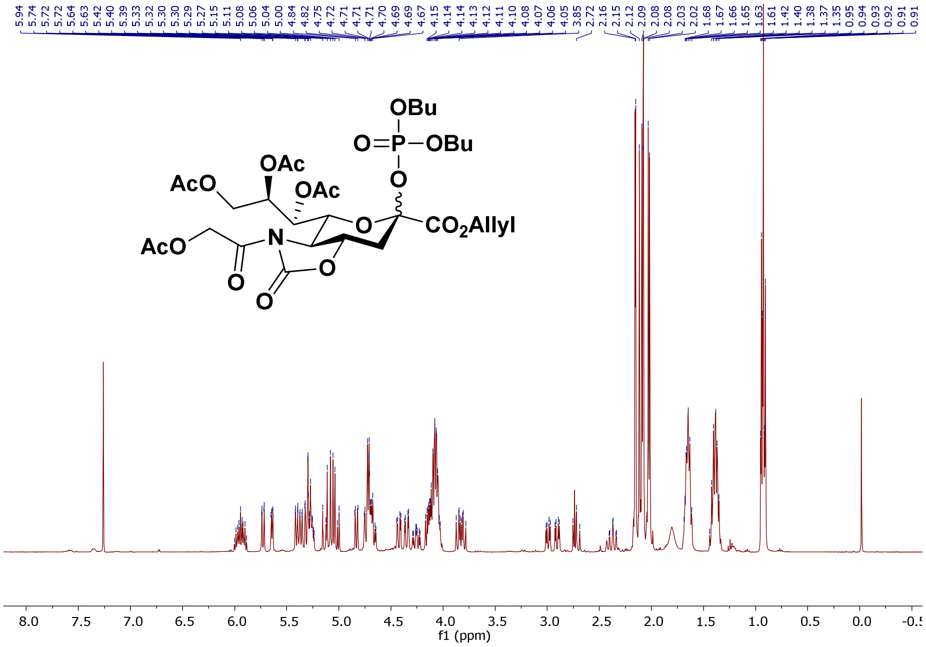

Figure S55: H1-NMR of compound 5

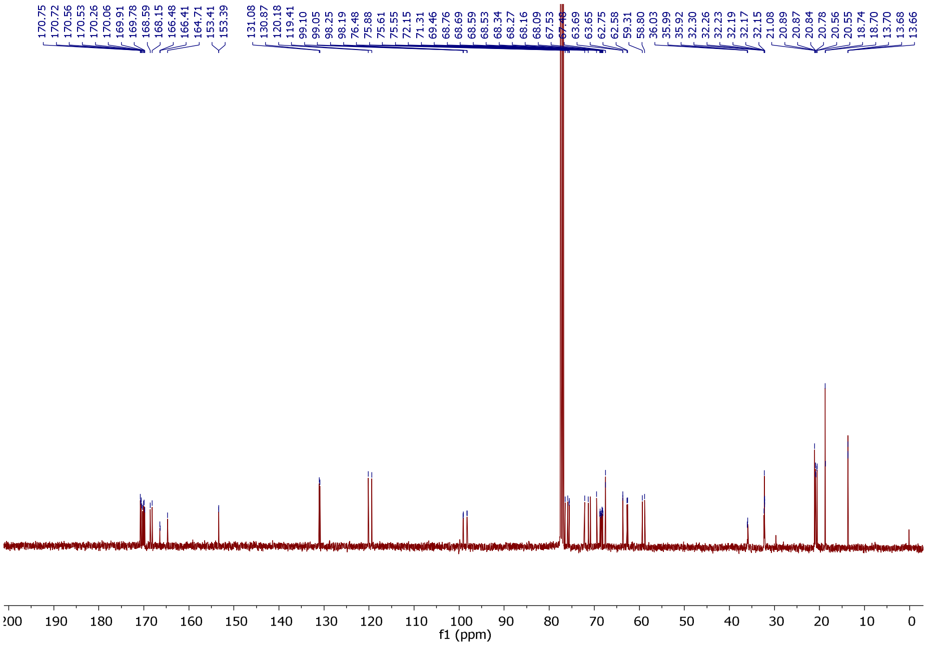

Figure S56: C13-NMR of compound 5

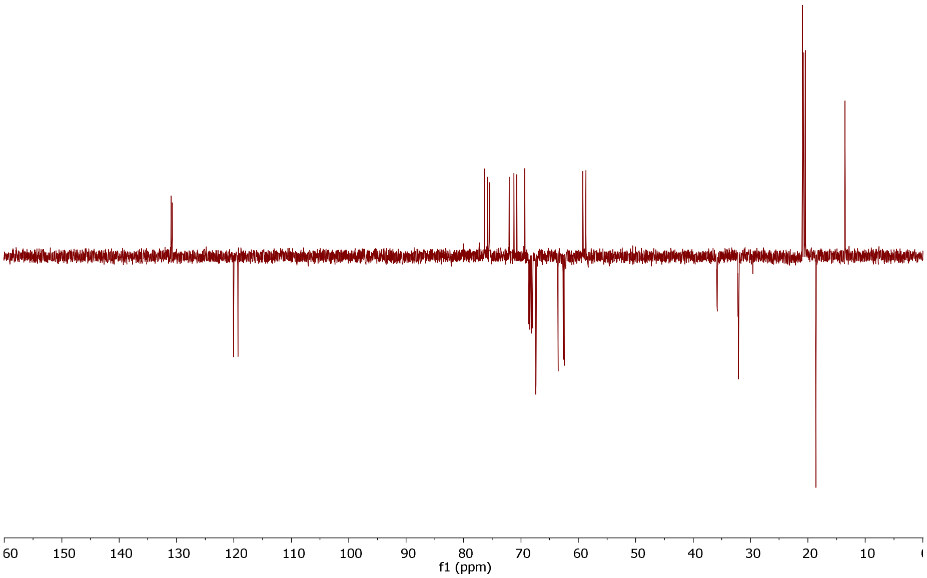

Figure S57: DEPT-NMR of compound 5

<u>NMR Analyses of compound 6a</u>

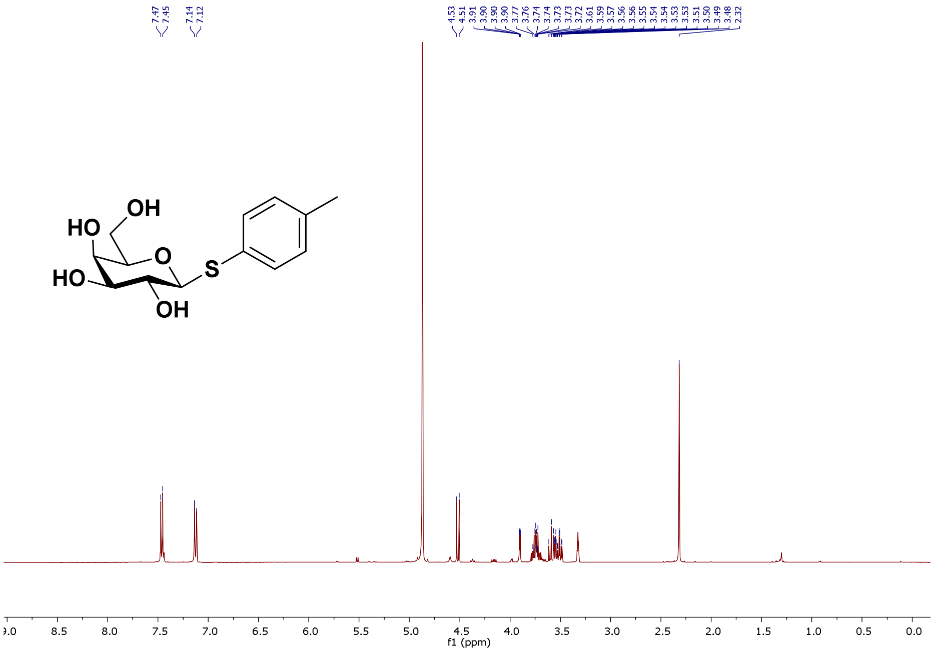

Figure S58: H1-NMR of compound 6a

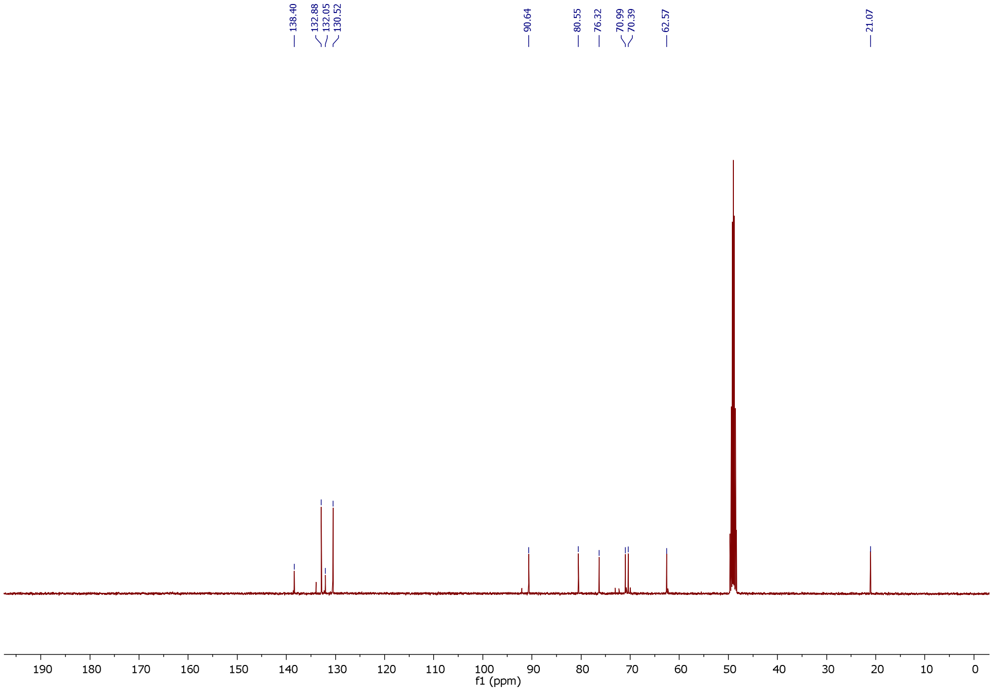

Figure S57: C13-NMR of compound 6a

<u>NMR Analyses of compound 6b</u>

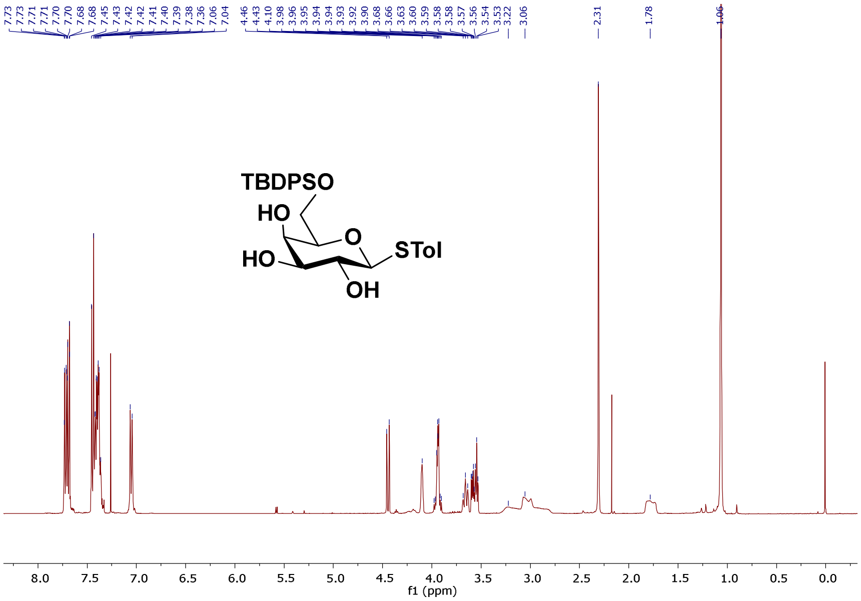

Figure S58: H1-NMR of compound 6b

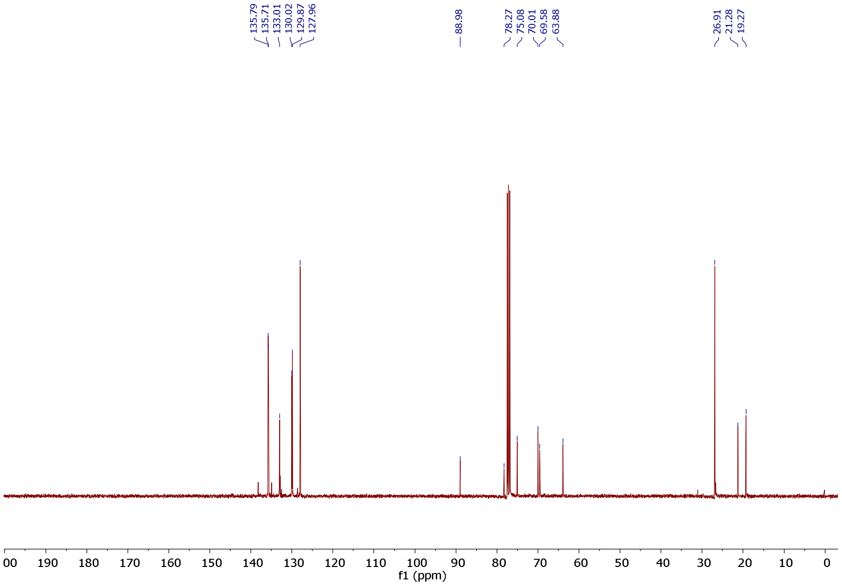

Figure S61: C13-NMR of compound 6b

NMR Analyses of compound 6c

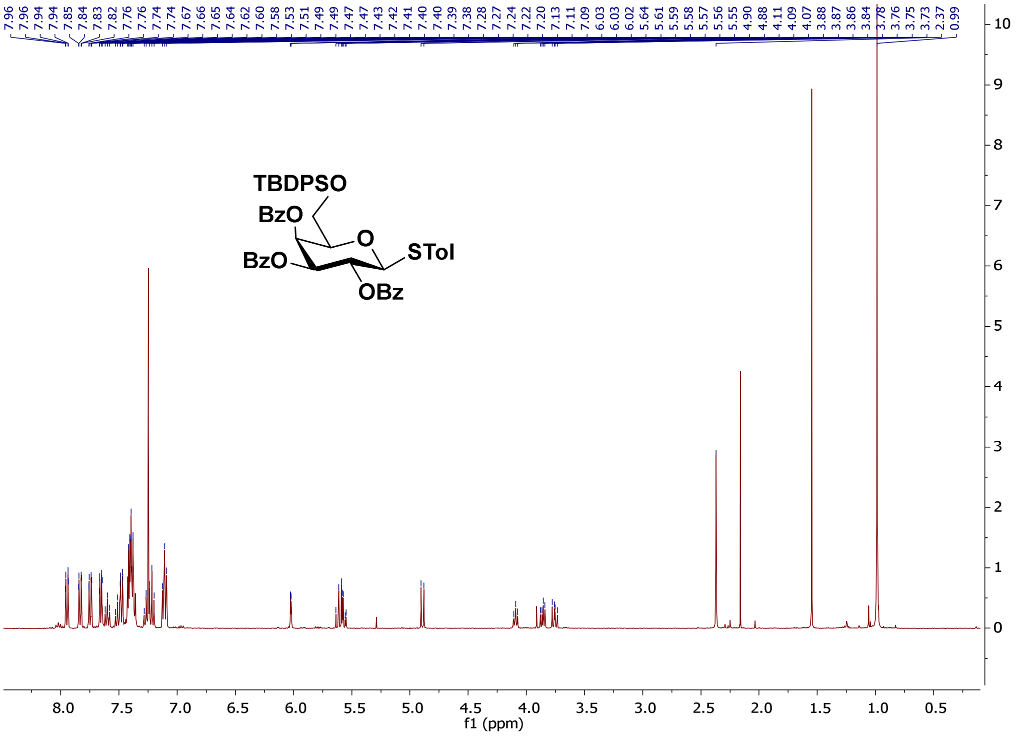

Figure S62: H1-NMR of compound 6c

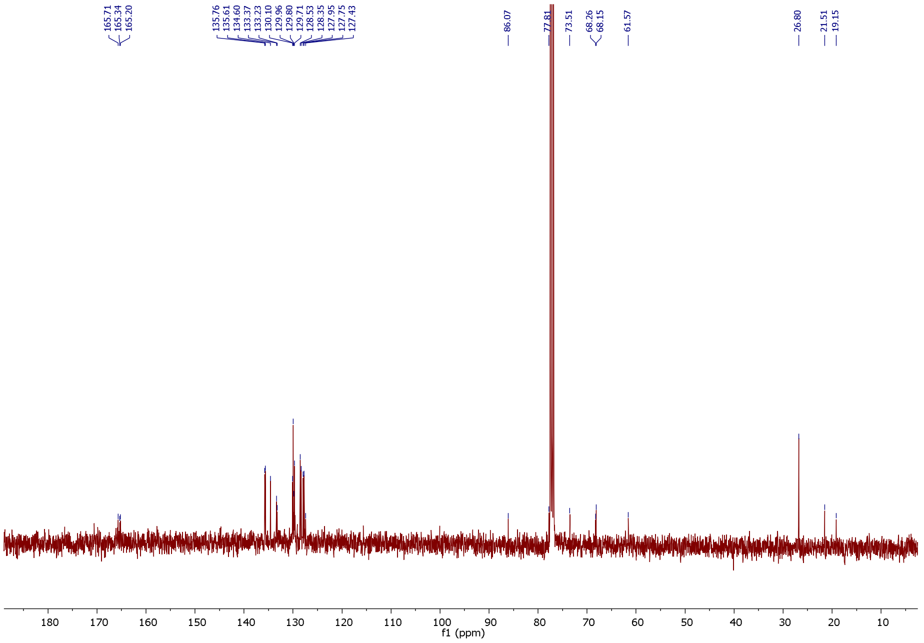

Figure S63: C13-NMR of compound 6c

NMR Analyses of compound 6

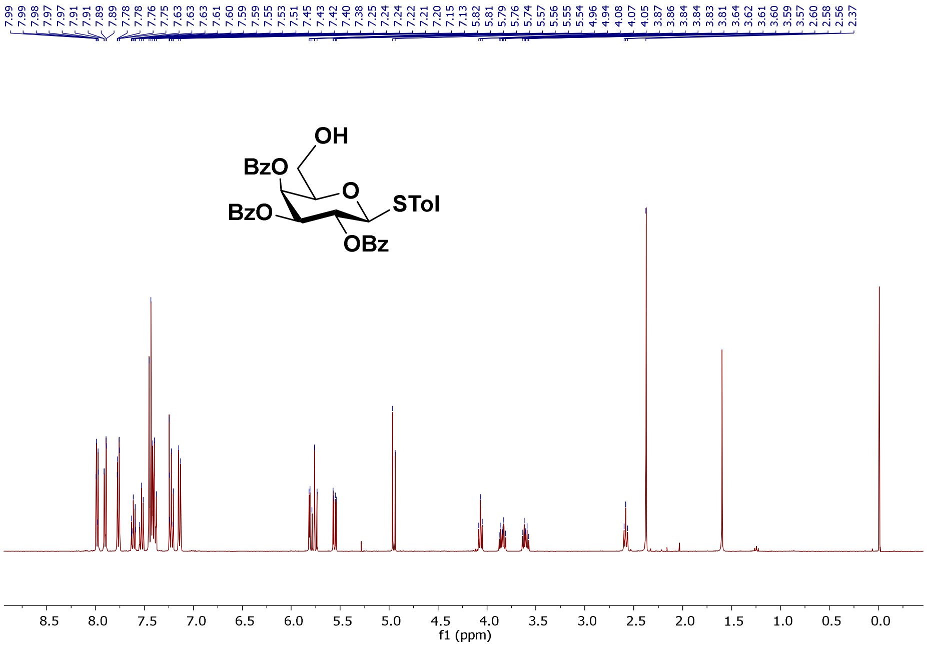

Figure S64: H1-NMR of compound 6

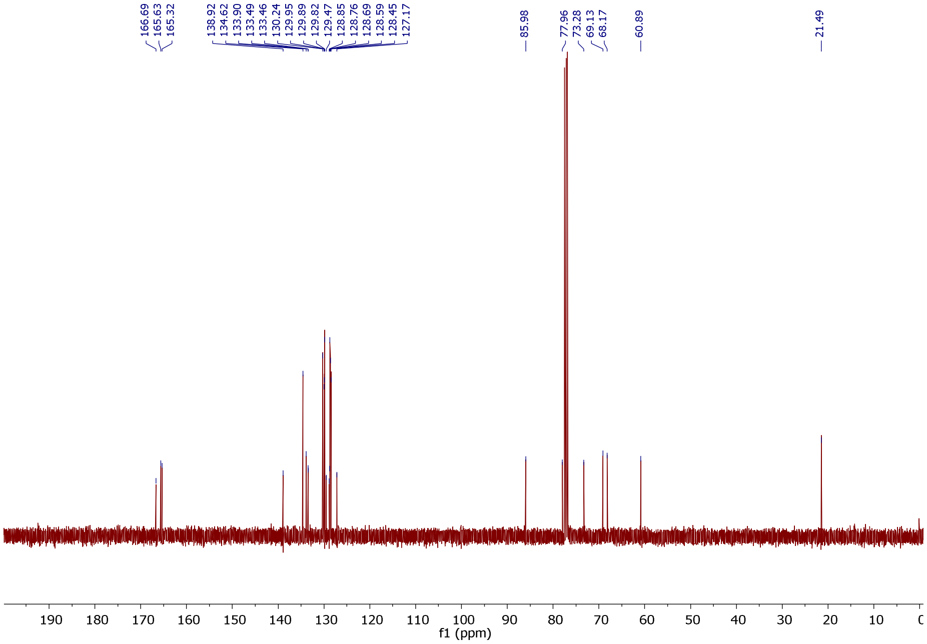

Figure S65: C13-NMR of compound 6

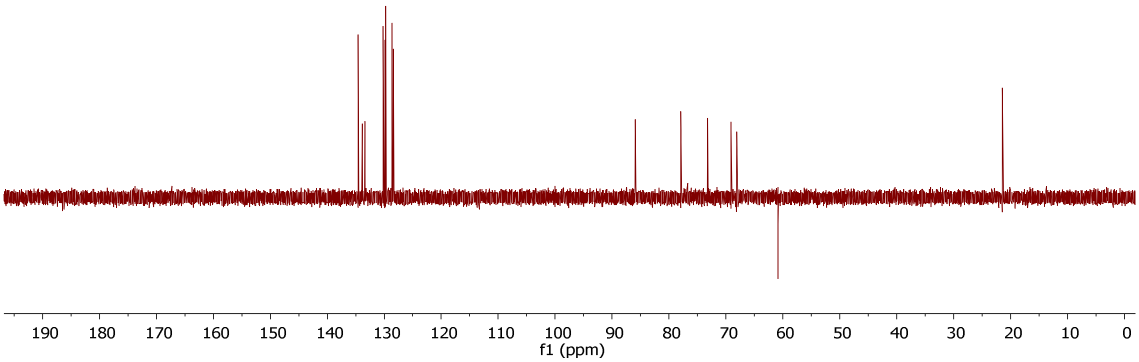

Figure S66: DEPT-NMR of compound 6

NMR Analyses of compound 7a

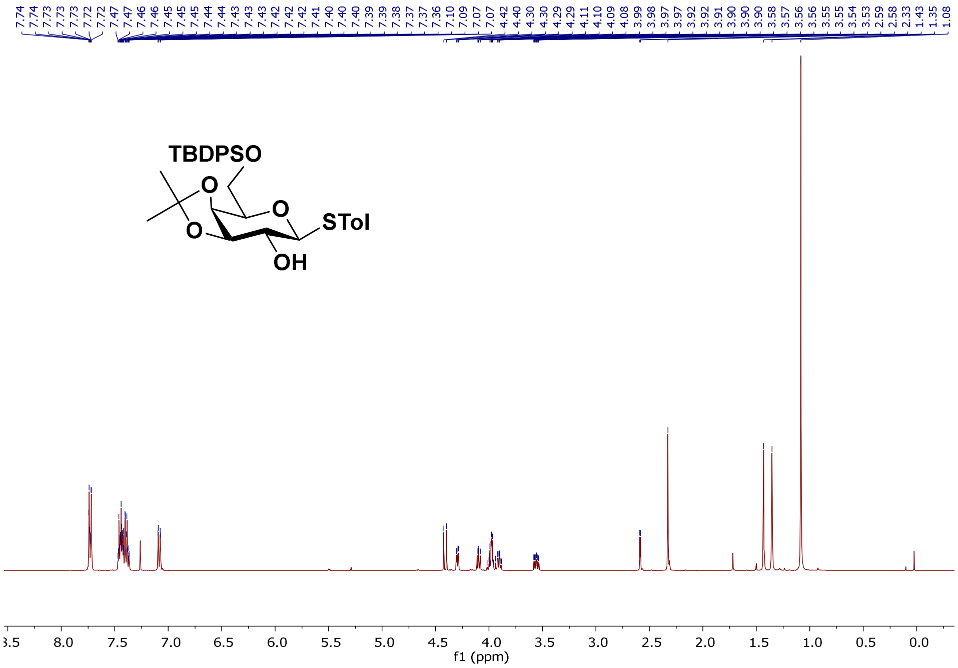

Figure S67: H1-NMR of compound 7a

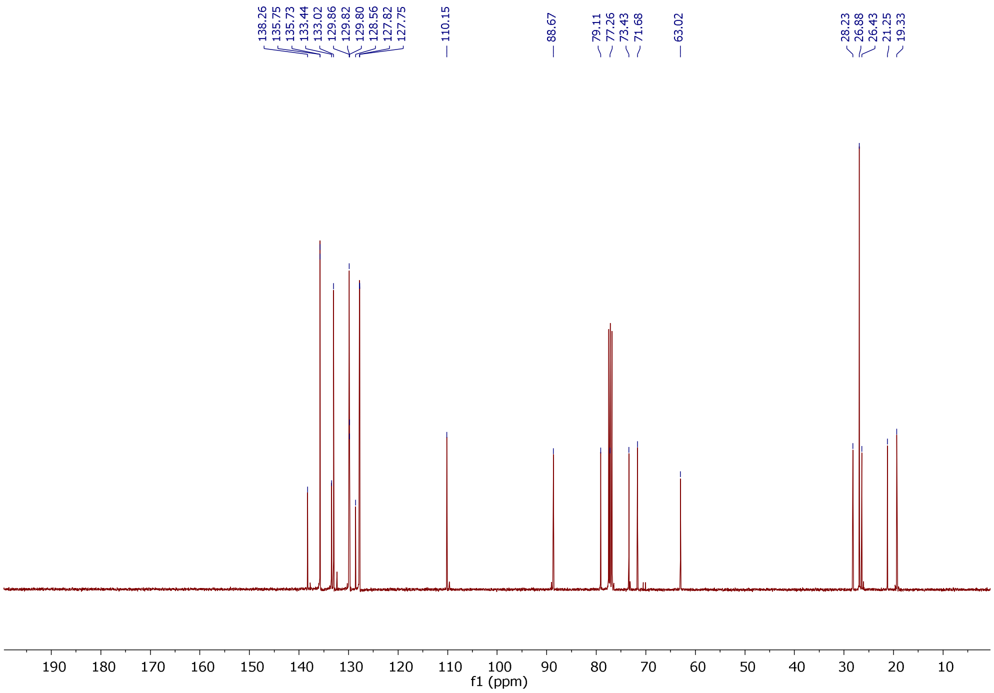

Figure S68: C13-NMR of compound 7a

NMR Analyses of compound 7b

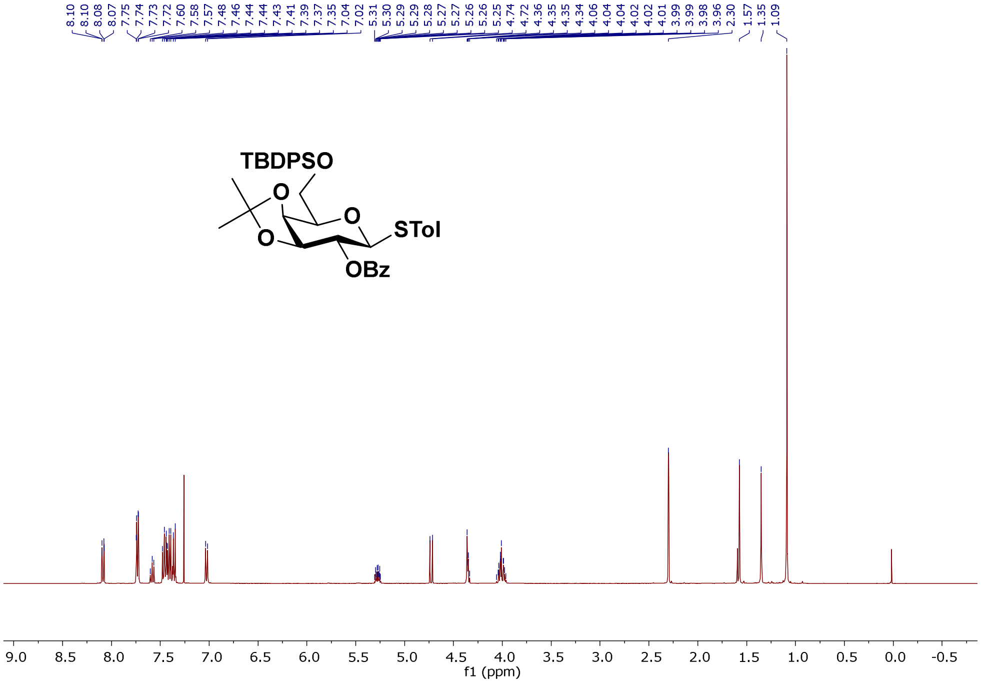

Figure S69: H1-NMR of compound 7b

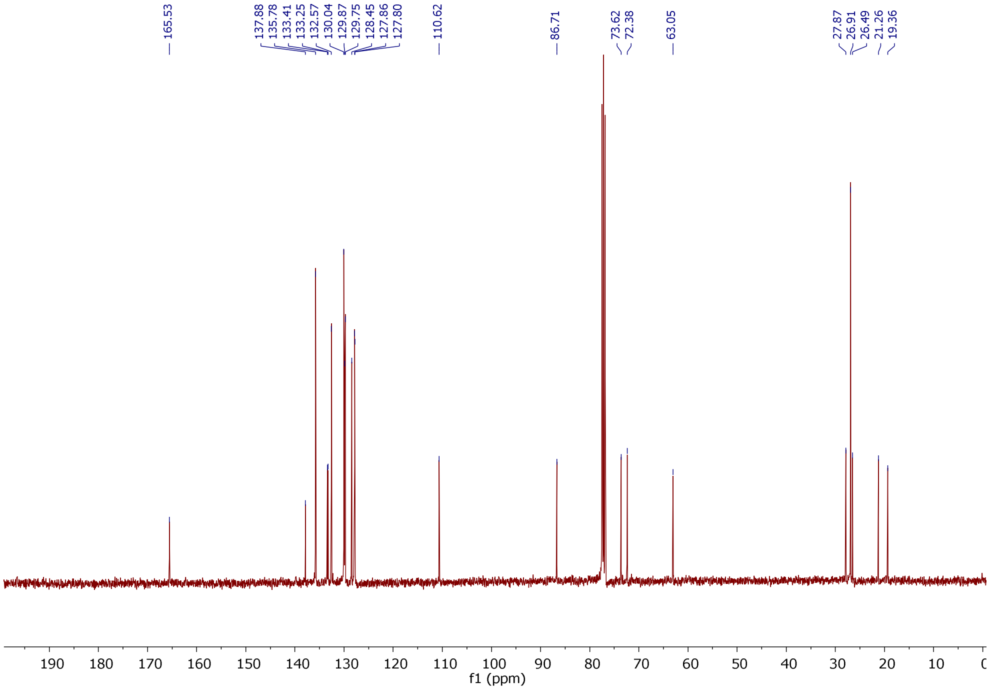

Figure S70: C13-NMR of compound 7b

NMR Analyses of compound 7c

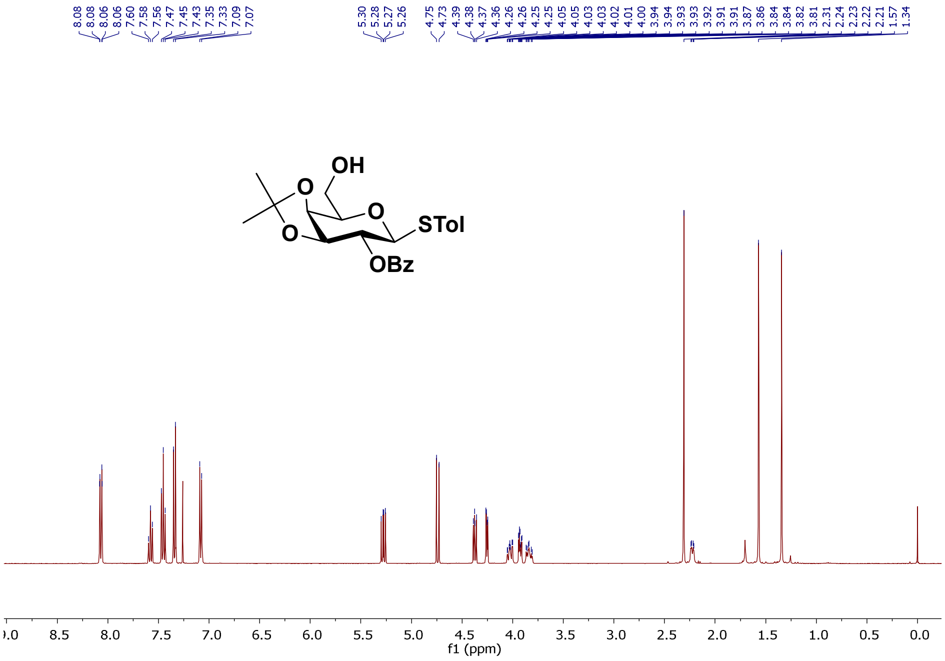

Figure S71: H1-NMR of compound 7c

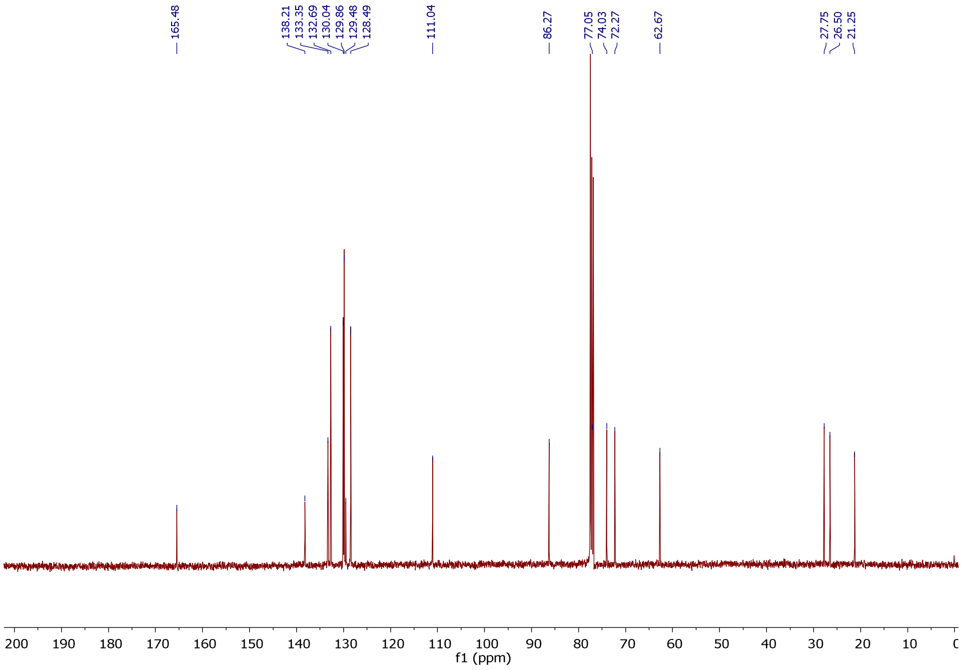

Figure S72: C13-NMR of compound 7c

NMR Analyses of compound 7d

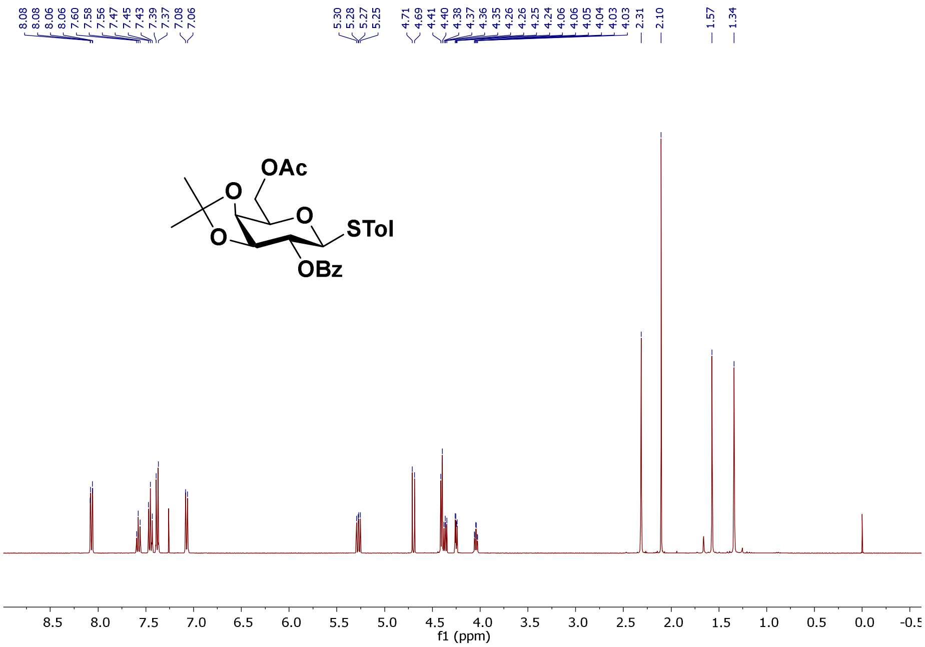

Figure S73: H1-NMR of compound 7d

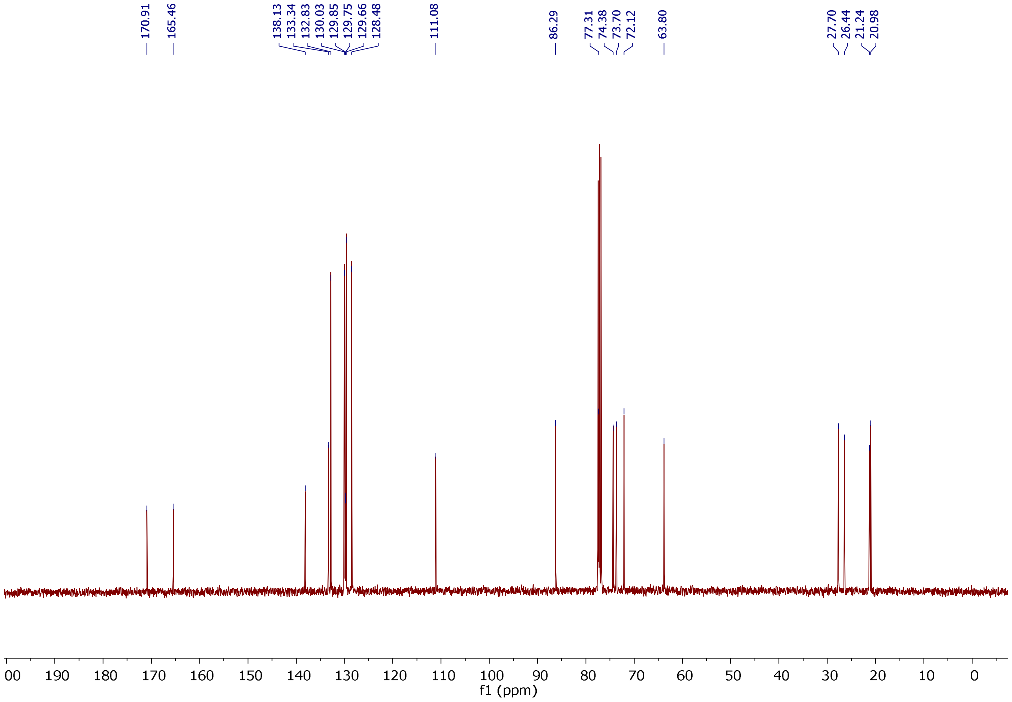

Figure S74: C13-NMR of compound 7d

<u>NMR Analyses of compound 7</u>

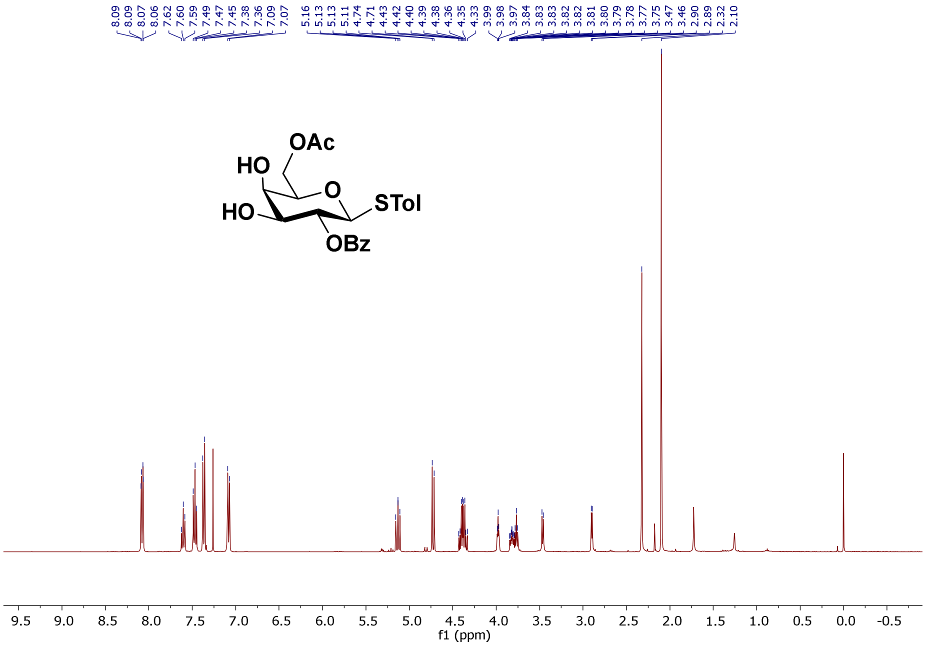

Figure S75: H1-NMR of compound 7

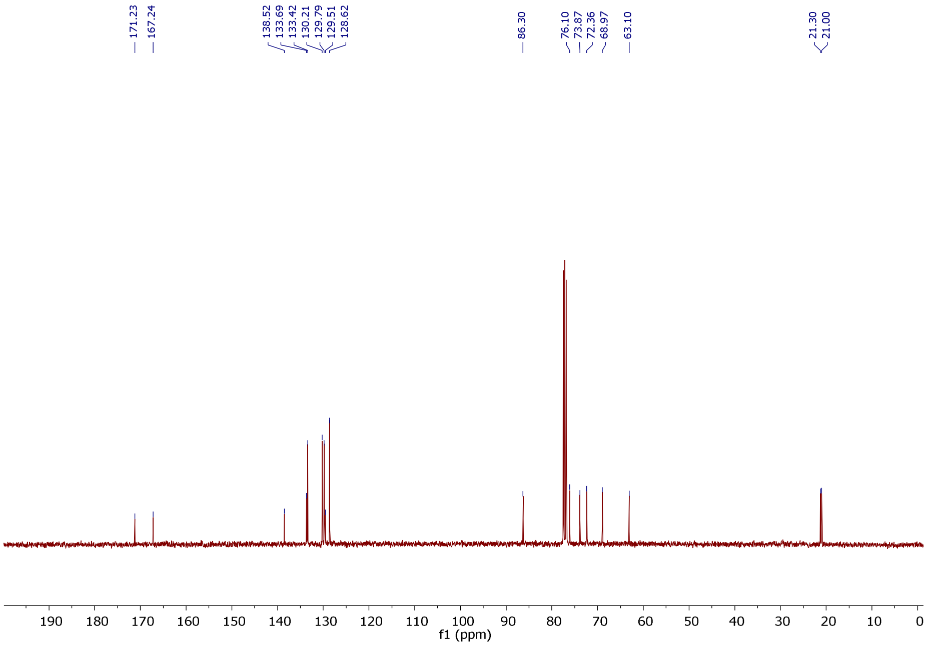

Figure S76: C13-NMR of compound 7

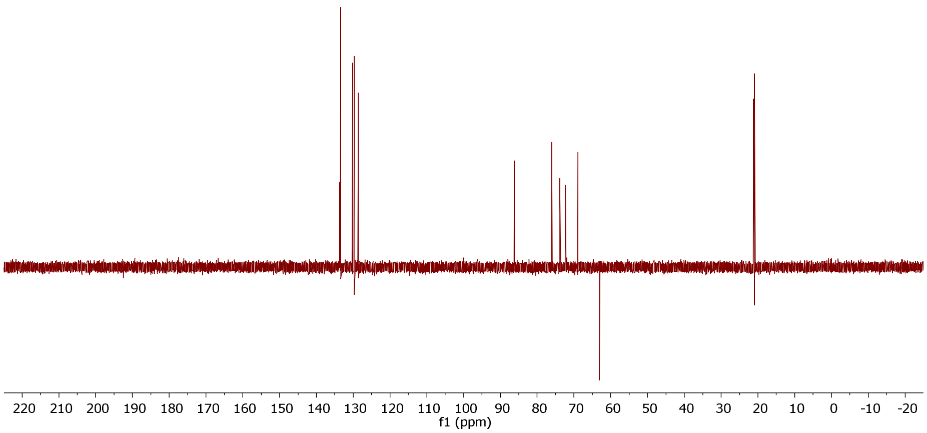

Figure S77: DEPT-NMR of compound 7

NMR Analyses of compound 8a

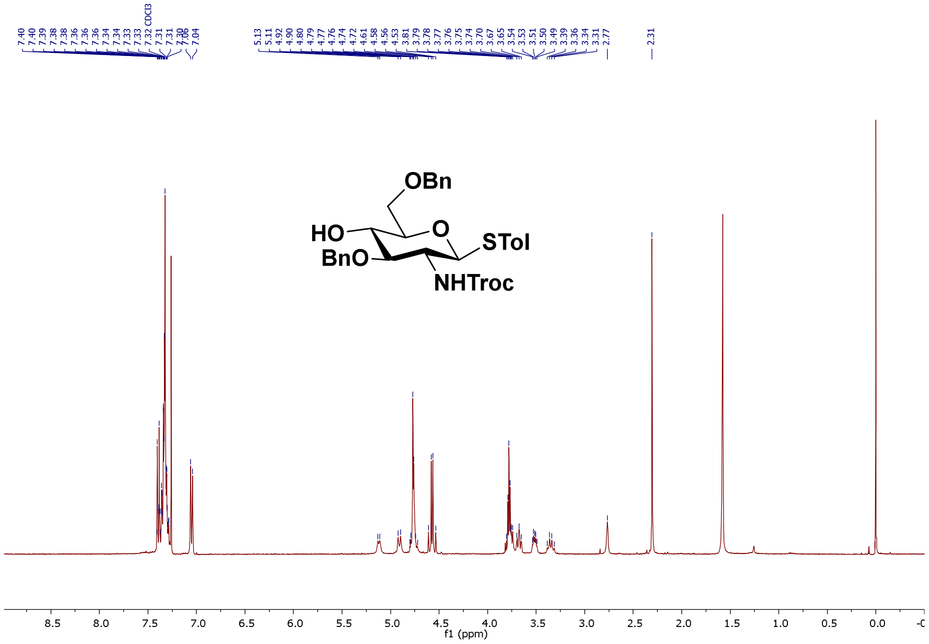

Figure S78: H1-NMR of compound 8a

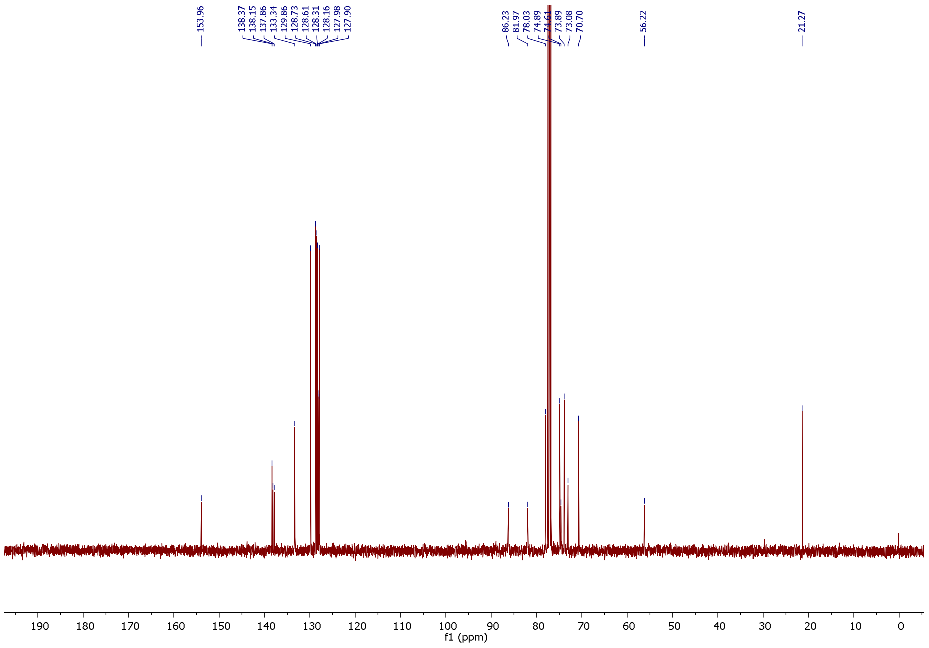

Figure S79: C13-NMR of compound 8a

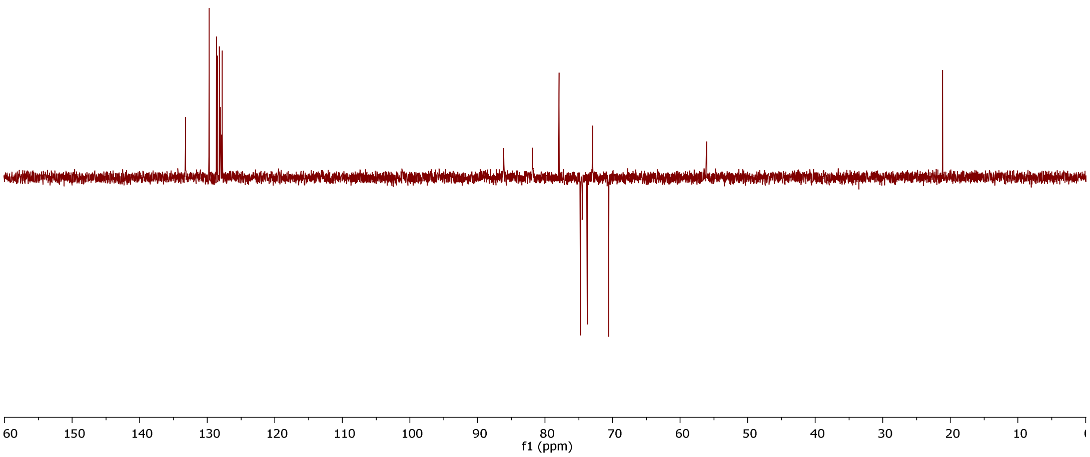

Figure S80: DEPT-NMR of compound 8a

NMR Analyses of compound 8

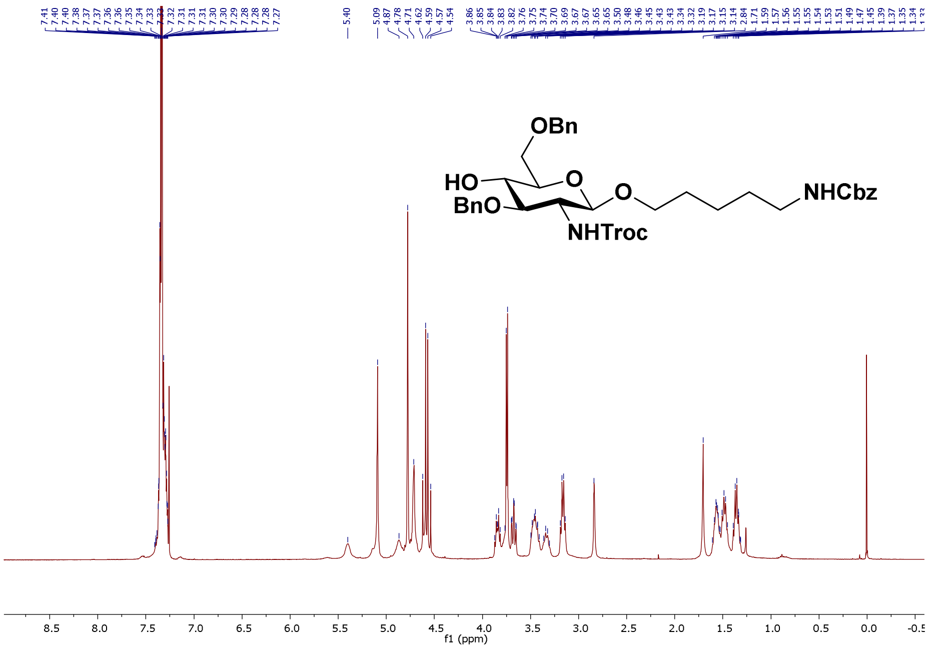

Figure S81: H1-NMR of compound 8

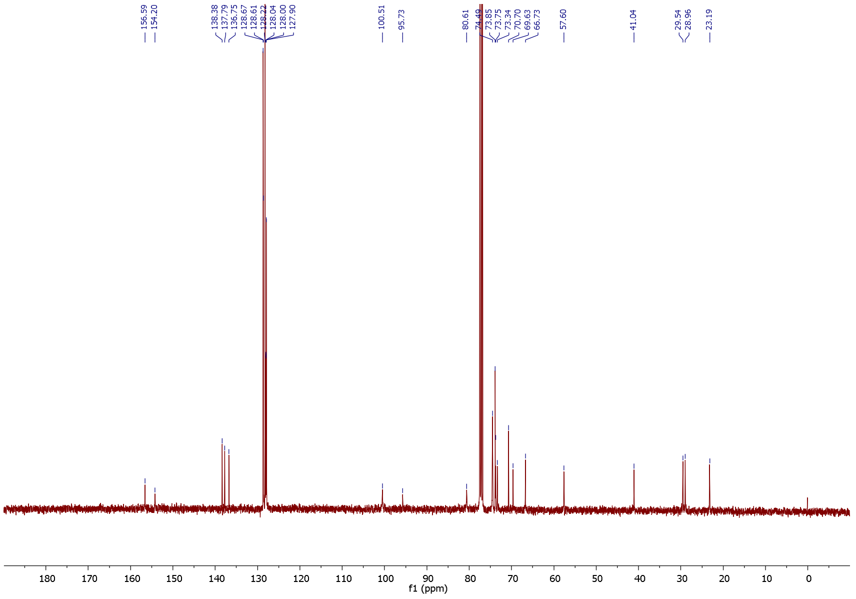

Figure S82: C13-NMR of compound 8

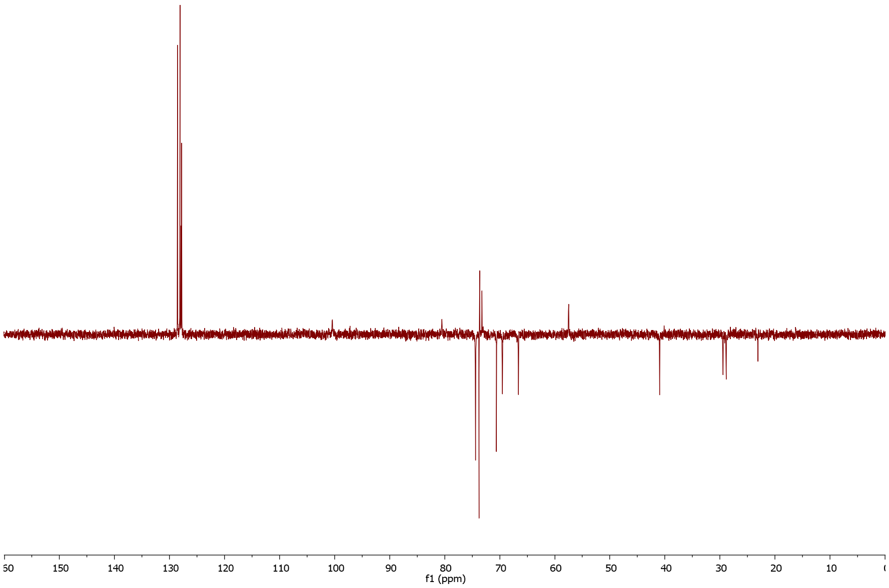

Figure S83: DEPT-NMR of compound 8

<u>NMR Analyses of compound 9</u>

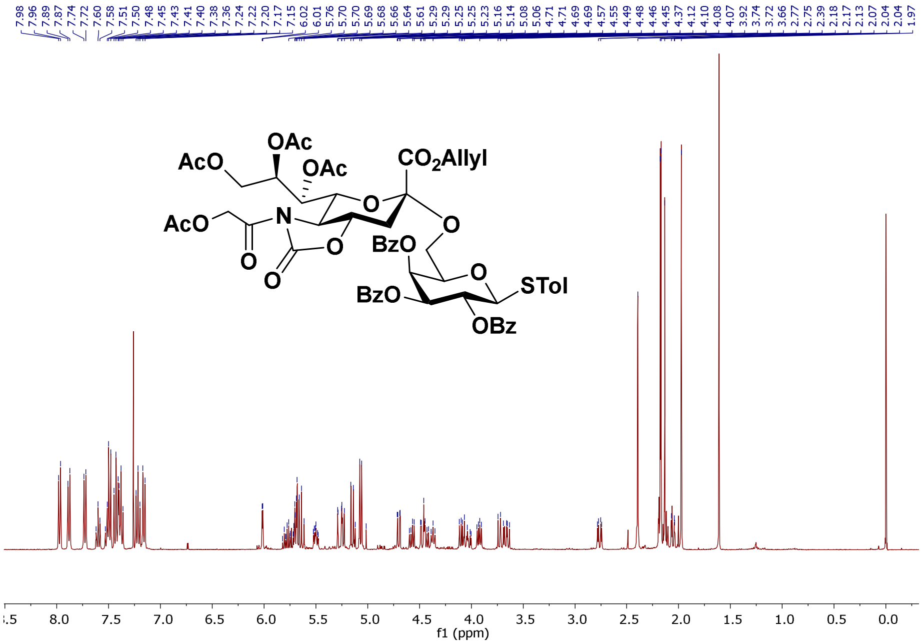

Figure S84: H1-NMR of compound 9

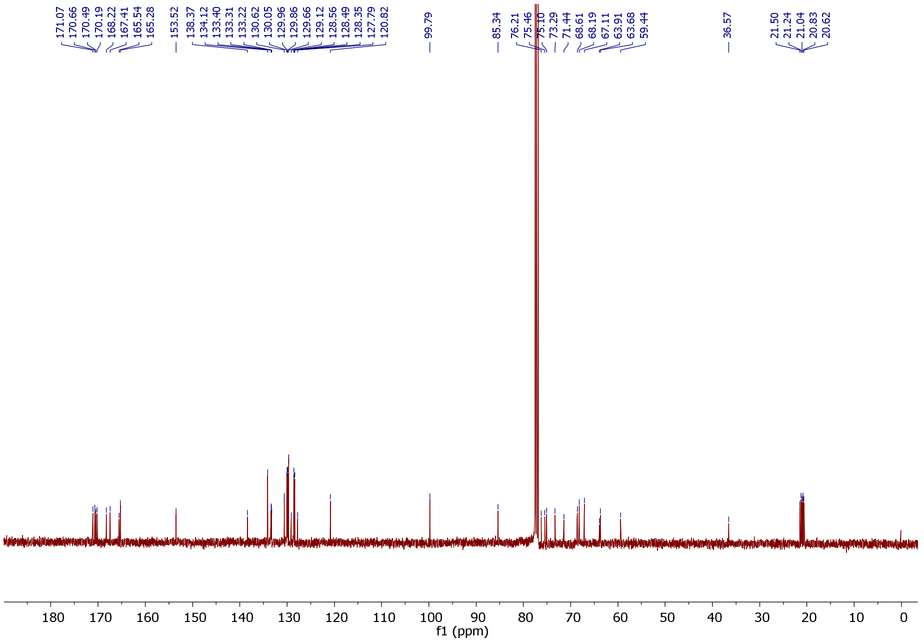

Figure S85: C13-NMR of compound 9

NMR Analyses of compound 11

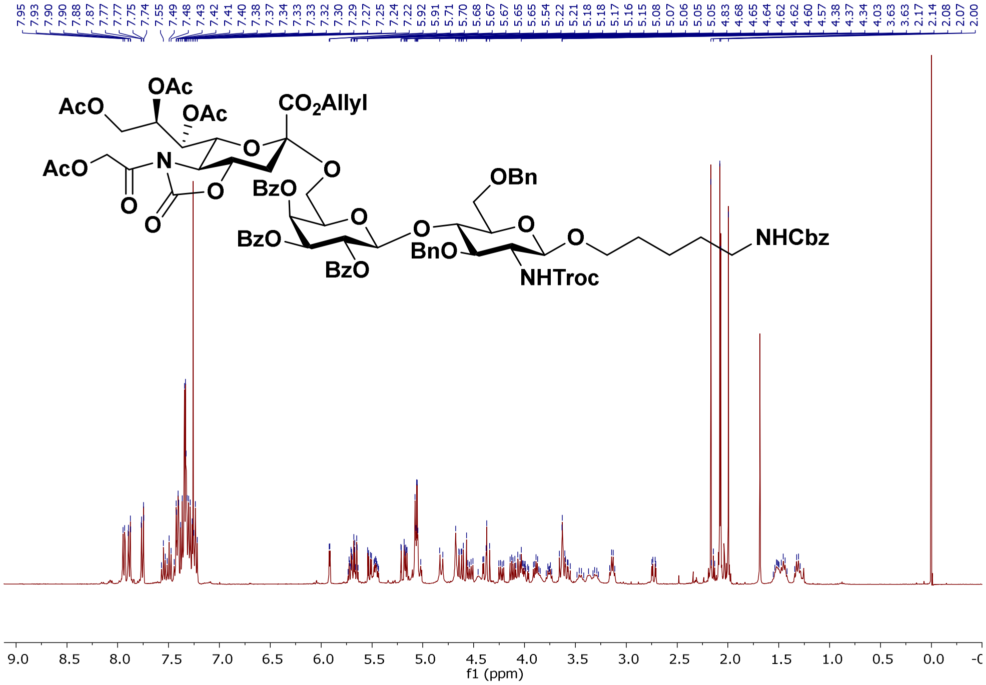

Figure S86: H1-NMR of compound 11

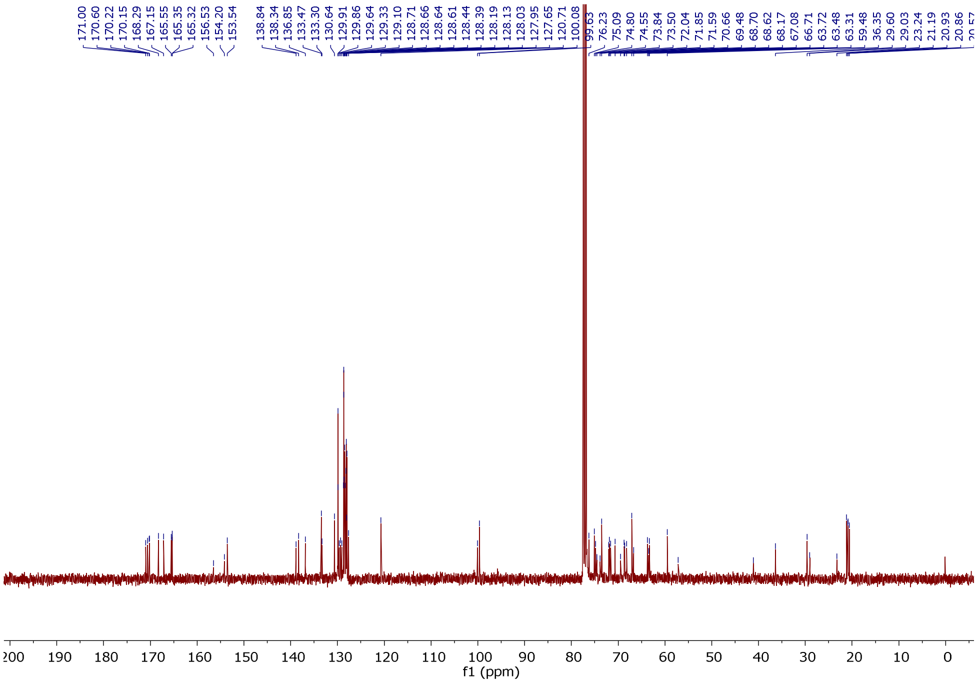

Figure S87: C13-NMR of compound 11

NMR Analyses of compound 13

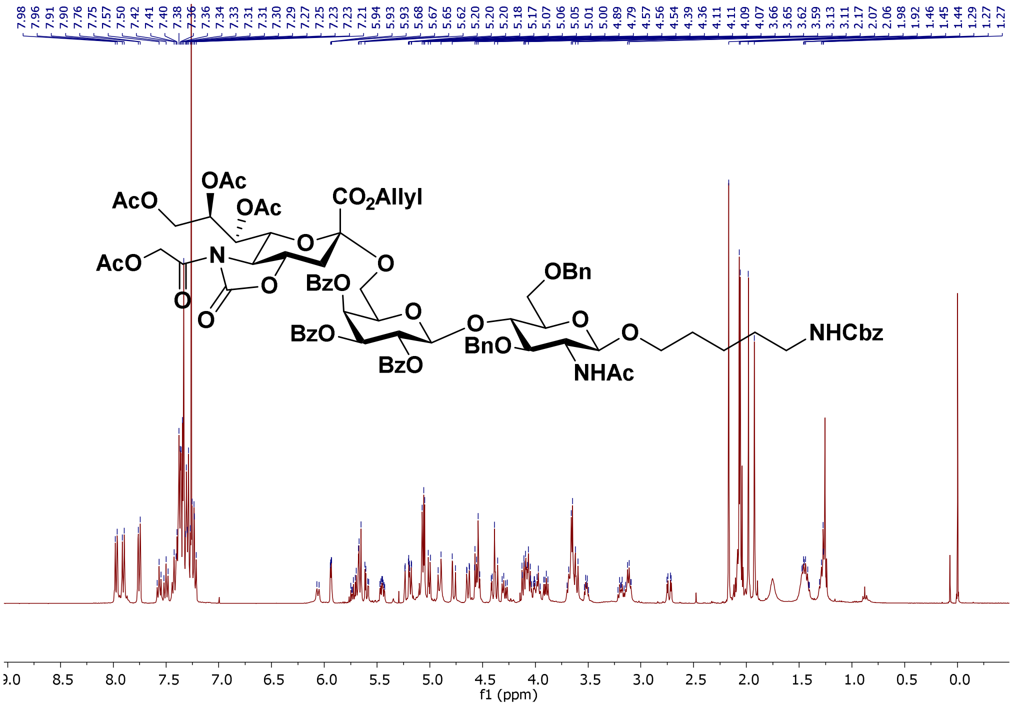

Figure S88: H1-NMR of compound 13

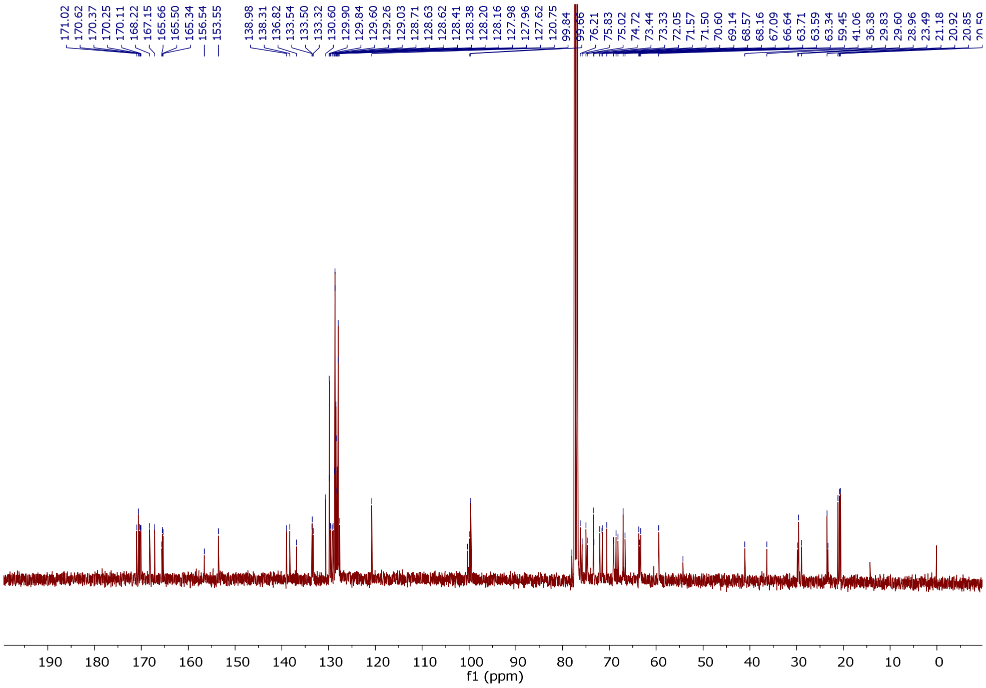

Figure S89: C13-NMR of compound 13

<u>NMR Analyses of compound 14</u>

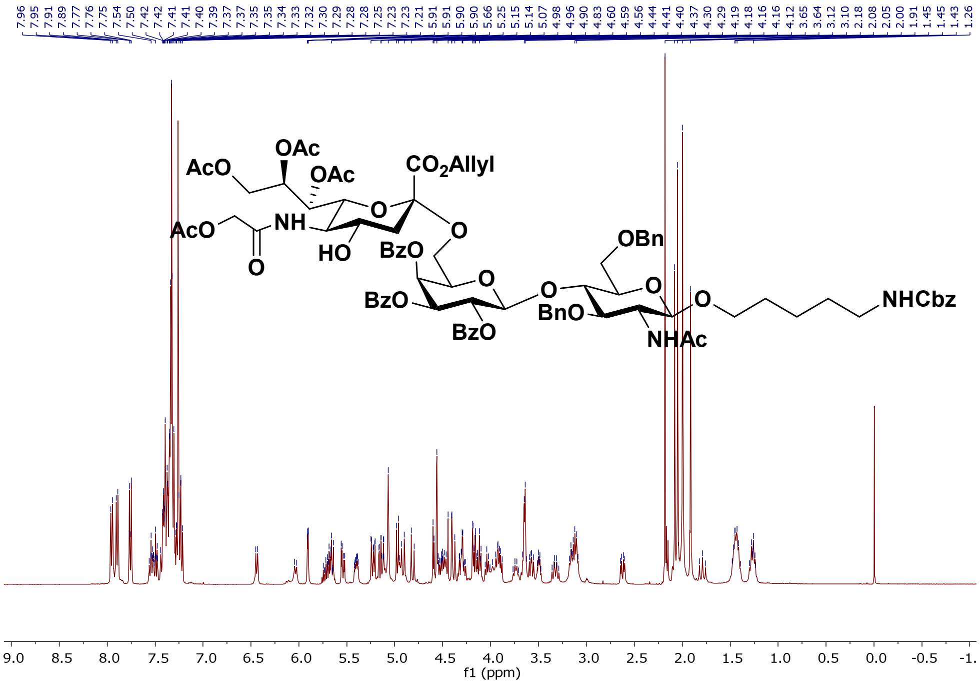

Figure S90: H1-NMR of compound 14

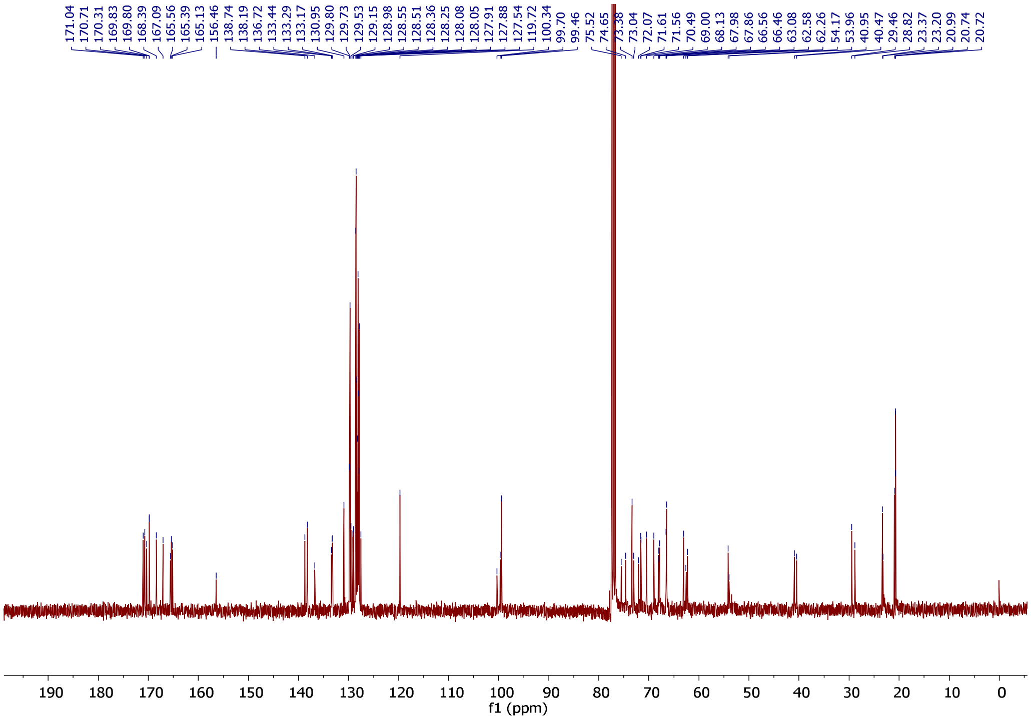

Figure S91: C13-NMR of compound 14

NMR Analyses of compound M6

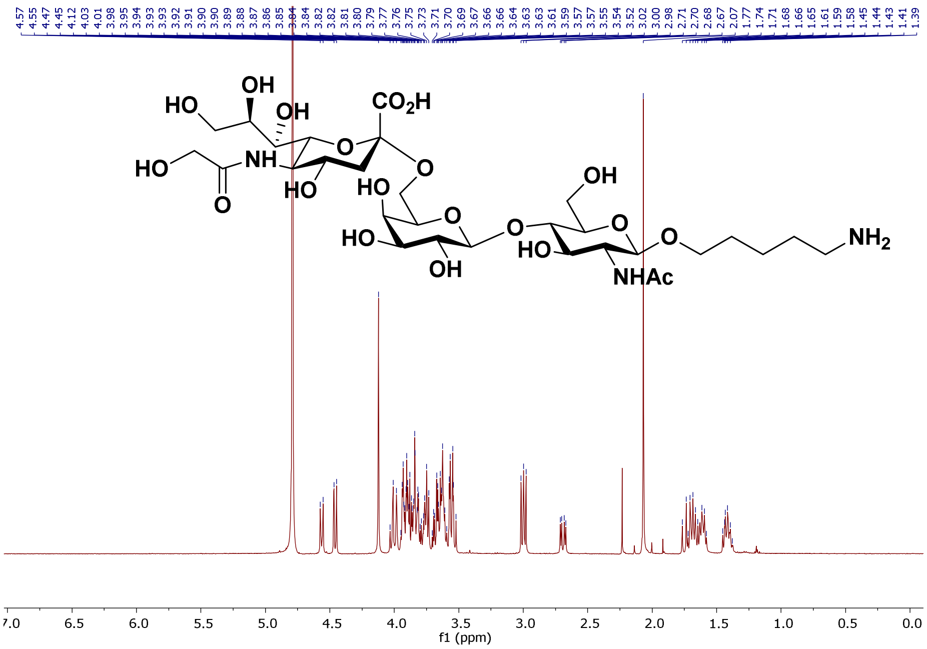

Figure S92: H1-NMR of compound M6

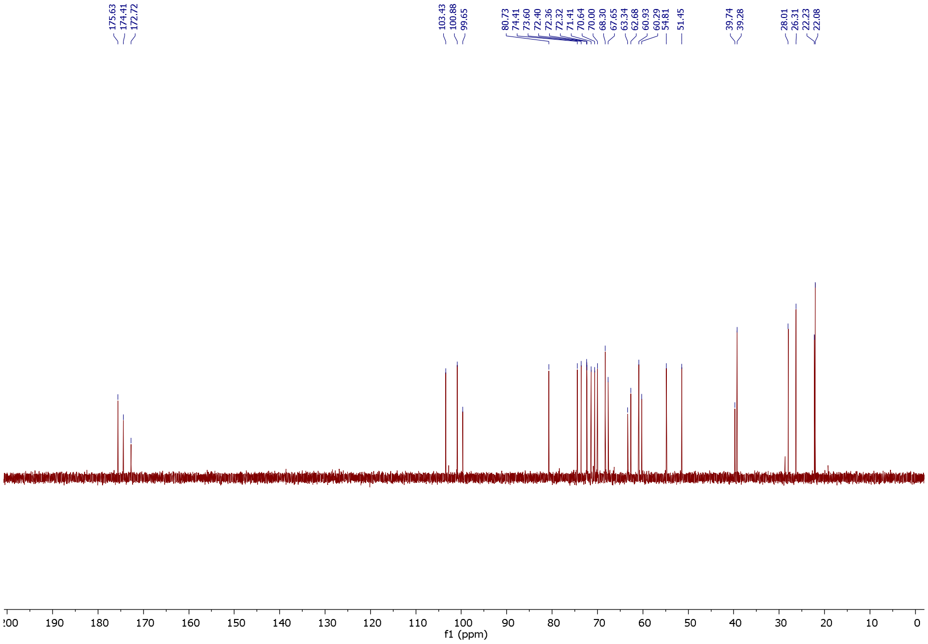

Figure S93: C13-NMR of compound M6

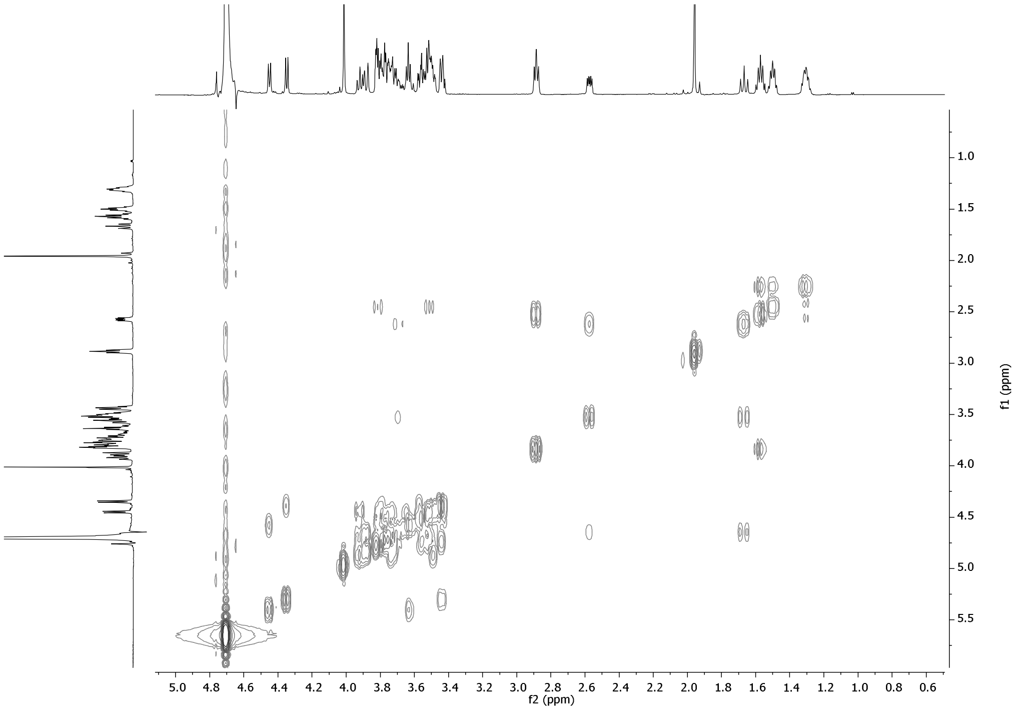

Figure S94: 1H-1H COSY NMR of compound M6

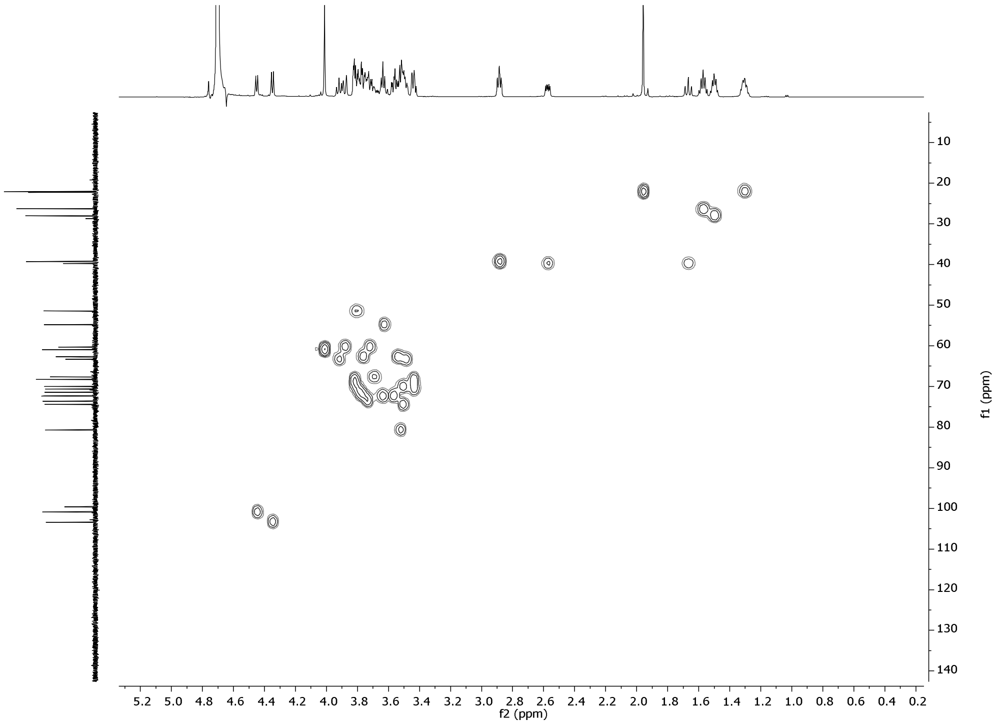

Figure S95: 1H-13C HSQC NMR of compound M6

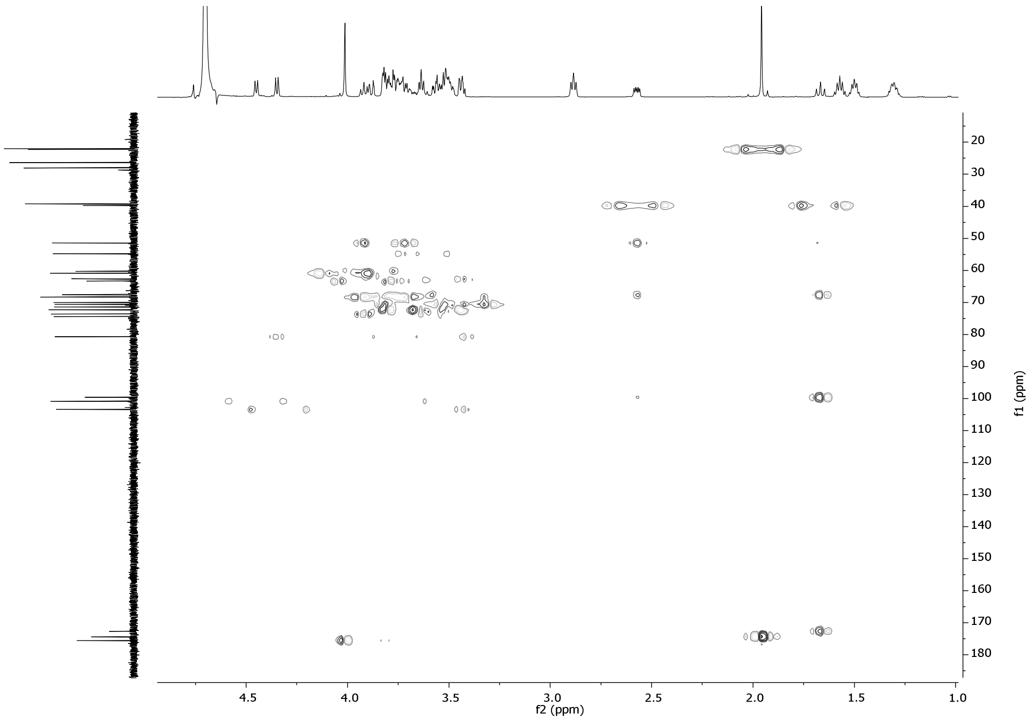

Figure S96: 1H-13C HMBC NMR of compound M6

NMR Analyses of compound 10

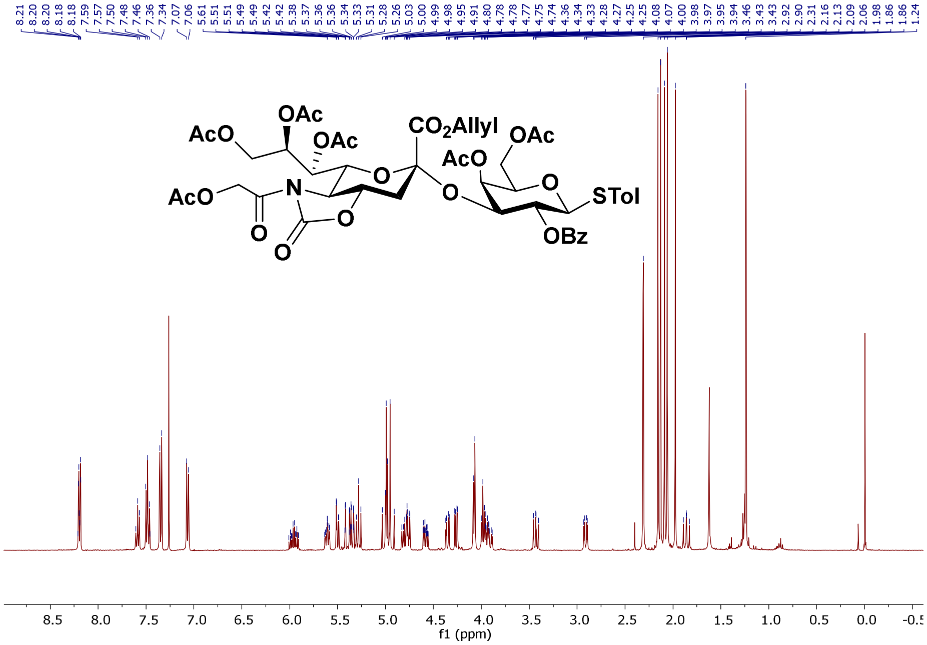

Figure S97: H1-NMR of compound 10

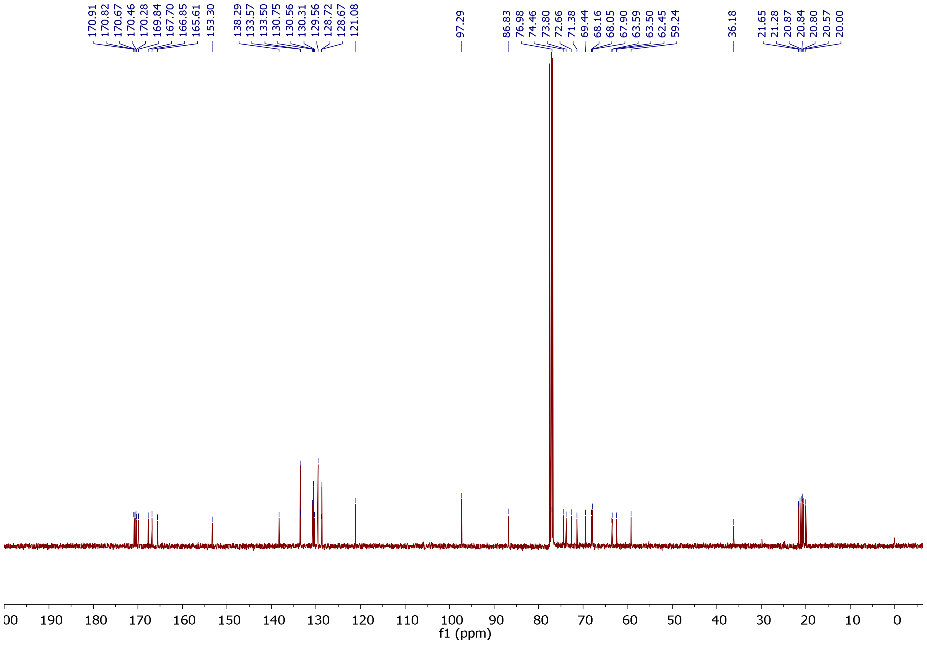

Figure S98: C13-NMR of compound 10

NMR Analyses of compound 12

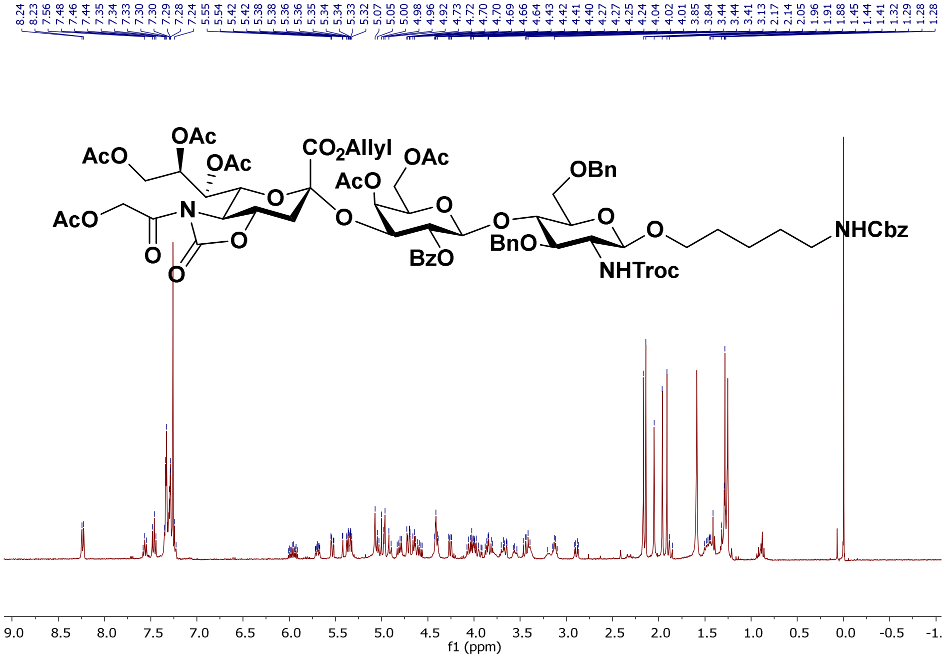

Figure S99: H1-NMR of compound 12

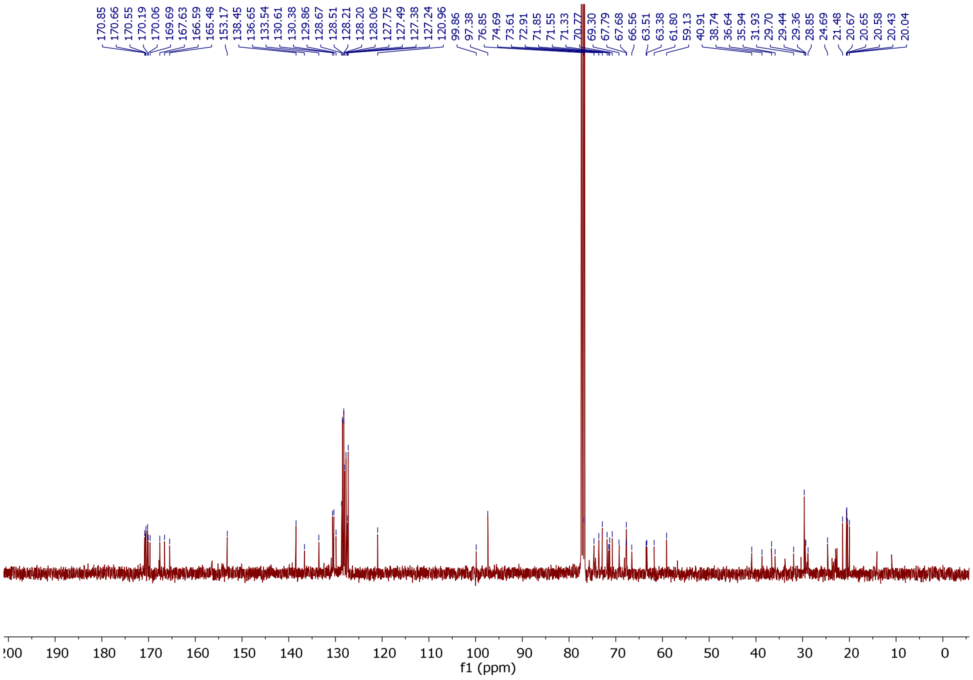

Figure S100: C13-NMR of compound 12

NMR Analyses of compound 15

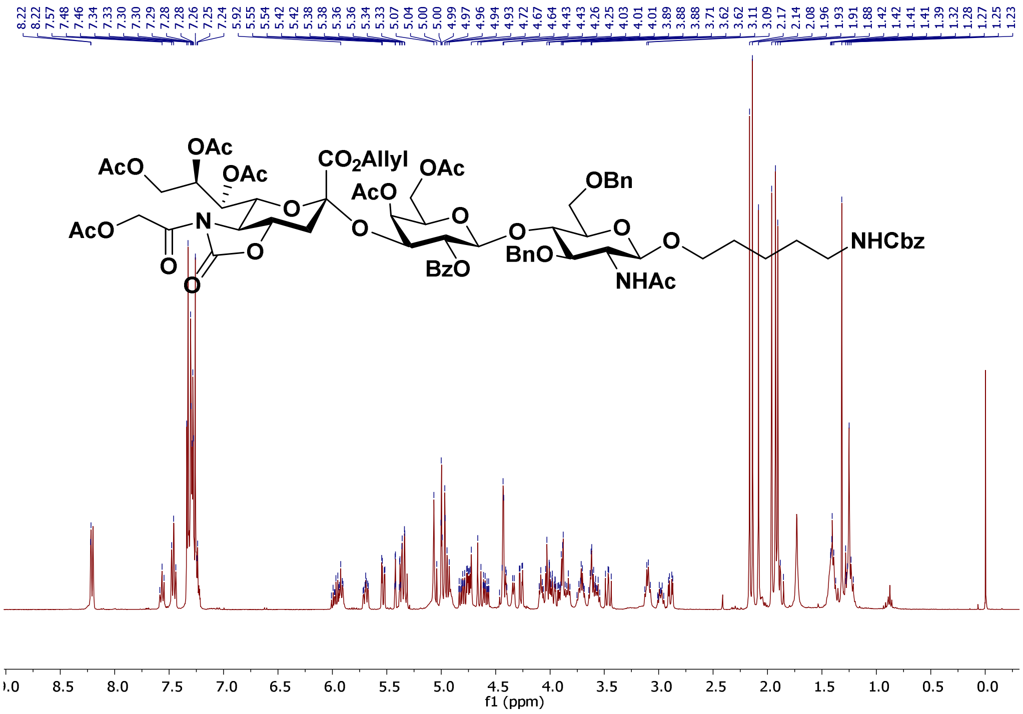

Figure S101: H1-NMR of compound 15

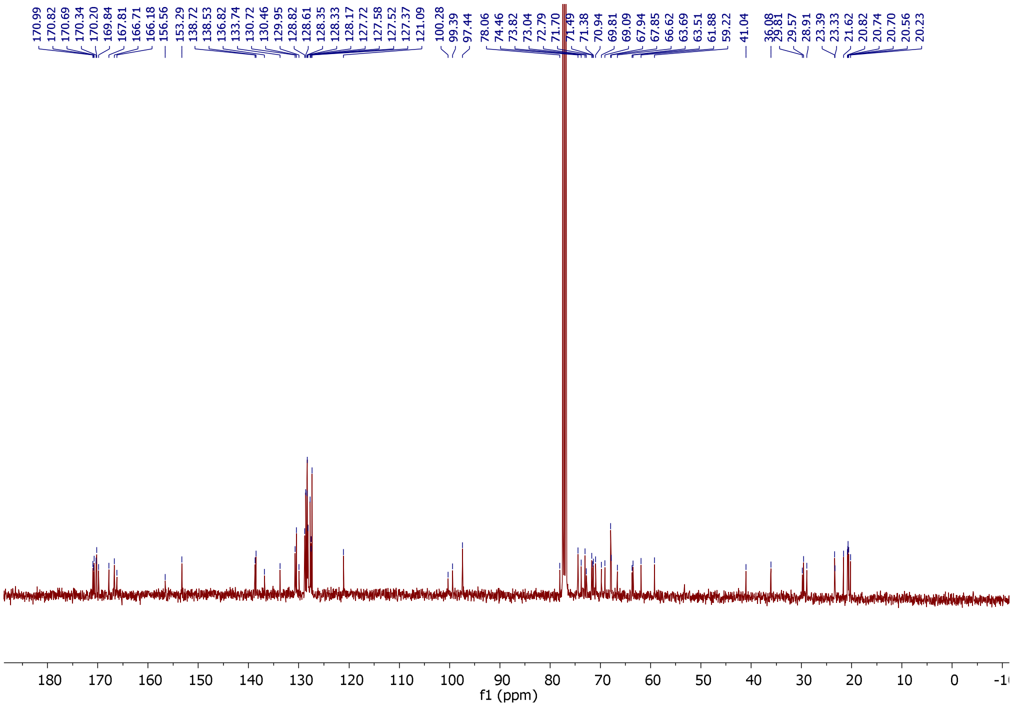

Figure S102: C13-NMR of compound 15

NMR Analyses of compound 16

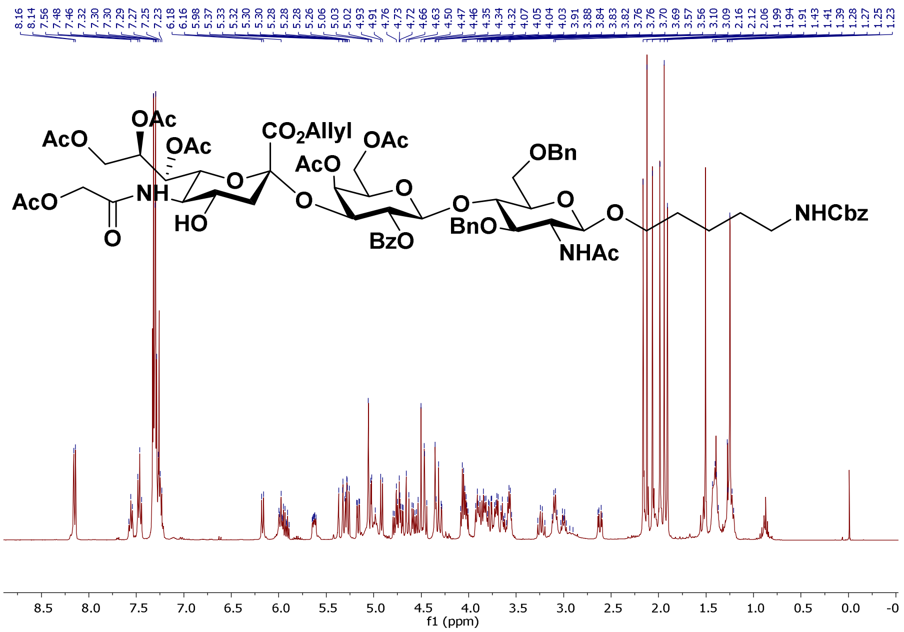

Figure S103: H1-NMR of compound 16

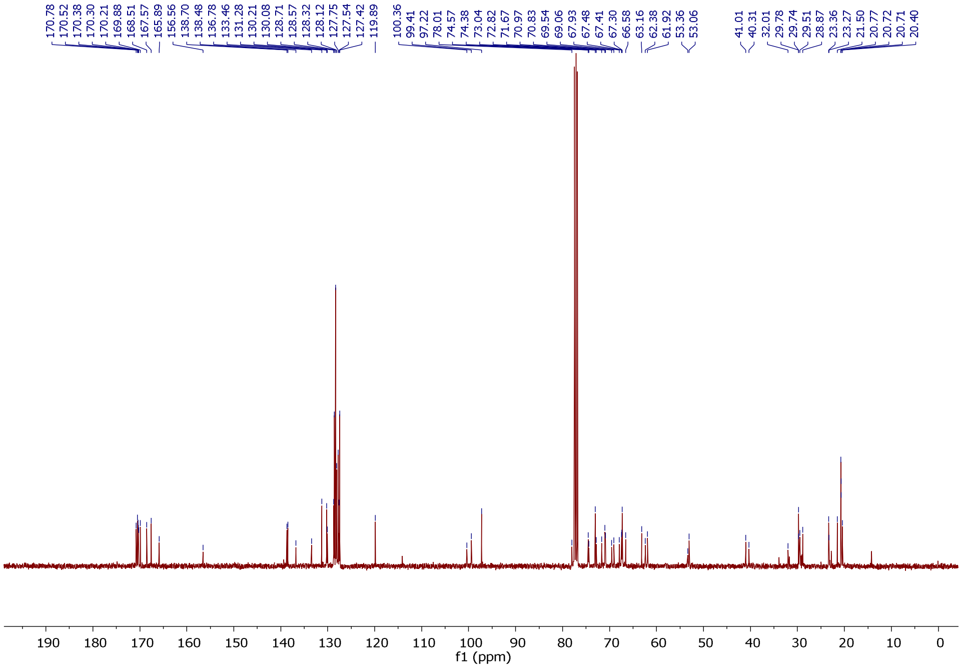

Figure S104: C13-NMR of compound 16

<u>NMR Analyses of compound M3</u>

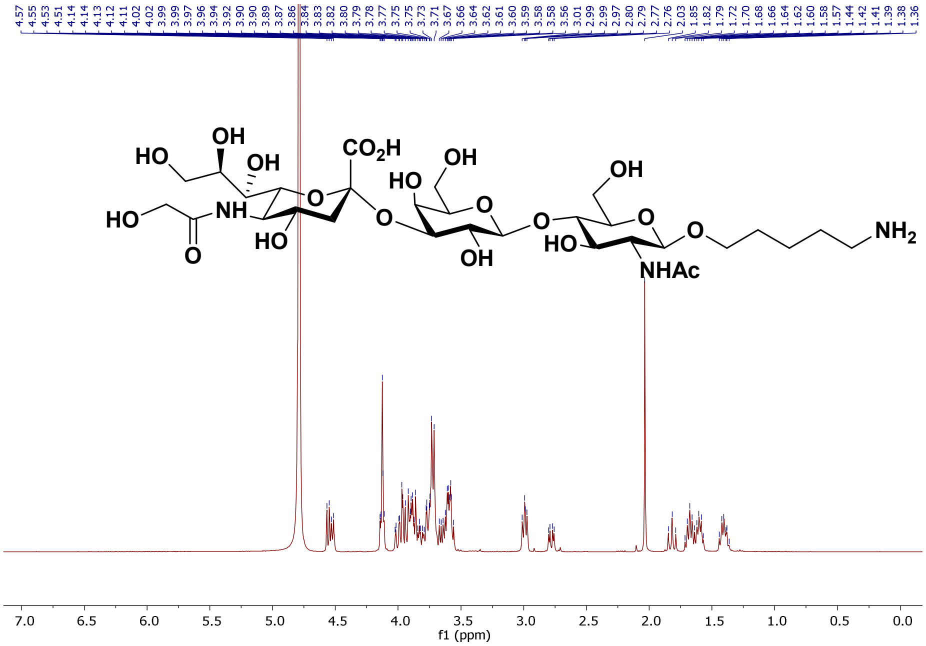

Figure S105: H1-NMR of compound M3

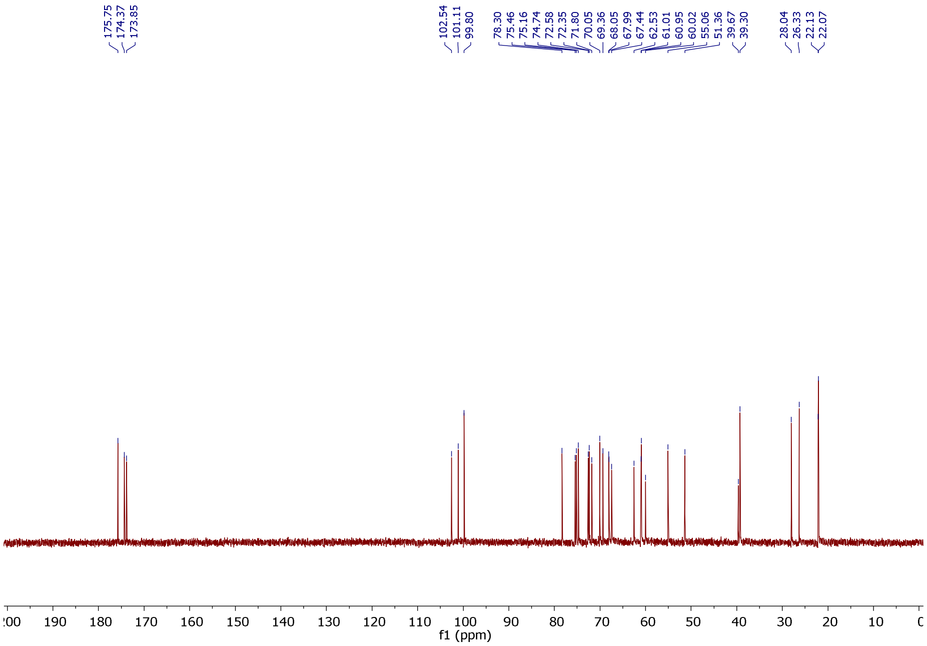

Figure S106: C13-NMR of compound M3

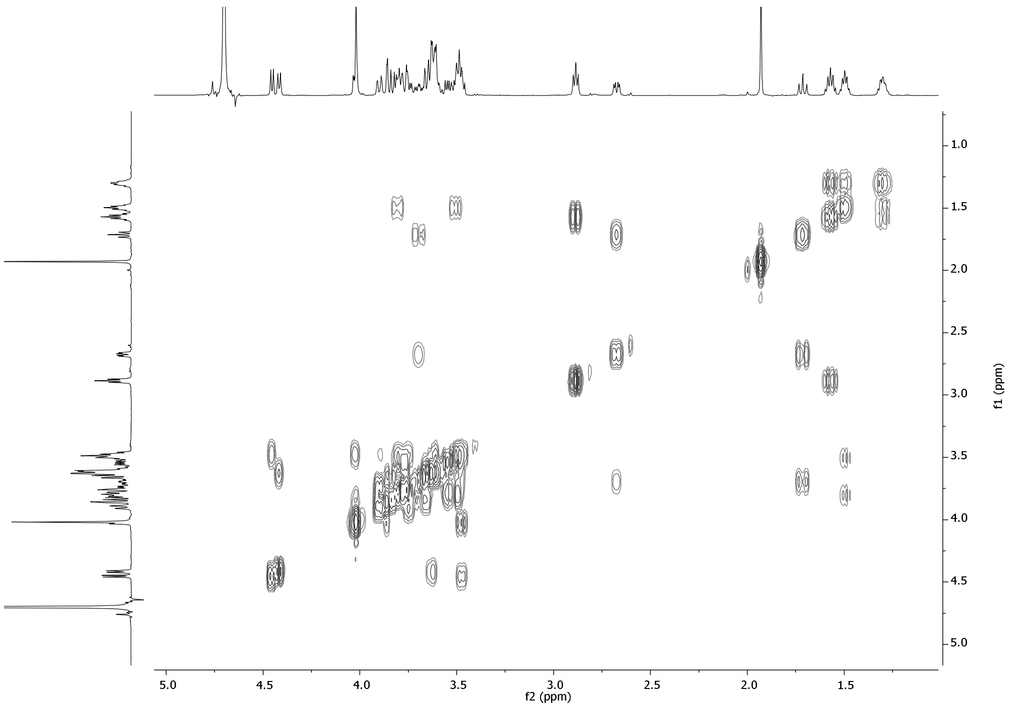

Figure S107: 1H-1H COSY NMR of compound M3

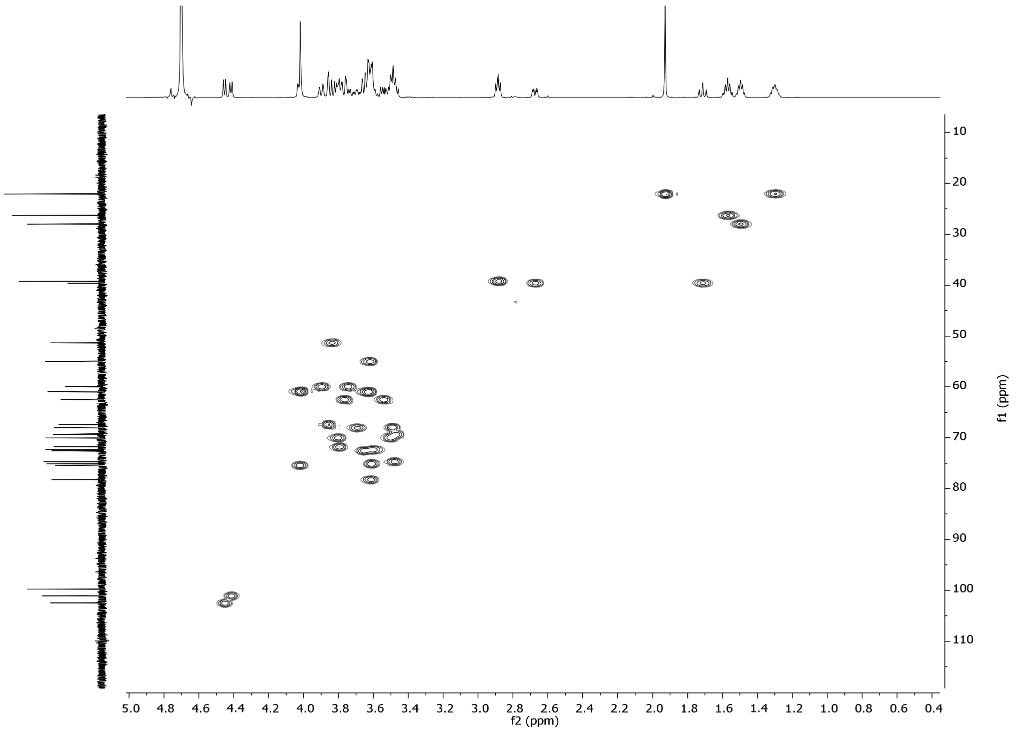

Figure S108: 1H-13C HSQC NMR of compound M3

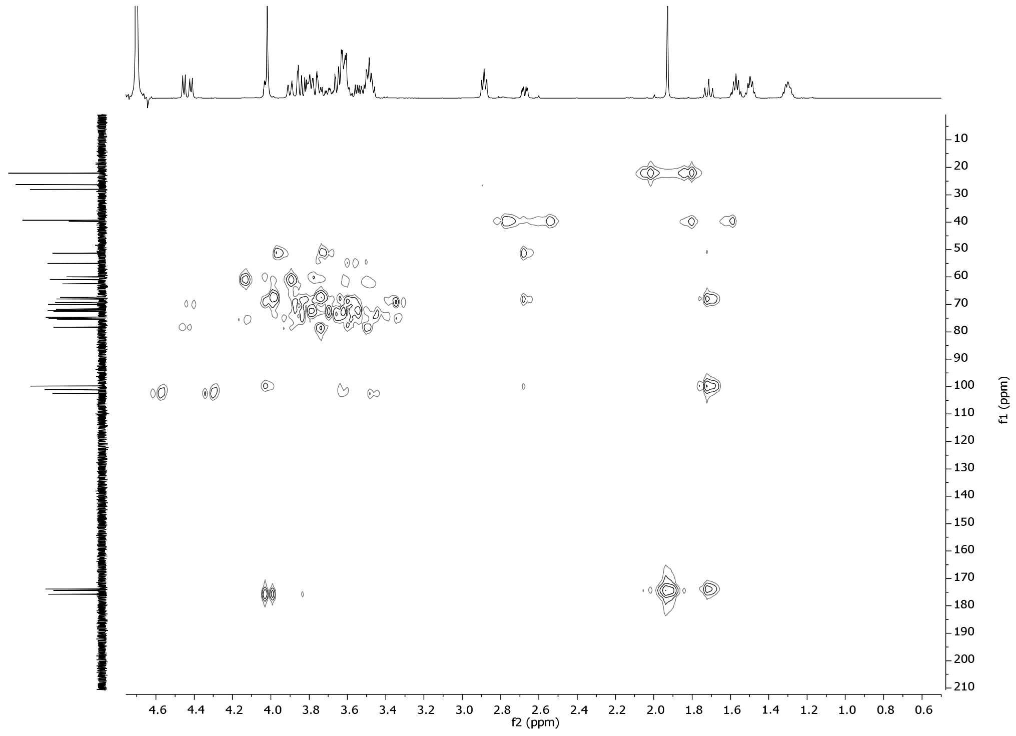

Figure S109: 1H-13C HMBC NMR of compound M3

NMR Analyses of compound 17

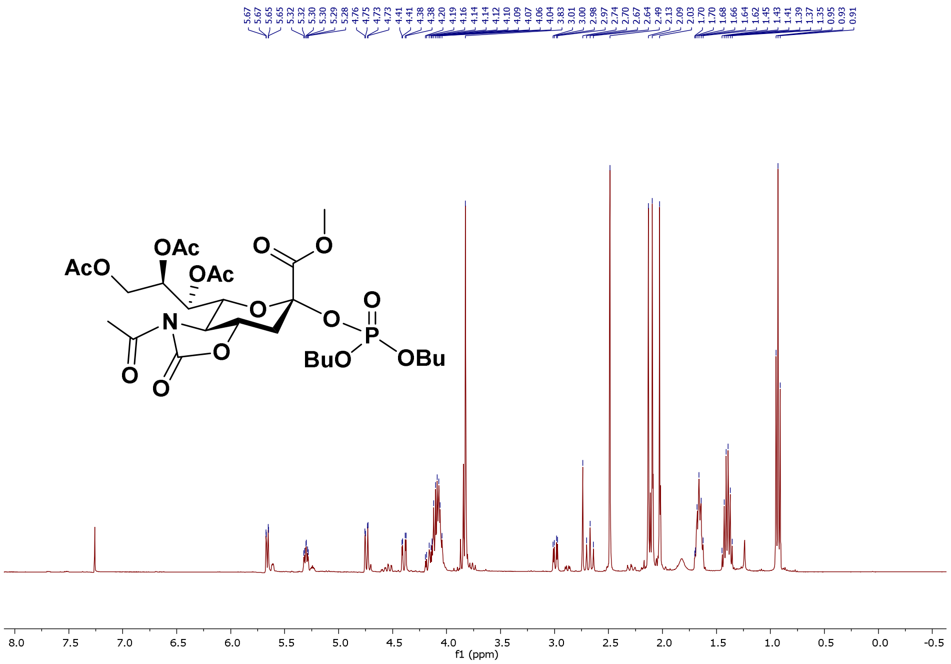

Figure S110: H1-NMR of compound 17

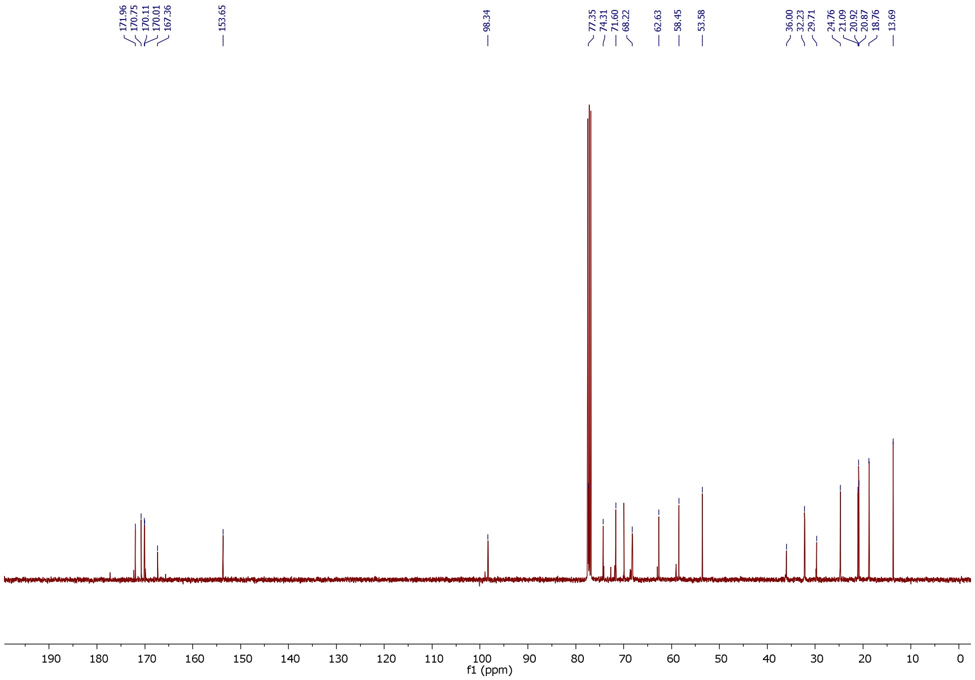

Figure S111: C13-NMR of compound 17

NMR Analyses of compound 18

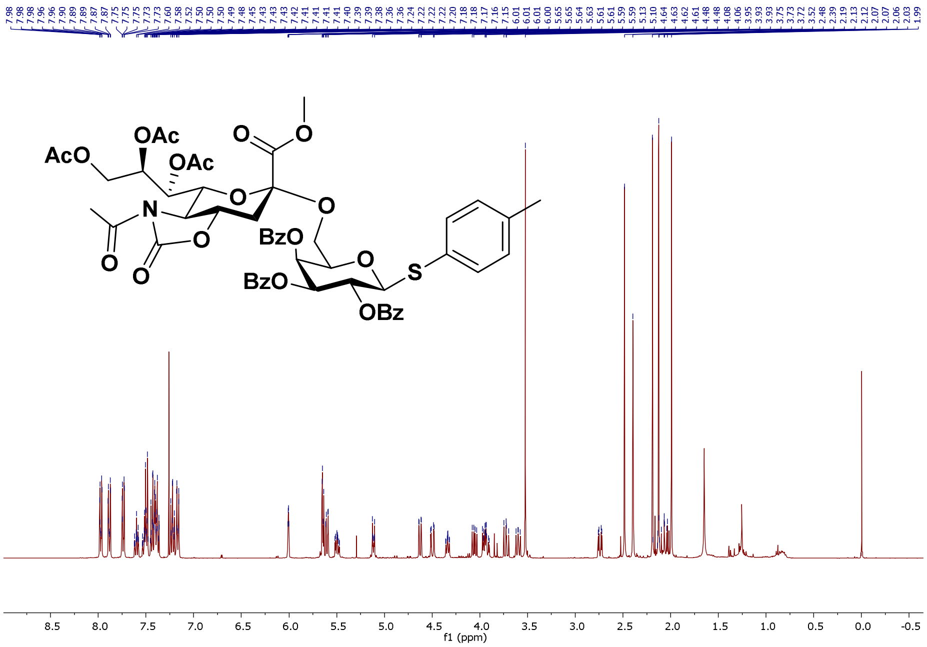

Figure S112: H1-NMR of compound 18

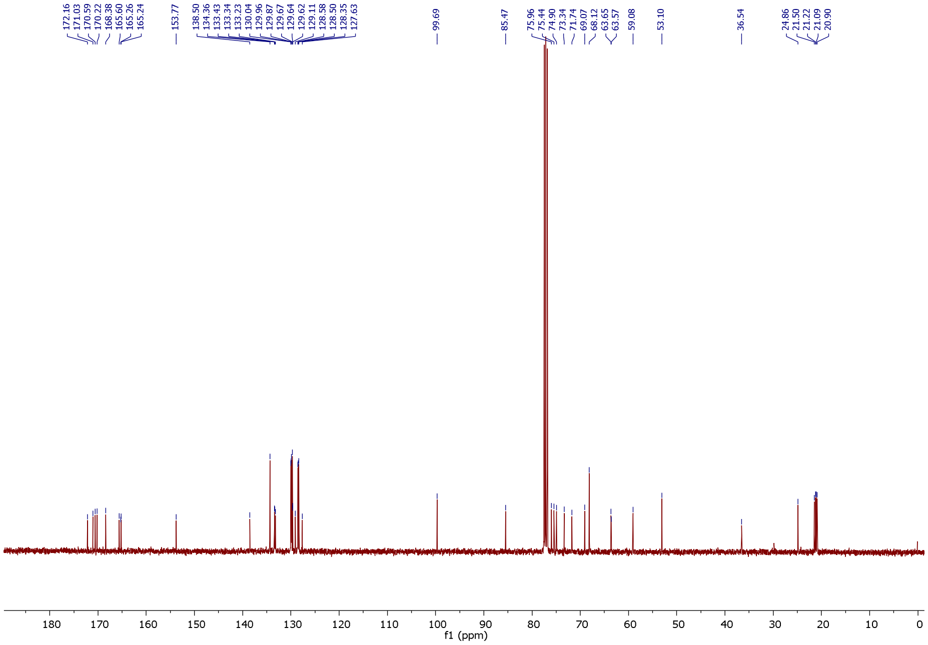

Figure S113: C13-NMR of compound 18

NMR Analyses of compound 19

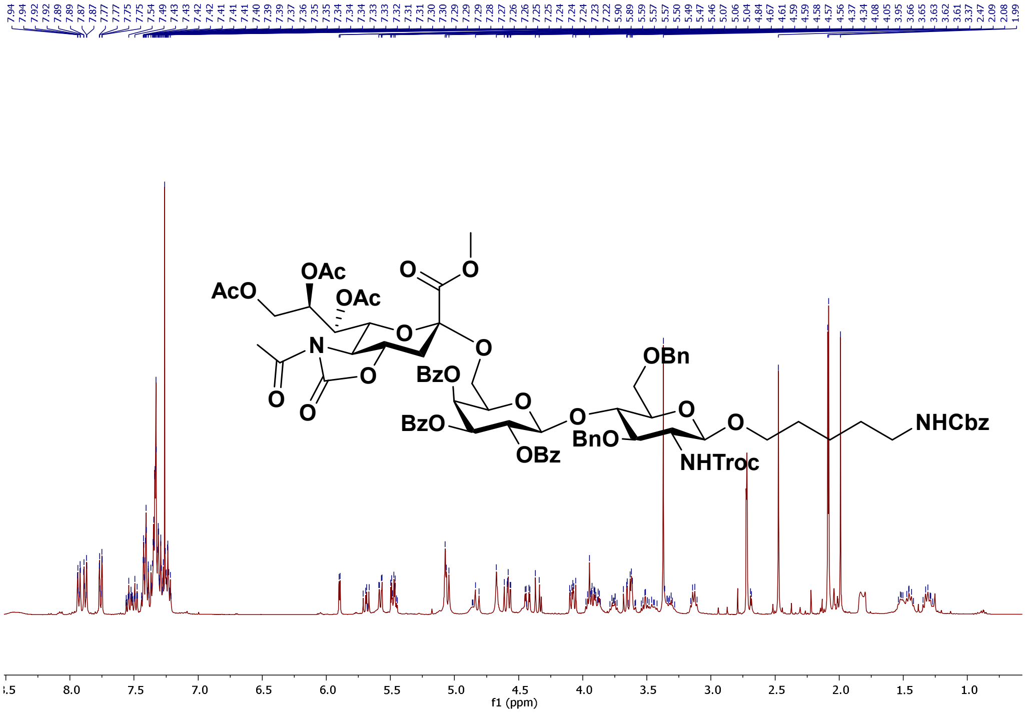

Figure S114: H1-NMR of compound 19

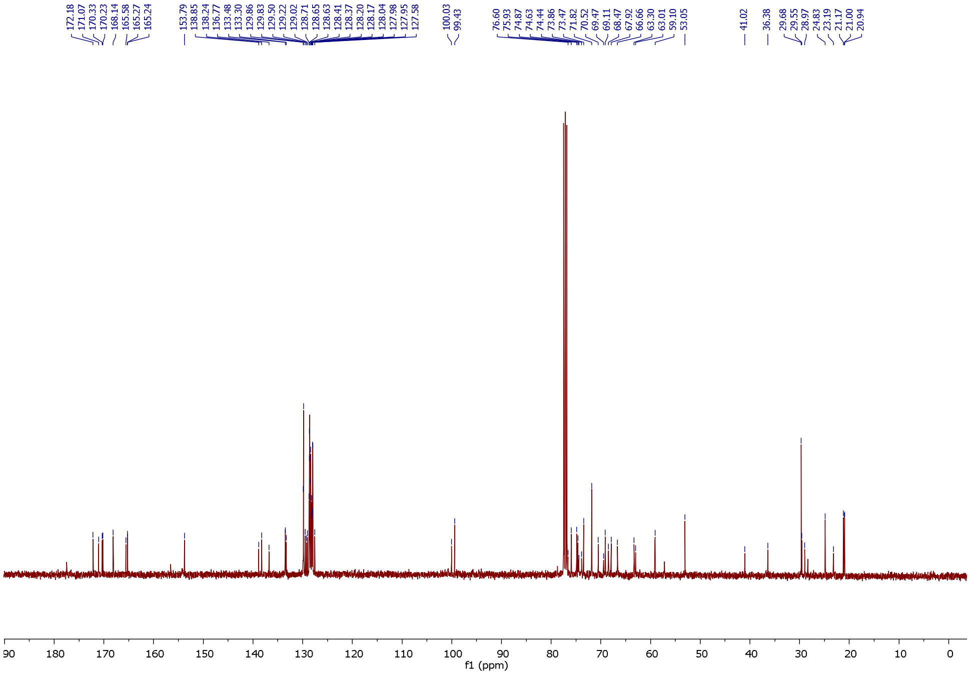

Figure S115: C13-NMR of compound 19

NMR Analyses of compound 20

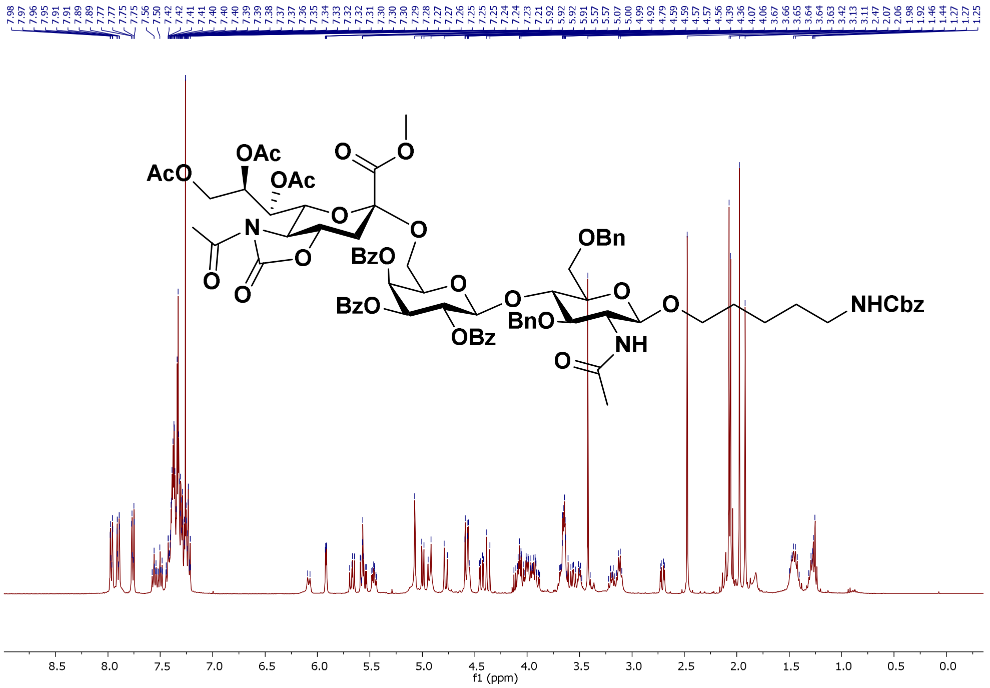

Figure S116: H1-NMR of compound 20

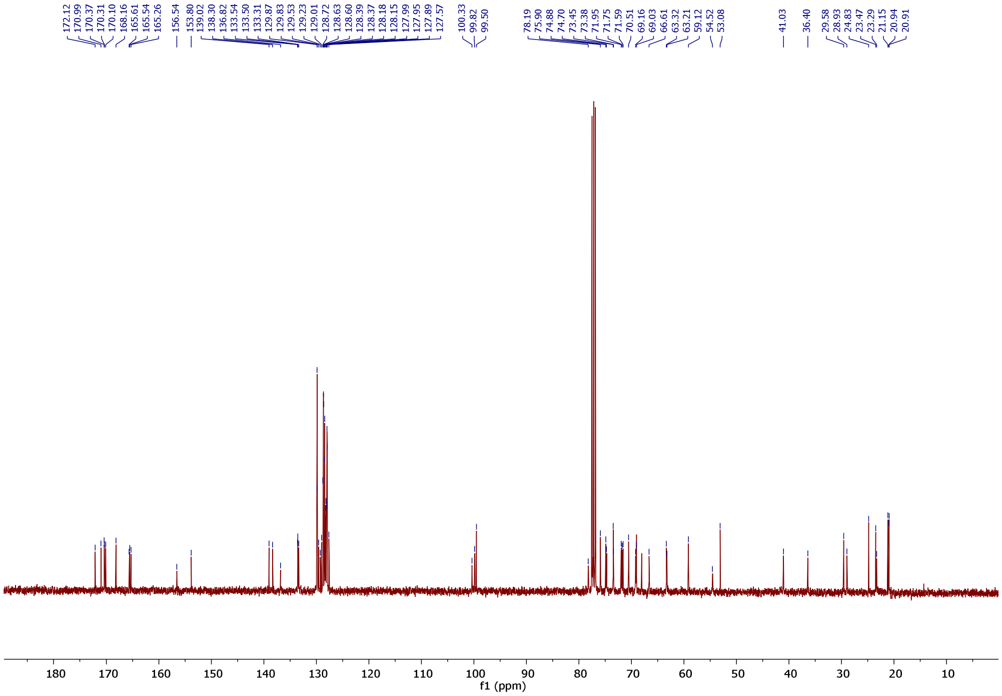

Figure S117: C13-NMR of compound 20

NMR Analyses of compound 21

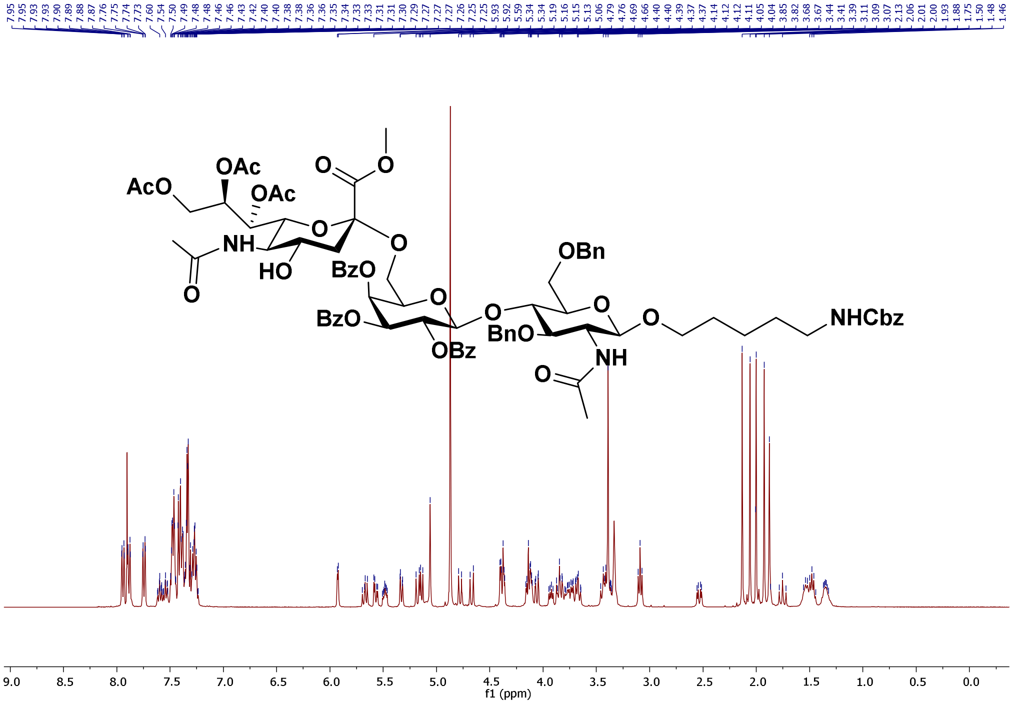

Figure S118: H1-NMR of compound 21

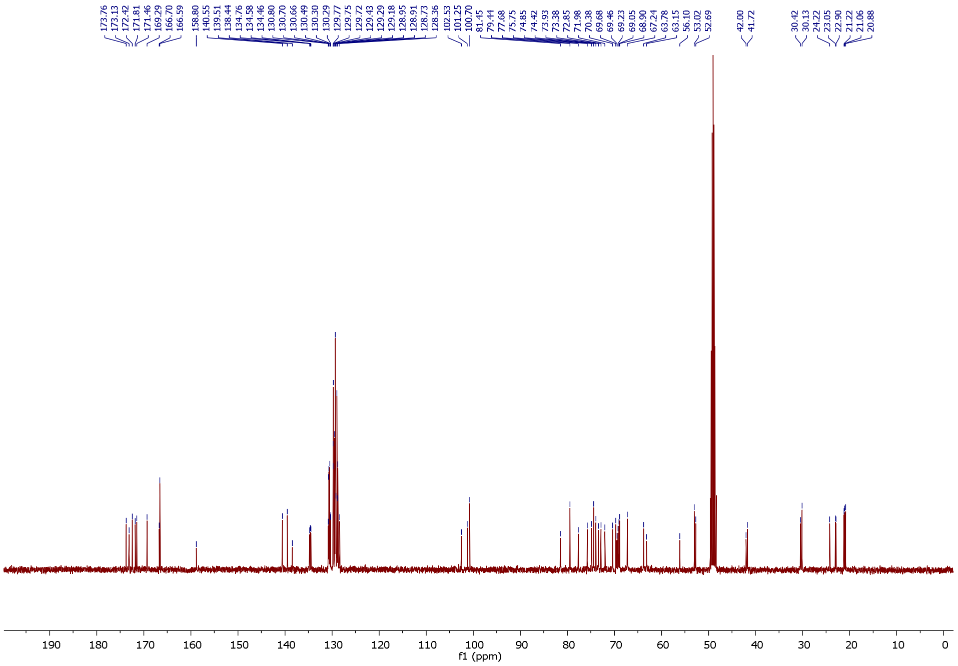

Figure S119: C13-NMR of compound 21

NMR Analyses of compound H6

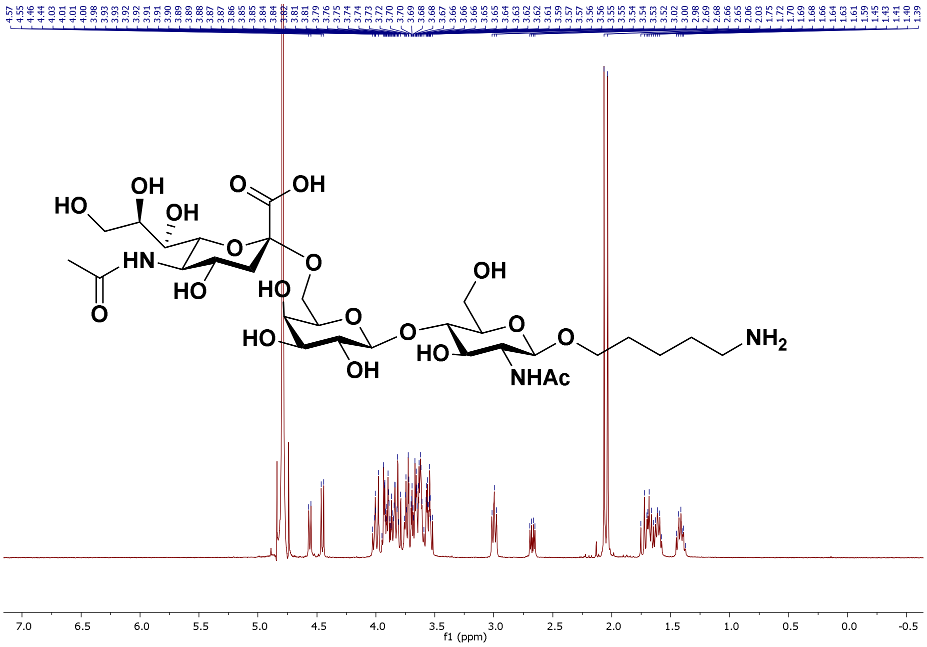

Figure S120: H1-NMR of compound H6

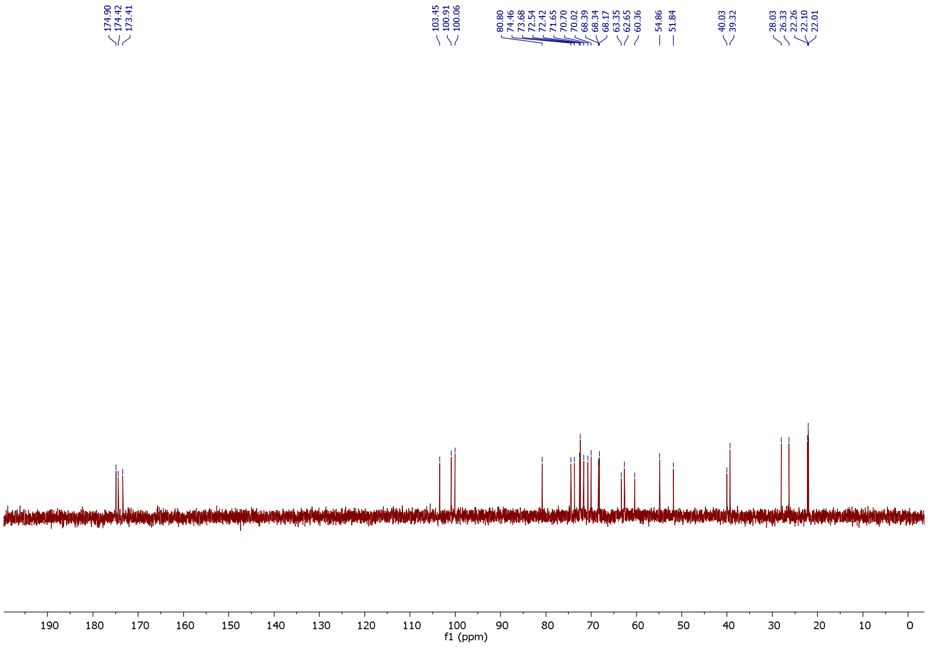

Figure S121: C13-NMR of compound H6

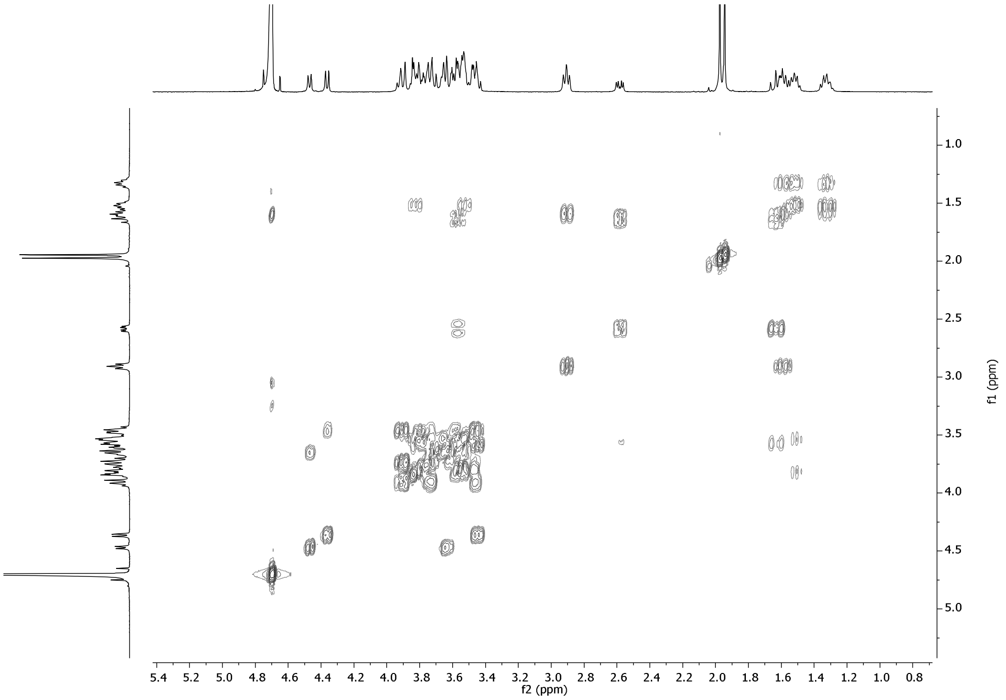

Figure S122: 1H-1H COSY NMR of compound H6

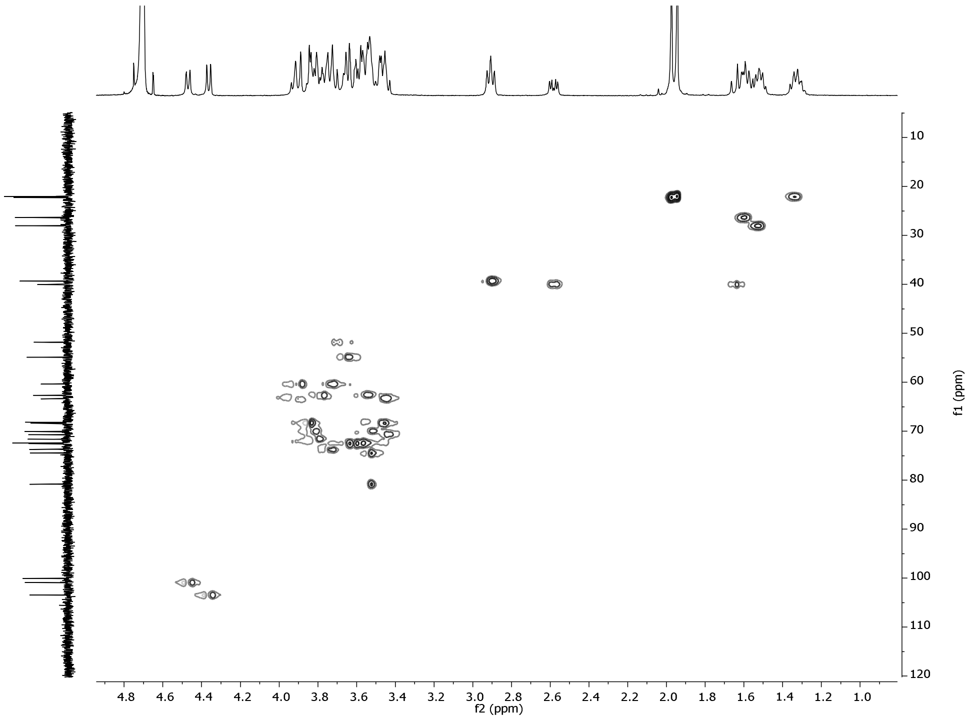

Figure S123: 1H-13C HSQC NMR of compound H6

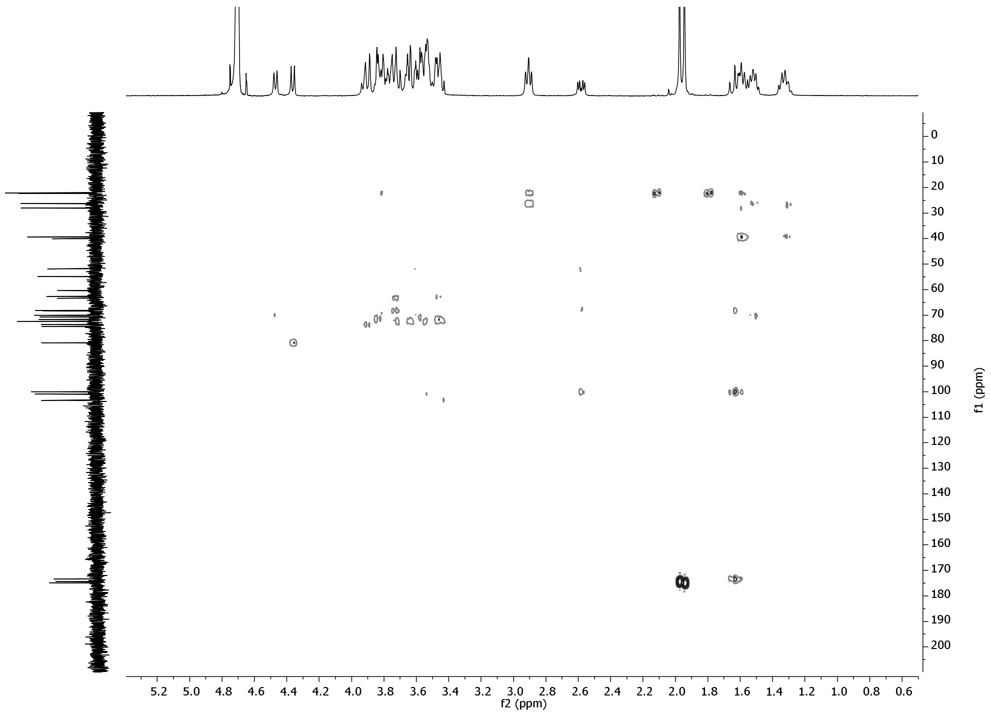

Figure S124: 1H-13C HMBC NMR of compound H6

NMR Analyses of compound 22

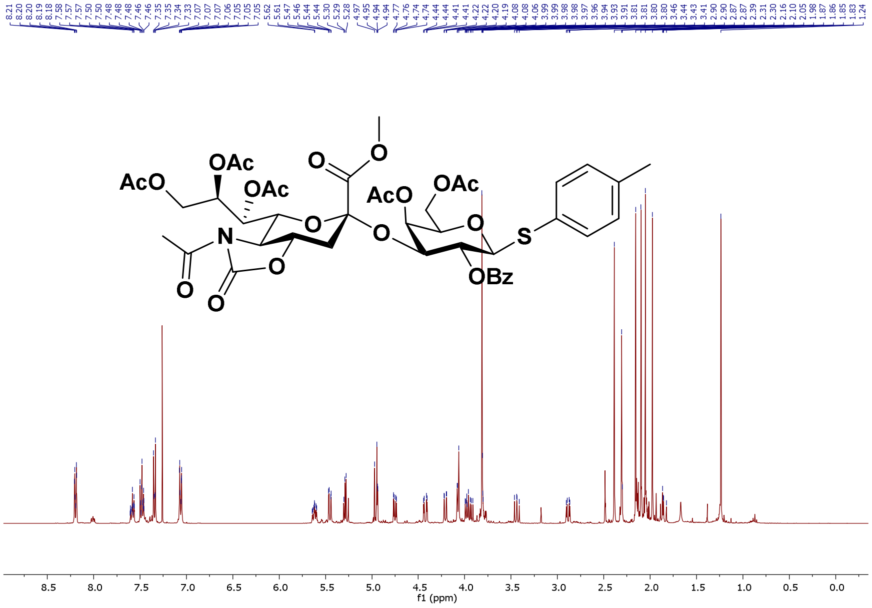

Figure S125: H1-NMR of compound 22

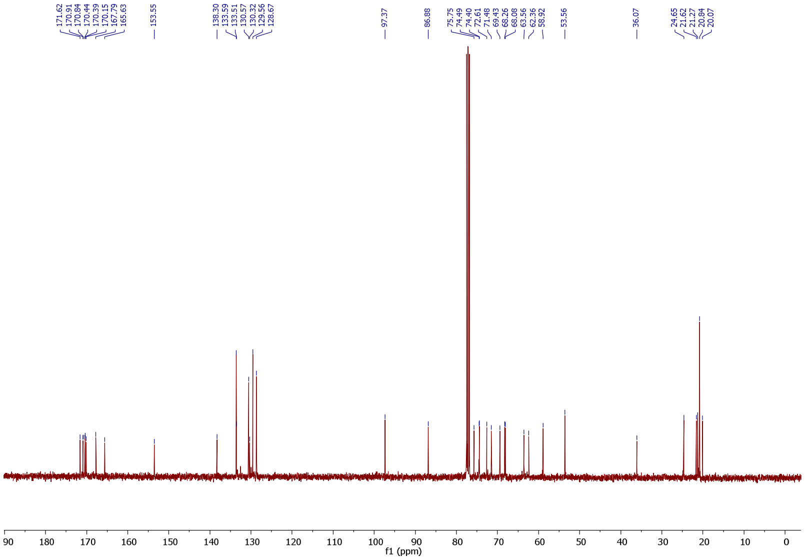

Figure S126: C13-NMR of compound 22

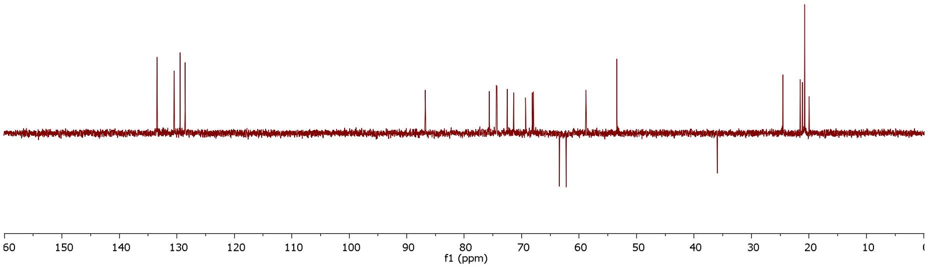

Figure S127: DEPT-NMR of compound 22

NMR Analyses of compound 23

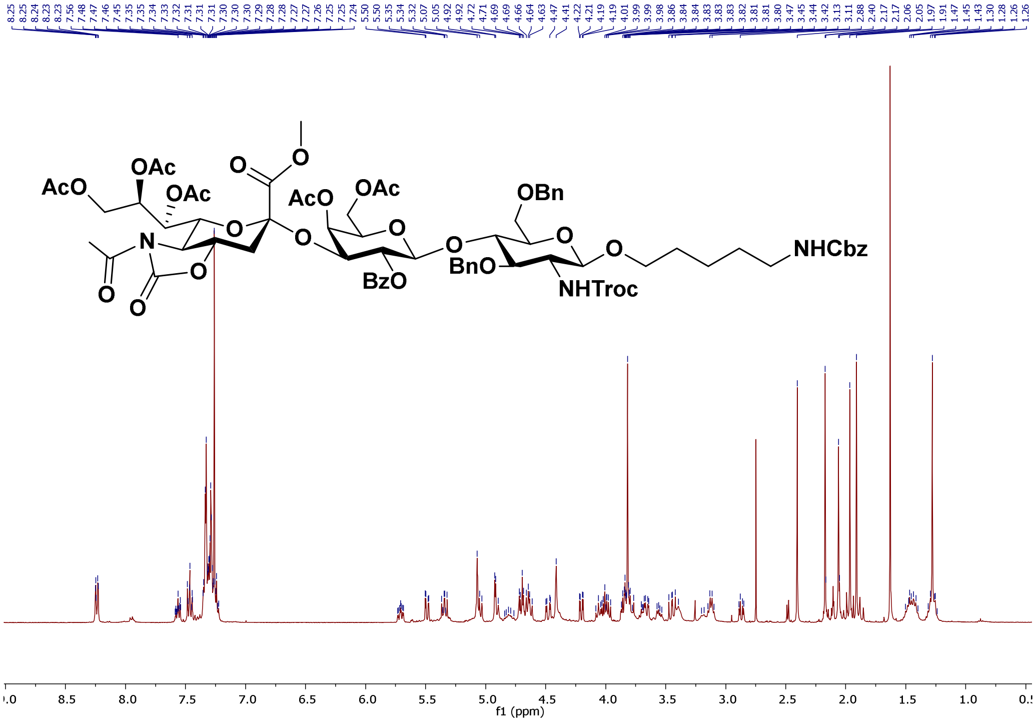

Figure S128: H1-NMR of compound 23

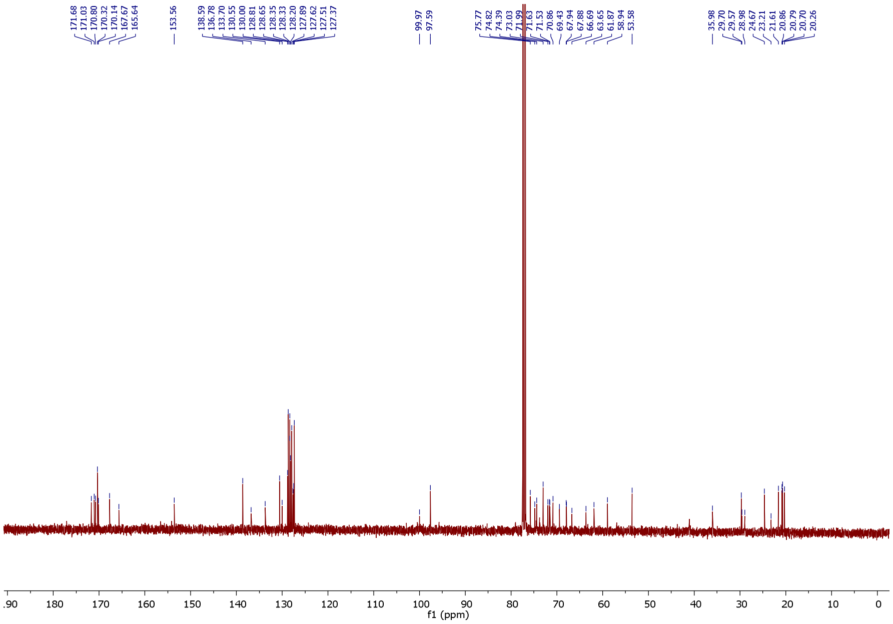

Figure S129: C13-NMR of compound 23

NMR Analyses of compound 24

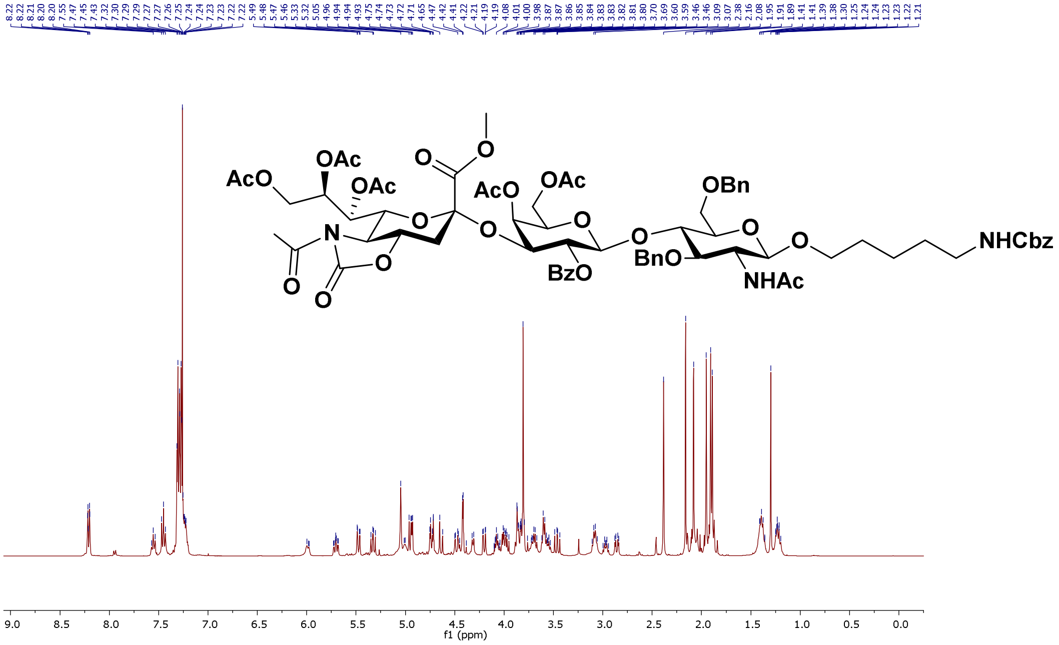

Figure S130: H1-NMR of compound 24

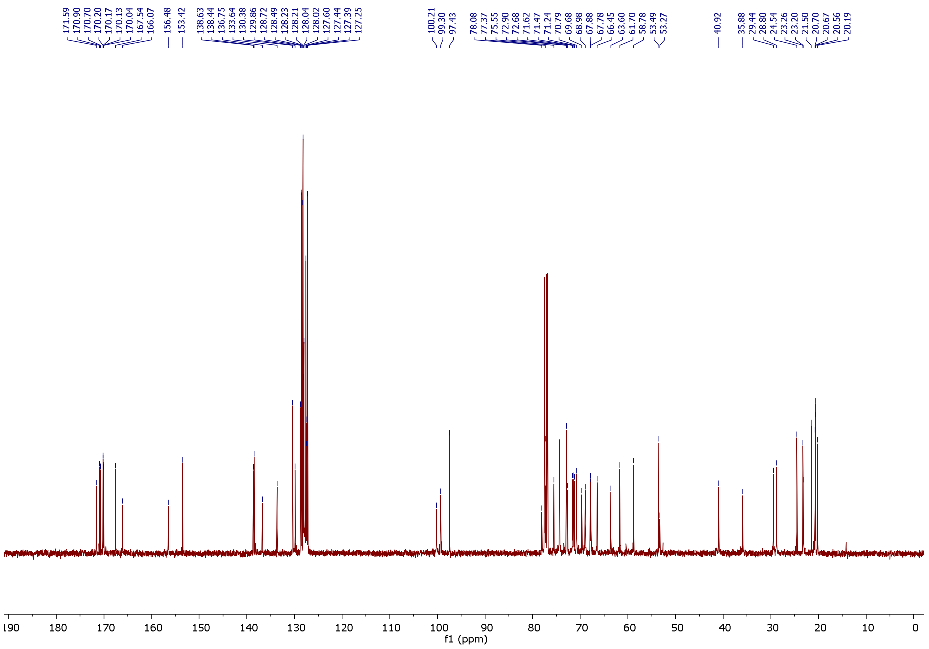

Figure S131: C13-NMR of compound 24

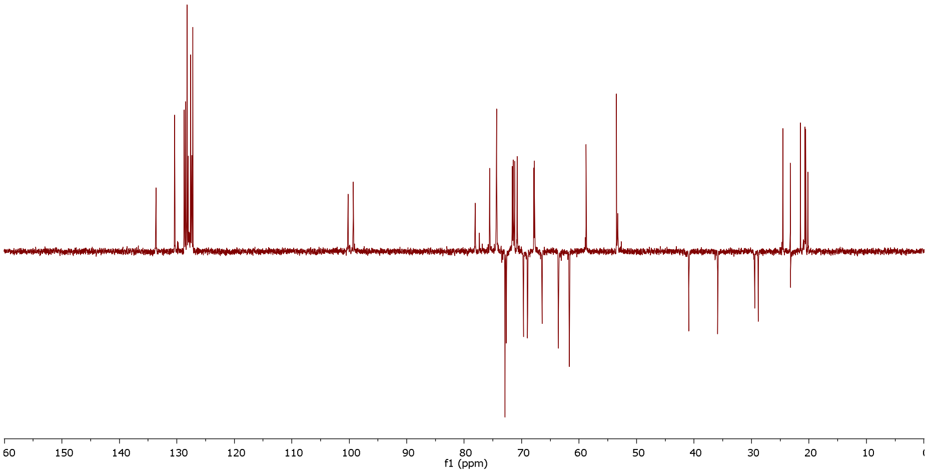

Figure S132: DEPT-NMR of compound 24

NMR Analyses of compound 25

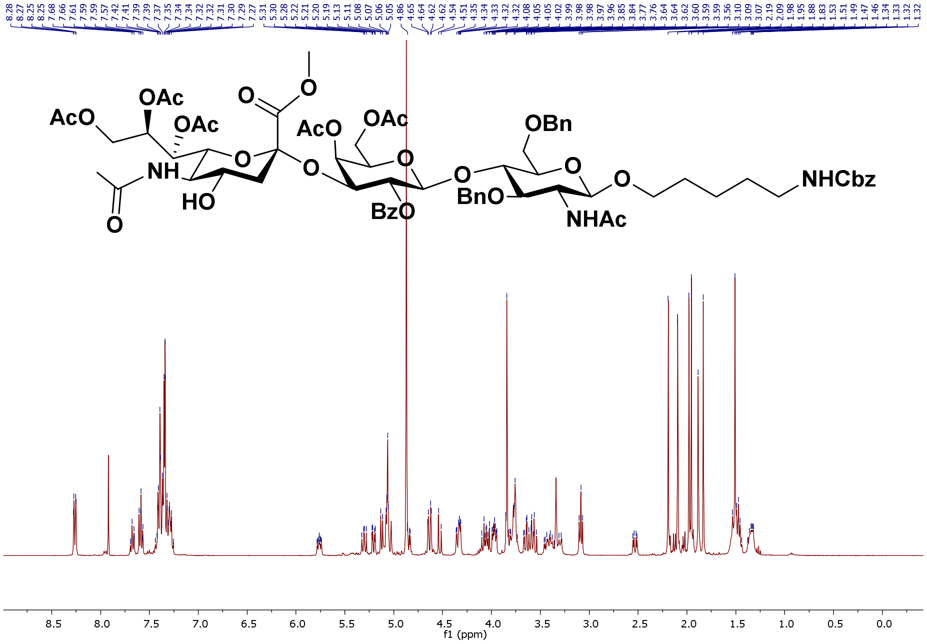

Figure S133: H1-NMR of compound 25

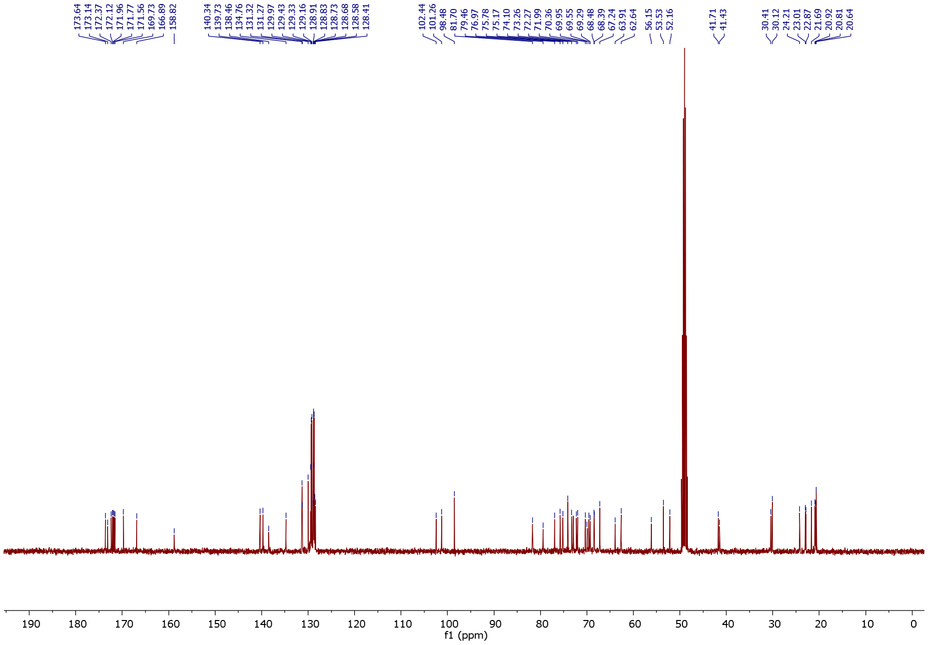

Figure S134: C13-NMR of compound 25

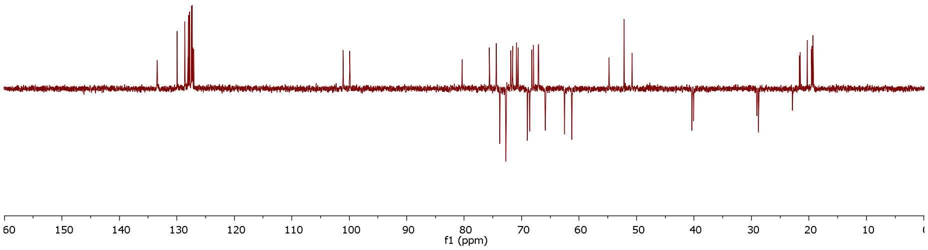

Figure S135: DEPT-NMR of compound 25

NMR Analyses of compound H3

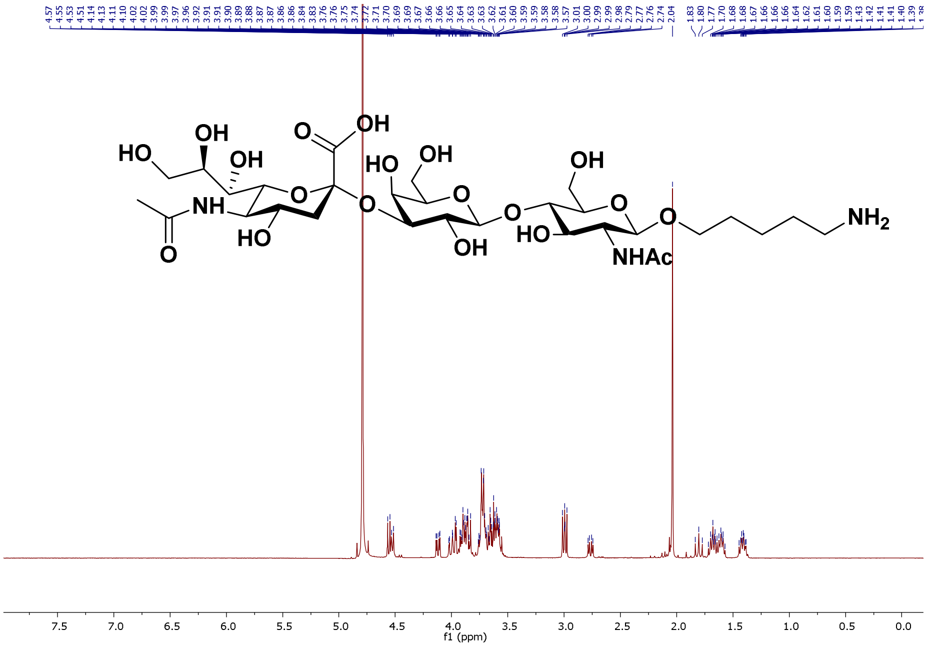

Figure S136: H1-NMR of compound H3

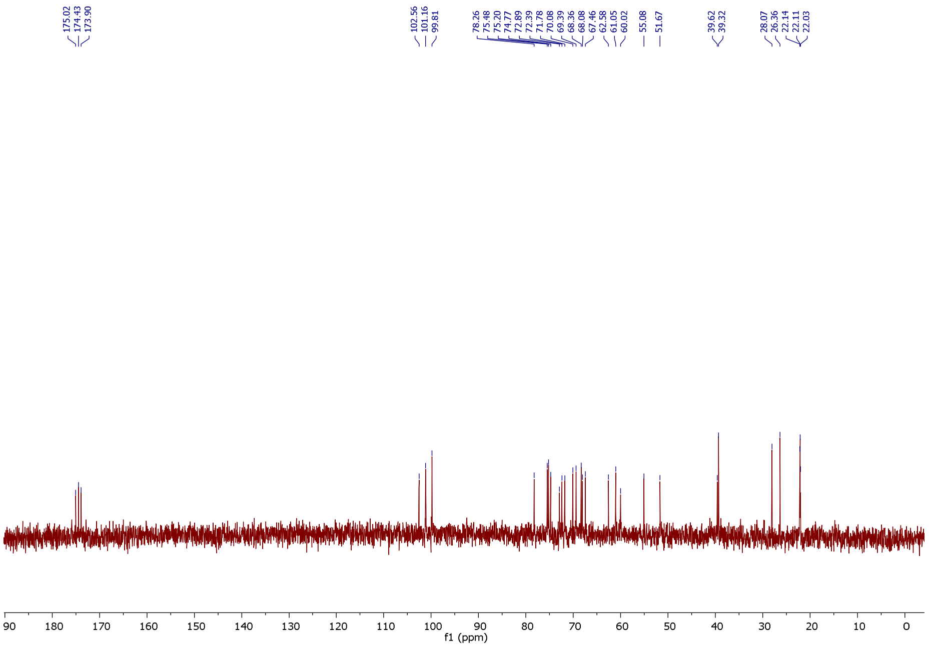

Figure S137: C13-NMR of compound H3.

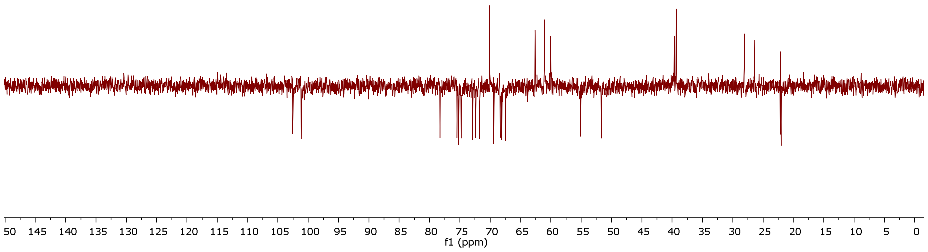

Figure S138: DEPT-NMR of compound H3

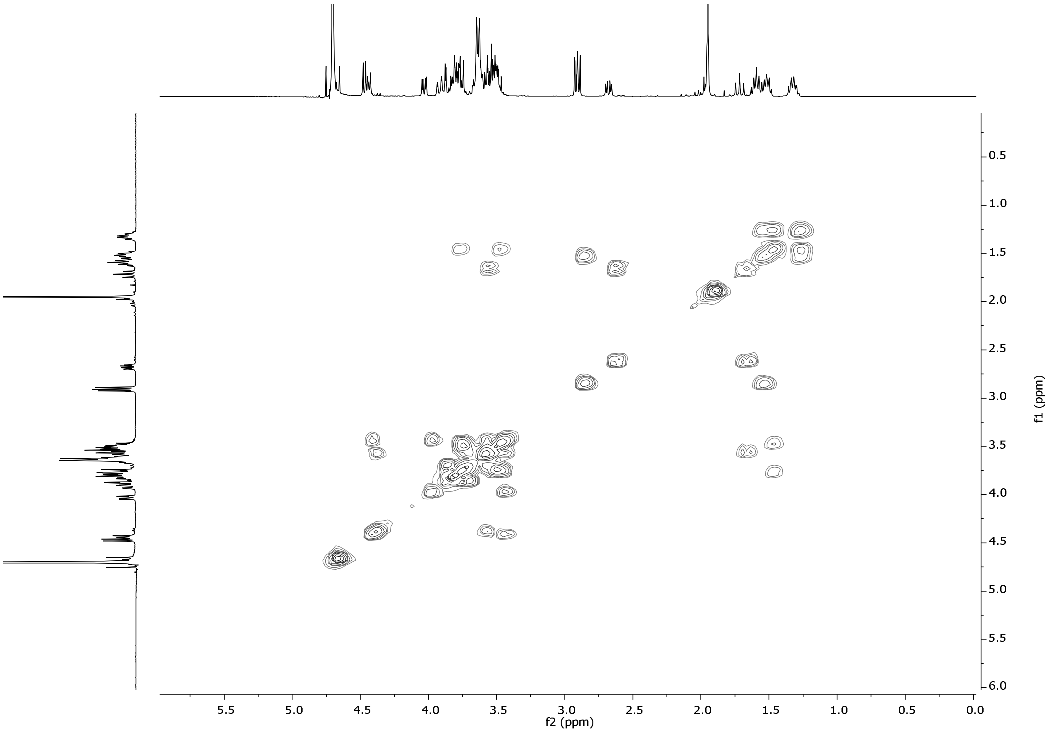

Figure S139: 1H-1H COSY NMR of compound H3

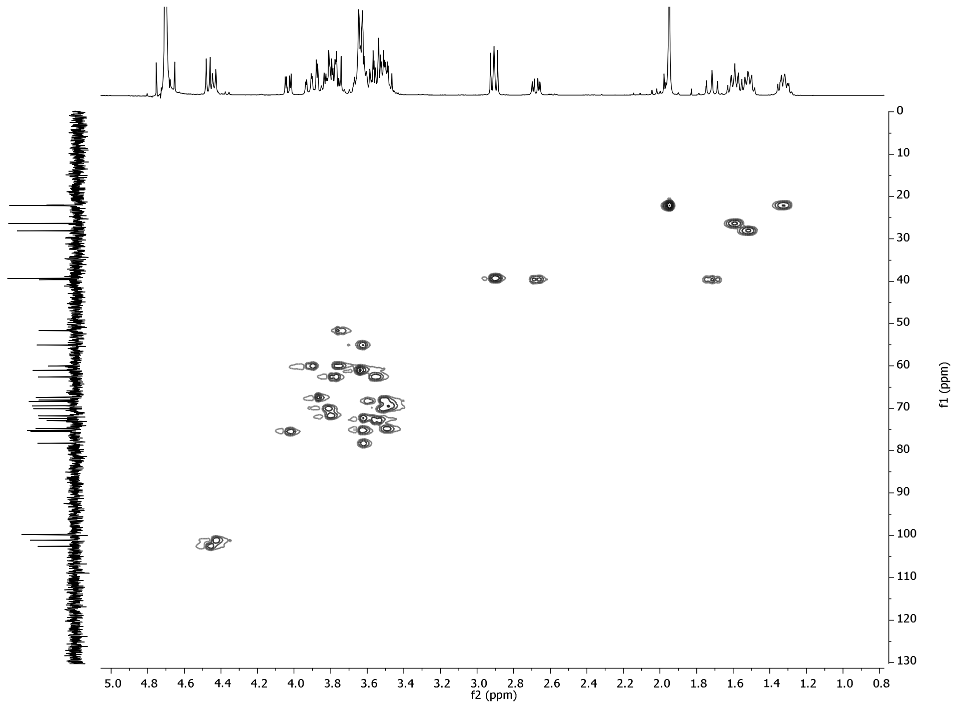

Figure S140: 1H-13C HSQC NMR of compound H3

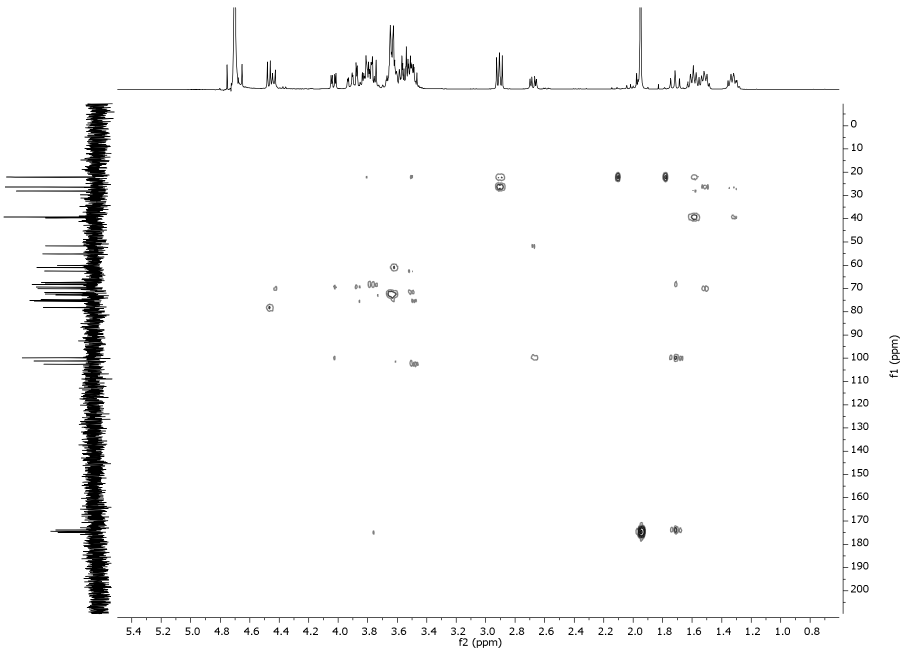

Figure S141: 1H-13C HMBC NMR of compound H3.

5. References

1) Wang Z, Zhou L, El-Boubbou K, Ye XS, Huang X. Multi-component one-pot synthesis of the tumor-associated carbohydrate antigen Globo-H based on preactivation of thioglycosyl donors. *J Org Chem*. 2007, 72(17):6409-20.
2) Mong TK, Huang CY, Wong CH. A new reactivity-based one-pot synthesis of N-acetyllactosamine oligomers. *J Org Chem*. 2003. 68(6), 2135-42.
3) Hsu CH, Chu KC, Lin YS, Han JL, Peng YS, Ren CT, Wu CY, Wong CH. Highly alpha-selective sialyl phosphate donors for efficient preparation of natural sialosides. *Chemistry*. 2010, 16(6), 1754-60.
4) Volkert, A.A., Subramaniam, V., Ivanov, M.R., Goodman, A.M., Haes, A.J., 2011. Salt-mediated self-assembly of thioctic acid on gold nanoparticles. ACS Nano. 2011, 5, 4570–4580.
